# A Galactoside-Binding Protein Tricked into Binding Unnatural Pyranose Derivatives: 3-Deoxy-3-Methyl-Gulosides Selectively Inhibit Galectin-1

**DOI:** 10.3390/ijms20153786

**Published:** 2019-08-02

**Authors:** Kumar Bhaskar Pal, Mukul Mahanti, Hakon Leffler, Ulf J. Nilsson

**Affiliations:** 1 Centre for Analysis and Synthesis, Department of Chemistry, Lund University, Box 124, SE-221 00 Lund, Sweden; 2 Section MIG, Department of Laboratory Medicine, Lund University, BMC-C1228b, Klinikgatan 28, SE-221 84 Lund, Sweden

**Keywords:** galectin-1, gulopyranosides, fluorescence polarization, benzamide, selective

## Abstract

Galectins are a family of galactoside-recognizing proteins involved in different galectin-subtype-specific inflammatory and tumor-promoting processes, which motivates the development of inhibitors that are more selective galectin inhibitors than natural ligand fragments. Here, we describe the synthesis and evaluation of 3-*C*-methyl-gulopyranoside derivatives and their evaluation as galectin inhibitors. Methyl 3-deoxy-3-*C*-(hydroxymethyl)-β-d-gulopyranoside showed 7-fold better affinity for galectin-1 than the natural monosaccharide fragment analog methyl β-d-galactopyranoside, as well as a high selectivity over galectin-2, 3, 4, 7, 8, and 9. Derivatization of the 3-*C*-hydroxymethyl into amides gave gulosides with improved selectivities and affinities; methyl 3-deoxy-3-*C*-(methyl-2,3,4,5,6-pentafluorobenzamide)-β-d-gulopyranoside had K_d_ 700 µM for galectin-1, while not binding any other galectin.

## 1. Introduction

Galectins are an evolutionary ancient family of small soluble proteins with affinity for β-d-galactopyranoside-containing glycoconjugates and a conserved amino acid sequence motif [1,2]. By their carbohydrate-binding activity they can cross-link glycoproteins, resulting in a variety of effects, such as regulation of cell adhesion, intracellular glycoprotein traffic, and cell signaling [3,4,5]. These effects in turn affect cell behavior in inflammation, immunity and cancer, and galectins appear to be rate limiting in some such pathophysiological conditions, e.g., based on effects in null mutant mice and other model systems [6,7,8,9,10]. This has stimulated development of galectin inhibitors as potential drug candidates, but different galectins have a different tissue distribution and function. Although all bind glycoconjugates containing β-galactose residues, each galectin may have a different affinities for larger natural glycans and for artificial small molecule ligands. Hence, there is an important need for selective galectin-inhibitors, that, for example, distinguish between the two most studied galectins in humans, galectin-1 and galectin-3.

The carbohydrate binding site of galectins is a concave groove and long enough to hold about a tetrasaccharide and based on this the carbohydrate binding site of galectins has been described as a combination of four subsites (A–D) together with an additional one less defined fifth subsite E [3]. Within this groove, subsite C is conserved among galectins, made up of the defining amino acid sequence motif and binds β-galactopyranosides by H-bond interaction with 4-OH, 6-OH and the ring 5-O, and CH-π interaction of the α-side of the pyranose ring with a Trp residue. The neighbouring sites, however, vary among galectins, and can be targeted for selective inhibitor development. To do this, previous inhibitor design has derivatized the positions on galactose not engaged by subsite C, namely C1, C2, and C3 [11]. Gulose is a rare saccharide not found in mammals, but can potentially bind galectins because it is structurally similar to galactose with the only difference being the stereoconfiguration at C3. Hence, the C3 is epimeric with the OH axial instead of equatorial in the galectin bound pyranose form. Here, we show that derivatization at C3 in gulose offers a new space for galectin inhibitor design and surprisingly selective inhibitors of galectin-1. In particular, amide-functionalised C3-methyl gulopyranosides are shown to be apparently selective towards human galectin-1.

## 2. Results and Discussion

### 2.1. Synthesis of Methyl 3-Deoxy-3-C-(methyl)-β-d-gulopyranosides and galectin inhibition evaluation

**Figure 1:**
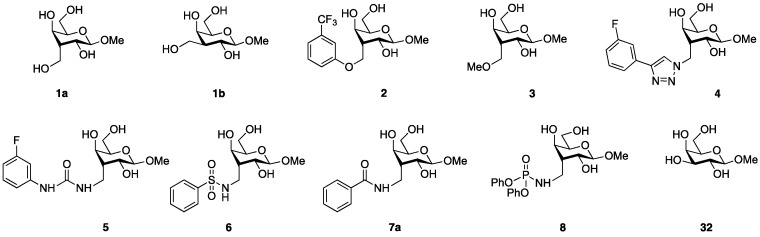
Structures of the tested compounds **1**–**8** and reference compound **32**.

**Scheme 1:**
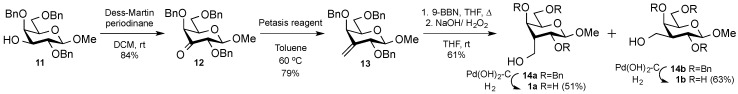
Synthesis of methyl 3-deoxy-3-*C*-(hydroxymethyl)-*β*-d-gulopyranoside **1a**, methyl 3-deoxy-3-*C*-(hydroxymethyl)-*β*-d-galactopyranoside **1b**.

The synthesis of the 3-*C*-methyl-gulo derivatives was initiated by Dess–Martin periodinane oxidation [12] of the known methyl 2,4,6-tri-*O-*benzyl-β-d-galactopyranoside **11** to afford the corresponding keto derivative **12** in 84% yield (Scheme 1). Methylenation of **12** with Petasis reagent gave the olefin **13** in 79% yield. Next, the olefin **13** was subjected to hydroboration with 9-borabicyclo-[3.3.1]nonane (9-BBN) [12], followed by oxidative cleavage of the carbon-boron bond with alkaline hydrogen peroxide to afford the corresponding gulo and galacto isomers **14a** (36%) and **14b** (24%), which were separated by flash column chromatography at a ratio 3:2. Both the gulo and galacto derivatives **14a** and **14b** were separately subjected to hydrogenation [13] in the presence of Pd(OH)_2_-C to give the desired methyl 3-deoxy-3-*C*-(hydroxymethyl)-*β*-d-gulopyranoside **1a** and methyl 3-deoxy-3-*C*-(hydroxymethyl)-*β*-d-galactopyranoside **1b** in yields of 51% and 63%, respectively. Evaluation of **1a** and **1b** as inhibitors of human galectin-1, 2, 3, 4N (N-terminal domain) 4C (C-terminal domain), 7, 8N, 8C, 9N, and 9C in a reported competitive fluorescence anisotropy assay [14,15] revealed that the gulo derivative **1a** was selective for galectin-1 with a dissociation constant of 1300 µM, which is about an order of magnitude better than for the virtually unselective reference compound methyl β-d-galactopyranoside **32** (Figure 1, Table 1).

**Scheme 2:**
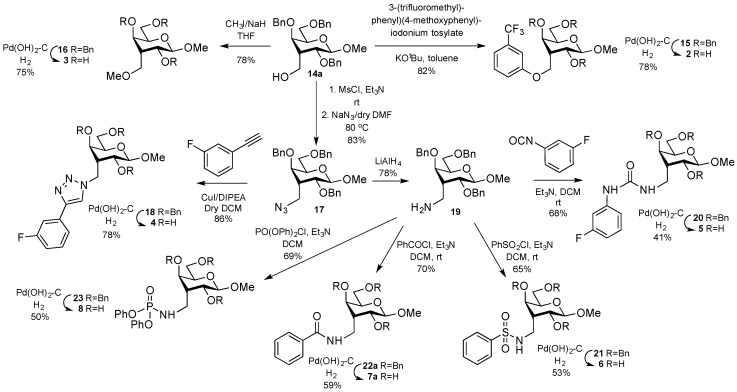
Synthesis of methyl 3-deoxy-3-*C*-methyl-*β*-d-gulopyranoside ether **2**–**3**, triazole **4**, urea **5**, sulfonamide **6**, amide **7a**, and phosphonamide **8** derivatives.

In stark contrast, the galacto derivative **1b** did not bind any galectin tested, except for a weak binding to galectin-4N. This observation encouraged us to further explore the 3-*C*-methyl gulopyranoside scaffold for the discovery of galectin-1-selective inhibitors. Hence, we initiated synthetic efforts toward replacing the hydroxymethyl of **1a** with amide, ether, urea, and triazole functionalities. An aryl ether was synthesized following a recently reported iodonium-salt mediated reaction [20] to give the aryl ether **15**, which after hydrogenolysis [13] of the benzyl protecting groups gave **2** (Scheme 2). The hydroxymethyl **14a** was methylated with methyl iodide to give the methyl ether **16**, which after debenzylation gave the 3-methoxymethyl guloside **3**. Treatment of **14a** with methanesulfonyl chloride furnished the corresponding gulo mesylate, which was then directly treated with NaN_3_ in dry DMF at 80 °C to provide the gulo azide, **17** in 83% yield. The gulo azide **17** was treated with 1-ethynyl-3-fluorobenzene in the presence of the CuI and DIPEA catalytic system [21] in dry dichloromethane to give the triazole **18** within 48 h in 86% yield. Debenzylation provided the desired triazole-derived methyl guloside **4**. The urea **20** was obtained via reduction of the azide **17** to give the amine **19**, followed by reaction with 3-fluorophenylisocyanate. Debenzylation [13] of **20** afforded the target gulo urea derivative **5** in 66% yield. The amine **19** was treated with benzensulfonyl chloride, benzoyl chloride, and diphenyl phosphoryl chloride in the presence of Et_3_N to give the protected sulfonamide **21**, amide **22a**, and diphenylphosphonamide **23**, which were subjected for hydrogenolysis [13] in the presence of Pd(OH)_2_ to get the unprotected amides **6**, **7a**, and **8**.

Evaluation of aryl ether **2**, methyl ether **3**, triazole **4**, urea **5**, sulfonamide **6**, benzamide **7a**, and phosphonamide **8** derived methyl gulosides’ affinities for the human galectin-1, 2, 3, 4N, 4C, 7, 8N, 8C, 9N, and 9C showed that most of the gulo derivatives were inactive as ligands for galectins, the benzamide **7a** displayed moderate affinity, similar to that of **1a**, for galectin-1 and with excellent selectivity (Table 1). Particularly noteworthy was that **7a** also had a significantly better affinity for galectin-1 than the simple reference monosaccharide methyl β-d-galactopyranoside **32**. Furthermore, the hydroxylmethyl group of **1a** plays an important role in the interaction with galectin-1, as the corresponding methyl ether **3** binds galectin-1 significantly worse than **1a** does.

### 2.2. Synthesis and Optimization of 3-Deoxy-3-C-Amidomethyl-β-d-Gulopyranoside Derivatives as Galectin-1 Inhibitors

**Scheme 3:**
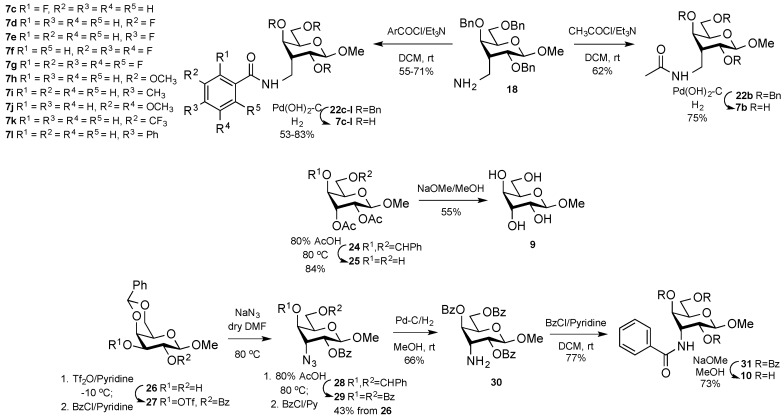
Synthesis of 3-deoxy-3-*C*-amidomethyl-*β*-d-gulo derivatives **7b**–**7l**, methyl *β*-d-gulopyranoside **9**, and methyl 3-deoxy-3-*N*-benzamido-*β*-d-gulopyranoside **10**.

The observation that the amide **7a** showed moderate affinity but high selectivity for galectin-1 prompted us to prepare a series **7c**–**7l** of benzamide analogs carrying selected different substituents at different positions, including four fluorbenzamide expected to possess improved metabolic stability and pharmacokinetic properties, as well as a reference acetamide analog **7b** (Scheme 3). Furthermore, in order to investigate the role of the gulo 3-*C*-methyl substituent, the 3-OH **9** and 3-benzamido **10** gulosides were synthesized (Scheme 3). Hydrolysis of the known 4,6-*O*-benzylidene gulose derivative, **24** [22] with 80% AcOH at 80 °C gave the diol **25** in 91% yield, which upon Zemplen de-*O*-acetylation [23] afforded the target methyl *β*-d-gulopyranoside **9** in 93% yield. Selective 3-*O*-triflation of methyl 4,6-*O*-benzylidene-*β*-d-galactopyranoside **26 [24]**, followed by one-pot benzoylation of 2-*O*-hydroxyl gave **27**. The crude triflate **27** was subsequently converted into the 3-azido-3-deoxy-guloside **28** by treatment with sodium azide in DMF. De-benzylidenation with 80% AcOH at 80 °C and subsequent benzoylation afforded **29** in 43% yield over four steps from **26**. Azide hydrogenation gave **30**, which upon benzoylation and de-*O*-benzoylation gave the benzamide **10**.

**Figure 2:**
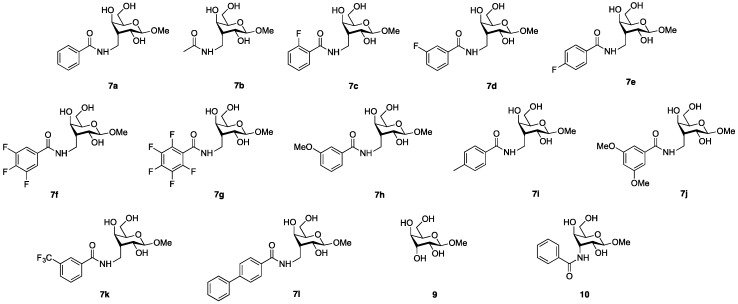
Structures of all tested compounds **7a**–**7l** and **9**–**10**.

An immediate observation upon evaluating the affinities of **7b**–**7l** and **9**–**10** (Figure 2, Table 2) was that the acetamide **7b** displays a similar affinity for galectin-1 as the benzamides **7a** and **7c**–**7k**. Hence, the phenyl moieties of **7a** and **7c**–**7k** do not contribute to enhancing the affinity for galectin-1. However, the phenyl moieties and substitution patterns of **7a** and **7c**–**7k** influence the selectivity over other galectins, as six substituted amides (**7a**, **7d**, and **7f**–**7i**) retained high selectivity for galectin-1 over the other galectins. The pentafluorophenyl **7g** turned out to be the best β-d-gulopyranoside-based monosaccharide inhibitor for human galectin-1 with 14-fold improved affinity over the reference methyl β-d-galactopyranoside **32**. The larger biphenyl **7l** did not bind galectin-1, which suggests that the galectin-1 site accommodating the axial gulo substituent is limited in size. Evaluation of the guloside **9** revealed that while it is similar to the reference galactoside **32** in the affinity for galectin-1, it displays a much higher selectivity in that it is inactive against the other galectins under the evaluation conditions used. Unfortunately, extensive molecular dynamics and docking analyses to explain the selective galectin-1 binding to 3-*C*-methyl-gulosides were inconclusive as such calculations cannot provide reliable relative affinities of bound ligands. Hence, it remains to find a plausible structural explanation for this selectivity. Interestingly, the benzamide **10** showed no binding to galectin-1 under the assay conditions but instead had improved binding to and selectivity for galectin-4N. Hence, while 3-*C*-methyl gulosides represent an interesting structural class for the discovery of selective galectin-1 inhibitors, 3-*C*-amido gulosides may represent a novel structural class for galectin-4 inhibitor discovery.

## 3. Materials and Methods

### 3.1. General Methods Experimental Procedures

All reactions were carried out in oven-dried glassware. All solvents and reagents were mainly purchased from Sigma-Aldrich or Fluka and were used without further purification or synthesized via the literature protocol. TLC analysis was performed on pre-coated Merck silica gel 60 F_254_ plates using UV light and charring solution (10 mL conc. H_2_SO_4_/90 mL EtOH). Flash column chromatography was done on SiO_2_ purchased from Aldrich (technical grade, 60 Å pore size, 230–400 mesh, 40–63 μm). All NMR spectra were recorded with the Bruker DRX 400 MHz spectrometer (400 MHz for ^1^H, 100 MHz for ^13^C (125 MHz ^13^C for compound **7k** with the Bruker Avance III 500 MHz spectrometer equipped with a broadband observe SMART probe, Fällanden, Switzerland), 376 MHz for ^19^F, 162 MHz for ^31^P, ESI) at ambient temperature using CDCl_3_ or CD_3_OD as solvents. Chemical shifts are given in ppm relative to the residual solvent peak (^1^H NMR: CDCl_3_ δ 7.26; CD_3_OD δ 3.31; ^13^C NMR: CDCl_3_ δ 77.16; CD_3_OD δ 49.00) with multiplicity (*b* = broad, *s* = singlet, *d* = doublet, *t* = triplet, *q* = quartet, *quin* = quintet, *sext* = sextet, *hept* = heptet, *m* = multiplet, *td* = triplet of doublets, *dt* = doublet of triplets), coupling constants (in Hz) and integration. Copies of nmr spectra are provided in the supplementary information. High-resolution mass analysis was obtained using the Micromass Q-TOF mass spectrometer. Analytical data is given if the compound is novel or not fully characterized in the literature. Final compounds were further purified via HPLC before evaluation of galectin affinity. All tested compounds were >95% pure according to the analytical HPLC analysis.

### 3.2. Methyl 2,4,6-Tri-O-Benzyl-β-d-Xylo-Hex-3-Ulopyranoside ***12***

Into a solution of alcohol **11** (8.1 g, 17.45 mmol) in dry dichloromethane (250 mL) Dess–Martin periodinane (9.62 g, 22.68 mmol, 1.3 equiv.) was added, under nitrogen atmosphere and the reaction mixture was stirred for 4 h (TLC heptane/EtOAc, 3:1, R_f_ 0.5). After that, a saturated NaHCO_3_ solution (400 mL) was added and the mixture was stirred for 30 min. Then, the organic layer was collected and washed successively with the saturated Na_2_S_2_O_3_ solution (2 × 250 mL). The organic layer was collected, dried over Na_2_SO_4_, filtered and concentrated in vacuo. Flash chromatography of the crude material (heptane/EtOAc, 7:2) afforded ketone **12** (6.45 g, 13.955 mmol, yield 80%) as a white solid. ${\left[\alpha \right]}_{D}^{25}$ −72.3 (c 1.4, CHCl_3_). ^1^H NMR (CDCl_3_, 400 MHz): 7.47–7.21 (m, 15H, Ar*H*), 4.76 (d, 1H, *J* 12.0 Hz, C*H*_2_Ph), 4.73 (d, 1H, *J* 12.0 Hz, C*H*_2_Ph), 4.58 (d, 1H, *J* 12.0 Hz, C*H*_2_Ph), 4.51 (d, 1H, *J* 12.0 Hz, C*H*_2_Ph), 4.48 (d, 1H, *J*_1,2_ 7.6 Hz, H-1), 4.44 (d, 1H, *J*_1,2_ 7.6 Hz, H-2), 4.43 (d, 1H, *J* 11.6 Hz, C*H*_2_Ph), 4.35 (d, 1H, *J* 11.6 Hz, C*H*_2_Ph), 3.95 (d, 1H, *J* 1.2 Hz, H-4), 3.83–3.75 (m, 3H, H-5, H-6a, H-6b), 3.61 (s, 3H, OC*H*_3_). ^13^C NMR (CDCl_3_, 100 MHz): 203.8, 137.7, 137.2, 136.4, 128.29, 128.27, 127.26, 128.0, 127.8, 127.6, 127.5, 104.9, 82.1, 80.7, 73.5, 73.4, 72.3, 72.1, 67.5, 57.1. HRMS calcd for C_28_H_30_O_6_^+^NH_4_^+^ (M+NH_4_)^+^: 480.2386, found: 480.2378.

### 3.3. Methyl 2,4,6-Tri-O-Benzyl-3-Deoxy-3-C-Methylene-β-d-Xylo-Hex-3-Ulopyranoside ***13***

Into a solution of ketone **12** (6.3 g, 13.63 mmol) in dry toluene (100 mL) bis (cyclopentadienyl) dimethyltitanium was added, 5 wt% in toluene (125 mL, 30 mmol, 2.2 equiv.), under nitrogen atmosphere and the reaction mixture was stirred for 48 h at 65 °C in the dark. After that, the reaction mixture (TLC heptane/EtOAc, 4:1, R_f_ 0.47) was concentrated in vacuo and flash chromatography of the crude material (heptane/EtOAc, 10:1–5:1) afforded methylene derivative **13** (4.6 g, 9.99 mmol, yield 71%) as a light-yellow oil. ${\left[\alpha \right]}_{D}^{25}$ −40.3 (c 1.1, CHCl_3_). ^1^H NMR (CDCl_3_, 400 MHz): 7.49–7.28 (m, 15H, Ar*H*), 5.61 (t, 1H, *J*_2,H-7a_ 2.0 Hz, C*H*_2_), 5.20 (t, 1H, *J*_2,H-7b_ 2.0 Hz, C*H*_2_), 5.00 (d, 1H, *J* 12.0 Hz, C*H*_2_Ph), 4.78 (d, 1H, *J* 12.0 Hz, C*H*_2_Ph), 4.65 (d, 1H, *J* 12.0 Hz, C*H*_2_Ph), 4.58 (d, 1H, *J* 12.0 Hz, C*H*_2_Ph), 4.56 (d, 1H, *J* 12.0 Hz, C*H*_2_Ph), 4.36 (d, 1H, *J* 7.6 Hz, H-1), 4.28 (d, 1H, *J* 12.0 Hz, C*H*_2_Ph), 4.14 (dt, 1H, *J*_1,2_ 7.6 Hz, *J*_2,H-7a_, *J*_2,H-7b_ 2.0 Hz, H-2), 4.03 (d, 1H, *J* 0.4 Hz, H-4), 3.91–3.79 (m, 3H, H-5, H-6a, H-6b), 3.65 (s, 3H, OC*H*_3_). ^13^C NMR (CDCl_3_, 100 MHz): 142.2, 138.5, 138.2, 137.9, 128.4, 128.3, 128.2, 128.0, 127.9, 127.7, 127.62, 127.59, 127.5, 113.7, 104.9, 77.7, 77.3, 76.6, 73.7, 73.6, 69.2, 69.0, 56.7. HRMS calcd for C_29_H_32_O_5_+NH_4_^+^ (M+NH_4_)^+^: 478.2593, found: 478.2607.

### 3.4. Methyl 2,4,6-Tri-O-Benzyl-3-Deoxy-3-C-Hydroxymethyl-β-d-Gulopyranoside ***14a*** and Methyl 2,4,6-Tri-O-Benzyl-3-Deoxy-3-C-Hydroxymethyl-β-d-Galactopyranoside ***14b***

A solution of **13** (4.6 g, 9.99 mmol) in dry THF (150 mL) was treated with a 9-BBN solution in THF (0.5 M, 125 mL) and heated to reflux for 24 h. After that, the solution was cooled to 0 °C and a 10% aqueous sodium hydroxide solution (100 mL) and a 30% hydrogen peroxide solution (100 mL) were added simultaneously within 5 min and stirring continued for another 30 min. Then, diethyl ether (200 mL) was added followed by careful addition of a 20% aqueous sodium hydrogen sulfite solution (7 mL). This mixture was stirred further for 60 min and extracted with diethyl ether, and the combined organic layers were dried with Na_2_SO_4_, filtered, and concentrated in vacuo (TLC heptane/EtOAc, 2:1 (double run), R_f_ 0.48 for **14a**, R_f_ 0.4 for **14b**). Flash chromatography (Heptane/EtOAc, 8:1 to 2:1) of the residue afforded a gulo-isomer, **14a** (1.74 g, 3.638 mmol) and galacto-isomer, **14b** (1.16 g, 2.426 mmol) to be ≈3:2 in favor of the guloisomer at an overall yield of 61% (2.9 g, 6.064 mmol). Gulo-isomer **14a**: ${\left[\alpha \right]}_{D}^{25}$ −25.7 (c 1.3, CHCl_3_). ^1^H NMR (CDCl_3_, 400 MHz): 7.36–7.20 (m, 15H, Ar*H*), 4.82 (d, 1H, *J* 12.0 Hz, C*H*_2_Ph), 4.65 (d, 1H, *J* 6.4 Hz, H-1), 4.57 (d, 1H, *J* 11.6 Hz, C*H*_2_Ph), 4.54 (d, 1H, *J* 11.6 Hz, C*H*_2_Ph), 4.52 (d, 1H, *J* 11.6 Hz, C*H*_2_Ph), 4.47 (d, 1H, *J* 12.0 Hz, C*H*_2_Ph), 4.41 (d, 1H, *J* 11.6 Hz, C*H*_2_Ph), 3.95–3.88 (m, 2H, H-4, H-5), 3.73 (dd, 1H, *J*_1,2_ 6.4 Hz, *J*_2,3_ 5.2 Hz, H-2), 3.74–3.57 (m, 4H, H-6a, H-6b, C*H*_2_OH), 3.54 (s, 3H, OC*H*_3_), 2.53–2.47 (m, 1H, H-3), 2.35 (bs, 1H, CH_2_O*H*). ^13^C NMR (CDCl_3_, 100 MHz): 138.2, 138.1, 138.0, 128.6, 128.52, 128.47, 128.2, 128.1, 128.03, 127.96, 127.9, 127.8, 101.2, 77.2, 74.8, 73.8, 73.6, 73.4, 71.9, 69.5, 62.0, 56.5, 41.6. HRMS calcd for C_29_H_34_O_6_+NH_4_^+^ (M+NH_4_)^+^: 496.2699, found: 496.2700. Galacto-isomer **14b**: ${\left[\alpha \right]}_{D}^{25}$ −13.4 (c 0.9, CHCl_3_). ^1^H NMR (CDCl_3_, 400 MHz): 7.39–7.28 (m, 15H, Ar*H*), 4.92 (d, 1H, *J* 11.2 Hz, C*H*_2_Ph), 4.65 (d, 1H, *J* 11.2 Hz, C*H*_2_Ph), 4.60–4.52 (m, 4H, C*H*_2_Ph), 4.41 (d, 1H, *J*_1,2_ 7.6 Hz, H-1), 3.90(d, 1H, *J*_3,4_ 2.8 Hz, H-4), 3.82 (dd, 1H, *J* 4.8 Hz, *J* 7.2 Hz, C*H*_2_OH), 3.73–3.55 (m, 8H, H-5, H-6a, H-6b, H-2, C*H*_2_OH, OC*H*_3_), 2.04 (bs, 1H, CH_2_O*H*), 1.87–1.82 (m, 1H, H-3). ^13^C NMR (CDCl_3_, 100 MHz): 138.5, 138.1, 137.8, 128.6, 128.53, 128.52, 128.4, 128.3, 128.03, 127.98, 127.8, 106.4, 76.5, 76.2, 74.8, 74.7, 74.6, 73.7, 68.6, 62.2, 56.8, 47.3. HRMS calcd for C_29_H_34_O_6_+H^+^ (M+H)^+^: 479.2434, found: 479.2434.

### 3.5. Methyl 2,4,6-Tri-O-Benzyl-3-Deoxy-3-C-(3-Trifluoromethylphenoxymethyl)-β-d-Gulopyranoside ***15***

Compound **14a** (80 mg, 0.17 mmol) was stirred in a 25 mL round-bottom flask in toluene (2 mL) for 3 min. A mixture of 3-(trifluoromethyl)phenyl)(4-methoxyphenyl)iodonium tosylate (140 mg, 0.25 mmol) and potassium tert-butoxide (28.5 mg, 0.25 mmol) were added under air and the mixture turned yellow. The reaction was stirred for 3 h, when the TLC showed almost complete consumption of the starting material (TLC heptane/EtOAc, 3:1, R_f_ 0.48). The mixture was then diluted with EtOAc (10 mL) and filtered. Then the volatiles were removed under reduced pressure, and the residue was subjected to column chromatography (heptane/EtOAc, 8:1 to 4:1) to provide the purified product **15** (92.6 mg, 0.15 mmol, 89%) as a colorless oil. ${\left[\alpha \right]}_{D}^{25}$ −70.9 (c 0.8, CHCl_3_). ^1^H NMR (CDCl_3_, 400 MHz): 7.40–7.22 (m, 17H, Ar*H*), 7.08 (bs, 1H, Ar*H*), 7.02 (dd, 1H, *J* 8.0 Hz, *J* 2.4 Hz, Ar*H*), 4.79 (d, 1H, *J* 12.0 Hz, C*H*_2_Ph), 4.62 (d, 1H, *J* 6.0 Hz, H-1), 4.58 (d, 1H, *J* 12.0 Hz, C*H*_2_Ph), 4.57 (d, 1H, *J* 11.6 Hz, C*H*_2_Ph), 4.54 (d, 1H, *J* 12.4 Hz, C*H*_2_Ph), 4.50 (s, 2H, C*H*_2_Ph), 4.24 (dd, 1H, *J* 6.0 Hz, *J* 9.6 Hz, H-3a*’*), 4.19–4.15 (m, 1H, H-5), 4.08 (dd, 1H, *J* 9.6 Hz, *J* 8.0 Hz, H-3b*’*), 3.87 (dd, 1H, *J* 5.2 Hz, *J* 2.8 Hz, H-4), 3.85 (t, 1H, *J* 6.0 Hz, H-2), 3.80 (dd, 1H, *J* 10.0 Hz, *J* 6.8 Hz, H-6a), 3.72 (dd, 1H, *J* 10.0 Hz, *J* 5.2 Hz, H-6b), 3.57 (s, 1H, OC*H*_3_), 2.76–2.70 (m, 1H, H-3). ^13^C NMR (CDCl_3_, 100 MHz): 158.8, 138.30, 138.27, 137.9, 131.9 (q, *J* 32.1 Hz), 130.0, 128.49, 128.46, 128.3, 128.0, 127.9, 127.83, 127.77, 124.1 (q, *J* 271 Hz), 118.0, 117.6 (q, *J* 3.8 Hz), 111.5 (q, *J* 3.7 Hz), 101.3, 74.7, 73.6, 73.5, 73.3, 72.8, 71.9, 69.8, 64.6, 56.4, 39.6. ^19^F NMR (CDCl_3_, 376 MHz): −62.6. HRMS calcd for C_36_H_41_F_3_NO_6_+NH_4_^+^ (M+NH_4_)^+^: 640.2886, found: 640.2895.

### 3.6. Methyl 2,4,6-Tri-O-Benzyl-3-Deoxy-3-C-Methoxymethyl-β-d-Gulopyranoside ***16***

Compound **14a** (57 mg, 0.12 mmol) was stirred in a 5 mL round-bottom flask in dry THF (2 mL) for 5 min at 0 °C. Into the solution, NaH (6 mg, 0.24 mmol) was added and the stirring was continued at 0 °C for 5 min. Then, into the reaction mixture iodomethane dropwise was added and the reaction temperature increased to rt gradually. Stirring continued overnight when the TLC showed almost complete consumption of the starting material (TLC heptane/EtOAc, 3:2, R_f_ 0.53). Then, NaH was quenched with EtOAc and the volatiles were removed under reduced pressure. The residue was subjected to column chromatography (heptane/EtOAc, 6:1 to 3:1) to provide the purified product **16** (46 mg, 0.09 mmol, 78%). ${\left[\alpha \right]}_{D}^{25}$ −62.5 (c 1.2, CHCl_3_). ^1^H NMR (CDCl_3_, 400 MHz): 7.35–7.20 (m, 15H, Ar*H*), 4.76 (d, 1H, *J* 12.0 Hz, C*H*_2_Ph), 4.58 (d, 1H, *J* 12.0 Hz, C*H*_2_Ph), 4.57 (d, 1H, *J* 11.6 Hz, C*H*_2_Ph), 4.53 (d, 1H, *J*_1,2_ 6.4 Hz, H-1), 4.48 (d, 2H, *J* 12.4 Hz, C*H*_2_Ph), 4.37 (d, 1H, *J* 11.6 Hz, C*H*_2_Ph), 4.11–4.07 (m, 1H, H-5), 3.75–3.71 (m, 3H, H-2, H-4, H-6a), 3.67–3.61 (m, 2H, H-6b, C*H*_2_OCH_3_), 3.56–3.50 (m, 4H, C*H*_2_OCH_3_, OC*H*_3_), 3.29 (s, 3H, CH_2_OC*H*_3_), 2.58–2.52 (m, 3H, H-3). ^13^C NMR (CDCl_3_, 100 MHz): 138.8, 138.43, 138.39, 128.5, 128.43, 128.36, 128.1, 127.94, 128.90, 127.8, 127.74, 127.69, 127.66, 101.8, 75.1, 74.7, 73.7, 73.3, 73.2, 71.8, 70.2, 69.2, 59.0, 56.4, 40.2. HRMS calcd for C_17_H_21_NO_6_+H^+^ (M+H)^+^: 335.1369, found: 335.1369.

### 3.7. Methyl 2,4,6-Tri-O-Benzyl-3-Deoxy-3-C-Azidomethyl-β-d-Gulopyranoside ***17***

Into a stirred solution of **14a** (1.6 g, 3.35 mmol) in DCM (25 mL) containing Et_3_N (890 μL, 6.69 mmol) at 0 °C MsCl (390 μL, 5.02 mmol) was added dropwise over 5 min, and the solution was stirred for 4 h at rt (TLC heptane/EtOAc, 1:1, R_f_ 0.31). The solution was extracted with 1N HCl (2 × 50 mL) followed by sat’d NaHCO_3_ (2 × 50 mL), and the organic layer was dried (Na_2_SO_4_). The solvent was removed by rotary evaporation to give a yellow liquid that was dissolved in dry DMF (10 mL). Sodium azide (1.3 g, 20.08 mmol) was added and the solution was heated at 80 °C for 6 h to give a yellowish-brown mixture. The mixture was cooled at rt, water (50 mL) was added, and the mixture was extracted with EtOAc (2 × 40 mL). The organic layer was washed with brine (50 mL) and dried (Na_2_SO_4_). The solvent was removed by rotary evaporation to give a yellow liquid that was then purified by flash chromatography (Heptane/EtOAc 8:1 to 3:1) to give compound **17** (1.4 g, 2.78 mmol, 83% from **14a**) as a colorless liquid. ${\left[\alpha \right]}_{D}^{25}$ −5.2 (c 0.8, CHCl_3_). ^1^H NMR (CDCl_3_, 400 MHz): 7.38–7.20 (m, 15H, Ar*H*), 4.76 (d, 1H, *J* 12.0 Hz, C*H*_2_Ph), 4.56 (d, 1H, *J* 12.0 Hz, C*H*_2_Ph), 4.55 (d, 1H, *J* 11.6 Hz, C*H*_2_Ph), 4.51 (d, 1H, *J*_1,2_ 6.4 Hz, H-1), 4.48 (d, 1H, *J* 12.4 Hz, C*H*_2_Ph), 4.44 (d, 1H, *J* 12.0 Hz, C*H*_2_Ph), 4.41 (d, 1H, *J* 12.0 Hz, C*H*_2_Ph), 4.09–4.05 (m, 1H, H-5), 3.77–3.64 (m, 5H, H-2, H-4, H-6a, H-6b, C*H*_2_N_3_), 3.50 (s, 3H, OC*H*_3_), 3.39 (dd, 1H, *J* 12.4 Hz, *J* 8.4 Hz, C*H*_2_N_3_), 2.42–2.36 (m, 1H, H-3). ^13^C NMR (CDCl_3_, 100 MHz): 138.4, 138.3, 137.9, 128.53, 128.52, 128.50, 128.2, 128.02, 127.95, 127.9, 127.8, 100.8, 75.0, 73.7, 73.6, 73.4, 72.5, 72.0, 69.6, 56.4, 48.6, 39.7. HRMS calcd for C_29_H_33_N_3_O_5_+NH_4_^+^ (M+NH_4_)^+^: 521.2764, found: 521.2775.

### 3.8. Methyl 2,4,6-Tri-O-Benzyl-3-Deoxy-3-C-[4-(3-Fluorophenyl)-1H-1,2,3-Triazol-1-Yl-Methyl]-β-d-Gulopyranoside ***18***

A solution of azide **17** (53 mg, 0.10 mmol) in dichloromethane (2 mL), 1-Ethynyl-3-fluorobenzene (18.1 μL, 0.16 mmol), CuI (10 mol%, 2 mg) and DIPEA (28 μL, 0.16 mmol) were added and the mixture was stirred at room temperature for 48 h (TLC heptane/EtOAc, 2:1, R_f_ 0.58). The solvent was removed under reduced pressure, and the residue was dissolved in EtOAc (10 mL) and the solution was washed with sat. NH_4_Cl (10 mL), brine (10 mL), dried over Na_2_SO_4_ and concentrated in vacuo. The product was purified by flash column chromatography (heptane/EtOAc, 6:1 to 1:1) to give the corresponding triazole, **18** as white amorphous solid (56.4 mg, 0.09 mmol, 86%). ${\left[\alpha \right]}_{D}^{25}$ −63 (c 0.6, CHCl_3_). ^1^H NMR (CDCl_3_, 400 MHz): 7.52–7.04 (m, 20H, Ar*H*), 4.80 (d, 1H, *J* 11.6 Hz, C*H*_2_Ph), 4.63 (dd, 1H, *J* 6.8 Hz, *J* 14.0 Hz, H-3′), 4.60–4.44 (m, 7H, H-1, H-3′, C*H*_2_Ph), 4.35 (d, 1H, *J* 11.6 Hz, C*H*_2_Ph), 4.26–4.22 (m, 1H, H-5), 3.79 (dd, 1H, *J* 10.0 Hz, *J* 7.2 Hz, H-6a), 3.74 (dd, 1H, *J* 6.4 Hz, *J* 3.2 Hz, H-4), 3.71 (dd, 1H, *J* 10.0 Hz, *J* 5.2 Hz, H-6b), 3.79 (t, 1H, *J* 4.8 Hz, H-2), 3.51 (s, 1H, OC*H*_3_), 2.68–2.61 (m, 1H, H-3). ^13^C NMR (CDCl_3_, 100 MHz): 163.3 (d, *J* 244 Hz), 146.8, 138.19, 138.16, 137.4, 132.8 (d, *J* 8.3 Hz), 130.5 (d, *J* 8.4 Hz), 128.7, 128.52, 128.51, 128.3, 128.2, 128.0, 127.9, 127.8, 121.3 (d, *J* 2.7 Hz), 120.6, 115.0 (d, *J* 22 Hz), 112.7 (d, *J* 23 Hz), 100.1, 75.2, 73.52, 73.47, 73.1, 72.5, 72.2, 69.4, 56.4, 47.4, 41.1. ^19^F NMR (CDCl_3_, 376 MHz): −112.7. HRMS calcd for C_37_H_38_FN_3_O_5_+H^+^ (M+H)^+^: 624.2874, found: 624.2884.

### 3.9. Methyl 2,4,6-Tri-O-Benzyl-3-Deoxy-3-C-(Aminomethyl)-β-d-Gulopyranoside ***19***

Into a stirred solution of **17** (1.31 g, 2.60 mmol) in dry THF (20 mL) at 0 °C, LiAlH_4_ (148 mg, 3.9 mmol) was added in portions over 5 min under nitrogen atmosphere, and the solution was stirred for 1 h at rt (TLC DCM/MeOH, 15:1, R_f_ 0.44). After 30 min, TLC was checked which shows complete conversion of the azide into amine. Then, the reaction was quenched EtOAc and the reaction mixture was filtered through a pad of Celite^®^ (St. Louis, MO, USA). Then, the filtrate was concentrated in vacuo and the crude was purified by column chromatography (DCM:MeOH 25:1) to give compound **19** (969 mg, 2.03 mmol, yield 78%) as a colorless oil. ${\left[\alpha \right]}_{D}^{25}$ −36.2 (c 1.1, CHCl_3_). ^1^H NMR (CDCl_3_, 400 MHz): 7.35–7.22 (m, 15H, Ar*H*), 4.80 (d, 1H, *J* 12.0 Hz, C*H*_2_Ph), 4.57–4.54 (m, 3H, H-1, C*H*_2_Ph), 4.51 (d, 1H, *J* 12.0 Hz, C*H*_2_Ph), 4.47 (d, 1H, *J* 12.0 Hz, C*H*_2_Ph), 4.42 (d, 1H, *J* 11.6 Hz, C*H*_2_Ph), 3.97–3.93 (m, 1H, H-5), 3.74–3.65 (m, 4H, H-2, H-4, H-6a, H-6b), 3.52 (s, 3H, OC*H*_3_), 3.08 (dd, 1H, *J* 6.4 Hz, *J* 12.8 Hz, C*H*_2_NH_2_), 2.68 (dd, 1H, *J* 12.8 Hz, *J* 6.4 Hz, C*H*_2_NH_2_), 2.32–2.27 (m, 1H, H-3), 1.99 (bs, 2H, CH_2_N*H*_2_). ^13^C NMR (CDCl_3_, 100 MHz): 138.6, 138.3, 138.1, 128.53, 128.49, 128.4, 128.2, 128.0, 127.9, 127.83, 127.81, 127.77, 101.3, 76.2, 75.2, 73.6, 73.4, 72.9, 71.7, 69.7, 56.4, 42.7, 39.7. HRMS calcd for C_29_H_35_NO_5_+H^+^ (M+H)^+^: 478.2593, found: 478.2603.

### 3.10. Methyl 2,4,6-Tri-O-Benzyl-3-Deoxy-3-C-(3-Fluorophenylureidomethyl)-β-d-Gulopyranoside ***20***

A solution of amine **19** (61 mg, 0.13 mmol) in dry dichloromethane (2 mL), Et_3_N (35.6 μL, 0.26 mmol) was added and the mixture was stirred at room temperature for 5 min under N_2_ atmosphere. Then into the solution phenyl isocyanate (29.2 μL, 0.26 mmol) was added and the solution was stirred at rt for 12 h (TLC heptane/EtOAc, 1:1, R_f_ 0.32). The solvent was removed under reduced pressure, and the residue was dissolved in EtOAc (10 mL) and the solution was washed with brine (10 mL), dried over Na_2_SO_4_ and concentrated in vacuo. The product was purified by flash column chromatography (heptane/EtOAc, 5:1 to 2:1) to give the corresponding semicarbazide **20** as a colorless oil (53.4 mg, 0.09 mmol, yield 68%). ${\left[\alpha \right]}_{D}^{25}$ −83.1 (c 0.8, CHCl_3_). ^1^H NMR (CDCl_3_, 400 MHz): 7.39–7.12 (m, 17H, Ar*H*), 6.85 (dd, 1H, *J* 1.2 Hz, *J* 8.0 Hz, Ar*H*), 6.71–6.66 (m, 1H, Ar*H*), 6.11 (bs, 1H, NHCON*H*C_6_H_4_F), 5.30 (bs, 1H, N*H*CONHC_6_H_4_F), 4.79 (d, 1H, *J* 11.6 Hz, C*H*_2_Ph), 4.59 (d, 1H, *J* 6.0 Hz, H-1), 4.55 (d, 1H, *J* 12.0 Hz, C*H*_2_Ph), 4.49 (d, 1H, *J* 12.4 Hz, C*H*_2_Ph), 4.48–4.42 (m, 3H, C*H*_2_Ph), 4.03–3.99 (m, 1H, H-5), 3.74–3.65 (m, 4H, H-2, H-4, H-6a, H-6b), 3.51 (s, 1H, OC*H*_3_), 3.42 (dd, 1H, *J* 14.0 Hz, *J* 5.6 Hz, C*H*_2_NHCON*H*C_6_H_4_F), 3.34 (dd, 1H, *J* 14.0 Hz, *J* 7.6 Hz, C*H*_2_NHCON*H*C_6_H_4_F), 2.42–2.36 (m, 1H, H-3). ^13^C NMR (CDCl_3_, 100 MHz): 163.3 (d, *J* 243 Hz), 154.9, 140.6 (d, *J* 11 Hz), 138.4, 138.2, 137.9, 130.2 (d, *J* 9.5 Hz), 128.8, 128.5, 128.3, 128.2, 128.0, 127.9, 127.8, 114.8 (d, *J* 2.7 Hz), 109.7 (d, *J* 21.2 Hz), 106.9 (d, *J* 26 Hz), 100.7, 75.3, 73.54, 73.49, 73.0, 72.1, 69.4, 56.5, 39.8, 39.1. ^19^F NMR (CDCl_3_, 376 MHz): −111.6. HRMS calcd for C_36_H_40_FN_2_O_6_+H^+^ (M+H)^+^: 615.2886, found: 615.2870.

### 3.11. General Procedure for the Synthesis of Amides ***21***, ***22a***–***22l***, and ***23***

To a solution of the amine (1 eq) in dry DCM (2 mL per 0.1 mmol) Et_3_N (2 eq) was added. Into the solution, acid chloride or anhydride (1.5 eq) was added and the solution was stirred at rt for 8 h. After that, 1(N) HCl solution was added to the reaction mixture and extracted with DCM and washed successively with saturated NaHCO_3_. After evaporating the solvents in vacuo, the crude material thus obtained was purified by flash chromatography using heptane–EtOAc (5:1 to 1:1) to give pure amides as colorless oils.

#### 3.11.1. Methyl 2,4,6-Tri-*O*-Benzyl-3-Deoxy-3-*C*-Phenylsulfonamidomethyl-β-d-Gulopyranoside **21**

Compound **21** (TLC heptane/EtOAc, 2:1, R_f_ 0.21) was prepared according to the general procedure 3.11 from the amine **19** (55 mg, 0.12 mmol). Obtained as a colorless oil in 65% yield (46.2 mg, 0.07 mmol). ${\left[\alpha \right]}_{D}^{25}$ −55.7 (c 0.7, CHCl_3_). ^1^H NMR (CDCl_3_, 400 MHz): 7.73–7.17 (m, 20H, Ar*H*), 5.23 (dd, 1H, *J* 5.2 Hz, *J* 6.8 Hz, CH_2_N*H*SO), 4.75 (d, 1H, *J* 11.6 Hz, C*H*_2_Ph), 4.53 (d, 1H, *J* 12.0 Hz, C*H*_2_Ph), 4.50 (d, 1H, *J* 12.0 Hz, C*H*_2_Ph), 4.45 (d, 1H, *J* 12.0 Hz, C*H*_2_Ph), 4.42 (d, 1H, *J* 6.0 Hz, H-1), 4.39 (d, 1H, *J* 11.2 Hz, C*H*_2_Ph), 4.36 (d, 1H, *J* 11.2 Hz, C*H*_2_Ph), 3.90–3.87 (m, 1H, H-5), 3.70–3.60 (m, 4H, H-2, H-4, H-6a, H-6b), 3.47 (s, 3H, OC*H*_3_), 3.17–3.10 (m, 1H, C*H*_2_NHSO), 3.00–2.93 (m, 1H, C*H*_2_NHSO), 2.41–2.35 (m, 1H, H-3). ^13^C NMR (CDCl_3_, 100 MHz): 139.7, 138.1, 137.9, 137.6, 132.6, 129.1, 128.7, 128.5, 128.2, 128.1, 127.94, 127.91, 127.8, 127.1, 100.5, 76.3, 74.8, 73.54, 73.50, 72.8, 71.8, 69.3, 56.3, 42.2, 39.1. HRMS calcd for C_35_H_39_NO_7_S+NH_4_^+^ (M+NH_4_)^+^: 635.2788, found: 635.2791.

#### 3.11.2. Methyl 2,4,6-Tri-*O*-Benzyl-3-Deoxy-3-*C*-(Benzamidomethyl)-β-d-Gulopyranoside **22a**

Compound **22a** (TLC heptane/EtOAc, 2:1, R_f_ 0.27) was prepared according to the general procedure *3.11* from the amine **19** (43 mg, 0.09 mmol). Obtained as a colorless oil in 70% yield (37 mg, 0.06 mmol). ${\left[\alpha \right]}_{D}^{25}$ −42.4 (c 0.8, CHCl_3_). ^1^H NMR (CDCl_3_, 400 MHz): 7.43–7.22 (m, 20H, Ar*H*), 7.03 (t, 1H, *J* 5.6 Hz, CH_2_N*H*CO), 4.89 (d, 1H, *J* 11.2 Hz, C*H*_2_Ph), 4.69 (d, 1H, *J*_1,2_ 6.0 Hz, H-1), 4.57 (d, 1H, *J* 12.0 Hz, C*H*_2_Ph), 4.55–4.46 (m, 4H, C*H*_2_Ph), 4.07–4.03 (m, 1H, H-5), 3.82 (dd, 1H, *J*_1,2_ 6.0 Hz, *J*_2,3_ 4.8 Hz, H-2), 3.79–3.68 (m, 4H, H-4, H-6a, H-6b, C*H*_2_NHCO), 3.60–3.53 (m, 4H, OC*H*_3_, C*H*_2_NHCO), 2.51–2.46 (m, 1H, H-3). ^13^C NMR (CDCl_3_, 100 MHz): 166.8, 138.3, 138.2, 137.8, 134.2, 131.2, 128.7, 128.53, 128.51, 128.47, 128.3, 128.2, 128.0, 127.8, 126.9, 100.9, 77.9, 75.9, 74.3, 73.5, 73.1, 72.2, 69.4, 56.5, 39.8, 39.3. HRMS calcd for C_36_H_39_NO_6_+H^+^ (M+H)^+^: 582.2856, found: 582.2851.

#### 3.11.3. Methyl 2,4,6-Tri-*O*-Benzyl-3-Deoxy-3-*C*-(Acetamidomethyl)-β-d-Gulopyranoside **22b**

Compound **22b** (TLC heptane/EtOAc, 1:1, R_f_ 0.4) was prepared according to the general procedure *3.11* from the amine **19** (49 mg, 0.10 mmol). Obtained as a colorless oil in 62% yield (33 mg, 0.06 mmol). ${\left[\alpha \right]}_{D}^{25}$ −31.6 (c 0.8, CHCl_3_). ^1^H NMR (CDCl_3_, 400 MHz): 7.39–7.21 (m, 15H, Ar*H*), 6.05 (bs, 1H, N*H*COCH_3_), 4.83 (d, 1H, *J* 11.6 Hz, C*H*_2_Ph), 4.59 (d, 1H, *J*_1,2_ 6.4 Hz, H-1), 4.55 (d, 1H, *J* 12.0 Hz, C*H*_2_Ph), 4.52–4.42 (m, 4H, C*H*_2_Ph), 3.98 (td, 1H, *J* 6.0 Hz, *J* 2.8 Hz, H-5), 3.74–3.64 (m, 4H, H-2, H-4, H-6a, H-6b), 3.53 (s, 3H, OCH_3_), 3.51–3.45 (m, 1H, C*H*_2_NHCO), 3.32–3.26 (s, 1H, C*H*_2_NHCO), 2.36–2.30 (m, 1H, H-3), 1.74 (s, 3H, NHCOC*H*_3_). ^13^C NMR (CDCl_3_, 100 MHz): 169.9, 138.4, 138.3, 137.9, 128.7, 128.5, 128.2, 128.12, 128.09, 127.94, 127.86, 127.7, 100.8, 77.1, 75.4, 73.8, 73.5, 73.0, 72.0, 69.4, 56.5, 39.7, 38.4, 23.2. HRMS calcd for C_31_H_37_NO_6_+H^+^ (M+H)^+^: 520.2699, found: 520.2704.

#### 3.11.4. Methyl 2,4,6-Tri-*O*-Benzyl-3-Deoxy-3-*C*-(2-Fluorobenzamidomethyl)-β-d-Gulopyranoside **22c**

Compound **22c** (TLC heptane/EtOAc, 2:1, R_f_ 0.19) was prepared according to the general procedure *3.11* from the amine **19** (49 mg, 0.10 mmol). Obtained as a colorless oil in 59% yield (31.5 mg, 0.06 mmol). ${\left[\alpha \right]}_{D}^{25}$ −41.7 (c 0.7, CHCl_3_). ^1^H NMR (CDCl_3_, 400 MHz): 8.05–8.01 (td, 1H, *J* 8.0 Hz, *J* 1.6 Hz, Ar*H*), 7.48–7.43 (m, 1H, Ar*H*), 7.36–7.16 (m, 17H, Ar*H*), 7.07–7.02 (m, 1H, Ar*H*), 4.84 (d, 1H, *J* 11.6 Hz, C*H*_2_Ph), 4.65 (d, 1H, *J*_1,2_ 6.8 Hz, H-1), 4.62 (d, 1H, *J* 12.0 Hz, C*H*_2_Ph), 4.57 (d, 1H, *J* 12.0 Hz, C*H*_2_Ph), 4.48 (d, 1H, *J* 12.0 Hz, C*H*_2_Ph), 4.46 (d, 1H, *J* 11.6 Hz, C*H*_2_Ph), 4.40 (d, 1H, *J* 11.6 Hz, C*H*_2_Ph), 4.07 (td, 1H, *J* 6.0 Hz, *J* 2.8 Hz, H-5), 3.79–3.58 (m, 6H, H-2, H-4, H-6a, H-6b, C*H*_2_NHCO), 3.54 (s, 3H, OC*H*_3_), 2.49–2.43 (m, 1H, H-3). ^13^C NMR (CDCl_3_, 100 MHz): 163.2 (d, *J* 3.0 Hz), 160.6 (d, *J* 247 Hz), 138.3, 138.2, 137.8, 133.1 (d, *J* 9.0 Hz), 132.0 (d, *J* 2.0 Hz), 128.46, 128.45, 128.4, 128.3, 128.2, 127.9, 127.8, 127.7, 124.7 (d, *J* 3.1 Hz), 121.4 (d, *J* 12 Hz), 116.1 (d, *J* 24 Hz), 101.0, 76.3, 75.1, 73.6, 73.5, 72.9, 72.0, 69.5, 56.5, 40.0, 38.5. ^19^F NMR (CDCl_3_, 376 MHz): -113.4. HRMS calcd for C_36_H_38_FNO_6_+NH_4_^+^ (M+NH_4_)^+^: 617.3027, found: 617.3025.

#### 3.11.5. Methyl 2,4,6-Tri-*O*-Benzyl-3-Deoxy-3-*C*-(3-Fluorobenzamidomethyl)-β-d-Gulopyranoside **22d**

Compound **22d** (TLC heptane/EtOAc, 2:1, R_f_ 0.24) was prepared according to the general procedure *3.11* from the amine **19** (46 mg, 0.10 mmol). Obtained as a colorless oil in 67% yield (48 mg, 0.06 mmol). ${\left[\alpha \right]}_{D}^{25}$ −61.8 (c 0.7, CHCl_3_). ^1^H NMR (CDCl_3_, 400 MHz): 7.36–6.99 (m, 20H, N*H*CO, Ar*H*), 4.89 (d, 1H, *J* 11.2 Hz, C*H*_2_Ph), 4.69 (d, 1H, *J*_1,2_ 6.0 Hz, H-1), 4.58 (d, 1H, *J* 12.0 Hz, C*H*_2_Ph), 4.54–4.47 (m, 4H, C*H*_2_Ph), 4.07 (td, 1H, *J* 6.4 Hz, *J* 2.8 Hz, H-5), 3.82 (dd, 1H, *J*_1,2_ 6.0 Hz, *J*_2,3_ 4.8 Hz, H-2), 3.81–3.66 (m, 4H, H-4, H-6a, H-6b, C*H*_2_NHCO), 3.60–3.54 (m, 4H, C*H*_2_NHCO, OC*H*_3_), 2.50–2.44 (m, 1H, H-3). ^13^C NMR (CDCl_3_, 100 MHz): 165.5 (d, *J* 2.2 Hz), 162.7 (d, *J* 246 Hz), 138.2, 138.0, 137.7, 136.6 (d, *J* 6.8 Hz), 130.0 (d, *J* 7.8 Hz), 128.7, 128.53, 128.50, 128.4, 128.29, 128.25, 128.0, 127.9, 127.8, 122.1 (d, *J* 3.0 Hz), 118.2 (d, *J* 22 Hz), 114.4 (d, *J* 23 Hz), 100.8, 78.8, 75.8, 74.3, 73.5, 73.0, 72.2, 69.3, 56.5, 39.6, 39.5. ^19^F NMR (CDCl_3_, 376 MHz): −111.9. HRMS calcd for C_36_H_38_FNO_6_+H^+^ (M+H)^+^: 600.2761, found: 600.2772.

#### 3.11.6. Methyl 2,4,6-Tri-*O*-Benzyl-3-Deoxy-3-*C*-(4-Fluorobenzamidomethyl)-β-d-Gulopyranoside **22e**

Compound **22e** (TLC heptane/EtOAc, 2:1, R_f_ 0.2) was prepared according to the general procedure *3.11* from the amine **19** (51 mg, 0.11 mmol). Obtained as a colorless oil in 71% yield (45.4 mg, 0.08 mmol). ${\left[\alpha \right]}_{D}^{25}$ +51.9 (c 0.6, CHCl_3_). ^1^H NMR (CDCl_3_, 400 MHz): 7.36–7.23 (m, 17H, Ar*H*), 7.05 (t, 1H, *J* 5.6 Hz, N*H*CO), 6.89–6.84 (m, 2H, Ar*H*), 4.90 (d, 1H, *J* 11.2 Hz, C*H*_2_Ph), 4.70 (d, 1H, *J*_1,2_ 6.0 Hz, H-1), 4.58 (d, 1H, *J* 12.0 Hz, C*H*_2_Ph), 4.54–4.48 (m, 4H, C*H*_2_Ph), 4.05 (td, 1H, *J* 6.0 Hz, *J* 2.8 Hz, H-5), 3.83 (dd, 1H, *J*_1,2_ 6.0 Hz, *J*_2,3_ 4.8 Hz, H-2), 3.80–3.67 (m, 4H, H-4, H-6a, H-6b, C*H*_2_NHCO), 3.59–3.52 (m, 4H, C*H*_2_NHCO, OC*H*_3_), 2.52–2.46 (m, 1H, H-3). ^13^C NMR (CDCl_3_, 100 MHz): 165.7, 164.5 (d, *J* 250 Hz), 138.2, 138.1, 130.3 (d, *J* 3.0 Hz), 129.1 (d, *J* 8.9 Hz), 128.8, 128.6, 128.53, 128.52, 128.33, 128.28, 128.0, 127.9, 127.8, 115.4 (d, *J* 22 Hz), 100.8, 78.1, 76.0, 74.4, 73.5, 73.1, 72.2, 69.3, 56.5, 39.61, 39.58. ^19^F NMR (CDCl_3_, 376 MHz): −108.8. HRMS calcd for C_36_H_38_FNO_6_+NH_4_^+^ (M+NH_4_)^+^: 617.3027, found: 617.3038.

#### 3.11.7. Methyl2,4,6-Tri-*O*-Benzyl-3-Deoxy-3-*C*-(3,4,5-Trifluorobenzamidomethyl)-β-d-Gulopyranoside **22f**

Compound **22f** (TLC heptane/EtOAc, 2:1, R_f_ 0.18) was prepared according to the general procedure *3.11* from the amine **19** (49 mg, 0.10 mmol). Obtained as a colorless oil in 53% yield (34.6 mg, 0.06 mmol). ${\left[\alpha \right]}_{D}^{25}$ −73.6 (c 0.8, CHCl_3_). ^1^H NMR (CDCl_3_, 400 MHz): 7.36–6.93 (m, 18H, Ar*H*), 4.87 (d, 1H, *J* 11.2 Hz, C*H*_2_Ph), 4.68 (d, 1H, *J*_1,2_ 5.6 Hz, H-1), 4.58 (d, 1H, *J* 11.6 Hz, C*H*_2_Ph), 4.51–4.48 (m, 4H, C*H*_2_Ph), 4.07 (td, 1H, *J* 6.4 Hz, *J* 3.6 Hz, H-5), 3.82–3.53 (m, 9H, H-2, H-4, H-6a, H-6b, OC*H*_3_, C*H*_2_NHCO), 2.46–2.40 (m, 1H, H-3). ^13^C NMR (CDCl_3_, 100 MHz): 163.7, 151.0 (ddd, *J* 3.4 Hz, *J* 10.2 Hz, *J* 251 Hz), 141.9 (dt, *J* 15.2 Hz, *J* 255 Hz), 138.2, 137.9, 137.7, 130.4–130.2 (m), 128.8, 128.6, 128.53, 128.47, 128.29, 128.27, 128.2, 127.90, 127.85, 111.5 (dd, *J* 6.1 Hz, *J* 16 Hz), 100.5, 78.1, 75.8, 74.3, 73.6, 73.0, 72.3, 69.3, 56.5, 40.0, 39.3. ^19^F NMR (CDCl_3_, 376 MHz): -132.1 (d, *J* 20 Hz), −155.7 (t, *J* 20 Hz). HRMS calcd for C_36_H_36_F_3_NO_6_+NH_4_^+^ (M+NH_4_)^+^: 653.2838, found: 653.2845.

#### 3.11.8. Methyl 2,4,6-Tri-*O*-Benzyl-3-Deoxy-3-*C*-(2,3,4,5,6-Pentafluorobenzamidomethyl)-β-d-Gulopyranoside **22g**

Compound **22g** (TLC heptane/EtOAc, 2:1, R_f_ 0.17) was prepared according to the general procedure *3.11* from the amine **19** (45 mg, 0.09 mmol). Obtained as a colorless oil in 49% yield (31 mg, 0.05 mmol). ${\left[\alpha \right]}_{D}^{25}$ −75.7 (c 0.5, CHCl_3_). ^1^H NMR (CDCl_3_, 400 MHz): 7.36–7.20 (m, 15H, Ar*H*), 6.81 (t, 1H, *J* 5.6 Hz, CH_2_N*H*CO), 4.89 (d, 1H, *J* 11.2 Hz, C*H*_2_Ph), 4.65 (d, 1H, *J*_1,2_ 6.0 Hz, H-1), 4.57 (d, 1H, *J* 12.0 Hz, C*H*_2_Ph), 4.51 (d, 1H, *J* 11.2 Hz, C*H*_2_Ph), 4.48 (d, 1H, *J* 12.0 Hz, C*H*_2_Ph), 4.47 (d, 1H, *J* 12.0 Hz, C*H*_2_Ph), 4.42 (d, 1H, *J* 12.0 Hz, C*H*_2_Ph), 4.06 (td, 1H, *J* 6.8 Hz, *J* 3.6 Hz, H-5), 3.81 (dd, 1H, *J*_1,2_ 6.0 Hz, *J*_2,3_ 3.6 Hz, H-2), 3.78–3.69 (m, 3H, H-4, H-6a, H-6b), 3.63–3.58 (m, 2H, C*H*_2_NHCO), 3.54 (s, 1H, OCH_3_), 2.45–3.39 (m, 1H, H-3). ^13^C NMR (CDCl_3_, 100 MHz): 156.9, 145.1–144.9 (m), 142.6–142.4 (m), 140.9–140.6 (m), 138.8–138.5 (m), 138.2, 137.9, 137.7, 136.3–136.0 (m), 128.5, 128.2, 128.1, 128.0, 127.9, 127.8, 111.9–111.5 (m), 106.4, 100.3, 77.8, 75.4, 73.9, 73.5, 72.4, 69.3, 56.5, 39.8, 38.9. ^19^F NMR (CDCl_3_, 376 MHz): −140.5 to −140.6 (m, 2F), −151.7 (t, 1F, *J* 21 Hz), −160.1 to −160.3 (m, 2F). HRMS calcd for C_36_H_34_F_5_NO_6_+NH_4_^+^ (M+NH_4_)^+^: 689.2650, found: 689.2656.

#### 3.11.9. Methyl 2,4,6-Tri-*O*-Benzyl-3-Deoxy-3-*C*-(3-Methoxybenzamidomethyl)-β-d-Gulopyranoside **22h**

Compound **22h** (TLC heptane/EtOAc, 1:1, R_f_ 0.45) was prepared according to the general procedure *3.11* from the amine **19** (47 mg, 0.10 mmol). Obtained as a colorless oil in 51% yield (30.7 mg, 0.05 mmol). ${\left[\alpha \right]}_{D}^{25}$ −43.2 (c 0.5, CHCl_3_). ^1^H NMR (CDCl_3_, 400 MHz): 7.35–7.21 (m, 17H, Ar*H*), 7.08 (t, 1H, *J* 8.0 Hz, Ar*H*), 7.00 (m, 2H, CH_2_N*H*CO, Ar*H*), 6.75–6.72 (m, 1H, Ar*H*), 4.87 (d, 1H, *J* 11.6 Hz, C*H*_2_Ph), 4.68 (d, 1H, *J*_1,2_ 6.4 Hz, H-1), 4.57 (d, 1H, *J* 11.6 Hz, C*H*_2_Ph), 4.54 (d, 1H, *J* 11.6 Hz, C*H*_2_Ph), 4.51 (d, 1H, *J* 12.8 Hz, C*H*_2_Ph), 4.48 (d, 1H, *J* 11.2 Hz, C*H*_2_Ph), 4.45 (d, 1H, *J* 11.2 Hz, C*H*_2_Ph), 4.05 (td, 1H, *J* 6.4 Hz, *J* 2.8 Hz, H-5), 3.83 (dd, 1H, *J*_1,2_ 6.4 Hz, *J*_2,3_ 5.2 Hz, H-2), 3.79 (s, 3H, C_6_H_4_OC*H*_3_), 3.78–3.67 (m, 5H, H-2, H-4, H-6a, H-6b, C*H*_2_NHCO), 3.59–3.53 (m, 4H, C*H*_2_NHCO, OCH_3_), 2.50–2.45 (m, 1H, H-3). ^13^C NMR (CDCl_3_, 100 MHz): 166.8, 159.9, 138.3, 138.2, 137.8, 129.5, 128.7, 128.5, 128.4, 128.3, 128.1, 128.0, 127.9, 127.8, 118.4, 117.7, 112.3, 100.9, 77.6, 75.8, 74.2, 73.5, 73.1, 72.1, 69.4, 56.5, 55.5, 39.8, 39.2. HRMS calcd for C_37_H_41_NO_7_+H^+^ (M+H)^+^: 612.2961, found: 612.2972.

#### 3.11.10. Methyl 2,4,6-Tri-*O*-Benzyl-3-Deoxy-3-*C*-(p-Toluamidomethyl)-β-d-Gulopyranoside **22i**

Compound **22i** (TLC heptane/EtOAc, 2:1, R_f_ 0.24) was prepared according to the general procedure *3.11* from the amine **19** (51 mg, 0.11 mmol). Obtained as a colorless oil in 61% yield (38.8 mg, 0.07 mmol). ${\left[\alpha \right]}_{D}^{25}$ −56.2 (c 0.5, CHCl_3_). ^1^H NMR (CDCl_3_, 400 MHz): 7.35–7.21 (m, 17H, Ar*H*), 7.04 (d, 1H, *J* 7.6 Hz, Ar*H*), 6.96 (t, 1H, *J* 6.0 Hz, CH_2_N*H*CO), 4.88 (d, 1H, *J* 11.2 Hz, C*H*_2_Ph), 4.68 (d, 1H, *J*_1,2_ 6.4 Hz, H-1), 4.57 (d, 1H, *J* 12.0 Hz, C*H*_2_Ph), 4.53 (d, 1H, *J* 11.2 Hz, C*H*_2_Ph), 4.51 (d, 1H, *J* 12.0 Hz, C*H*_2_Ph), 4.48 (d, 1H, *J* 12.0 Hz, C*H*_2_Ph), 4.45 (d, 1H, *J* 11.6 Hz, C*H*_2_Ph), 4.04 (td, 1H, *J* 6.4 Hz, *J* 2.8 Hz, H-5), 3.82 (dd, 1H, *J*_1,2_ 6.4 Hz, *J*_2,3_ 5.2 Hz, H-2), 3.78–3.67 (m, 3H, H-4, H-6a, H-6b, C*H*_2_NHCO), 3.58–3.52 (m, 4H, C*H*_2_NHCO, OC*H*_3_), 2.51–2.45 (m, 1H, H-3), 2.36 (s, 3H, C*H*_3_). ^13^C NMR (CDCl_3_, 100 MHz): 166.8, 141.6, 138.3, 138.2, 137.9, 131.4, 130.3, 129.24, 129.16, 128.7, 128.52, 128.51, 128.4, 128.3, 128.1, 128.0, 127.9, 127.8, 126.9, 101.0, 77.8, 75.9, 74.2, 73.5, 73.1, 72.1, 69.4, 56.5, 39.9, 39.2, 21.5. HRMS calcd for C_37_H_41_NO_6_+H^+^ (M+H)^+^: 596.3012, found: 596.3019.

#### 3.11.11. Methyl 2,4,6-Tri-*O*-Benzyl-3-Deoxy-3-*C*-(3,5-Dimethoxybenzamidomethyl)-β-d-Gulopyranoside **22j**

Compound **22j** (TLC heptane/EtOAc, 2:1, R_f_ 0.22) was prepared according to the general procedure *3.11* from the amine **19** (53 mg, 0.11 mmol). Obtained as a colorless oil in 67% yield (48 mg, 0.07 mmol). ${\left[\alpha \right]}_{D}^{25}$ −39.5 (c 0.8, CHCl_3_). ^1^H NMR (CDCl_3_, 400 MHz): 7.35–7.18 (m, 15H, Ar*H*), 6.82 (t, 1H, *J* 6.0 Hz, N*H*CO), 6.72 (d, 2H, *J* 2.4 Hz, Ar*H*), 6.54 (t, 1H, *J* 2.4 Hz, Ar*H*), 4.84 (d, 1H, *J* 11.6 Hz, C*H*_2_Ph), 4.65 (d, 1H, *J*_1,2_ 6.4 Hz, H-1), 4.57 (d, 1H, *J* 12.0 Hz, C*H*_2_Ph), 4.56 (d, 1H, *J* 12.0 Hz, C*H*_2_Ph), 4.48 (d, 1H, *J* 11.6 Hz, C*H*_2_Ph), 4.47 (d, 1H, *J* 11.6 Hz, C*H*_2_Ph), 4.43 (d, 1H, *J* 11.6 Hz, C*H*_2_Ph), 4.03 (td, 1H, *J* 6.4 Hz, *J* 2.8 Hz, H-5), 3.78 (dd, 1H, *J*_1,2_ 6.0 Hz, *J*_2,3_ 4.8 Hz, H-2), 3.75–3.65 (m, 10H, H-4, H-6a, H-6b, C*H*_2_NHCO, 2 × OCH_3_), 3.57–3.52 (m, 4H, C*H*_2_NHCO, OC*H*_3_), 2.47–2.41 (m, 1H, H-3). ^13^C NMR (CDCl_3_, 100 MHz): 167.1, 160.9, 163.3, 138.3, 138.2, 137.8, 136.8, 128.7, 128.5, 128.2, 128.1, 128.0, 127.93, 127.88, 127.8, 104.9, 103.6, 100.9, 77.2, 75.6, 73.9, 73.5, 73.1, 72.1, 69.5, 56.5, 55.6, 39.9, 39.0. HRMS calcd for C_38_H_43_NO_8_+H^+^ (M+H)^+^: 642.3066, found: 642.3067.

#### 3.11.12. Methyl 2,4,6-tri-*O*-Benzyl-3-Deoxy-3-*C*-(3-Trifluoromethylbenzamidomethyl)-β-d-Gulopyranoside **22k**

Compound **22k** (TLC heptane/EtOAc, 2:1, R_f_ 0.25) was prepared according to the general procedure *3.11* from the amine **19** (43 mg, 0.09 mmol). Obtained as a colorless oil in 55% yield (32.2 mg, 0.05 mmol). ${\left[\alpha \right]}_{D}^{25}$ −38.7 (c 0.8, CHCl_3_). ^1^H NMR (CDCl_3_, 400 MHz): 8.00 (s, 1H, Ar*H*), 7.69–7.66 (m, 1H, Ar*H*), 7.34–7.21 (m, 17H, Ar*H*), 7.09 (t, 1H, *J* 6.0 Hz, CH_2_N*H*CO), 4.88 (d, 1H, *J* 11.6 Hz, C*H*_2_Ph), 4.69 (d, 1H, *J*_1,2_ 6.0 Hz, H-1), 4.58 (d, 1H, *J* 12.0 Hz, C*H*_2_Ph), 4.53 (d, 1H, *J* 11.2 Hz, C*H*_2_Ph), 4.51–4.47 (m, 3H, C*H*_2_Ph), 4.07 (s td, 1H, *J* 6.4 Hz, *J* 3.2 Hz, H-5), 3.83 (dd, 1H, *J* 6.0 Hz, *J* 4.8 Hz, H-2), 3.81–3.68 (m, 4H, H-2, H-4, H-6a, H-6b, C*H*_2_NHCO), 3.62–3.59 (m, 1H, C*H*_2_NHCO), 3.56 (s, 3H, OC*H*_3_), 2.50–2.45 (m, 1H, H-3). ^13^C NMR (CDCl_3_, 100 MHz): 165.5, 138.2, 138.1, 137.8, 135.1, 133.4 131.2 (q, *J* 32 Hz), 129.5, 129.2, 129.1, 128.7, 128.54, 128.52, 128.4, 128.31, 128.25, 128.0, 127.9, 127.8, 125.2, 124.6 (q, *J* 3.7 Hz), 123.7 (q, *J* 271 Hz), 100.8, 77.8, 75.8, 74.3, 73.6, 73.1, 72.2, 69.3, 56.5, 39.62, 39.61. ^19^F NMR (CDCl_3_, 376 MHz): −62.7. HRMS calcd for C_37_H_38_F_3_NO_6_+H^+^ (M+H)^+^: 650.2729, found: 650.2727.

#### 3.11.13. Methyl 2,4,6-Tri-*O*-Benzyl-3-Deoxy-3-*C*-(4-Phenylbenzamidomethyl)-β-d-Gulopyranoside **22l**

Compound **22l** (TLC heptane/EtOAc, 2:1, R_f_ 0.32) was prepared according to the general procedure *3.11* from the amine **19** (60 mg, 0.13 mmol). Obtained as a colorless oil in 55% yield (45.5 mg, 0.07 mmol). ${\left[\alpha \right]}_{D}^{25}$ −47.8 (c 0.9, CHCl_3_). ^1^H NMR (CDCl_3_, 400 MHz): 7.61–7.24 (m, 24H, Ar*H*), 7.10 (t, 1H, *J* 6.0 Hz, CH_2_N*H*CO), 4.92 (d, 1H, *J* 11.2 Hz, C*H*_2_Ph), 4.72 (d, 1H, *J*_1,2_ 6.4 Hz, H-1), 4.59 (d, 1H, *J* 12.0 Hz, C*H*_2_Ph), 4.57–4.48 (m, 4H, C*H*_2_Ph), 4.08 (td, 1H, *J* 6.0 Hz, *J* 2.8 Hz, H-5), 3.85 (dd, 1H, *J*_1,2_ 6.0 Hz, *J*_2,3_ 4.8 Hz, H-2), 3.82–3.70 (m, 4H, H-4, H-6a, H-6b, C*H*_2_NHCO), 3.64–3.59 (m, 1H, C*H*_2_NHCO), 3.58 (s, 3H, OC*H*_3_), 2.55–2.50 (m, 1H, H-3). ^13^C NMR (CDCl_3_, 100 MHz): 166.5, 143.9, 140.3, 138.3, 138.2, 137.9, 132.9, 129.0, 128.7, 128.53, 128,49, 128.3, 128.2, 128.02, 127.98, 127.9, 127.8, 127.4, 127.24, 127.15, 100.9, 77.9, 75.9, 74.3, 73.5, 73.1, 72.2, 69.4, 56.5, 39.8, 39.4. HRMS calcd for C_42_H_43_NO_6_+NH_4_^+^ (M+NH_4_)^+^: 675.3434, found: 675.3433.

#### 3.11.14. Methyl 2,4,6-tri-*O*-Benzyl-3-Deoxy-3-*C*-(Diphenylphosphonamidomethyl)-β-d-Gulopyranoside **23**

Compound **23** (TLC heptane/EtOAc, 2:1, R_f_ 0.26) was prepared according to the general procedure *3.11* from the amine **19** (52 mg, 0.11 mmol). Obtained as a colorless oil in 69% yield (53.3 mg, 0.08 mmol). ${\left[\alpha \right]}_{D}^{25}$ −77.8 (c 0.7, CHCl_3_). ^1^H NMR (CDCl_3_, 400 MHz): 7.36–7.13 (m, 20H, Ar*H*), 4.76 (d, 1H, *J* 12.0 Hz, CH_2_Ph), 4.55–49 (m, 3H, H-1, C*H*_2_Ph), 4.45 (d, 1H, *J* 12.0 Hz, C*H*_2_Ph), 4.34 (d, 1H, *J* 11.6 Hz, C*H*_2_Ph), 4.28 (d, 1H, *J* 11.6 Hz, C*H*_2_Ph), 3.95–92 (m, 1H, H-5), 3.71–3.58 (m, 5H, H-2, H-4, H-6a, H-6b, N*H*PO(OPh)_2_), 3.47 (s, 3H, OC*H*_3_), 3.40–3.31 (m, 1H, C*H*_2_NHSO), 3.15–3.09 (m, 1H, C*H*_2_NHSO), 2.33–2.27 (m, 1H, H-3). ^13^C NMR (CDCl_3_, 100 MHz): 150.9 (dd, *J* 6.7 Hz, *J* 2.4 Hz), 138.22, 138.21, 137.9, 128.6, 128.51, 128.45, 128.1, 128.0, 127.93, 127.87, 127.8, 125.0 (d, *J* 4.3 Hz), 120.3 (d, *J* 5.0 Hz, *J* 8.0 Hz), 100.8, 76.1, 74.9, 73.6, 73.4, 72.8, 71.8, 69.5, 56.5, 42.1 (d, *J* 1.8 Hz), 40.2. ^31^P NMR (CDCl_3_, 162 MHz): -1.01. HRMS calcd for C_41_H_44_PNO_8_+H^+^ (M+H)^+^: 710.2883, found: 710.2889.

### 3.12. General Procedure the Synthesis of ***1a***, ***1b***, ***2–6***, ***7a***–***7l***, and ***8***

A solution of crude in EtOAc/isopropanol (1:3) was stirred with Pd(OH)_2_/C (10% wt., 1 mg per 4 mg of crude) under hydrogen atmosphere at room temperature for 12 h. All the hydrogenation reactions were carried out in an EtOAc-isopropanol mixture (1:3, 4 mL). After the completion of the reaction (as indicated by TLC), the reaction mixture was filtered through a Celite bed and washed with methanol. The filtrate was concentrated under reduced pressure and purified through the flash column (DCM:MeOH) to get the desired compounds as white amorphous solids or colorless oils.

#### 3.12.1. Methyl 3-Deoxy-3-*C*-Hydroxymethyl-β-d-Gulopyranoside **1a**

Compound **1a** (TLC, DCM/MeOH, 5:1, R_f_ 0.41) was prepared according to the general procedure *3.12* from the alcohol **14a** (63 mg, 0.13 mmol). Obtained as a white amorphous solid in 51% yield (14 mg, 0.07 mmol) from flash column chromatography (DCM:MeOH 12:1–5:1). ${\left[\alpha \right]}_{D}^{25}$ −50.7 (c 0.6, CH_3_OH). ^1^H NMR (CD_3_OD, 400 MHz): 4.39 (d, 1H, *J* 7.6 Hz, H-1), 3.96 (dd, 1H, *J*_3,4_ 4.0 Hz, *J*_4,5_ 2.0 Hz, H-4), 3.92 (dd, 1H, *J* 11.2 Hz, *J* 5.6 Hz, C*H*_2_OH), 3.86 (dd, 1H, *J*_1,2_ 7.6 Hz, *J*_2,3_ 6.0 Hz H-2), 3.84–3.74 (m, 3H, H-5, H-6a, H-6b), 3.67 (dd, 1H, *J* 11.2 Hz, *J* 8.4 Hz, C*H*_2_OH), 3.51 (s, 3H, OC*H*_3_), 2.32–2.26 (m, 1H, H-3). ^13^C NMR (CD_3_OD, 125 MHz): 104.0, 76.1, 69.0, 68.3, 63.1, 59.6, 56.8, 49.0. HRMS calcd for C_8_H_16_O_6_-H^+^ (M-H)^+^: 207.0869, found: 207.0865.

#### 3.12.2. Methyl 3-Deoxy-3-*C*-Hydroxymethyl-β-d-Galactopyranoside **1b**

Compound **1b** (TLC, DCM/MeOH, 5:1, R_f_ 0.40) was prepared according to the general procedure *3.12* from the alcohol **14b** (46 mg, 0.10 mmol). Obtained as a white amorphous solid in 63% yield (12.6 mg, 0.06 mmol) from flash column chromatography (DCM:MeOH 12:1–6:1). ${\left[\alpha \right]}_{D}^{25}$ −32.1 (c 0.5, CH_3_OH). ^1^H NMR (CD_3_OD, 500 MHz): 4.16 (d, 1H, *J* 7.6 Hz, H-1), 3.97 (d, 1H, *J*_3,4_ 2.4 Hz, H-4), 3.90 (dd, 1H, *J* 10.4 Hz, *J* 4.4 Hz, C*H*_2_OH), 3.78 (dd, 1H, *J* 10.8 Hz, *J* 8.4 Hz, H-2), 3.72 (dd, 1H, *J* 5.6 Hz, H-6a, H-6b), 3.55–3.51 (m, 4H, H-5, OC*H*_3_), 3.44 (dd, 1H, *J* 10.8 Hz, *J* 7.6 Hz, C*H*_2_OH), 1.73–1.66 (m, 1H, H-3). ^13^C NMR (CD_3_OD, 125 MHz): 107.6, 79.8, 68.8, 67.1, 62.7, 61.2, 57.1, 49.0. HRMS calcd for C_8_H_16_O_6_+Na^+^ (M+Na)^+^: 231.0845, found: 231.0840.

#### 3.12.3. Methyl 3-Deoxy-3-*C*-(3-Trifluoromethylphenoxymethyl)-β-d-Galactopyranoside **2**

Compound **2** (TLC, DCM/MeOH, 10:1, R_f_ 0.5) was prepared according to the general procedure *3.12* from the ether **15** (53 mg, 0.09 mmol). Obtained as a colorless oil in 75% yield (22.5 mg, 0.06 mmol) from flash column chromatography (DCM:MeOH 20:1–12:1). ${\left[\alpha \right]}_{D}^{25}$ −12.8 (c 0.7, CH_3_OH). ^1^H NMR (CD_3_OD, 400 MHz): 7.46 (t, 1H, *J* 8.0 Hz, Ar*H*), 7.23–7.21 (m, 3H, Ar*H*), 4.50 (d, 1H, *J* 7.2 Hz, H-1), 4.35 (dd, 1H, *J* 4.8 Hz, *J* 10.0 Hz, C*H*_2_OH), 4.22 (t, 1H, *J* 8.8 Hz, C*H*_2_OH), 4.08 (bs, 1H, H-4), 3.97–3.94 (m, 2H, H-2, H-5), 3.78 (d, 2H, *J* 6.0 Hz, H-6a, H-6b), 3.54 (s, 3H, OC*H*_3_), 2.64–2.58 (m, 1H, H-3). ^13^C NMR (CD_3_OD, 100 MHz): 160.6, 132.8 (q*, *J* 32 Hz), 131.4, 125.5 (q*, *J* 270 Hz), 119.3, 118.3 (br q, *J* 3.7 Hz), 112.4 (br q, *J* 3.8 Hz), 104.1, 76.1, 68.2, 68.1, 65.9, 62.9, 56.8, 46.1. ^19^F NMR (CD_3_OD, 376 MHz): −64.2. HRMS calcd for C_15_H_20_F_3_O_6_+H^+^ (M+H)^+^: 353.1212, found: 353.1208.

*Only two peaks from the q are observed: See Supplementary information page S43)

#### 3.12.4. Methyl 3-Deoxy-3-*C*-Methoxymethyl-β-d-Galactopyranoside **3**

Compound **3** (TLC, DCM/MeOH, 10:1, R_f_ 0.5) was prepared according to the general procedure *3.12* from **16** (36 mg, 0.07 mmol). Obtained as a colorless oil in 64% yield (10.4 mg, 0.05 mmol). ${\left[\alpha \right]}_{D}^{25}$ −33.4 (c 0.5, CH_3_OH) from flash column chromatography (DCM:MeOH 15:1–9:1). ^1^H NMR (CD_3_OD, 400 MHz): 4.41 (d, 1H, *J* 7.6 Hz, H-1), 3.93 (dd, 1H, *J*_4,5_ 3.2 Hz, *J*_3,4_ 2.0 Hz, H-4), 3.88–3.82 (m, 2H, H-2, H-5), 3.73 (d, 2H, *J* 6.0 Hz, H-6a, H-6b), 3.65 (dd, 1H, *J* 10.0 Hz, *J* 4.8 Hz, CH_2_OCH_3_), 3.57 (dd, 1H, *J* 10.0 Hz, *J* 4.8 Hz, CH_2_OCH_3_), 3.50 (s, 3H, OC*H*_3_), 3.33 (s, 3H, OC*H*_3_), 2.38–2.34 (m, 1H, H-3). ^13^C NMR (CD_3_OD, 100 MHz): 104.1, 76.3, 70.2, 68.7, 68.5, 63.1, 59.1, 56.8, 46.6. HRMS calcd for C_9_H_18_O_6_+Na^+^ (M+Na)^+^: 245.1001, found: 245.1004.

#### 3.12.5. Methyl 3-Deoxy-3-*C*-[4-(3-Fluorophenyl)-1H-1,2,3-Triazol-1-Ylmethyl]-β-d-Galactopyranoside **4**

Compound **4** (TLC, DCM/MeOH, 10:1, R_f_ 0.43) was prepared according to the general procedure *3.12* from triazole **18** (55 mg, 0.09 mmol). Obtained as a colorless oil in 78% yield (24.3 mg, 0.07 mmol) from flash column chromatography (DCM:MeOH 20:1–9:1). ${\left[\alpha \right]}_{D}^{25}$ −20.5 (c 0.7, CH_3_OH). ^1^H NMR (CD_3_OD, 400 MHz): 8.40 (s, 1H, Ar*H*), 7.65–7.05 (m, 4H, Ar*H*), 4.80 (dd, 1H, *J* 14.4 Hz, *J* 6.0 Hz, C*H*_2_N_3_C_8_H_5_F), 4.63 (dd, 1H, *J* 14.4 Hz, *J* 9.2 Hz, C*H*_2_OH), 4.48 (d, 1H, *J* 6.4 Hz, H-1), 4.02–3.99 (m, 1H, H-5), 3.82–3.78 (m, 4H, H-2, H-4, H-6a, H-6b), 3.53 (s, 3H, OC*H*_3_), 2.72–2.66 (m, 1H, H-3). ^13^C NMR (CD_3_OD, 100 MHz): 164.3 (d, *J* 243 Hz), 147.7 (d, *J* 3.1 Hz), 134.0 (d, *J* 8.3 Hz), 131.8 (d, *J* 8.4 Hz), 123.4, 122.4 (d, *J* 2.5 Hz), 115.9 (d, *J* 21 Hz), 113.2 (d, *J* 23 Hz), 103.6, 75.8, 68.0, 67.4, 62.8, 56.8, 48.2, 46.8. HRMS calcd for C_16_H_20_FN_3_O_5_+H^+^ (M+H)^+^: 354.1465, found: 354.1462.

#### 3.12.6. Methyl 3-Deoxy-3-*C*-(3-Fluorophenylureido)Methyl-β-d-Galactopyranoside **5**

Compound **5** (TLC, DCM/MeOH, 10:1, R_f_ 0.44) was prepared according to the general procedure *3.12* from **20** (50 mg, 0.0814 mmol). Obtained as a colorless oil in 41% yield (11.5 mg, 0.03 mmol) from flash column chromatography (DCM:MeOH 12:1–5:1). ${\left[\alpha \right]}_{D}^{25}$ −17.3 (c 0.6, CH_3_OH). ^1^H NMR (CD_3_OD, 400 MHz): 7.34 (dt, 1H, *J* 12.0 Hz, *J* 2.0 Hz, Ar*H*), 7.24–6.64 (m, 3H, Ar*H*), 4.44 (d, 1H, *J* 7.2 Hz, H-1), 3.90–3.83 (m, 3H, H-2, H-4, H-5), 4.22 (t, 1H, *J* 8.8 Hz, C*H*_2_OH), 4.08 (bs, 1H, H-4), 3.97–3.94 (m, 2H, H-2, H-5), 3.78–3.76 (d, 2H, H-6a, H-6b), 3.52 (s, 3H, OC*H*_3_), 3.47 (dd, 1H, *J* 14.4 Hz, *J* 6.4 Hz, C*H*_2_NHCONH), 3.41 (dd, 1H, *J* 14.4 Hz, *J* 7.6 Hz, C*H*_2_NHCONH), 2.27–2.21 (m, 1H, H-3). ^13^C NMR (CD_3_OD, 100 MHz): 164.5 (d, *J* 240 Hz), 158.0, 143.0 (d, *J* 11 Hz), 131.0 (d, *J* 9.8 Hz), 115.2 (d, *J* 3.2 Hz), 109.4 (d, *J* 22 Hz), 106.7 (d, *J* 22 Hz), 103.8, 76.1, 69.2, 69.0, 63.0, 56.9, 46.8, 38.0. HRMS calcd for C_15_H_21_FN_2_O_6_+H^+^ (M+H)^+^: 354.1462, found: 345.1459.

#### 3.12.7. Methyl 3-Deoxy-3-*C*-(Phenylsufonamido)Methyl-β-d-Galactopyranoside **6**

Compound **6** (TLC, DCM/MeOH, 10:1, R_f_ 0.45) was prepared according to the general procedure *3.12* from amide **21** (39 mg, 0.06 mmol). Obtained as a colorless oil in 53% yield (11.6 mg, 0.03 mmol) from flash column chromatography (DCM:MeOH 20:1–10:1). ${\left[\alpha \right]}_{D}^{25}$ −21.4 (c 0.6, CH_3_OH). ^1^H NMR (CD_3_OD, 400 MHz): 7.88–7.56 (m, 5H, Ar*H*), 4.26 (d, 1H, *J* 7.2 Hz, H-1), 3.93 (d, 1H, *J* 3.6 Hz, H-4), 3.80 (dd, 1H, *J*_1,2_ 7.2 Hz, *J*_2,3_ 6.0 Hz, H-2), 3.76–3.70 (m, 3H, H-5, H-6a, H-6b), 3.20 (dd, 1H, *J* 5.2 Hz, *J* 11.2 Hz, C*H*_2_NH), 3.20 (dd, 1H, *J* 11.2 Hz, *J* 10.0 Hz, C*H*_2_NH), 2.26–2.21 (m, 1H, H-3). ^13^C NMR (CD_3_OD, 100 MHz): 141.7, 133.7, 130.3, 128.0, 103.5, 75.7, 68.4, 67.9, 63.2, 56.8, 46.3, 40.6. HRMS calcd for C_14_H_21_NO_7_S+H^+^ (M+H)^+^: 348.1117, found: 348.1115.

#### 3.12.8. Methyl 3-Deoxy-3-*C*-Benzamidomethyl-β-d-Gulopyranoside **7a**

Compound **7a** (TLC, DCM/MeOH, 10:1, R_f_ 0.41) was prepared according to the general procedure *3.12* from the amide **22a** (35 mg, 0.05 mmol). Obtained as a colorless oil in 59% yield (11 mg, 0.04 mmol) from flash column chromatography (DCM:MeOH 20:1–10:1). ${\left[\alpha \right]}_{D}^{25}$ −6.5 (c 0.6, CH_3_OH). ^1^H NMR (CD_3_OD, 400 MHz): 7.82–7.80 (m, 2H, Ar*H*), 7.56–7.52 (m, 1H, Ar*H*), 7.49–7.44 (m, 2H, Ar*H*), 4.48 (d, 1H, *J*_1,2_ 6.8 Hz, H-1), 3.95 (td, 1H, *J* 6.0 Hz, *J* 2.4 Hz, H-5), 3.89–3.86 (m, 2H, H-2, H-4), 3.79 (d, 1H, *J*_6a,6b_ 12.4 Hz, *J*_5,6a_ 5.6 Hz, H-6a), 3.75 (dd, 1H, *J*_6a,6b_ 12.4 Hz, *J*_5,6b_ 5.6 Hz, H-6b), 3.68 (dd, 1H, *J* 14.0 Hz, *J* 6.4 Hz, C*H*_2_NH), 3.61 (dd, 1H, *J* 11.2 Hz, *J* 6.4 Hz, C*H*_2_NH), 3.54 (s, 3H, OC*H*_3_), 2.42–2.35 (m, 1H, H-3). ^13^C NMR (CD_3_OD, 100 MHz): 170.5, 135.6, 132.7, 129.6, 128.2, 103.7, 76.0, 69.0, 68.7, 63.1, 56.8, 46.4, 37.8. HRMS calcd for C_14_H_19_NO_6_+H^+^ (M+H)^+^: 289.1291, found: 298.1289.

#### 3.12.9. Methyl 3-Deoxy-3-*C*-Acetamidomethyl-β-d-Gulopyranoside **7b**

Compound **7b** (TLC, DCM/MeOH, 10:1, R_f_ 0.42) was prepared according to the general procedure *3.12* from the amide **22b** (27 mg, 0.05 mmol). Obtained as a colorless oil in 75% yield (9.7 mg, 0.04 mmol) from flash column chromatography (DCM:MeOH 15:1–7:1). ${\left[\alpha \right]}_{D}^{25}$ −1.5 (c 0.6, CH_3_OH). ^1^H NMR (CD_3_OD, 400 MHz): 4.40 (d, 1H, *J*_1,2_ 6.8 Hz, H-1), 3.88–3.84 (m, 1H, H-5), 3.82–371 (m, 4H, H-2, H-4, H-6a, H-6b), 3.51 (s, 3H, OC*H*_3_), 3.44 (dd, 1H, *J* 14.0 Hz, *J* 6.0 Hz, C*H*_2_NH), 3.38 (dd, 1H, *J* 14.0 Hz, *J* 8.8 Hz, C*H*_2_NH), 2.24–2.18 (m, 1H, H-3), 1.95 (s, 3H, NHCOC*H*_3_). ^13^C NMR (CD_3_OD, 100 MHz): 173.6, 103.6, 75.9, 68.7, 68.5, 63.1, 56.8, 46.3, 37.1, 22.6. HRMS calcd for C_10_H_19_NO_6_+H^+^ (M+H)^+^: 250.1291, found: 250.1291.

#### 3.12.10. Methyl 3-Deoxy-3-*C*-(2-Fluorobenzamidomethyl)-β-d-Galactopyranoside **7c**

Compound **7c** (TLC, DCM/MeOH, 10:1, R_f_ 0.4) was prepared according to the general procedure 3.12 from the amide **22c** (33 mg, 0.06 mmol). Obtained as a colorless oil in 69% yield (12.5 mg, 0.04 mmol) from flash column chromatography (DCM:MeOH 20:1–9:1). ${\left[\alpha \right]}_{D}^{25}$ −9.3 (c 0.5, CH_3_OH). ^1^H NMR (CD_3_OD, 400 MHz): 7.76 (td, 1H, *J* 7.6 Hz, *J* 2.0 Hz, Ar*H*), 7.56–7.50 (m, 1H, Ar*H*), 7.28 (td, 1H, *J* 7.6 Hz, *J* 0.8 Hz, Ar*H*), 7.20 (ddd, 1H, *J* 11.2 Hz, *J* 8.4 Hz, *J* 0.8 Hz, Ar*H*), 4.48 (d, 1H, *J*_1,2_ 7.2 Hz, H-1), 3.93–3.86 (m, 3H, H-2, H-4, H-5), 3.77 (d, 2H, *J*_5,6a_, *J*_5,6b_ 5.6 Hz, H-6a, H-6b), 3.66 (d, 2H, *J* 7.2 C*H_2_*NH), 3.53 (s, 3H, OC*H*_3_), 2.42–2.34 (m, 1H, H-3). ^13^C NMR (CD_3_OD, 100 MHz): 166.7, 161.4 (d, *J* 248 Hz), 134.2 (d, *J* 8.8 Hz), 131.6 (d, *J* 2.3 Hz), 125.7 (d, *J* 3.4 Hz), 123.9 (d, *J* 14 Hz), 131.6 (d, *J* 23 Hz), 103.7, 76.0, 69.0, 68.8, 63.1, 56.9, 46.1, 38.1. ^19^F NMR (CD_3_OD, 376 MHz): −116.0. HRMS calcd for C_15_H_20_FNO_6_+H^+^ (M+H)^+^: 330.1353, found: 330.1352.

#### 3.12.11. Methyl 3-Deoxy-3-*C*-(3-Fluorobenzamidomethyl)-β-d-Galactopyranoside **7d**

Compound **7d** (TLC, DCM/MeOH, 10:1, R_f_ 0.45) was prepared according to the general procedure *3.12* from the amide **22d** (35 mg, 0.06 mmol). Obtained as a colorless oil in 59% yield (11.3 mg, 0.03 mmol) from flash column chromatography (DCM:MeOH 20:1–10:1). ${\left[\alpha \right]}_{D}^{25}$ −14.6 (c 0.6, CH_3_OH). ^1^H NMR (CD_3_OD, 400 MHz): 7.65–7.26 (m, 4H, Ar*H*), 4.47 (d, 1H, *J* 7.2 Hz, H-1), 3.94 (td, 1H, *J* 6.0 Hz, *J* 2.0 Hz, H-5), 3.88–3.85 (m, 2H, H-2, H-4), 3.77 (d, 2H, *J* 5.6 Hz, H-6a, H-6b), 3.67 (dd, 1H, *J* 14.0 Hz, *J* 6.4 Hz, C*H*_2_OH), 3.67 (dd, 1H, *J* 14.0 Hz, *J* 9.2 Hz, C*H*_2_OH), 3.53 (s, 3H, OC*H*_3_), 2.41–2.35 (m, 1H, H-3). ^13^C NMR (CD_3_OD, 100 MHz): 168.9 (d, *J* 2.7 Hz), 164.1 (d, *J* 244 Hz), 138.0 (d, *J* 6.8 Hz), 131.6 (d, *J* 7.9 Hz), 124.0 (d, *J* 2.9 Hz), 119.4 (d, *J* 22 Hz), 115.2 (d, *J* 23 Hz), 103.6, 75.9, 68.9, 68.6, 63.1, 56.8, 46.4, 37.8. HRMS calcd for C_15_H_20_FNO_6_+H^+^ (M+H)^+^: 330.1353, found: 330.1354.

#### 3.12.12. Methyl 3-Deoxy-3-*C*-(4-Fluorobenzamidomethyl)-β-d-Galactopyranoside **7e**

Compound **7e** (TLC, DCM/MeOH, 10:1, R_f_ 0.44) was prepared according to the general procedure *3.12* from the amide **22e** (40 mg, 0.07 mmol). Obtained as a colorless oil in 53% yield (11.6 mg, 0.04 mmol) from flash column chromatography (DCM:MeOH 20:1–9:1). ${\left[\alpha \right]}_{D}^{25}$ −16.4 (c 0.5, CH_3_OH). ^1^H NMR (CD_3_OD, 400 MHz): 7.89–7.84 (m, 2H, Ar*H*), 7.22–7.16 (m, 2H, Ar*H*), 4.47 (d, 1H, *J*_1,2_ 7.2 Hz, H-1), 3.94 (td, 1H, *J* 6.0 Hz, *J* 3.0 Hz, H-5), 3.88–3.85 (m, 2H, H-2, H-4), 3.78 (d, 1H, *J*_6a,6b_ 11.6 Hz, *J*_5,6a_ 5.6 Hz, H-6a), 3.75 (d, 1H, *J*_6a,6b_ 11.6 Hz, *J*_5,6b_ 5.6 Hz, H-6b), 3.66 (d, 1H, *J* 14.0 Hz, *J* 6.4 Hz, C*H*_2_NH), 3.61 (d, 1H, *J* 14.0 Hz, *J* 6.4 Hz, C*H*_2_NH), 3.53 (s, 3H, OC*H*_3_), 2.40–2.34 (m, 1H, H-3). ^13^C NMR (CD_3_OD, 100 MHz): 169.3, 166.2 (d, *J* 249 Hz), 131.9 (d, *J* 3.0 Hz), 130.8 (d, *J* 8.9 Hz), 116.4 (d, *J* 22 Hz), 103.7, 75.9, 69.0, 68.6, 63.1, 56.8, 46.4, 37.8. ^19^F NMR (CD_3_OD, 376 MHz): −110.7. HRMS calcd for C_15_H_20_FNO_6_+H^+^ (M+H)^+^: 330.1353, found: 330.1354.

#### 3.12.13. Methyl 3-Deoxy-3-*C*-(3,4,5-Trifluorobenzamidomethyl)-β-d-Galactopyranoside **7f**

Compound **7f** (TLC, DCM/MeOH, 10:1, R_f_ 0.47) was prepared according to the general procedure *3.12* from the amide **22f** (31 mg, 0.05 mmol). Obtained as a colorless oil in 70% yield (12.5 mg, 0.03 mmol) from flash column chromatography (DCM:MeOH 20:1–9:1). ${\left[\alpha \right]}_{D}^{25}$ −13.5 (c 0.6, CH_3_OH). ^1^H NMR (CD_3_OD, 400 MHz): 7.66–7.59 (m, 2H, Ar*H*), 4.45 (d, 1H, *J* 6.8 Hz, H-1), 4.00 (td, 1H, *J* 6.0 Hz, *J* 2.0 Hz, H-5), 3.87–3.84 (m, 2H, H-2, H-4), 3.76 (d, 2H, *J* 5.6 Hz, H-6a, H-6b), 3.66 (dd, 1H, *J* 14.0 Hz, *J* 6.0 Hz, C*H*_2_NH), 3.59 (dd, 1H, *J* 14.0 Hz, *J* 9.2 Hz, C*H*_2_NH), 3.53 (s, 3H, OC*H*_3_), 2.40–2.40–2.34 (m, 1H, H-3). ^13^C NMR (CD_3_OD, 100 MHz): 166.7, 152.3 (ddd, 248.3 Hz, *J* 9.8 Hz, *J* 3.8 Hz), 143.0 (dt, *J* 254 Hz, *J* 16 Hz,), 132.2–132.0 (m), 113.1 (dd, *J* 17 Hz, *J* 6.1 Hz), 103.7, 75.9, 68.8, 68.5, 63.1, 56.8, 46.3, 38.0. ^19^F NMR (CD_3_OD, 376 MHz): −135.7 (d, *J* 20 Hz). −159.1 (t, *J* 20 Hz). HRMS calcd for C_15_H_18_F_3_NO_6_+H^+^ (M+H)^+^: 366.1164, found: 366.1161.

#### 3.12.14. Methyl 3-Deoxy-3-*C*-(2,3,4,5,6-Pentafluorobenzamidomethyl)-β-d-Galactopyranoside **7g**

Compound **7g** (TLC, DCM/MeOH, 10:1, R_f_ 0.38) was prepared according to the general procedure *3.12* from the amide **22g** (30 mg, 0.04 mmol). Obtained as a colorless oil in 83% yield (14.9 mg, 0.04 mmol) from flash column chromatography (DCM:MeOH 20:1–8:1). ${\left[\alpha \right]}_{D}^{25}$ −18.9 (c 0.6, CH_3_OH). ^1^H NMR (CD_3_OD, 400 MHz): 4.46 (d, 1H, *J* 6.4 Hz, H-1), 3.94–3.85 (m, 4H, H-2, H-4, H-5), 3.79 (dd, 1H, *J* 6.0 Hz, *J* 11.2 Hz, H-6a), 3.79 (dd, 1H, *J* 5.2 Hz, *J* 11.2 Hz, H-6a), 3.68 (dd, 1H, *J* 6.4 Hz, *J* 14.4 Hz, C*H*_2_NHCO), 3.62 (dd, 1H, *J* 14.4 Hz, *J* 8.8 Hz, C*H*_2_NHCO), 3.53 (s, 3H, OC*H*_3_), 2.38–2.32 (m, 1H, H-3). ^13^C NMR (CD_3_OD, 100 MHz): 159.9, 146.4, 144.0, 140.2, 137.6, 103.6, 75.8, 68.5, 68.4, 63.2, 56.9, 46.3, 37.7. ^19^F NMR (CD_3_OD, 376 MHz): −143.8 to −143.2 (m), −155.2 to −155.3 (m), −163.7 to −163.9 (m). HRMS calcd for C_15_H_16_F_5_NO_6_+H^+^ (M+H)^+^: 402.0976, found: 402.0974.

#### 3.12.15. Methyl 3-Deoxy-3-*C*-(3-Methoxybenzamidomethyl)-β-d-Galactopyranoside **7h**

Compound **7h** (TLC, DCM/MeOH, 10:1, R_f_ 0.42) was prepared according to the general procedure *3.12* from the amide **22h** (28 mg, 0.03 mmol). Obtained as a colorless oil in 66% yield (10.3 mg, 0.03 mmol) from flash column chromatography (DCM:MeOH 20:1–10:1). ${\left[\alpha \right]}_{D}^{25}$ −9.5 (c 0.5, CH_3_OH). ^1^H NMR (CD_3_OD, 400 MHz): 8.47 (t, *J* 5.2 Hz, CON*H*), 7.38–7.33 (m, 3H, Ar*H*), 7.08 (m, 1H, Ar*H*), 4.47 (d, 1H, *J* 7.2 Hz, H-1), 3.94 (td, 1H, *J* 5.6 Hz, *J* 2.0 Hz, H-5), 3.88–3.85 (m, 2H, H-2, H-4), 3.84 (s, 3H, OCH_3_), 3.78 (d, 2H, *J* 5.6 Hz, H-6a, H-6b), 3.66–3.62 (m, 2H, C*H*_2_NN), 3.53 (s, 3H, OC*H*_3_), 2.41–2.35 (m, 1H, H-3). ^13^C NMR (CD_3_OD, 100 MHz): 170.3, 161.3, 137.0, 130.7, 120.3, 118.5, 113.6, 103.7, 75.9, 70.0, 68.6, 63.1, 56.9, 55.9, 46.4, 37.8. HRMS calcd for C_16_H_23_NO_7_+H^+^ (M+H)^+^: 342.1553, found: 342.1555.

#### 3.12.16. Methyl 3-Deoxy-3-*C*-(p-Toluamidomethyl)-β-d-Galactopyranoside **7i**

Compound **7i** (TLC, DCM/MeOH, 10:1, R_f_ 0.48) was prepared according to the general procedure *3.12* from the amide **22i** (32 mg, 0.05 mmol). Obtained as a colorless oil in 53% yield (11.9 mg, 0.04 mmol) from flash column chromatography (DCM:MeOH 20:1–10:1). ${\left[\alpha \right]}_{D}^{25}$ −4.8 (c 0.5, CH_3_OH). ^1^H NMR (CD_3_OD, 400 MHz): 7.72–7.69 (m, 2H, Ar*H*), 7.27 (d, 2H, *J* 8.0 Hz, Ar*H*), 4.47 (d, 1H, *J* 7.2 Hz, H-1), 3.94 (td, 1H, *J* 5.6 Hz, *J* 2.0 Hz, H-5), 3.88–3.85 (m, 2H, H-2, H-4), 3.77 (d, 2H, *J* 5.6 Hz, H-6a, H-6b), 3.66–3.62 (m, 2H, C*H*_2_NN), 3.53 (s, 3H, OC*H*_3_), 2.39–2.34 (m, 1H, H-3, C*H*_3_). ^13^C NMR (CD_3_OD, 100 MHz): 170.4, 143.4, 132.7, 130.2, 128.2, 103.7, 76.0, 69.0, 68.7, 63.2, 56.9, 46.4, 37.7, 21.4. HRMS calcd for C_16_H_23_NO_6_+H^+^ (M+H)^+^: 326.1604, found: 326.1603.

#### 3.12.17. Methyl 3-Deoxy-3-*C*-(3,5-Dimethoxybenzamidomethyl)-β-d-Galactopyranoside **7j**

Compound **7j** (TLC, DCM/MeOH, 10:1, R_f_ 0.43) was prepared according to the general procedure *3.12* from the amide **22j** (24 mg, 0.04 mmol). Obtained as a colorless oil in 62% yield (10 mg, 0.03 mmol) from flash column chromatography (DCM:MeOH 20:1–9:1). ${\left[\alpha \right]}_{D}^{25}$ −25.7 (c 0.5, CH_3_OH). ^1^H NMR (CD_3_OD, 400 MHz): 6.96 (d, 1H, *J* 2.0 Hz, Ar*H*), 6.63 (t, 1H, *J* 2.0 Hz, Ar*H*), 4.47 (d, 1H, *J*_1,2_ 7.2 Hz, H-1), 3.94 (td, 1H, *J* 5.6 Hz, *J* 2.0 Hz, H-5), 3.88–3.85 (m, 2H, H-2, H-4), 3.82 (s, 6H, 2×OC*H*_3_), 3.77 (d, 2H, *J* 6.0 Hz), 3.67–3.57 (m, 2H, C*H_2_*NH), 3.53 (s, 3H, OC*H*_3_), 2.40–2.34 (m, 1H, H-3). ^13^C NMR (CD_3_OD, 100 MHz): 170.2, 162.4, 137.6, 106.1, 104.5, 103.7, 75.9, 69.0, 68.6, 63.1, 56.8, 56.0, 46.4, 37.8. HRMS calcd for C_17_H_25_NO_8_+H^+^ (M+H)^+^: 372.1658, found: 372.1663.

#### 3.12.18. Methyl 3-Deoxy-3-*C*-(3-Trifluoromethylbenzamidomethyl)-β-d-Galactopyranoside **7k**

Compound **7k** (TLC, DCM/MeOH, 10:1, R_f_ 0.51) was prepared according to the general procedure *3.12* from the amide **22k** (25 mg, 0.04 mmol). Obtained as a colorless oil in 66% yield (11.2 mg, 0.03 mmol) from flash column chromatography (DCM:MeOH 20:1–10:1). ${\left[\alpha \right]}_{D}^{25}$ −3.9 (c 0.6, CH_3_OH). ^1^H NMR (CD_3_OD, 400 MHz): 8.13 (s, 1H, Ar*H*), 8.08 (t, 1H, *J* 8.0 Hz, ArH), 7.85 (d, 1H, *J* 8.0 Hz, Ar*H*), 7.68 (t, 1H, *J* 8.0 Hz, Ar*H*), 4.47 (d, 1H, *J*_1,2_ 7.2 Hz, H-1), 3.95 (td, 1H, *J* 6.0 Hz, *J* 2.0 Hz, H-5), 3.89–3.86 (m, 2H, H-2, H-4), 3.79 (dd, 1H, *J*_6a,6b_ 12.0 Hz, *J*_5,6a_ 6.0 Hz, H-6a), 3.76 (dd, 1H, *J*_6a,6b_ 12.0 Hz, *J*_5,6b_ 6.0 Hz, H-6b), 3.69 (dd, 1H, *J* 13.6 Hz, *J* 5.6 Hz, C*H*_2_NH), 3.63 (dd, 1H, *J* 13.6 Hz, *J* 9.2 Hz, C*H*_2_NH), 3.54 (s, 3H, OC*H*_3_), 2.43–2.37 (m, 1H, H-3). ^13^C NMR (CD_3_OD, 125 MHz): 168.7, 136.7, 132.0 (q, *J* 32 Hz), 131.9, 130.6, 129.2 (q *J* 3.6 Hz), 125.4 (q, *J* 270 Hz), 125.1 (q, *J* 4.0 Hz), 103.7, 75.9, 68.9, 68.6, 63.1, 56.9, 46.5, 37.8. ^19^F NMR (CD_3_OD, 376 MHz): −64.2. HRMS calcd for C_16_H_20_F_3_NO_6_+H^+^ (M+H)^+^: 380.1321, found: 380.1321.

#### 3.12.19. Methyl 3-Deoxy-3-*C*-(4-Phenylbenzamidomethyl)-β-d-Galactopyranoside **7l**

Compound **7l** (TLC, DCM/MeOH, 10:1, R_f_ 0.54) was prepared according to the general procedure *3.12* from the amide **22l** (39 mg, 0.06 mmol). Obtained as a colorless oil in 67% yield (15.4 mg, 0.04 mmol) from flash column chromatography (DCM:MeOH 20:1–12:1). ${\left[\alpha \right]}_{D}^{25}$ −21.4 (c 0.7, CH_3_OH). ^1^H NMR (CD_3_OD, 400 MHz): 7.70 (dd, 2H, *J* 6.8 Hz, *J* 2.0 Hz, Ar*H*), 7.71 (dd, 2H, *J* 6.8 Hz, *J* 2.0 Hz, Ar*H*), 7.65 (m, 2H, Ar*H*), 7.48–7.44 (m, 2H, Ar*H*), 7.40–7.35 (m, 1H, Ar*H*), 4.49 (d, 1H, *J*_1,2_ 7.2 Hz, H-1), 3.96 (td, 1H, *J* 6.0 Hz, *J* 2.4 Hz, H-5), 3.82–3.75 (m, 2H, H-6a, H-6b), 3.73–3.62 (m, 2H, C*H_2_*NH), 3.54 (s, 3H, OC*H*_3_), 2.44–2.38 (m, 1H, H-3). ^13^C NMR (CD_3_OD, 100 MHz): 170.1, 145.7, 141.2, 134.2, 130.0, 129.1, 128.8, 128.11, 128.07, 103.7, 76.0, 69.0, 68.7, 63.2, 56.9, 46.4, 37.8. HRMS calcd for C_21_H_25_NO_6_+H^+^ (M+H)^+^: 388.1760, found: 388.1761.

#### 3.12.20. Methyl 3-Deoxy-3-*C*-(Diphenylphosphonamidomethyl)-β-d-Galactopyranoside **8**

Compound **8** (TLC, DCM/MeOH, 10:1, R_f_ 0.45) was prepared according to the general procedure 3.12 from the amide **23** (43 mg, 0.06 mmol). Obtained as a colorless oil in 50% yield (13.3 mg, 0.03 mmol) from flash column chromatography (DCM:MeOH 15:1–9:1). ${\left[\alpha \right]}_{D}^{25}$ −18.6 (c 0.7, CH_3_OH). ^1^H NMR (CD_3_OD, 400 MHz): 7.40 (t, 4H, *J* 8.0 Hz, Ar*H*), 7.29–7.21 (m, 6H, Ar*H*), 4.36 (d, 1H, *J* 7.2 Hz, H-1), 3.90 (dd, 1H, *J* 4.0 Hz, *J* 2.0 Hz, H-4), 3.83 (dd, 1H, *J* 7.2 Hz, *J* 5.6 Hz, H-2), 3.79–3.76 (m, 1H, H-5), 3.71 (dd, 1H, *J*_5,6a_ 10.4 Hz, *J*_6a,6b_ 4.4 Hz, H-6a), 3.71 (dd, 1H, *J*_5,6b_ 10.8 Hz, *J*_6a,6b_ 4.4 Hz, H-6b), 3.51 (s, 3H, OC*H*_3_), 3.50–3.44 (m, 1H, C*H*_2_NH), 3.17–3.08 (m, 1H, C*H*_2_NH), 2.28–2.22 (m, 1H, H-3). ^13^C NMR (CD_3_OD, 100 MHz): 152.2 (dd, *J* 6.2 Hz, *J* 2.7 Hz), 130.9, 126.3 (d, *J* 3.1 Hz), 121.4 (dd, *J* 4.6 Hz, *J* 11.1 Hz), 103.6, 75.7, 68.7, 68.1, 63.2, 56.8, 47.4 (d, 5.7 Hz), 39.2. ^31^P NMR (CD_3_OD, 162 MHz): −1.0. HRMS calcd for C_20_H_26_PNO_8_+H^+^ (M+H)^+^: 440.1474, found: 440.1470.

### 3.13. Methyl 2,3-Di-O-Acetyl-β-d-Gulopyranoside ***25***

Compound **24** (300 mg, 0.82 mmol) was dissolved in 80% aqueous AcOH (5 mL) and the solution was stirred at 80 °C for 2 h. When the TLC (TLC, heptane/EtOAc, 1:2, R_f_ 0.39) showed complete consumption of the starting material, the solvents were evaporated under reduced pressure and co-evaporated twice with toluene (10 mL). Then, the crude was purified via flash chromatography (Heptane/EtOAc, 3:1–1:2) to obtain pure compound **25** (191 mg, 0.69 mmol, 84%) as a white foam. ${\left[\alpha \right]}_{D}^{25}$ −30.4 (c 0.8, CHCl_3_). ^1^H NMR (CDCl_3_, 400 MHz): 5.35 (t, 1H, *J*_2,3_ 3.6 Hz, H-3), 5.10 (dd, 1H, *J*_1,2_ 8.0 Hz, *J*_2,3_ 3.6 Hz, H-2), 4.68 (d, 1H, *J* 8.0 Hz, H-1), 3.93–3.89 (m, 4H, H4, H-5, H-6a, H-6b), 3.52 (s, 3H, OC*H*_3_), 2.11 (s, 3H, COC*H*_3_), 2.03 (s, 3H, COC*H*_3_). ^13^C NMR (CDCl_3_, 100 MHz): 170.0, 169.9, 99.9, 73.3, 70.6, 69.0, 68.4, 62.8, 56.9, 20.98, 20.95. HRMS calcd for C_11_H_18_O_8_+Na^+^ (M+Na)^+^: 301.0899, found: 301.0898.

### 3.14. Methyl β-d-Gulopyranoside ***9***

Compound **25** (120 mg, 0.43 mmol) was dissolved in MeOH (3 mL). NaOMe (1.0 mL, 0.5 M in MeOH) was added and the solution was stirred at room temperature for 2 h (TLC, DCM/MeOH, 5:1, R_f_ 0.3). The solution was neutralized with DOWEX 50 W H^+^ resin, filtered and the solvents were evaporated under reduced pressure and the crude was purified via flash chromatography (DCM/MeOH, 7:1–3:1) to obtain pure compound **9** (46 mg, 0.23 mmol, 55%) as a colorless oil. ${\left[\alpha \right]}_{D}^{25}$ −19.2 (c 0.9, CH_3_OH). ^1^H NMR (D_2_O, 400 MHz): 4.60 (d, 1H, *J*_1,2_ 8.4 Hz, H-1), 4.05 (t, 1H, *J*_3,4_ 3.6 Hz, H-4), 4.00–3.96 (m, 1H, H-5), 3.80 (dd, 1H, *J*_3,4_ 3.6 Hz, *J*_4,5_ 1.2 Hz, H-4), 3.75 (dd, 1H, *J*_6a,6b_ 12.0 Hz, *J*_5,6a_ 6.4 Hz, H-6a), 3.76 (dd, 1H, *J*_6a,6b_ 12.0 Hz, *J*_5,6a_ 4.8 Hz, H-6b), 3.76 (dd, 1H, *J*_1,2_ 8.4 Hz, *J*_2,3_ 3.6 Hz, H-2), 3.56 (s, 3H, OC*H*_3_). ^13^C NMR (D_2_O, 100 MHz): 101.5, 73.8, 71.1, 69.3, 68.0, 61.0, 56.9. HRMS calcd for C_7_H_14_O_6_+Na^+^ (M+Na)^+^: 217.0688, found: 217.0687.

### 3.15. Methyl 3-Azido-2,4,6-Tri-O-Benzoyl-3-Deoxy-β-d-Gulopyranoside ***29***

Triflic anhydride (235 μL, 1.4 mmol) was added dropwise to a stirred solution of **26** (400 mg, 1.4 mmol) in DCM (10 mL) and pyridine (451 μL, 5.6 mmol) at −30 °C and under N_2_ atmosphere after which the reaction was allowed to reach rt under 2 h. BzCl (179 μL, 1.54 mmol) was added and the reaction was stirred for another 2 h before the reaction was diluted with DCM (25 mL) and washed with saturated NaHCO_3_ (2 × 25 mL). The combined aqueous phases were extracted with DCM (40 mL). The pooled organic phases were dried over MgSO_4_ and concentrated to give crude **27**. Sodium azide (637 mg, 9.8 mmol) was added to the crude **27** (≤1.4 mmol) in DMF (15 mL) and the reaction was stirred overnight at 70 °C under N_2_ atmosphere. The reaction was concentrated to give crude **28**, which was dissolved in 90% AcOH (20 mL) and heated at 80 °C for 3 h. The solvent was evaporated in vacuo and co-evaporated with toluene to remove the residual AcOH. The residue was dissolved in pyridine (15 mL), into the solution catalytic amount of DMAP and benzoyl chloride (488 μL, 4.2 mmol) was added subsequently. The solution was stirred at room temperature for 4 h when TLC (heptane/EtOAc, 4:1, R_f_ 0.48) showed complete conversion of the starting material to a faster moving spot. The solvents were evaporated in vacuo and co-evaporated with toluene to remove residual pyridine. The solid residue thus obtained was dissolved in EtOAc (50 mL) and washed with 1 N HCl (50 mL), followed by saturated NaHCO_3_ and brine (50 mL). The organic layer was collected, dried (Na_2_SO_4_), filtered and evaporated in vacuo. The crude was purified by flash chromatography using heptane/EtOAc (6:1 to 5:2) as the eluent to afford pure compound **29** (324 mg, 0.61 mmol, 43% over four steps) as a white amorphous mass. ${\left[\alpha \right]}_{D}^{25}$ −45.3 (c 0.7, CHCl_3_). ^1^H NMR (CDCl_3_, 400 MHz): 8.14–7.39 (m, 15H, Ar*H*), 5.51 (dd, 1H, *J*_1,2_ 7.6 Hz, *J*_2,3_ 4.0 Hz, H-2), 5.41 (dd, 1H, *J*_3,4_ 4.0 Hz, *J*_3,4_ 0.8 Hz, H-4), 5.00 (d, 1H, *J* 7.6 Hz, H-1), 4.66–4.61 (m, 1H, H-5), 4.52–4.45 (m, 3H, H-3, H-6a, H-6b), 3.58 (s, 3H, OC*H*_3_). ^13^C NMR (CDCl_3_, 100 MHz): 166.0, 165.3, 165.2, 133.8, 133.6, 133.2, 130.02, 129.98, 129.7, 129.5, 129.0, 128.7, 128.6, 128.5, 128.4, 99.6, 70.3, 70.1, 69.5, 62.4, 60.1, 57.0. HRMS calcd for C_28_H_25_N_3_O_8_+NH_4_^+^ (M+NH_4_)^+^: 549.1985, found: 549.1989.

### 3.16. Methyl 3-Amino-2,4,6-Tri-O-Benzoyl-3-Deoxy-β-d-Gulopyranoside ***30***

A solution of **29** (201 mg, 0.3784 mmol) in MeOH (7 mL) was stirred with Pd(OH)_2_/C (10% wt., 1 mg per 5 mg of crude, 40 mg) under hydrogen atmosphere at room temperature for 2 h. After the completion of the reaction (as indicated by TLC, heptane/EtOAc, 1:1, R_f_ 0.26), the reaction mixture was filtered through a Celite bed and washed with methanol. The filtrate was concentrated under reduced pressure and purified through flash column (heptane/EtOAc, 4:1–1:1) to get the desired compound as a white amorphous solid. Yield: 126 mg (0.2494 mmol, 66%). ${\left[\alpha \right]}_{D}^{25}$ −39.9 (c 0.8, CHCl_3_). ^1^H NMR (CDCl_3_, 400 MHz): 8.13–7.38 (m, 15H, Ar*H*), 5.38 (dd, 1H, *J*_1,2_ 7.2 Hz, *J*_2,3_ 4.0 Hz, H-2), 5.29 (dd, 1H, *J*_3,4_ 4.4 Hz, *J*_4,5_ 2.4 Hz, H-4), 5.11 (d, 1H, *J* 7.2 Hz, H-1), 4.83–4.79 (m, 1H, H-5), 4.64 (dd, 1H, dd, 1H, *J*_6a,6b_ 11.2 Hz, *J*_5,6a_ 6.8 Hz, H-6a), 4.51 (dd, 1H, dd, 1H, *J*_6a,6b_ 11.2 Hz, *J*_5,6b_ 6.0 Hz, H-6b), 3.90 (t, 1H, *J*_3,4_, *J*_2,3_ 4.0 Hz, H-3), 3.57 (s, 3H, OC*H*_3_), 1.97 (bs, 1H, N*H*_2_). ^13^C NMR (CDCl_3_, 100 MHz): 166.3, 165.9, 165.3, 133.7, 133.5, 133.2, 130.1, 129.9, 129.8, 128.69, 128.67, 128.5, 99.3, 72.1, 71.7, 70.2, 63.3, 57.1, 50.6. HRMS calcd for C_28_H_27_NO_8_+H^+^ (M+H)^+^: 506.1815, found: 506.1817.

### 3.17. Methyl 3-Benzamido-2,4,6-Tri-O-Benzoyl-3-Deoxy-β-d-Gulopyranoside ***31***

Compound **30** was dissolved in pyridine (3 mL), into the solution catalytic amount of DMAP and benzoyl chloride (29 μL, 0.2464 mmol) was added subsequently. The solution was stirred at room temperature for 3 h when TLC (heptane/EtOAc, 1:1, R_f_ 4.8) showed complete conversion of the starting material to a faster moving spot. The solvents were evaporated in vacuo and co-evaporated with toluene to remove residual pyridine. The solid residue thus obtained was dissolved in EtOAc (7 mL) and washed with 1 (N) HCl (5 mL), followed by saturated NaHCO_3_ and brine (5 mL). The organic layer was collected, dried over Na_2_SO_4_, filtered and evaporated in vacuo. The crude was purified by flash chromatography using heptane/EtOAc (7:1 to 3:1) as the eluent to afford pure compound **31** (77 mg, 0.1265 mmol, 77%) as a white amorphous solid. ${\left[\alpha \right]}_{D}^{25}$ −48.8 (c 0.6, CHCl_3_). ^1^H NMR (CDCl_3_, 400 MHz): 8.11–7.29 (m, 20H, Ar*H*), 6.60 (d, 1H, *J*_3,N*H*COPh_ 8.4 Hz, N*H*COPh), 5.96 (dd, 1H, *J* 10.8 Hz, *J* 6.0 Hz, H-4), 5.55 (t, 1H, *J* 2.8 Hz, H-2), 5.34–5.29 (m, 1H, H-3), 4.99 (d, 1H, *J*_1,2_ 2.8 Hz, H-1), 4.92 (dd, 1H, *J*_6a,6b_ 11.6 Hz, *J*_5,6a_ 5.6 Hz, H-6a), 4.86 (dd, 1H, *J*_6a,6b_ 11.6 Hz, *J*_5,6b_ 6.4 Hz, H-6a), 4.77 (dd, 1H, *J*_6a,6b_ 12.4 Hz, *J*_4,5_ 6.0 Hz, H-5), 3.61 (s, 3H, OC*H*_3_). ^13^C NMR (CDCl_3_, 100 MHz): 167.4, 166.8, 166.2, 165.5, 133.9, 133.8, 133.7, 133.0, 131.7, 130.0, 129.9, 129.7, 129.6, 129.1, 128.7, 128.59, 128.57, 128.3, 127.0, 99.5, 72.4, 71.8, 68.4, 64.5, 60.4, 56.8, 46.3. HRMS calcd for C_35_H_31_NO_9_+H^+^ (M+H)^+^: 610.2077, found: 610.2081.

### 3.18. Methyl 3-Benzamido-3-Deoxy-β-d-Gulopyranoside ***10***

Compound **31** (54 mg, 0.0886 mmol) was dissolved in MeOH (2 mL). NaOMe (0.5 mL, 0.5 M in MeOH) was added and the solution was stirred at room temperature for 12 h (TLC, DCM/MeOH, 10:1, R_f_ 0.4). The solution was neutralized with DOWEX 50 W H+ resin, filtered and the solvents were evaporated under reduced pressure and the residue was purified by a short flash column using DCM–MeOH (9:1) to afford the compound **10** (19.2 mg, 0.0646 mmol, 73%). ${\left[\alpha \right]}_{D}^{25}$ −18.3 (c 0.6, CH_3_OH). ^1^H NMR (CD_3_OD, 400 MHz): 7.83 (d, 2H, *J* 7.6 Hz, Ar*H*), 7.57–7.44 (m, 3H, Ar*H*), 4.70 (d, 1H, *J*_1,2_ 8.4 Hz, H-1), 4.48–4.44 (m, 1H, H-3), 4.01 (dd, 1H, *J*_3,4_ 3.6 Hz, *J*_4,5_ 1.2 Hz, H-4), 3.94 (dd, 1H, *J*_1,2_ 7.6 Hz, *J*_2,3_ 5.2 Hz, H-2), 3.84 (td, 1H, *J*_5,6a,_ *J*_5,6a_ 6.0 Hz, *J*_4,5_ 1.6 Hz, H-5), 3.77 (dd, 1H, *J*_6a,6b_ 11.2 Hz, *J*_5,6a_ 6.0 Hz, H-6a), 3.74 (dd, 1H, *J*_6a,6b_ 11.2 Hz, *J*_5,6a_ 6.0 Hz, H-6b), 3.57 (s, 3H, OC*H*_3_). ^13^C NMR (CD_3_OD, 100 MHz): 171.4, 164.6, 135.9, 132.7, 129.5, 128.6, 103.4, 75.7, 68.8, 67.8, 62.6, 56.9, 56.0. HRMS calcd for C_14_H_19_NO_6_+H^+^ (M+H)^+^: 298.1291, found: 298.1289.

### 3.19. Expression Constructs, Expression, and Purification of Recombinant Galectins

Human galectin-1 [25], galectin-2 [26], galectin-3 [27], galectin-4N [19], galectin-4C [19], galectin-8N [28], galectin-8C [28], galectin-9N [29], and galectin-9C [30], were expressed and purified as described earlier. Human galectin-7 was expressed using a pET3c plasmid in *E. coli* BL21-star. The plasmid containing expression optimized DNA encoding the full human galectin-7 sequence (NCBI Reference Sequence: NP_002298.1) was obtained from GenScript (Piscataway, NJ, USA). Bacterial culture and induction, and galectin purification was essential as described for galectin-3 expressed with the same vector [27]; a typical yield was 1.5–2 mg/L culture. Lactose was removed by chromatography on a PD-10 column (Amersham Biosciences) with repeated ultrafiltration with Centriprep (Amicon).

### 3.20. Fluorescence Polarization Assay

Fluorescence polarization experiments were carried out either with a POLARStar plate reader and FLUOstar Galaxy software or with a PheraStarFS plate reader and PHERAstar Mars version 2.10 R3 software (BMG, Offenburg, Germany). The dissociation constant (K_d_) values were determined in PBS as described earlier [18,19]. Specific conditions for galectin-1, 2, 3, 4N, 4C, 8N, 8C, 9N, and 9C were kept as reported [29]. Experiments were performed at room temperature with human galectin-7 at 5 µM and the fluorescent probe β-d-galactopyranosyl-(1–4)-2-acetamido-2-deoxy-β-d-glucopyranosyl-(1–3)-β-d-galactopyranosyl-(1–4)-(*N*1-fluorescein-5-yl-carbonylaminomethylcarbonyl)-β-d-glucopyranosylamine [29] at 0.02 µM. All the compounds in Table 1 except 32 were dissolved in a neat DMSO at 100 mM and diluted in PBS to three to six different concentrations to be tested in duplicate. Average K_d_ values and SEMs were calculated from 2–8 single-triple point measurements showing between 30%–70% inhibition.

## 4. Conclusions

In summary, we report the synthesis and discovery of 3-*C*-methyl-guloside derivatives as highly selective galectin-1 inhibitors with 3-*C*-benzamidomethyl-3-deoxy-gulosides being the most selective structural class. The reason for the exceptional galectin-1-selectivites discovered remains to be elucidated as molecular modelling failed to provide insight into this and experimental structural studies by X-ray diffraction or nmr spectroscopy are likely necessary. Although the galectin-1 affinities are in the high-µM to low mM range, they are significantly higher affinity than that of simple galactosides, such as methyl β-d-galactopyranoside, and thus points towards a novel structural class and synthetic route towards the discovery of galectin-1 inhibitors with high selectivity. This is important in light of the roles of galectin-1 in tumor progression and immune regulation [31,32]. 

## Figures and Tables

**Table 1:** *K*_d_-values (mM)^a^ of compounds **1a**–**1b**, **2**–**3**, **7a**, **8**, and the reference methyl β-d-galactopyranoside **32** against human galectin-1, 2, 3, 4N, 4C, 7, 8N, 8C, 9N, and 9C in a competitive fluorescence polarization assay [15,16].

Compounds	Galectin									
	1	2	3	4N ^b^	4C ^c^	7	8N ^b^	8C ^c^	9N^b^	9C ^c^
**1a**	1.3 ± 0.15	ND ^d^	NB ^e^	NB	ND	NB	NB	3.7 ± 0.02	NB	NB
**1b**	NB	NB	NB	>4	NB	NB	NB	NB	NB	NB
**2**	NB	NB	NB	NB	NB	NB	NB	NB	NB	NB
**3**	NB	ND	NB	ND	ND	ND	NB	NB	ND	ND
**4**	NB	NB	NB	NB	NB	NB	NB	NB	NB	NB
**5**	NB	NB	NB	NB	NB	NB	NB	NB	NB	NB
**6**	>10	ND	NB	NB	NB	NB	NB	NB	ND	NB
**7a**	1.8 ± 0.15	NB	NB	NB	NB	NB	NB	NB	ND	NB
**8**	>10	ND	NB	NB	NB	NB	NB	NB	ND	>5
**32**	>10 [16]	13 [17]	4.4 [16]	6.6 [17]	10 [17]	4.8 [16]	6.3 [16]	>30 [18]	3.3 [16]	8.6 [19]

^a^ The data are average and SEM (standard error of mean) of 4–8 single-triple point measurements. ^b^ N-terminal domain. ^c^ C-terminal domain. ^d^ Not determined. ^e^ Not binding at the highest concentration tested: 4 mM.

**Table 2:** *K*_d_-values (mM)^a^ of compounds **7a**–**7l**, **9**, and **10** against human galectin-1, 2, 3, 4N, 4C, 7, 8N, 8C, 9N, and 9C in a competitive fluorescence polarization assay.

Compounds	Galectin									
	1	2	3	4N ^b^	4C ^c^	7	8N ^b^	8C ^c^	9N ^b^	9C ^c^
**7a**	1.8 ± 0.15	NB ^d^	NB	NB	NB	NB	NB	NB	ND ^e^	NB
**7b**	1.5 ± 0.08	NB	NB	1.9 ± 0.05	NB	NB	NB	2.7 ± 0.5	NB	NB
**7c**	1.9 ± 0.04	NB	NB	2.2 ± 0.16	NB	NB	NB	NB	NB	NB
**7d**	1.9 ± 0.4	NB	NB	NB	NB	NB	NB	NB	NB	NB
**7e**	1.7 ± 0.06	NB	1.6 ± 0.03	1.7 ± 0.07	NB	NB	NB	NB	NB	NB
**7f**	2.5 ± 0.4	NB	NB	NB	NB	NB	NB	NB	NB	NB
**7g**	0.7 ± 0.005	NB	NB	NB	NB	NB	NB	NB	NB	NB
**7h**	3.2 ± 0.5	NB	NB	NB	NB	NB	NB	NB	NB	NB
**7i**	2.3 ± 0.4	NB	NB	NB	NB	NB	NB	NB	NB	NB
**7j**	1.8 ± 0.04	NB	NB	2 ± 0.4	NB	NB	NB	2.6 ± 0.6	NB	NB
**7k**	1.8 ± 0.07	NB	NB	1.9 ± 0.1	NB	NB	NB	NB	NB	NB
**7l**	NB	NB	NB	NB	NB	NB	NB	NB	NB	NB
**9**	10 ± 0.25	10 ± 1.5	NB	ND	11 ± 1.2	NB	NB	NB	NB	NB
**10**	NB	NB	NB	1.3 ± 0.2	NB	ND	NB	NB	NB	NB

^a^ The data are average and SEM of 4–8 single-triple point measurements. ^b^ N-terminal domain. ^c^ C-terminal domain. ^d^ Not binding at the highest concentration tested: 4 mM. ^e^ Not determined.

## Supplementary Materials

Supplementary materials can be found at https://www.mdpi.com/1422-0067/20/15/3786/s1.

## Abbreviations

Ac	Acetyl
Bn	Benzyl
DCM	Dichloromethane
THF	Tetrahydrofuran
DMF	Dimethylformamide
DIPEA	Diisopropylethylamine
AcOH	AcOH
EtOAc	EtOAc
TLC	Thin layer chromatography
HPLC	High-performance liquid chromatography
HRMS	High resolution mass spectrometry
DMSO	Dimethylsulfoxide
µM	Micromolar
mM	Milimolar
9-BBN	9-Borabicyclo[3.3.1]nonane
DMAP	4-Dimethylaminopyridine
