# Supplementary material for: A Galactoside-Binding Protein Tricked into Binding Unnatural Pyranose Derivatives: 3-Deoxy-3-Methyl-Gulosides Selectively Inhibit Galectin-1
Source: Int J Mol Sci. 2019 Aug 2;20(15):3786. doi: 10.3390/ijms20153786 (PMC6696278; doi:10.3390/ijms20153786)
Supplement: Supplementary file 1 [file ijms-20-03786-s001.pdf]

Supporting Information

A Galactoside-Binding Protein Tricked into Binding Unnatural Pyranose Derivatives: 3-Deoxy-3-Methylene Gulosides Selectively Inhibit Galectin-1

Kumar Bhaskar Pal [1], Mukul Mahanti [1], Hakon Leffler [2] and Ulf J. Nilsson [1,*]

Copy of NMR Spectra

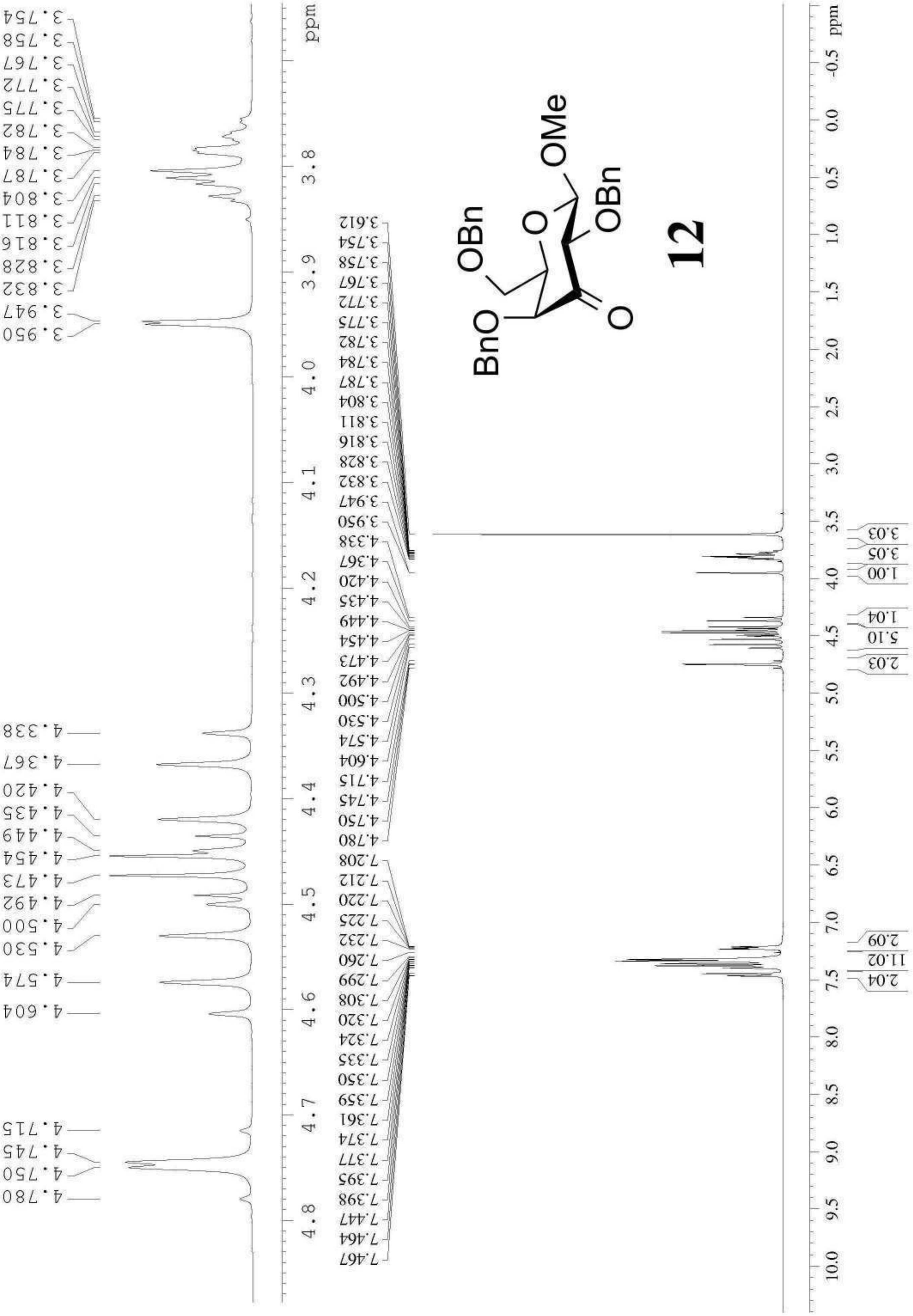

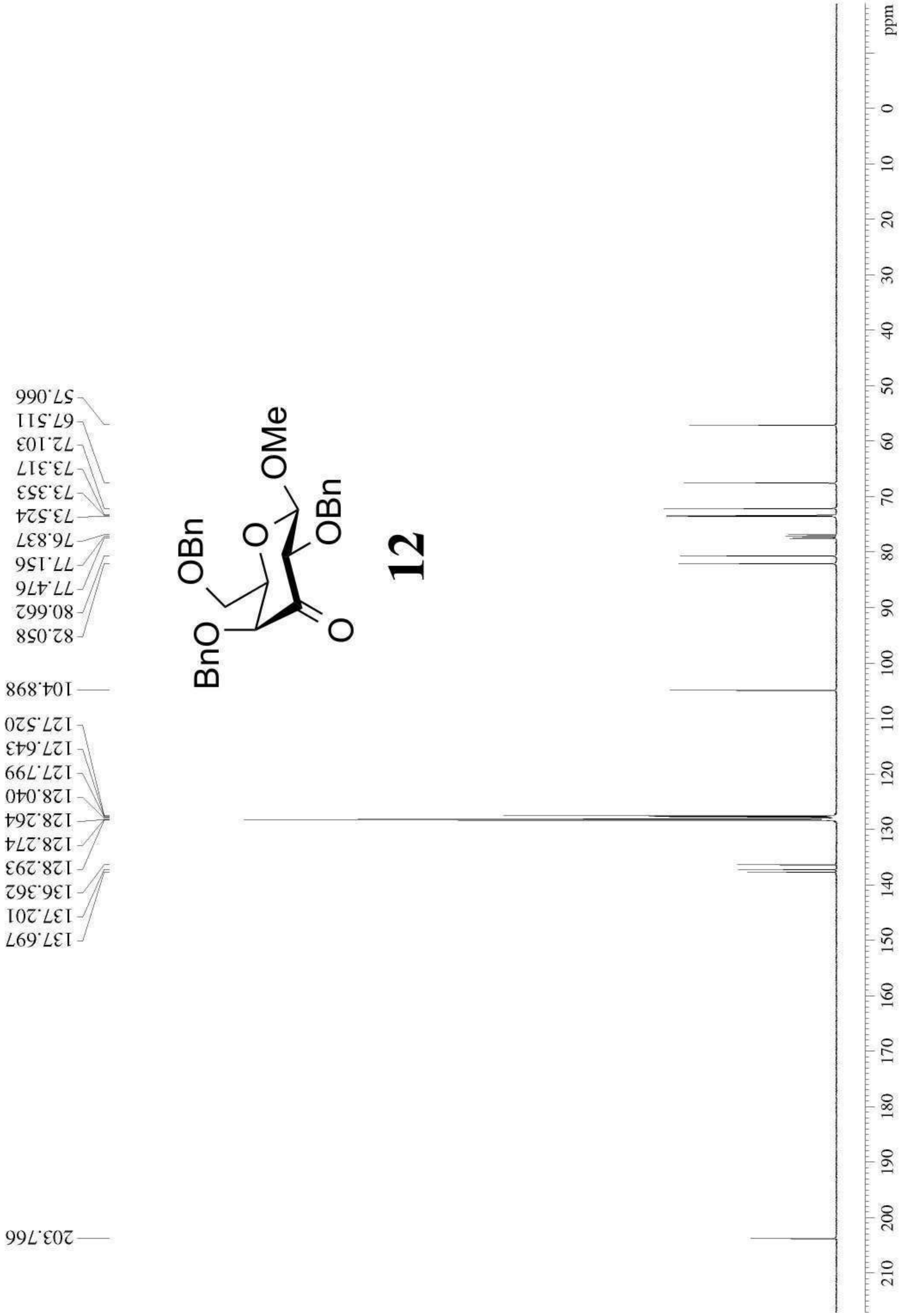

57.066
67.511
72.103
73.317
73.353
73.524
76.837
77.156
77.476
80.662
82.058
104.898
127.520
127.643
127.799
128.040
128.264
128.274
128.293
136.362
137.201
137.697
203.766
BnO
OBn
O
OMe
OBn
O
12
ppm

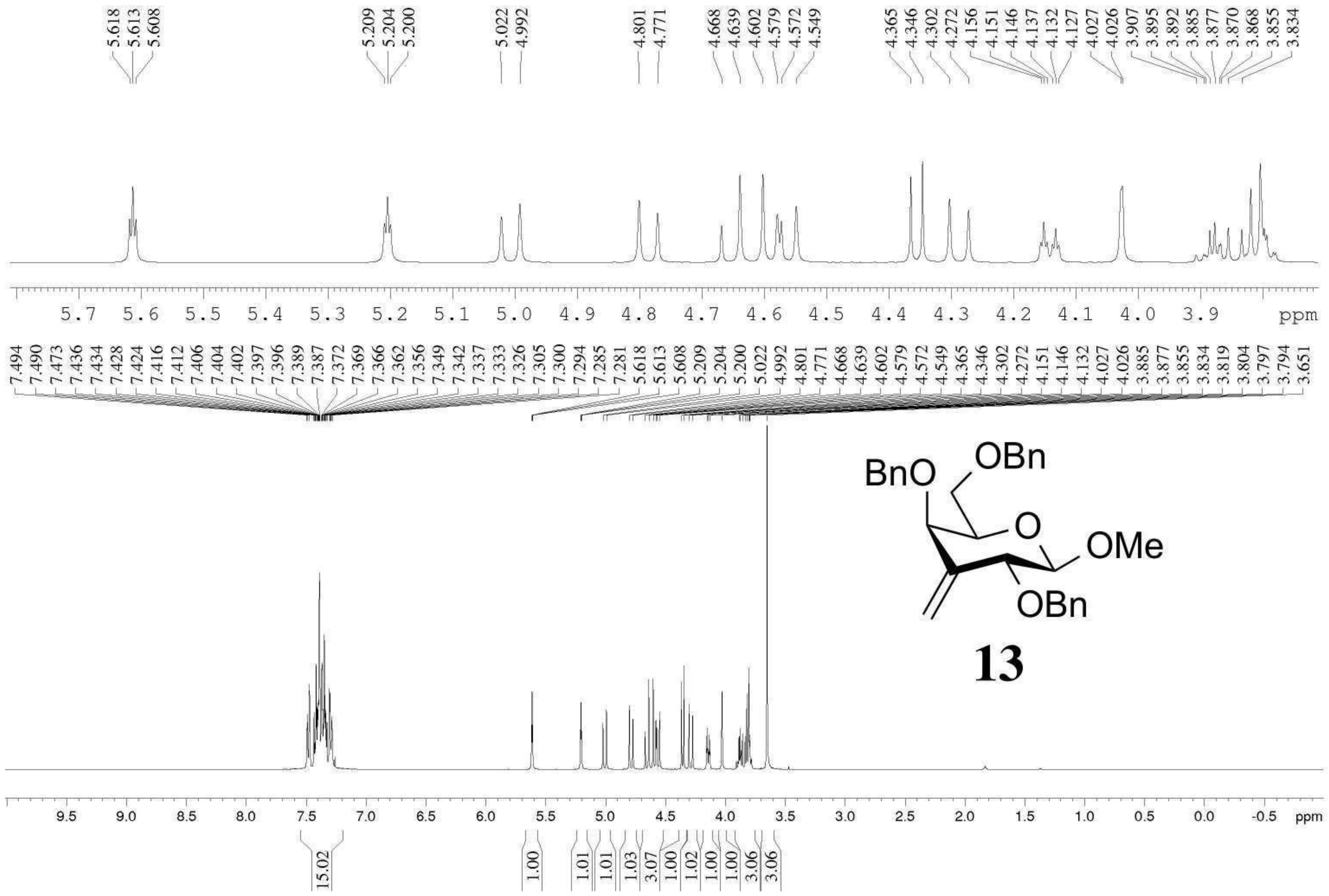
5.618 5.613 5.608 5.209 5.204 5.200 5.022 4.992 4.801 4.771 4.668 4.639 4.602 4.579 4.572 4.549 4.365 4.346 4.302 4.272 4.156 4.151 4.146 4.137 4.132 4.127 4.027 4.026 3.907 3.895 3.892 3.885 3.877 3.870 3.868 3.855 3.834
5.7 5.6 5.5 5.4 5.3 5.2 5.1 5.0 4.9 4.8 4.7 4.6 4.5 4.4 4.3 4.2 4.1 4.0 3.9 ppm
7.494 7.490 7.473 7.436 7.434 7.428 7.424 7.416 7.412 7.406 7.404 7.402 7.397 7.396 7.389 7.387 7.372 7.369 7.366 7.362 7.356 7.349 7.342 7.337 7.333 7.326 7.305 7.300 7.294 7.285 7.281 5.618 5.613 5.608 5.209 5.204 5.200 5.022 4.992 4.801 4.771 4.668 4.639 4.602 4.579 4.572 4.549 4.365 4.346 4.302 4.272 4.151 4.146 4.132 4.027 4.026 3.885 3.877 3.855 3.834 3.819 3.804 3.797 3.794 3.651
9.5 9.0 8.5 8.0 7.5 7.0 6.5 6.0 5.5 5.0 4.5 4.0 3.5 3.0 2.5 2.0 1.5 1.0 0.5 0.0 -0.5 ppm
15.02 1.00 1.01 1.01 1.03 3.07 1.00 1.02 1.00 1.00 3.06 3.06
BnO
OBn
O
OMe
OBn

13

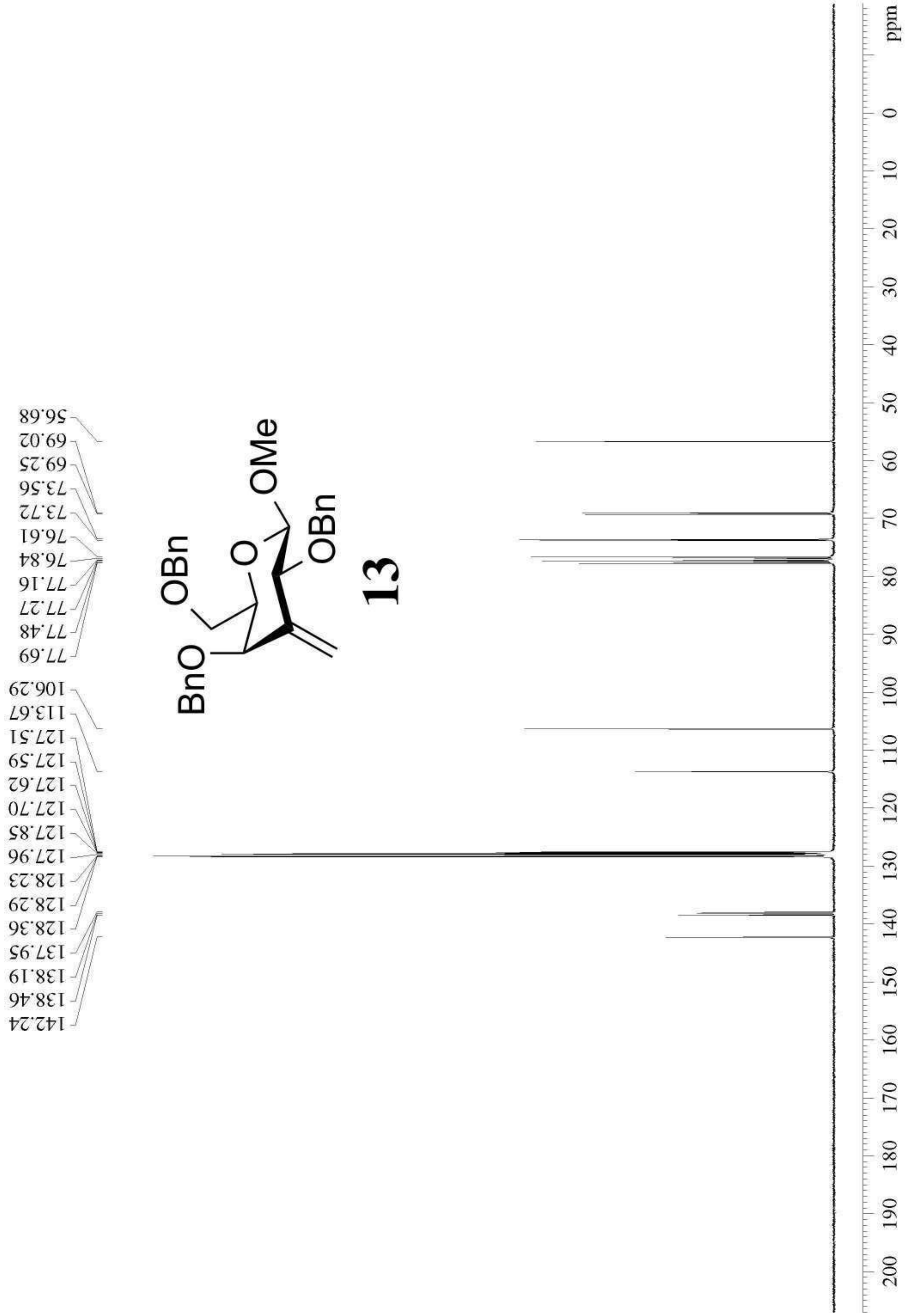
56.68
69.02
69.25
73.56
73.72
76.61
76.84
77.16
77.27
77.48
77.69
106.29
113.67
127.51
127.59
127.62
127.70
127.85
127.96
128.23
128.29
128.36
137.95
138.19
138.46
142.24
BnO
OBn
O
OMe
OBn
13
ppm
0
10
20
30
40
50
60
70
80
90
100
110
120
130
140
150
160
170
180
190
200

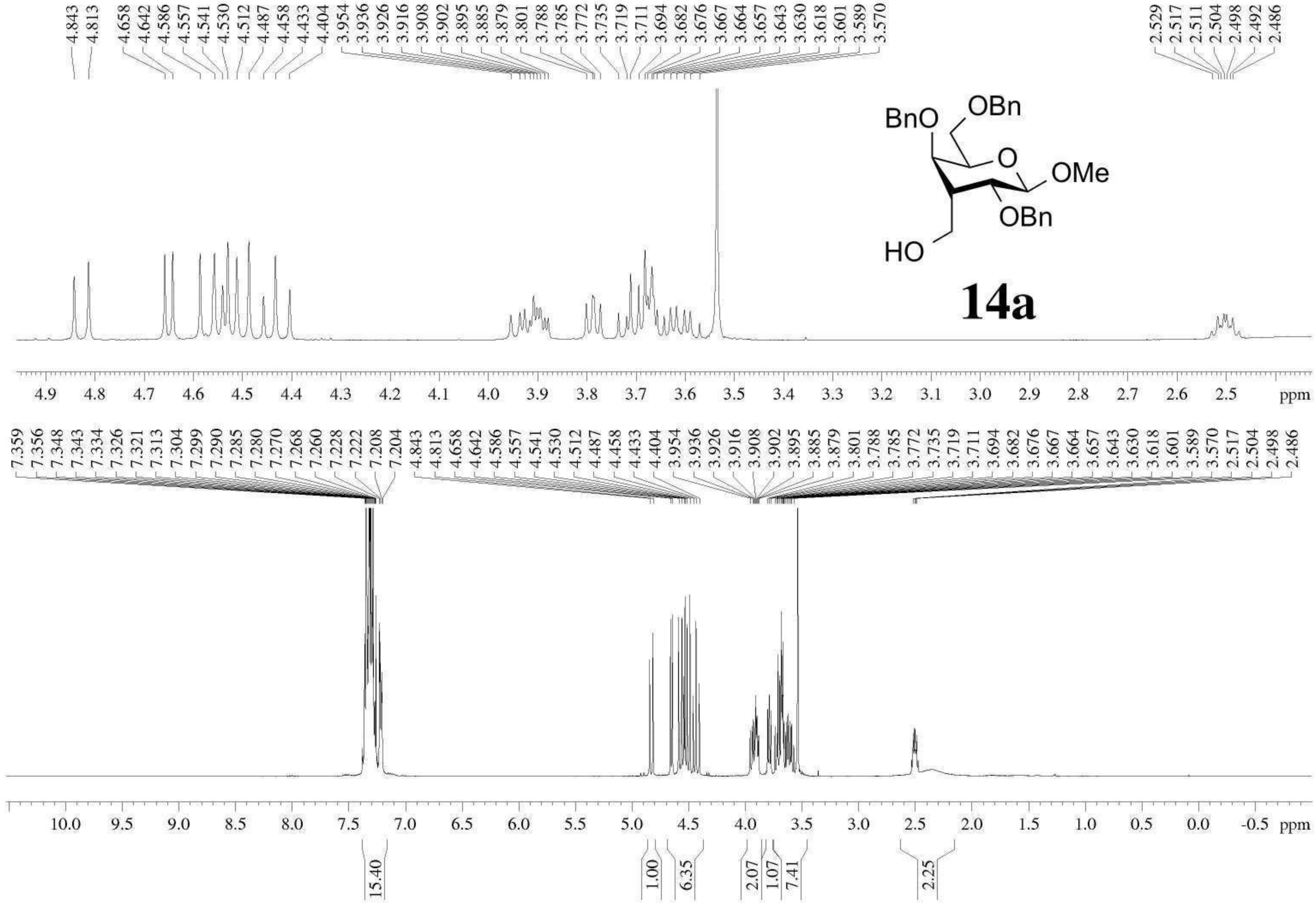
4.843 4.813 4.658 4.642 4.586 4.557 4.541 4.530 4.512 4.487 4.458 4.433 4.404 3.954 3.936 3.926 3.916 3.908 3.902 3.895 3.885 3.879 3.801 3.788 3.785 3.772 3.735 3.719 3.711 3.694 3.682 3.676 3.667 3.664 3.657 3.643 3.630 3.618 3.601 3.589 3.570
2.529 2.517 2.511 2.504 2.498 2.492 2.486
BnO
OBn
O
OMe
OBn
HO
14a
4.9 4.8 4.7 4.6 4.5 4.4 4.3 4.2 4.1 4.0 3.9 3.8 3.7 3.6 3.5 3.4 3.3 3.2 3.1 3.0 2.9 2.8 2.7 2.6 2.5 ppm
7.359 7.356 7.348 7.343 7.334 7.326 7.321 7.313 7.304 7.299 7.290 7.285 7.280 7.270 7.268 7.260 7.228 7.222 7.208 7.204 4.843 4.813 4.658 4.642 4.586 4.557 4.541 4.530 4.512 4.487 4.458 4.433 4.404 3.954 3.936 3.926 3.916 3.908 3.902 3.895 3.885 3.879 3.801 3.788 3.785 3.772 3.735 3.719 3.711 3.694 3.682 3.676 3.667 3.664 3.657 3.643 3.630 3.618 3.601 3.589 3.570 2.517 2.504 2.498 2.486
10.0 9.5 9.0 8.5 8.0 7.5 7.0 6.5 6.0 5.5 5.0 4.5 4.0 3.5 3.0 2.5 2.0 1.5 1.0 0.5 0.0 -0.5 ppm
15.40
1.00
6.35
2.07
1.07
7.41
2.25

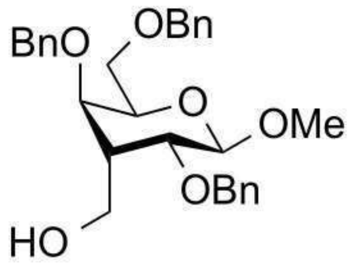

14a

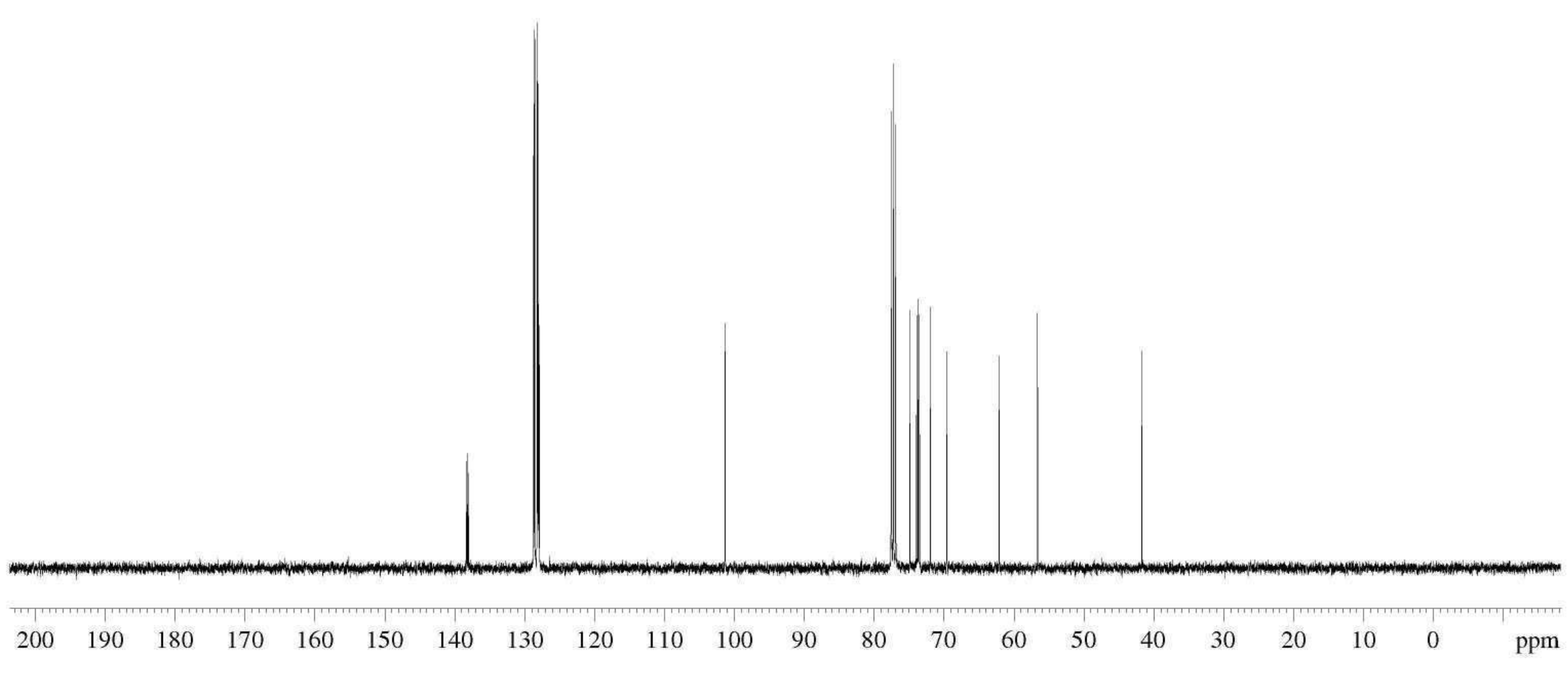
138.22
138.12
137.98
128.62
128.52
128.47
128.15
128.03
127.96
127.90
127.81
101.24
77.48
77.19
77.16
76.84
74.78
73.83
73.64
73.44
71.85
69.53
62.02
56.52
41.62
200
190
180
170
160
150
140
130
120
110
100
90
80
70
60
50
40
30
20
10
0
ppm

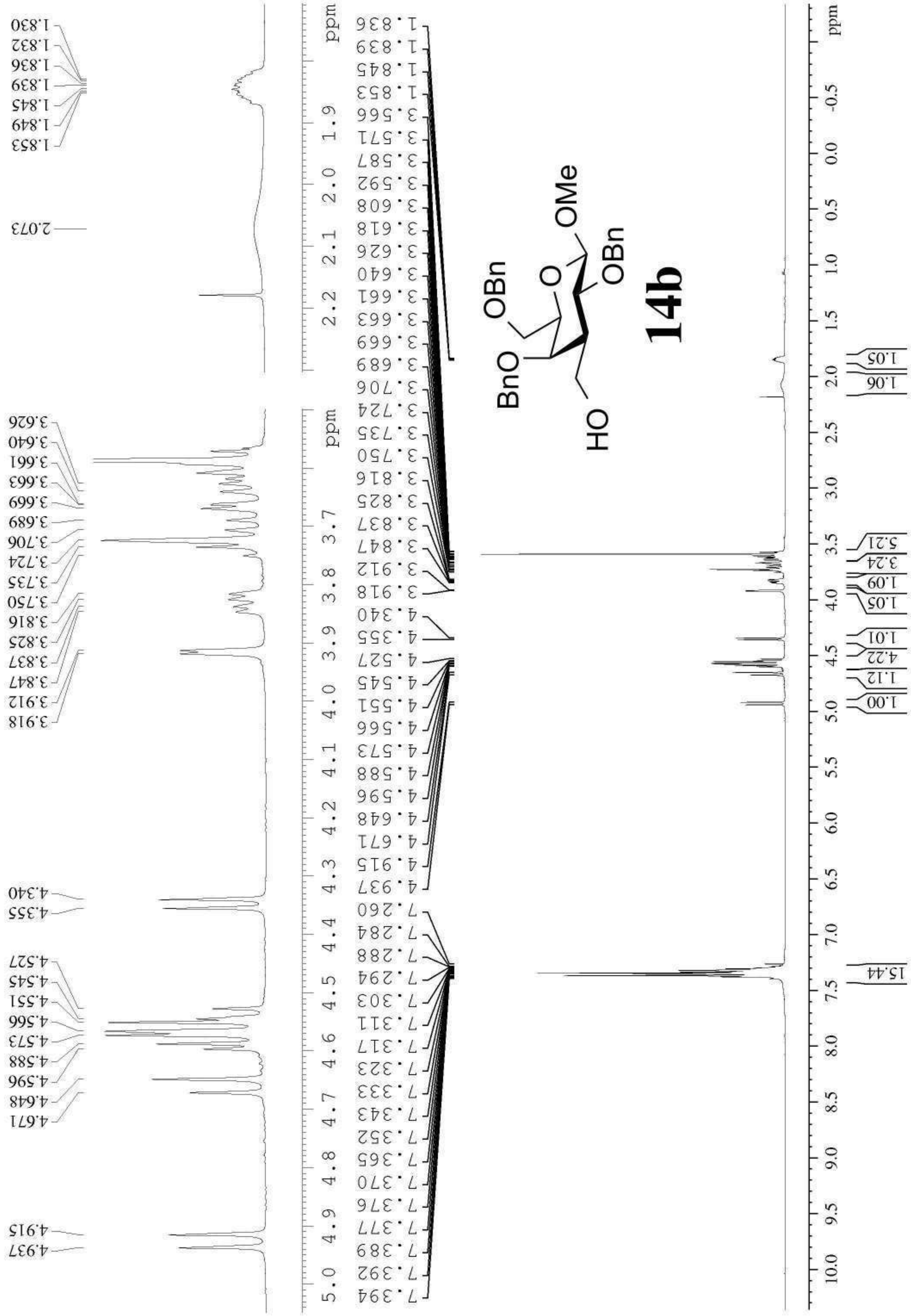
BnO
OBn
O
OMe
OBn
HO
14b
ppm
1.836
1.839
1.845
1.853
3.566
3.571
3.587
3.592
3.608
3.618
3.626
3.640
3.661
3.663
3.669
3.689
3.706
3.724
3.735
3.750
3.816
3.825
3.837
3.847
3.912
3.918
4.340
4.355
4.527
4.545
4.551
4.566
4.573
4.588
4.596
4.648
4.671
4.915
4.937
7.260
7.284
7.288
7.294
7.303
7.311
7.317
7.323
7.333
7.343
7.352
7.365
7.370
7.376
7.377
7.389
7.392
7.394
1.05
1.06
5.21
3.24
1.09
1.05
1.01
4.22
1.12
1.00
15.44
2.073

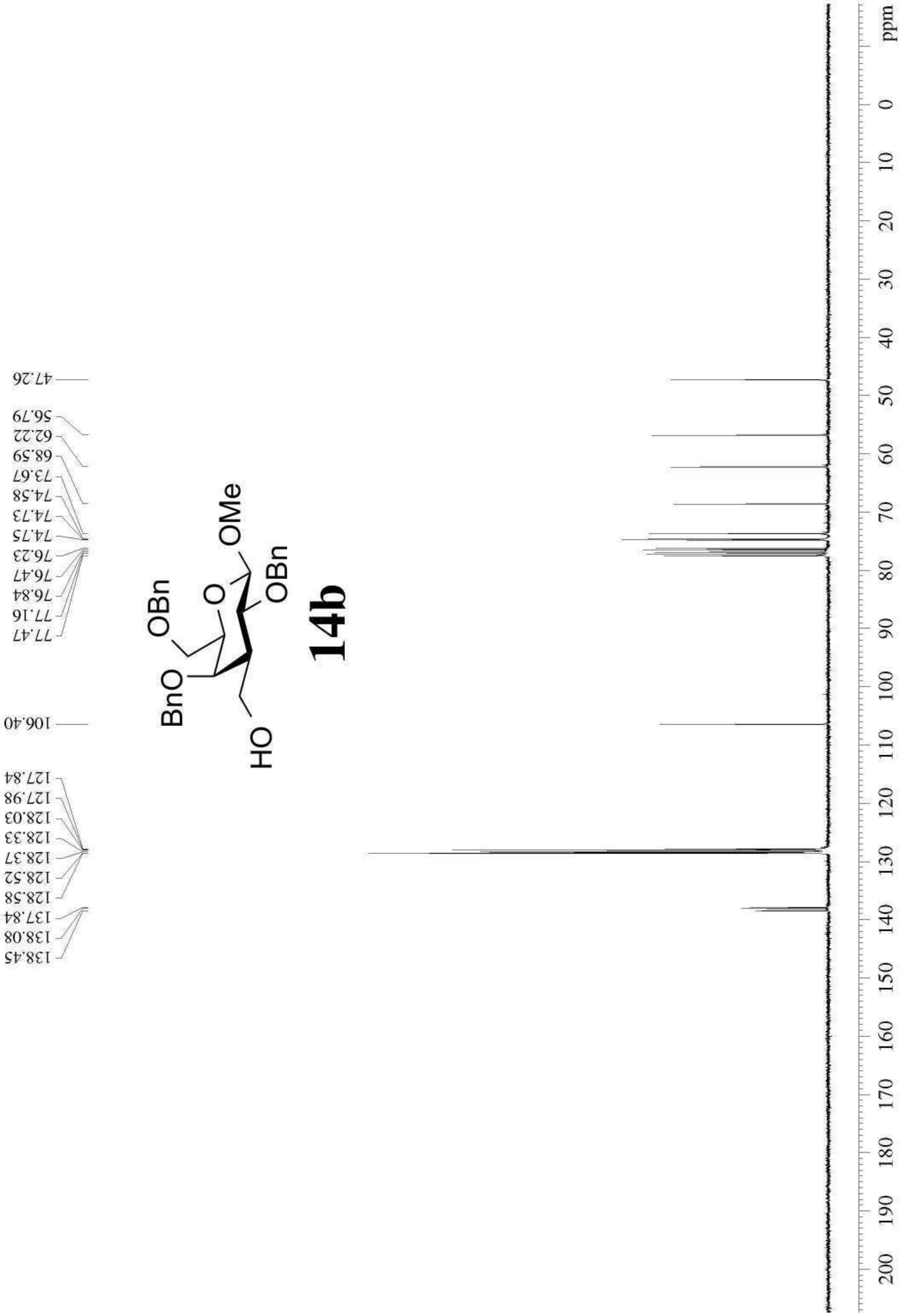
47.26
56.79
62.22
68.59
73.67
74.58
74.73
74.75
76.23
76.47
76.84
77.16
77.47
106.40
127.84
127.98
128.03
128.33
128.37
128.52
128.58
137.84
138.08
138.45
BnO
OBn
O
OMe
OBn
HO
14b
ppm
0
10
20
30
40
50
60
70
80
90
100
110
120
130
140
150
160
170
180
190
200

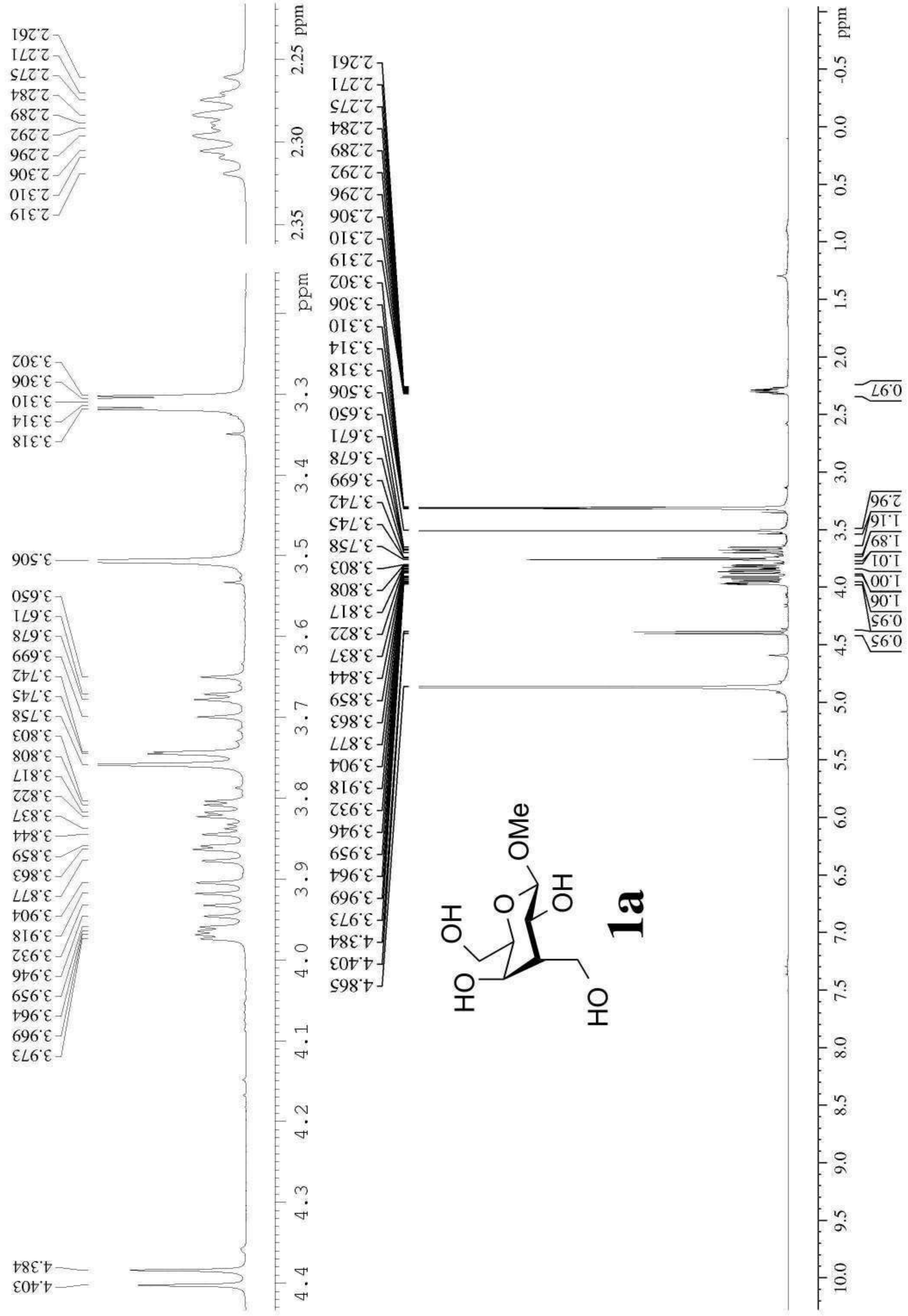
1a

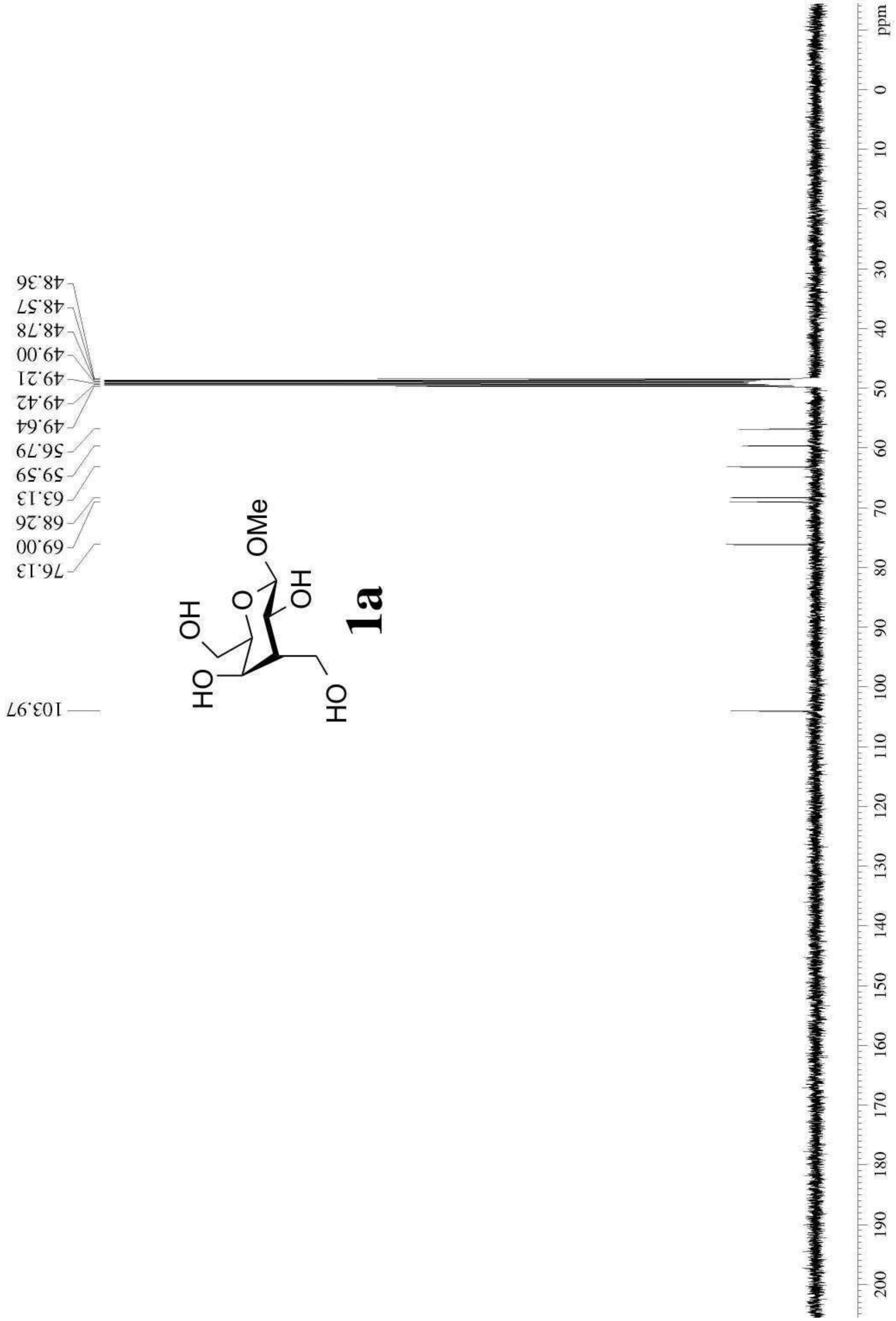
48.36
48.57
48.78
49.00
49.21
49.42
49.64
56.79
59.59
63.13
68.26
69.00
76.13
103.97
OMe
OH
HO
OH
HO
1a
ppm

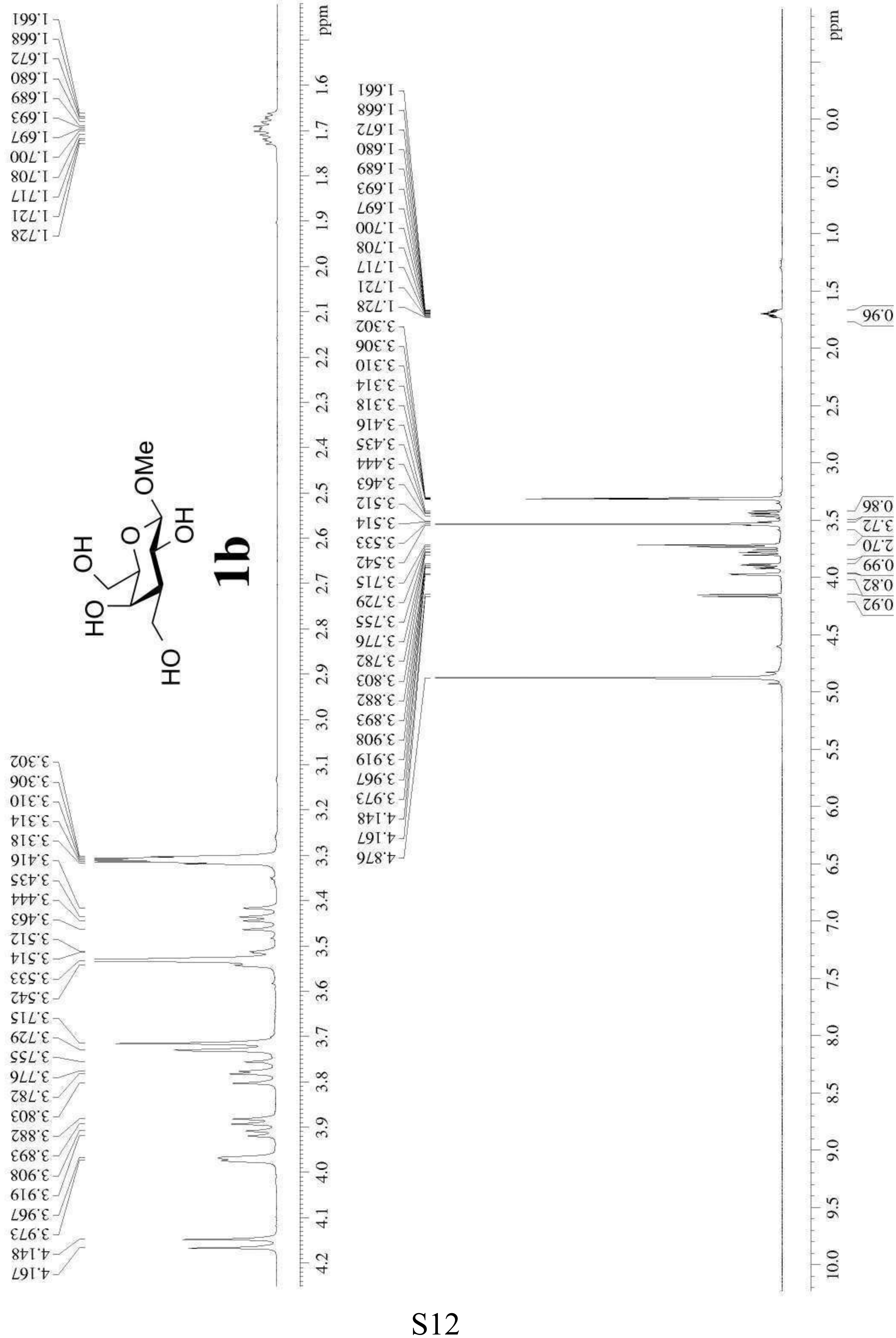

1.661
1.668
1.672
1.680
1.689
1.693
1.697
1.700
1.708
1.717
1.721
1.728
3.302
3.306
3.310
3.314
3.318
3.416
3.435
3.444
3.463
3.512
3.514
3.533
3.542
3.715
3.729
3.755
3.776
3.782
3.803
3.882
3.893
3.908
3.919
3.967
3.973
4.148
4.167
4.876
OMe
OH
HO
1b
ppm
0.96
0.86
3.72
2.70
0.99
0.82
0.92

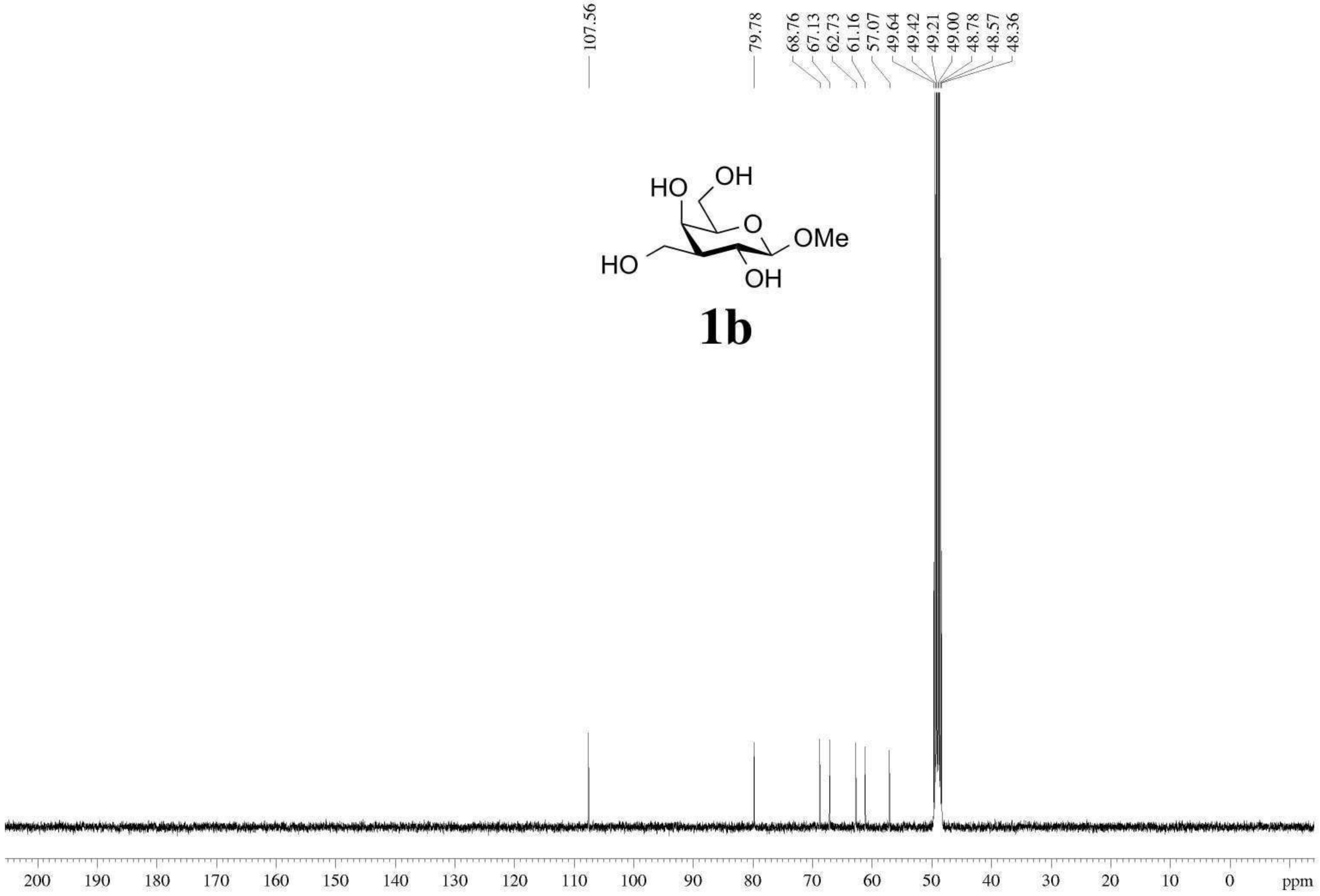
107.56
79.78
68.76
67.13
62.73
61.16
57.07
49.64
49.42
49.21
49.00
48.78
48.57
48.36
HO
OH
O
OMe
HO
OH
1b
200
190
180
170
160
150
140
130
120
110
100
90
80
70
60
50
40
30
20
10
0
ppm

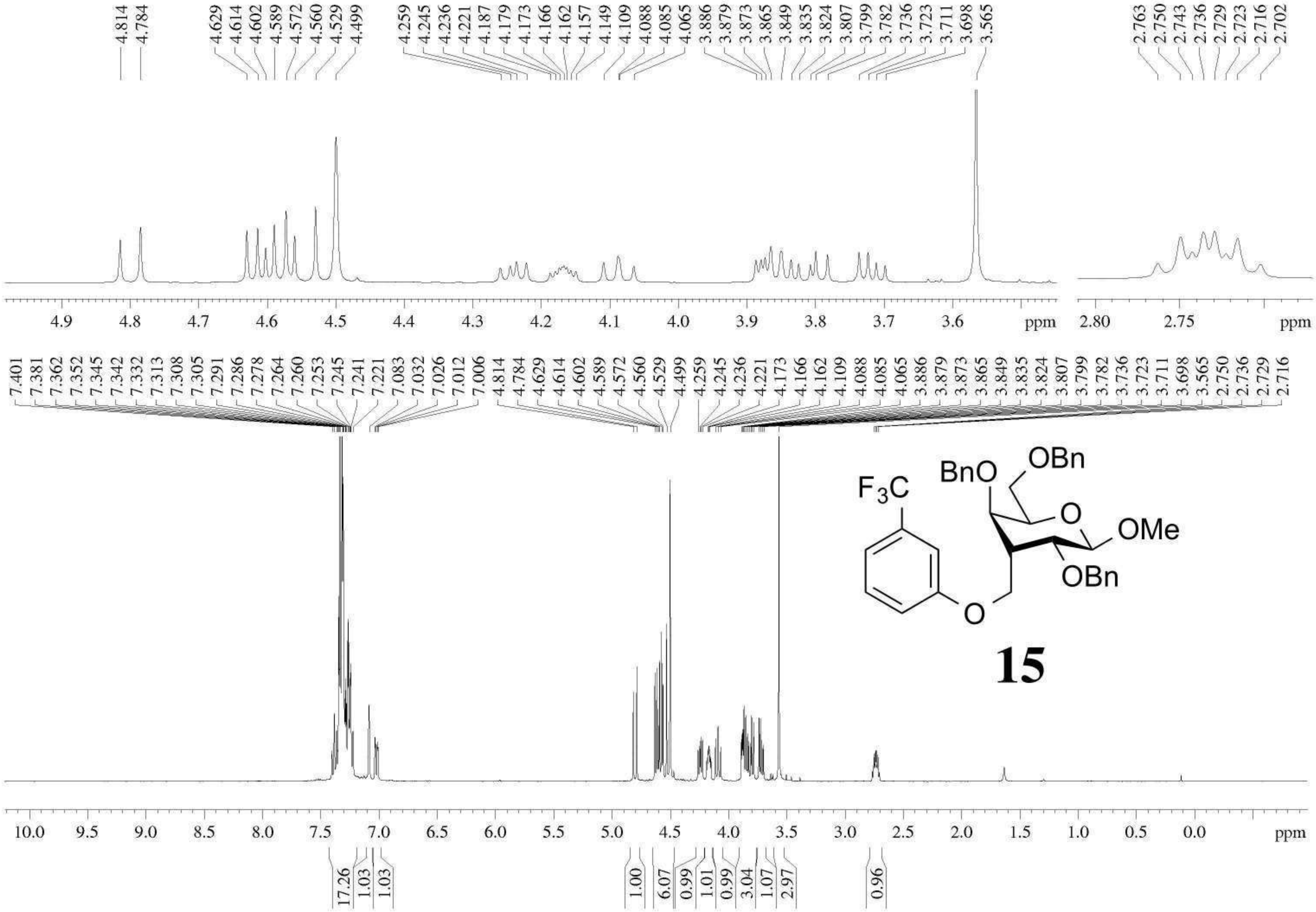
F3C
BnO
OBn
OMe
OBn
15
ppm

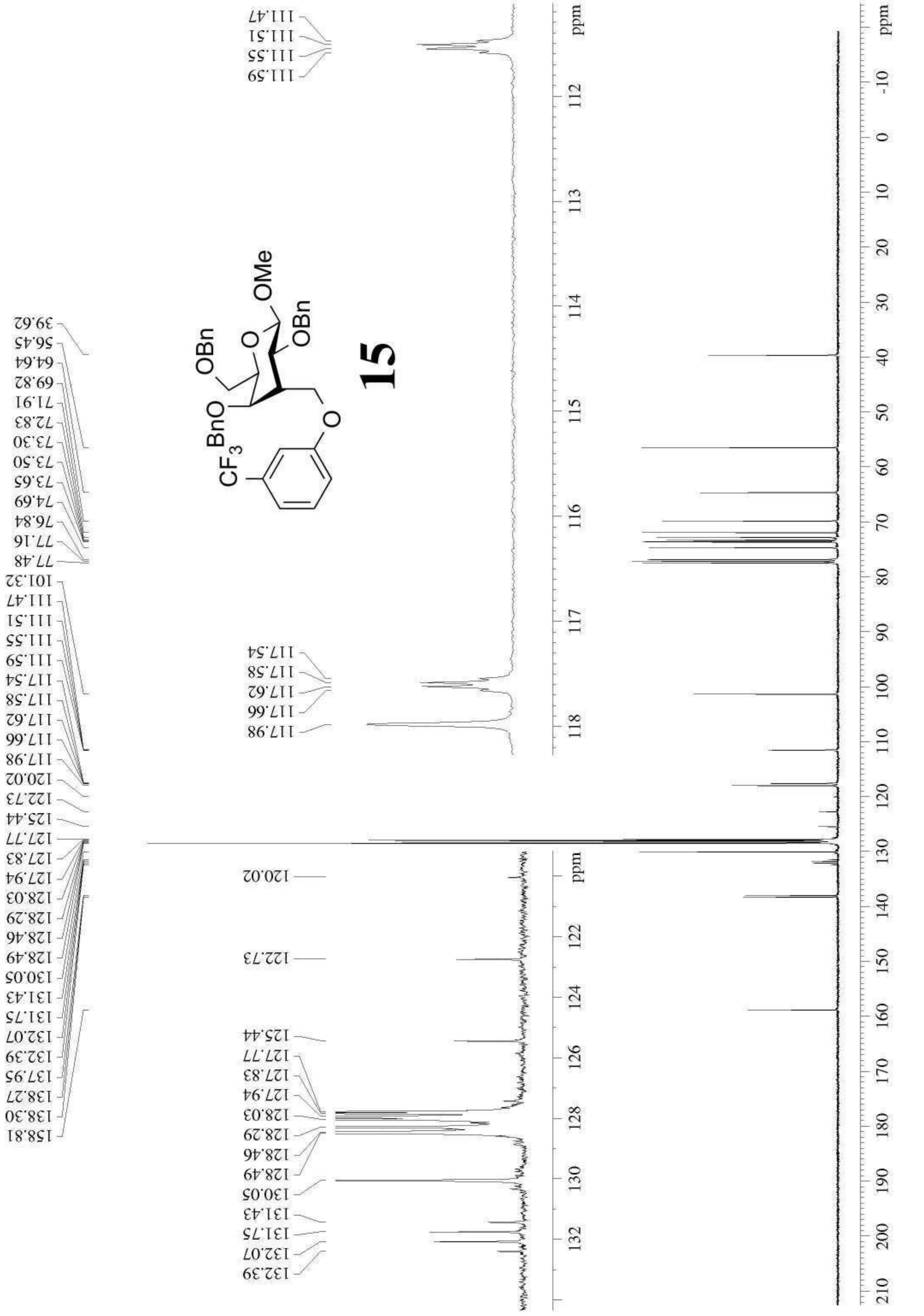

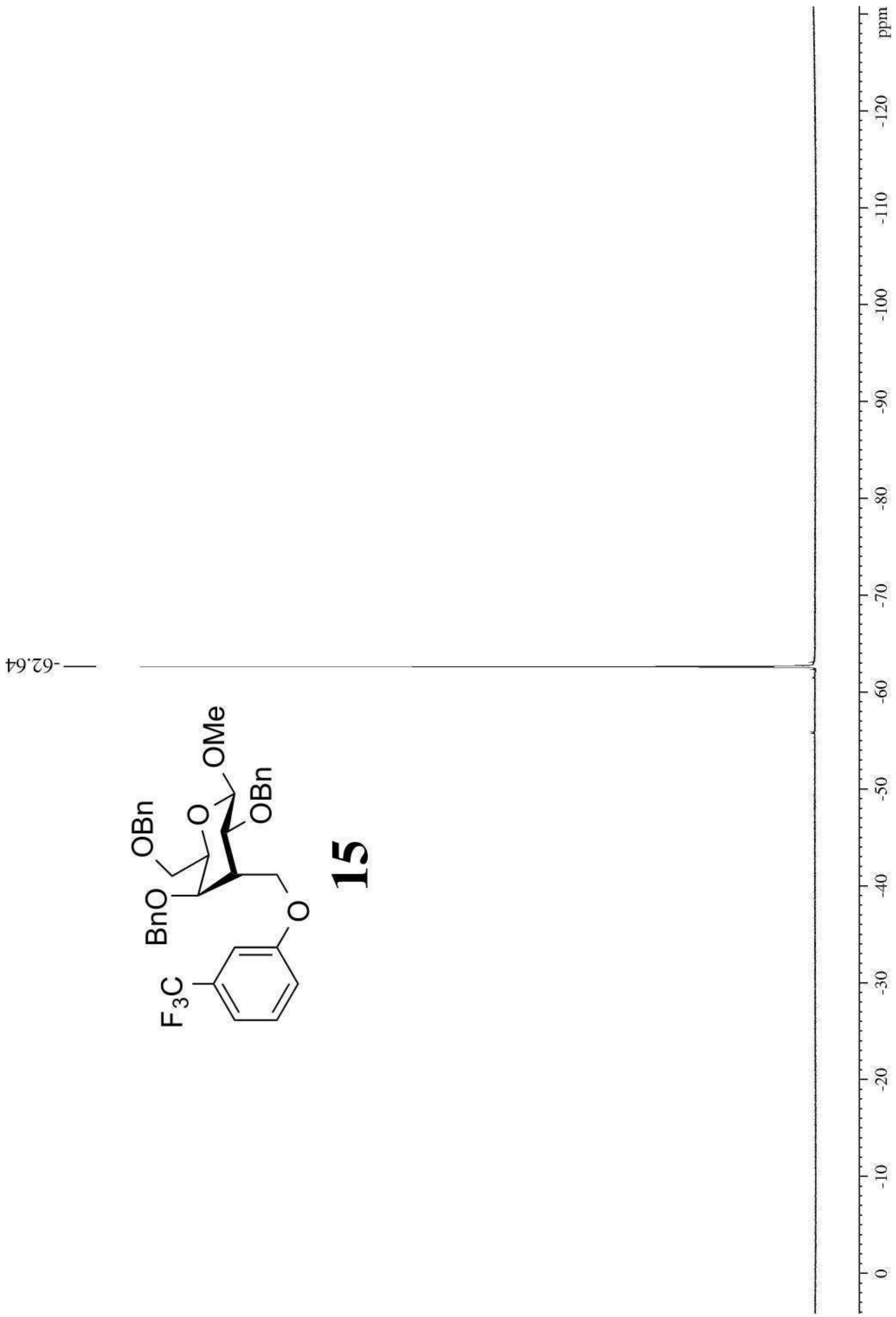

-62.64
15
F3C
BnO
OBn
OMe
ppm

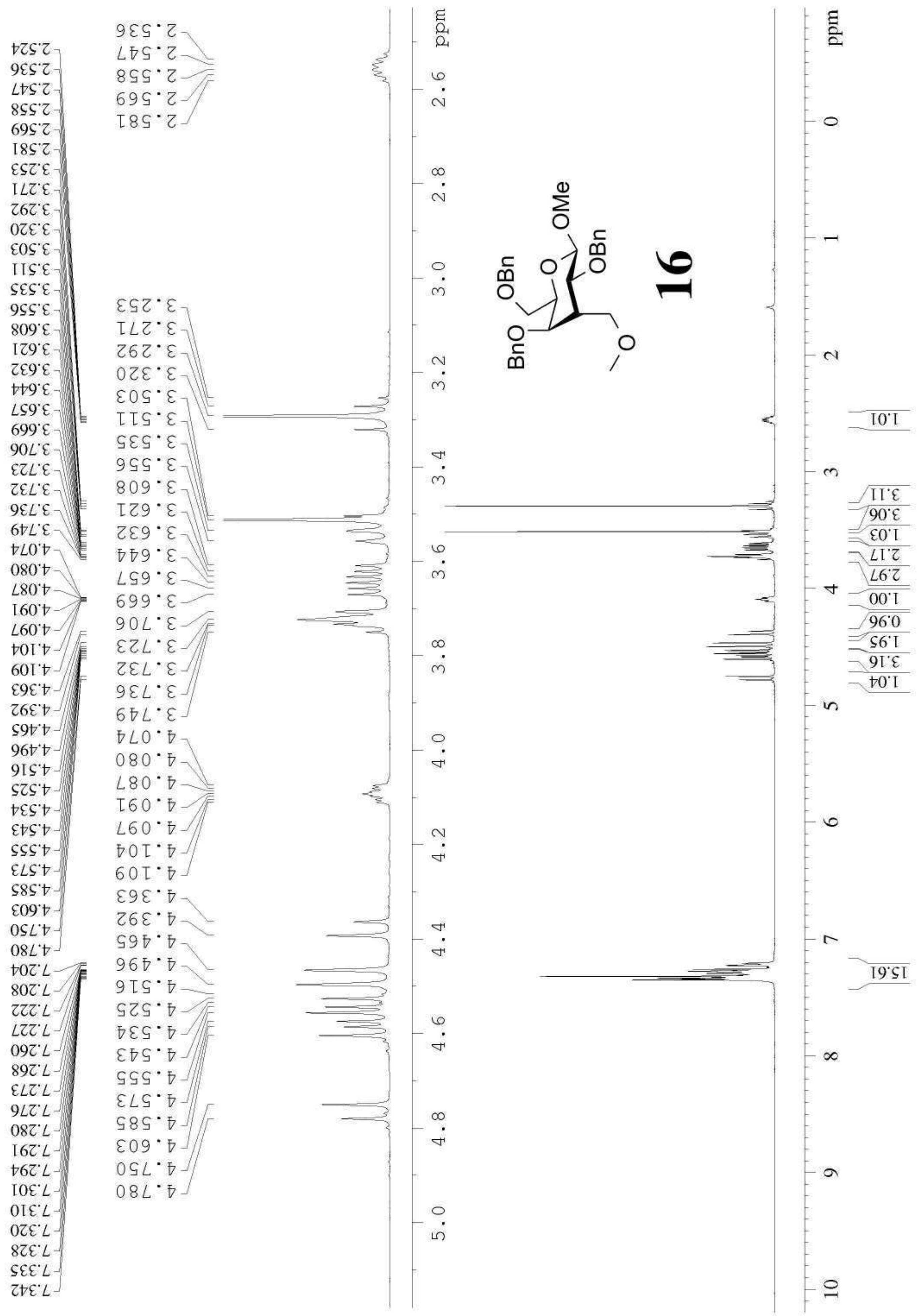

16
BnO
OBn
OMe
OBn
ppm
2.524
2.536
2.547
2.558
2.569
2.581
3.253
3.271
3.292
3.320
3.503
3.511
3.535
3.556
3.608
3.621
3.632
3.644
3.657
3.669
3.706
3.723
3.732
3.736
3.749
4.074
4.080
4.087
4.091
4.097
4.104
4.109
4.363
4.392
4.465
4.496
4.516
4.525
4.534
4.543
4.555
4.573
4.585
4.603
4.750
4.780
7.204
7.208
7.222
7.227
7.260
7.268
7.273
7.276
7.280
7.291
7.294
7.301
7.310
7.320
7.328
7.335
7.342
1.01
3.11
3.06
1.03
2.17
2.97
1.00
0.96
1.95
3.16
1.04
15.61

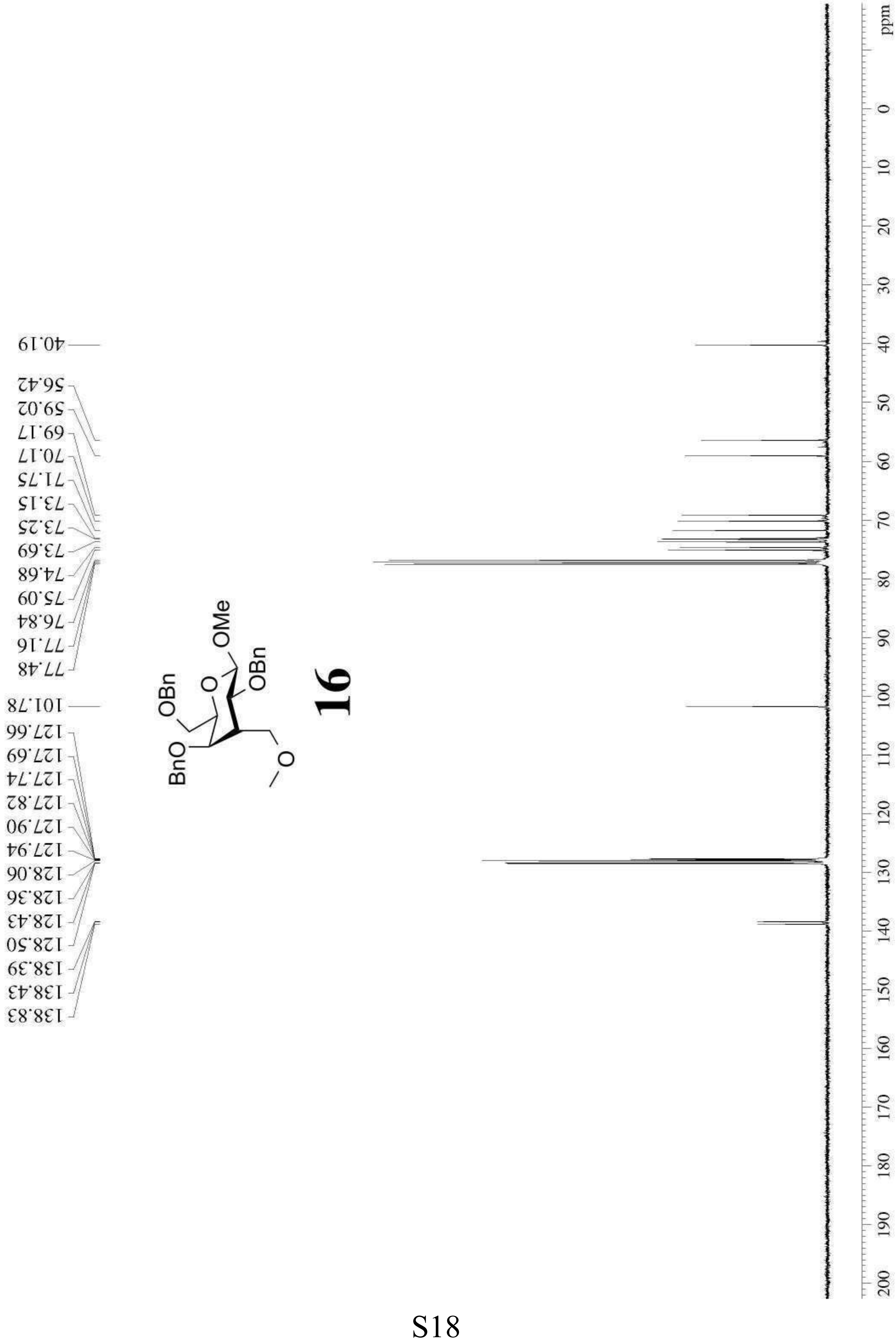
40.19
56.42
59.02
69.17
70.17
71.75
73.15
73.25
73.69
74.68
75.09
76.84
77.16
77.48
101.78
127.66
127.69
127.74
127.82
127.90
127.94
128.06
128.36
128.43
128.50
138.39
138.43
138.83
BnO
OBn
O
OMe
OBn
O
16
ppm

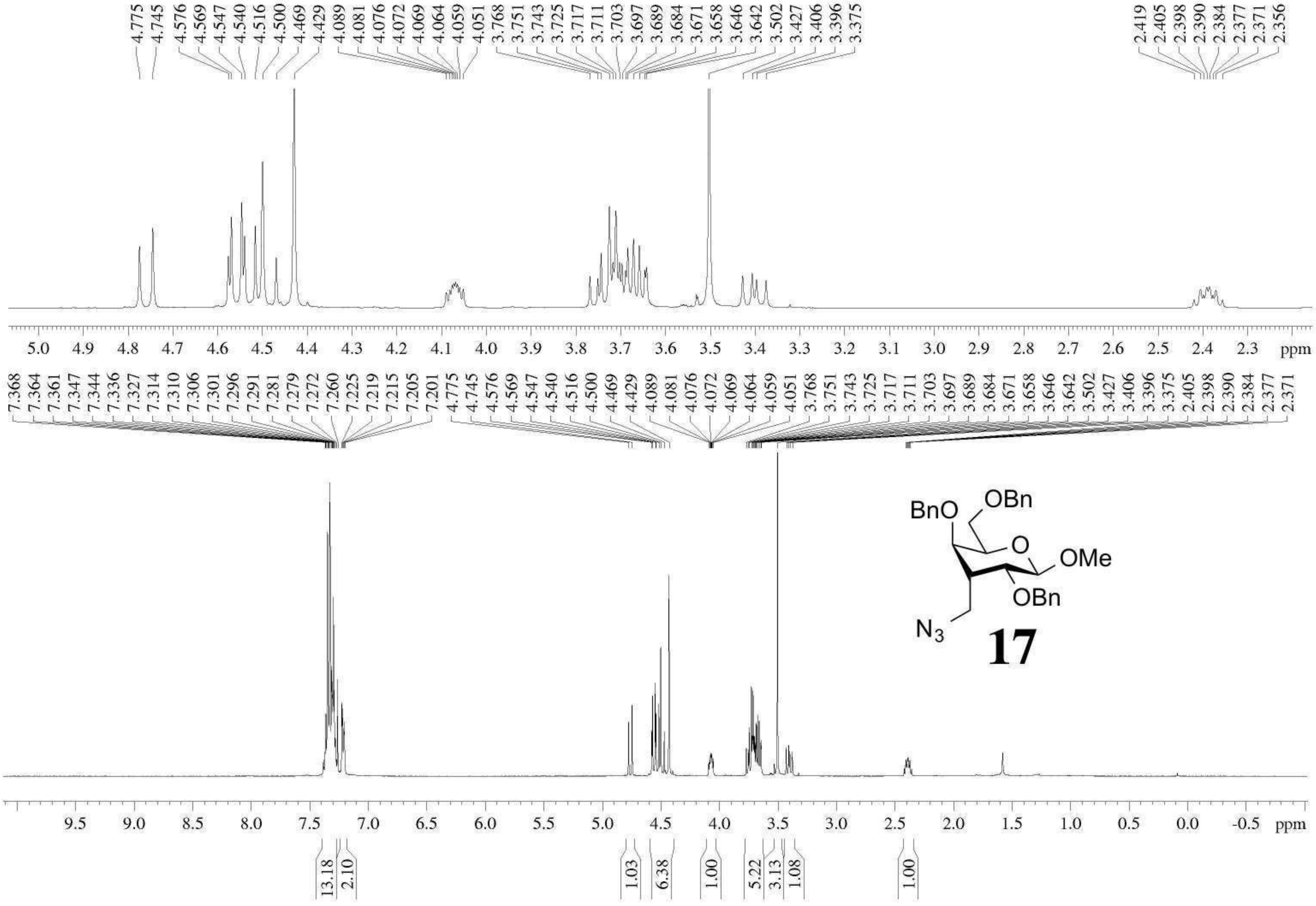
4.775 4.745 4.576 4.569 4.547 4.540 4.516 4.500 4.469 4.429 4.089 4.081 4.076 4.072 4.069 4.064 4.059 4.051 3.768 3.751 3.743 3.725 3.717 3.711 3.703 3.697 3.689 3.684 3.671 3.658 3.646 3.642 3.502 3.427 3.406 3.396 3.375
2.419 2.405 2.398 2.390 2.384 2.377 2.371 2.356
5.0 4.9 4.8 4.7 4.6 4.5 4.4 4.3 4.2 4.1 4.0 3.9 3.8 3.7 3.6 3.5 3.4 3.3 3.2 3.1 3.0 2.9 2.8 2.7 2.6 2.5 2.4 2.3 ppm
7.368 7.364 7.361 7.347 7.344 7.336 7.327 7.314 7.310 7.306 7.301 7.296 7.291 7.281 7.279 7.272 7.260 7.225 7.219 7.215 7.205 7.201 4.775 4.745 4.576 4.569 4.547 4.540 4.516 4.500 4.469 4.429 4.089 4.081 4.076 4.072 4.069 4.064 4.059 4.051 3.768 3.751 3.743 3.725 3.717 3.711 3.703 3.697 3.689 3.684 3.671 3.658 3.646 3.642 3.502 3.427 3.406 3.396 3.375 2.405 2.398 2.390 2.384 2.377 2.371
BnO
OBn
O
OMe
OBn
N3
17
9.5 9.0 8.5 8.0 7.5 7.0 6.5 6.0 5.5 5.0 4.5 4.0 3.5 3.0 2.5 2.0 1.5 1.0 0.5 0.0 -0.5 ppm
13.18 2.10 1.03 6.38 1.00 5.22 3.13 1.08 1.00

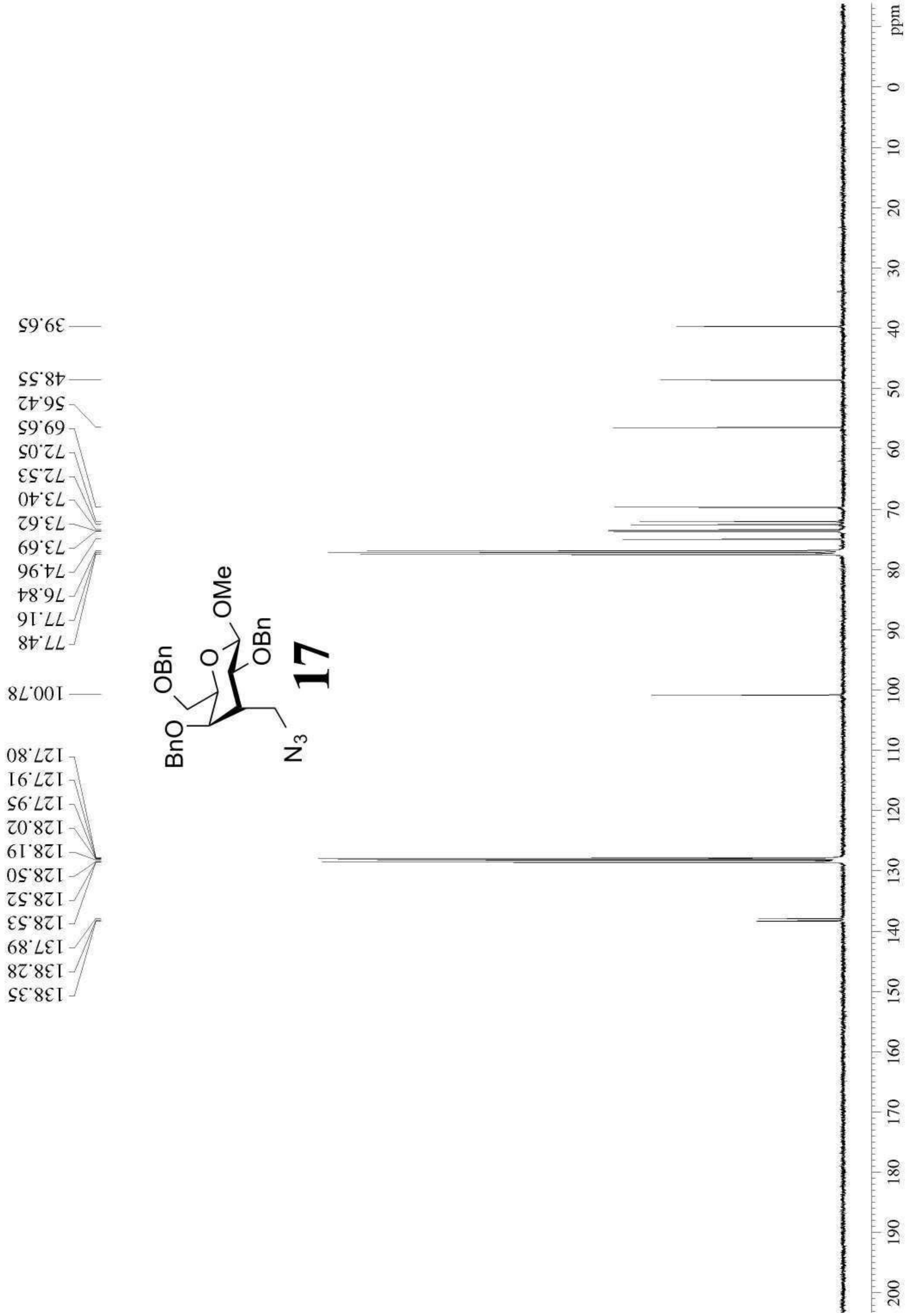

39.65
48.55
56.42
69.65
72.05
72.53
73.40
73.62
73.69
74.96
76.84
77.16
77.48
100.78
127.80
127.91
127.95
128.02
128.19
128.50
128.52
128.53
137.89
138.28
138.35
BnO
OBn
O
OMe
OBn
N_3
17
ppm
0
10
20
30
40
50
60
70
80
90
100
110
120
130
140
150
160
170
180
190
200

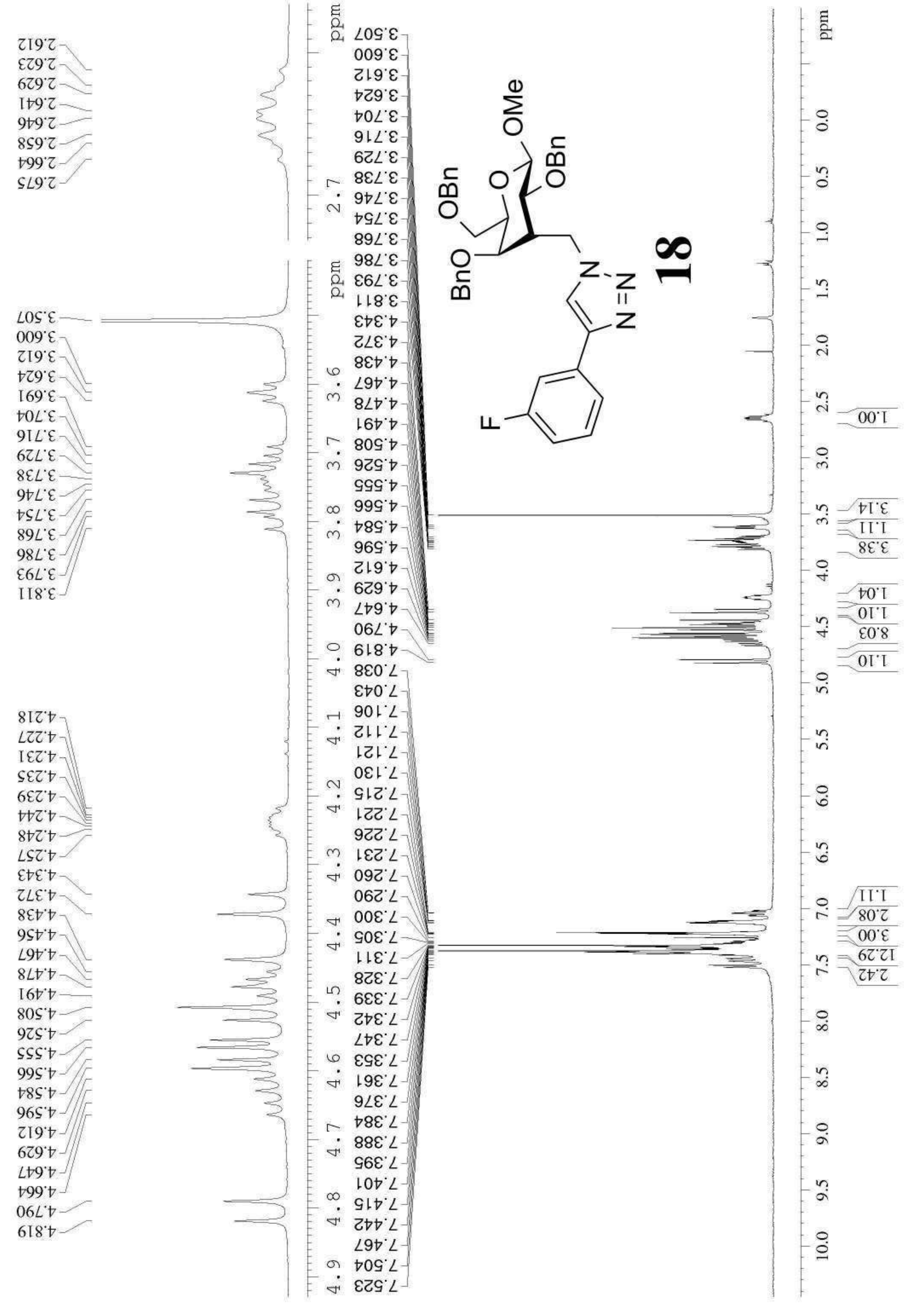
18

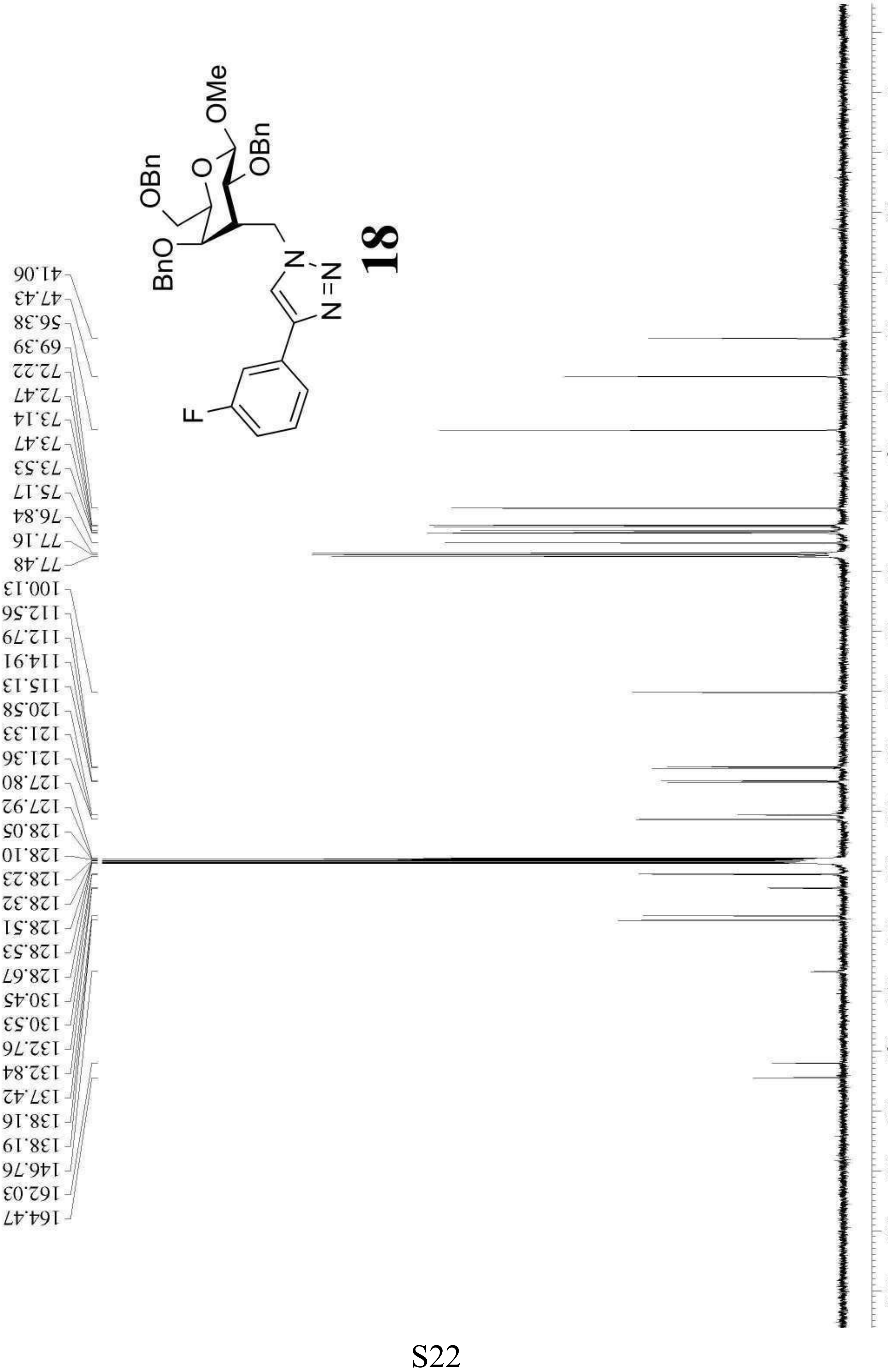
41.06
47.43
56.38
69.39
72.22
72.47
73.14
73.47
73.53
75.17
76.84
77.16
77.48
100.13
112.56
112.79
114.91
115.13
120.58
121.33
121.36
127.80
127.92
128.05
128.10
128.23
128.32
128.51
128.53
128.67
130.45
130.53
132.76
132.84
137.42
138.16
138.19
146.76
162.03
164.47
BnO
OBn
O
OMe
OBn
N
N=N
F
18
ppm

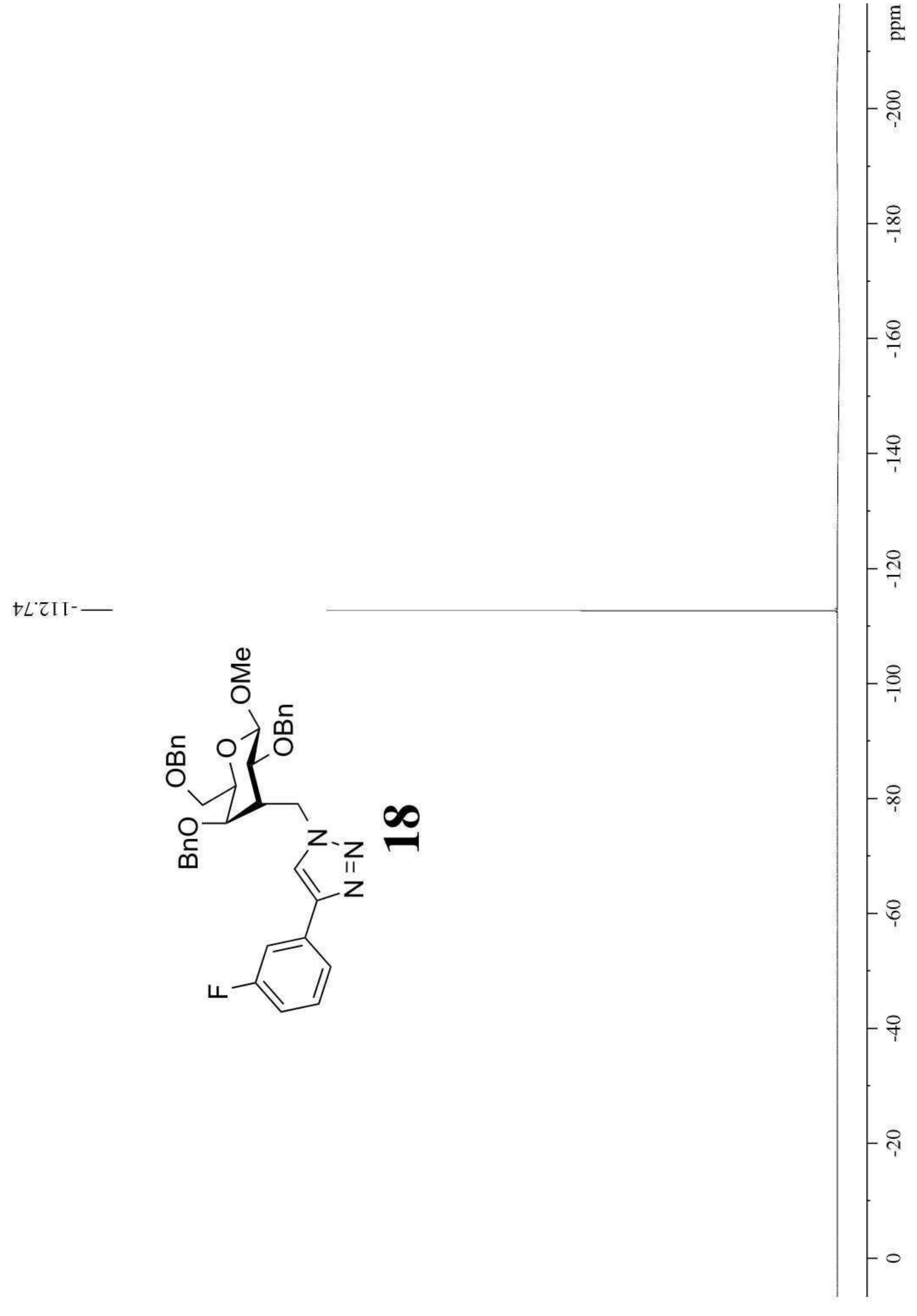
-112.74
18
ppm

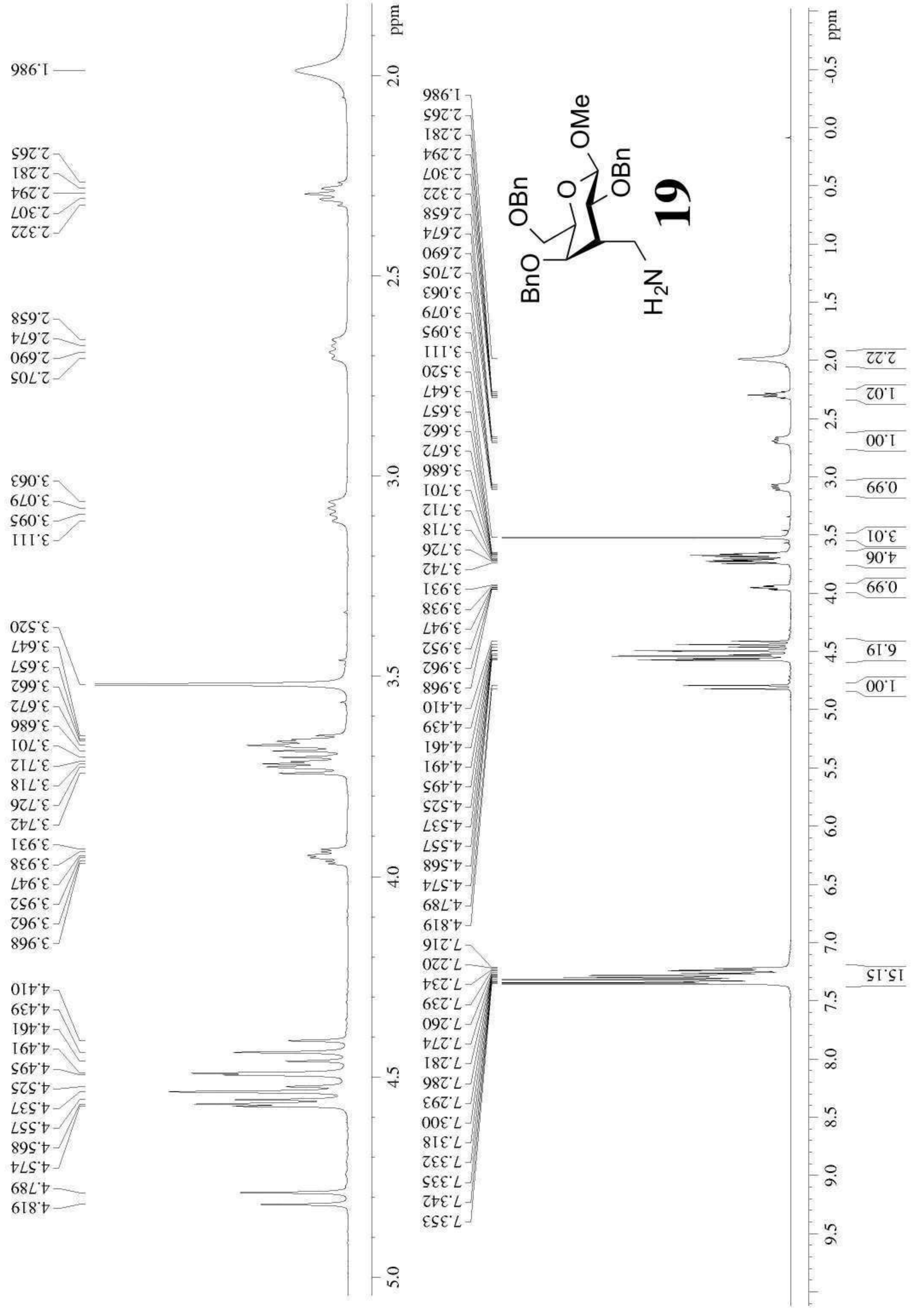
BnO
OBn
OMe
OBn
H2N
19
ppm

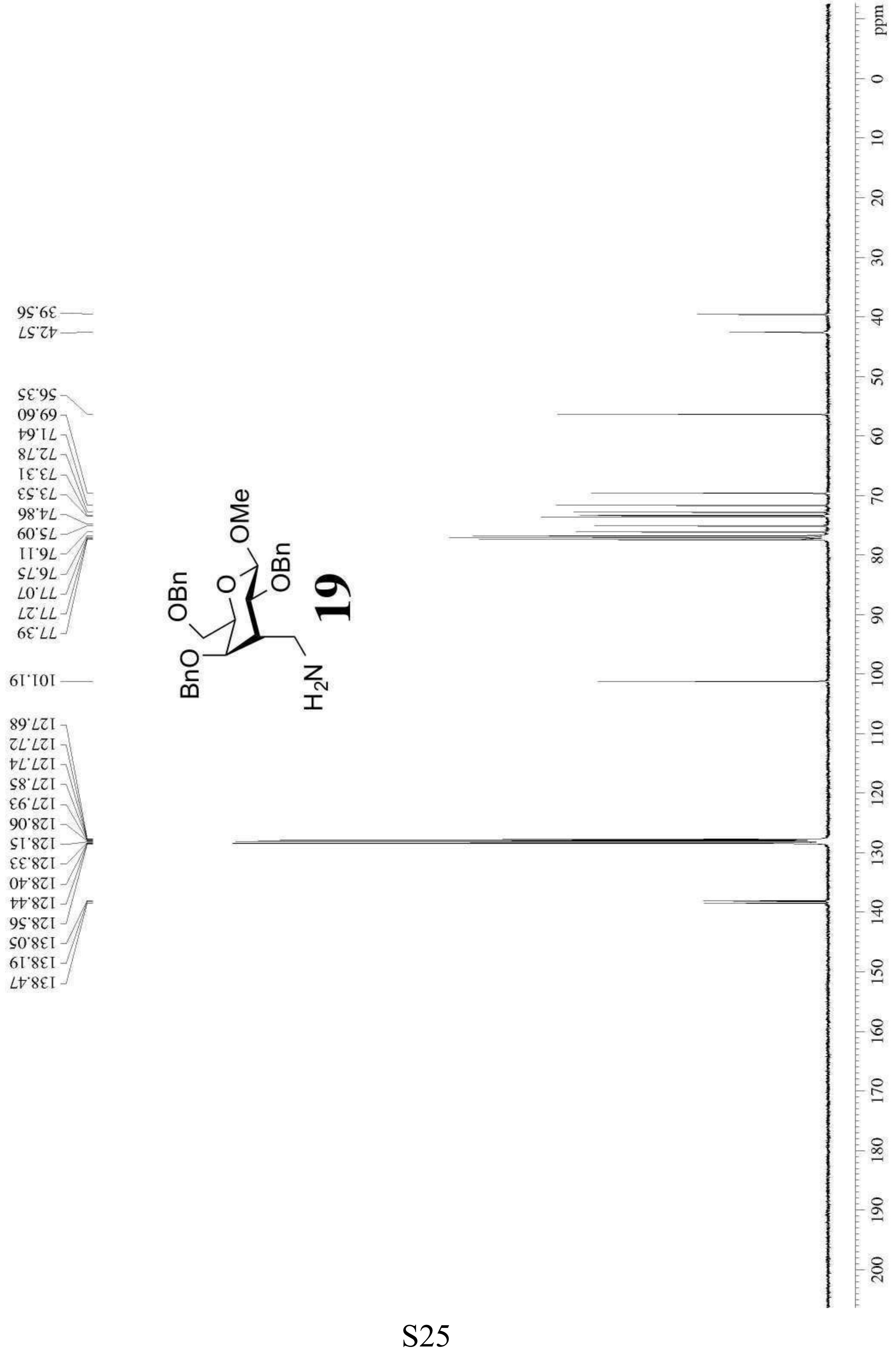

39.56
42.57
56.35
69.60
71.64
72.78
73.31
73.53
74.86
75.09
76.11
76.75
77.07
77.27
77.39
101.19
127.68
127.72
127.74
127.85
127.93
128.06
128.15
128.33
128.40
128.44
128.56
138.05
138.19
138.47
OBn
BnO
O
OMe
OBn
H2N
19
ppm

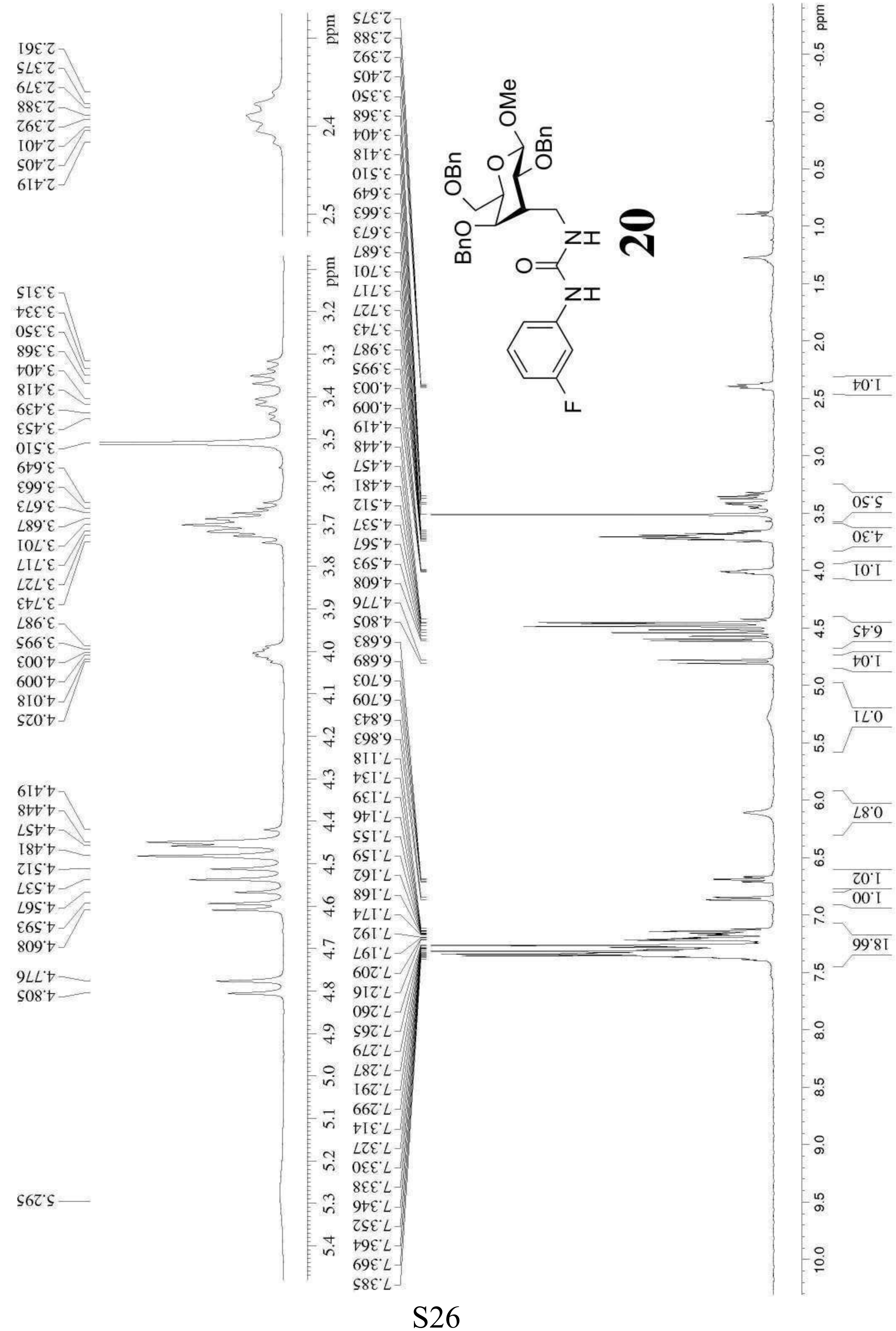

20

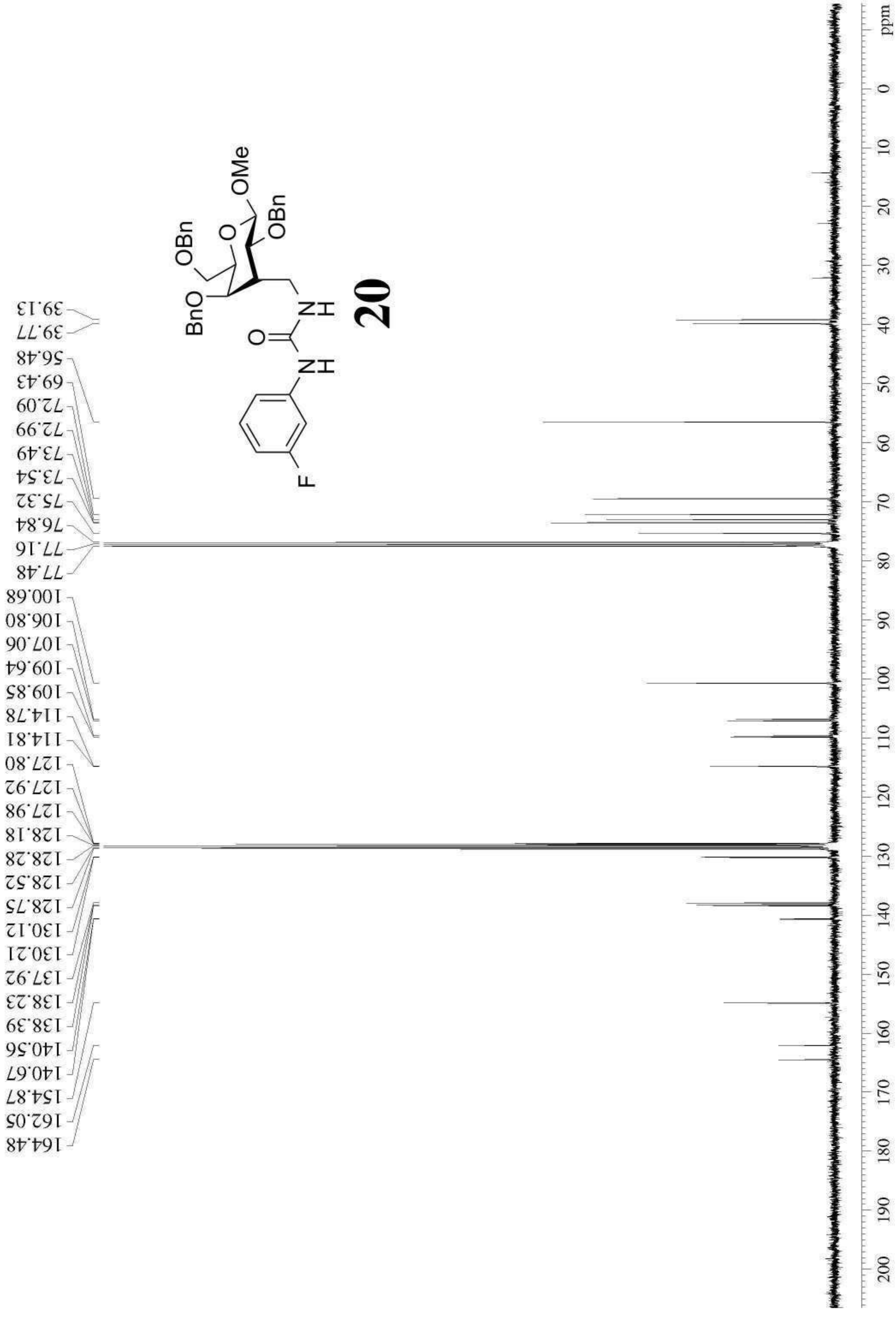
39.13
39.77
56.48
69.43
72.09
72.99
73.49
73.54
75.32
76.84
77.16
77.48
100.68
106.80
107.06
109.64
109.85
114.78
114.81
127.80
127.92
127.98
128.18
128.28
128.52
128.75
130.12
130.21
137.92
138.23
138.39
140.56
140.67
154.87
162.05
164.48
BnO
OBn
O
OMe
OBn
O
N
H
N
H
F
20
ppm
200
190
180
170
160
150
140
130
120
110
100
90
80
70
60
50
40
30
20
10
0

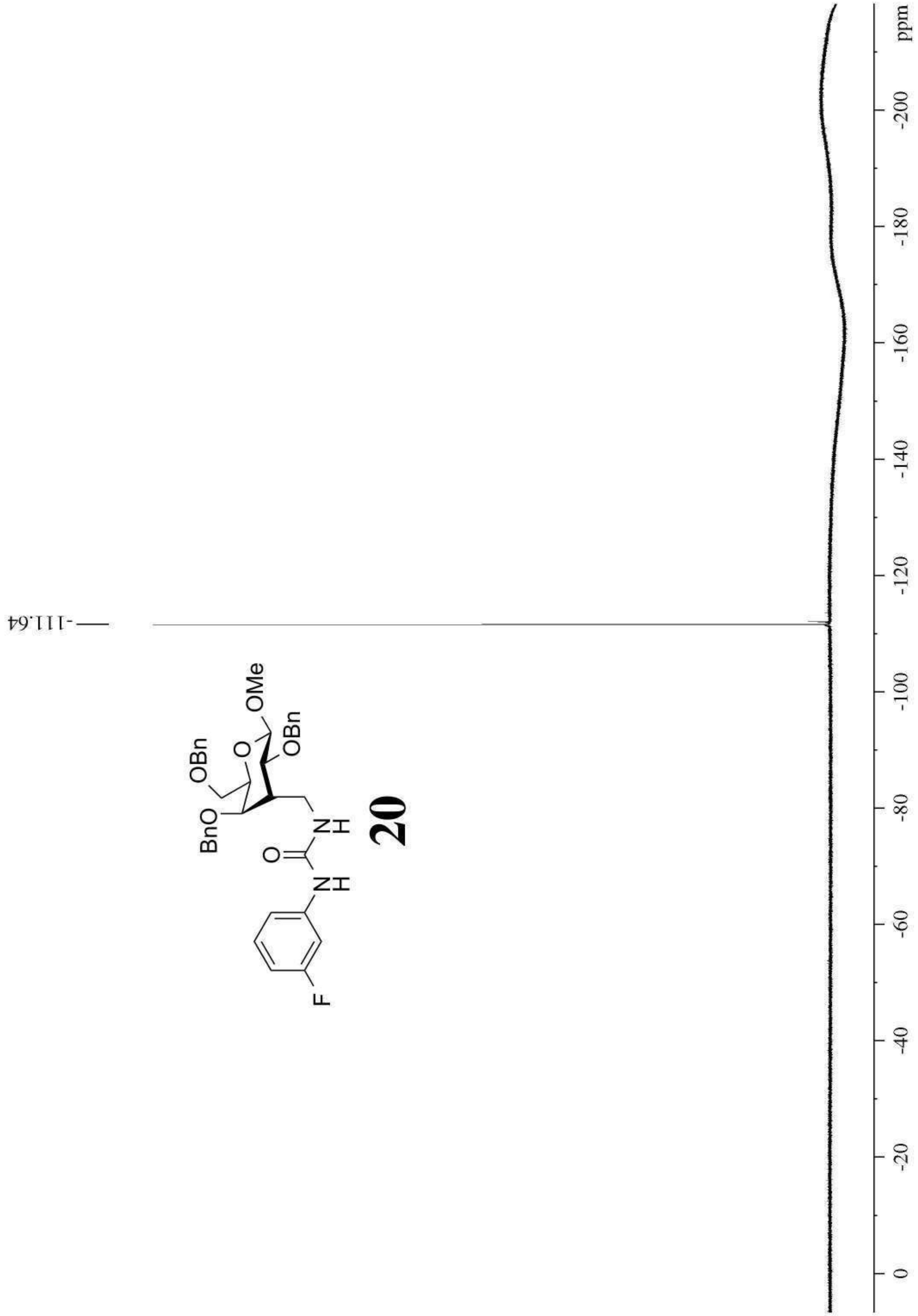
-111.64
BnO
OBn
O
OMe
OBn
O
N
H
N
H
F
20
ppm
-200
-180
-160
-140
-120
-100
-80
-60
-40
-20
0

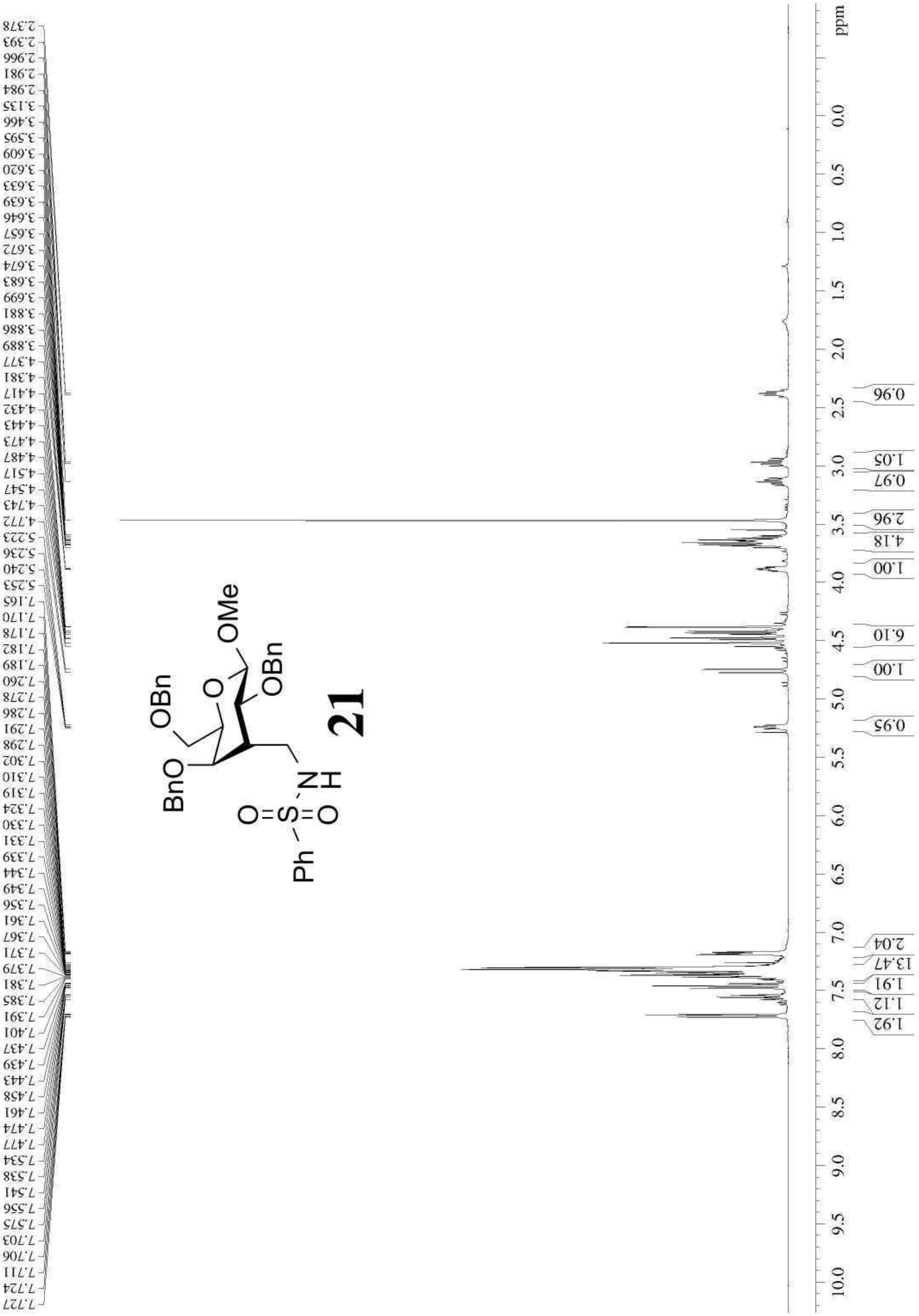
2.378
2.393
2.966
2.981
2.984
3.135
3.466
3.595
3.609
3.620
3.633
3.639
3.646
3.657
3.672
3.674
3.683
3.699
3.881
3.886
3.889
4.377
4.381
4.417
4.432
4.443
4.473
4.487
4.517
4.547
4.743
4.772
5.223
5.236
5.240
5.253
7.165
7.170
7.178
7.182
7.189
7.260
7.278
7.286
7.291
7.298
7.302
7.310
7.319
7.324
7.330
7.331
7.339
7.344
7.349
7.356
7.361
7.367
7.371
7.379
7.381
7.385
7.391
7.401
7.437
7.439
7.443
7.458
7.461
7.474
7.477
7.534
7.538
7.541
7.556
7.575
7.703
7.706
7.711
7.724
7.727
0.96
1.05
0.97
2.96
4.18
1.00
6.10
1.00
0.95
2.04
13.47
1.91
1.12
1.92
ppm
0.0
0.5
1.0
1.5
2.0
2.5
3.0
3.5
4.0
4.5
5.0
5.5
6.0
6.5
7.0
7.5
8.0
8.5
9.0
9.5
10.0
BnO
OBn
O
OMe
OBn
Ph
S
O
O
N
H
21

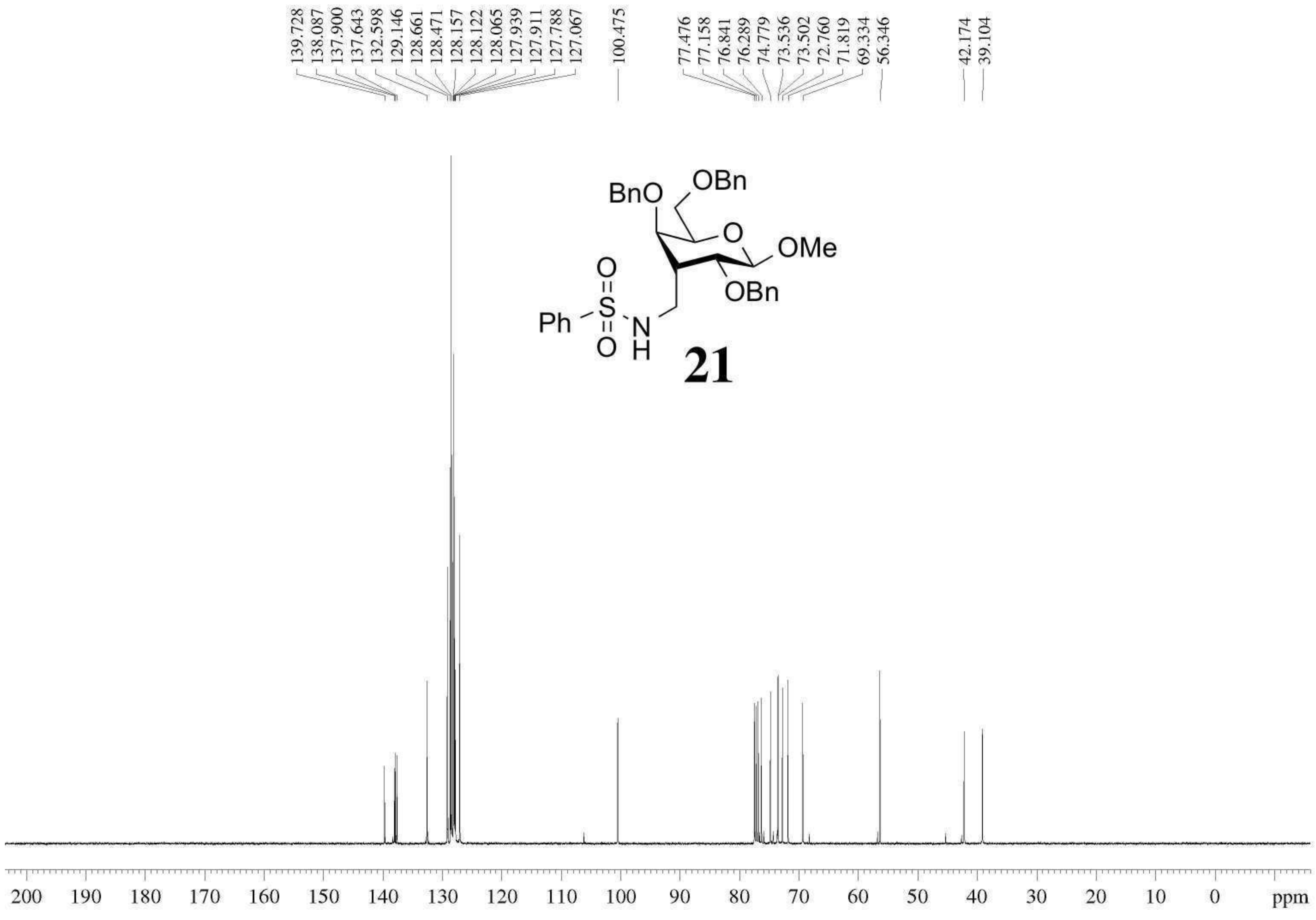
139.728
138.087
137.900
137.643
132.598
129.146
128.661
128.471
128.157
128.122
128.065
127.939
127.911
127.788
127.067
100.475
77.476
77.158
76.841
76.289
74.779
73.536
73.502
72.760
71.819
69.334
56.346
42.174
39.104
BnO
OBn
O
OMe
OBn
Ph
S
O
O
N
H
21
200
190
180
170
160
150
140
130
120
110
100
90
80
70
60
50
40
30
20
10
0
ppm

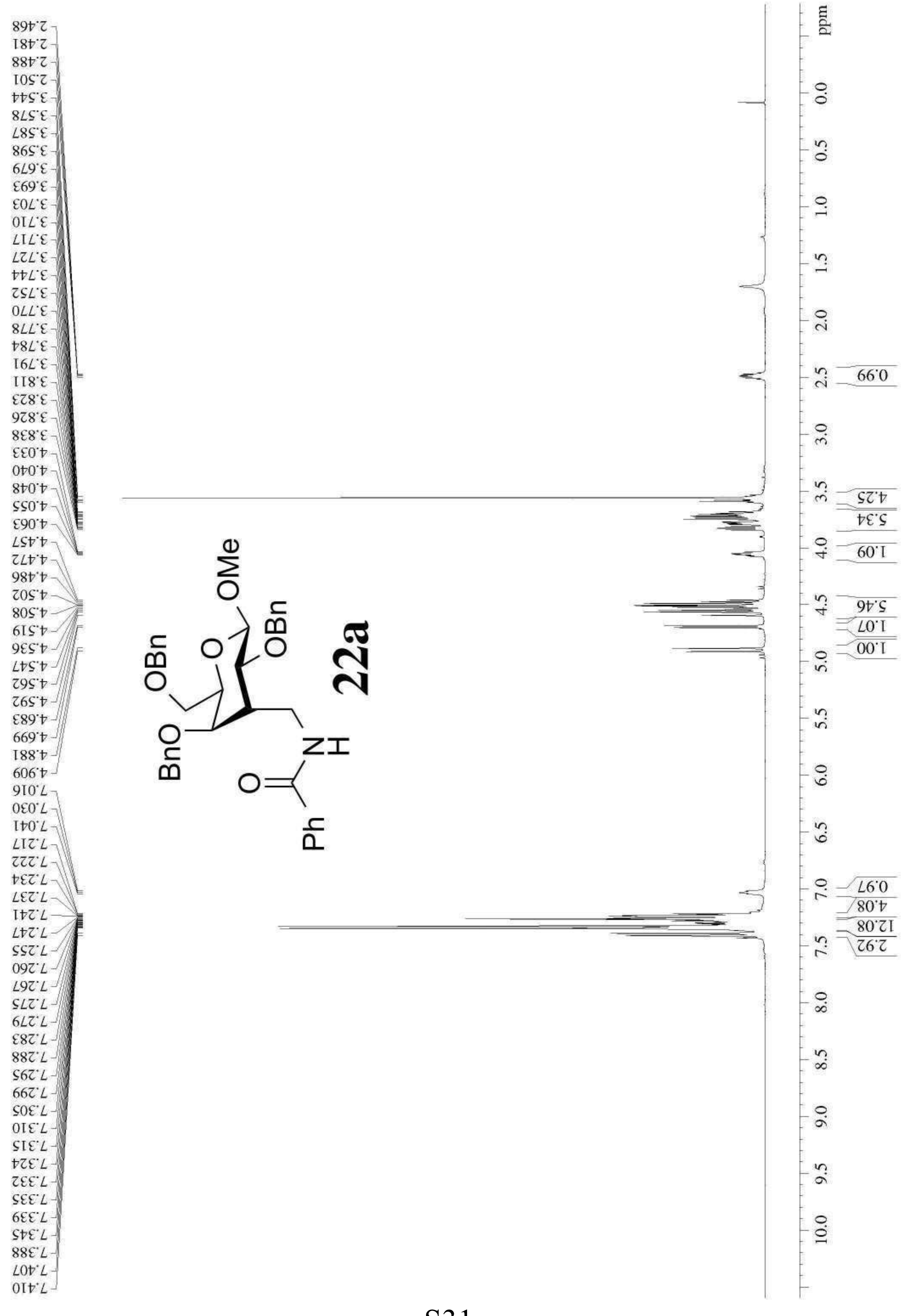
2.468
2.481
2.488
2.501
3.544
3.578
3.587
3.598
3.679
3.693
3.703
3.710
3.717
3.727
3.744
3.752
3.770
3.778
3.784
3.791
3.811
3.823
3.826
3.838
4.033
4.040
4.048
4.055
4.063
4.457
4.472
4.486
4.502
4.508
4.519
4.536
4.547
4.562
4.592
4.683
4.699
4.881
4.909
7.016
7.030
7.041
7.217
7.222
7.234
7.237
7.241
7.247
7.255
7.260
7.267
7.275
7.279
7.283
7.288
7.295
7.299
7.305
7.310
7.315
7.324
7.332
7.335
7.339
7.345
7.388
7.407
7.410
BnO
OBn
O
OMe
OBn
N
H
O
Ph
22a
0.99
4.25
5.34
1.09
5.46
1.07
1.00
0.97
4.08
12.08
2.92
0.0
0.5
1.0
1.5
2.0
2.5
3.0
3.5
4.0
4.5
5.0
5.5
6.0
6.5
7.0
7.5
8.0
8.5
9.0
9.5
10.0
ppm

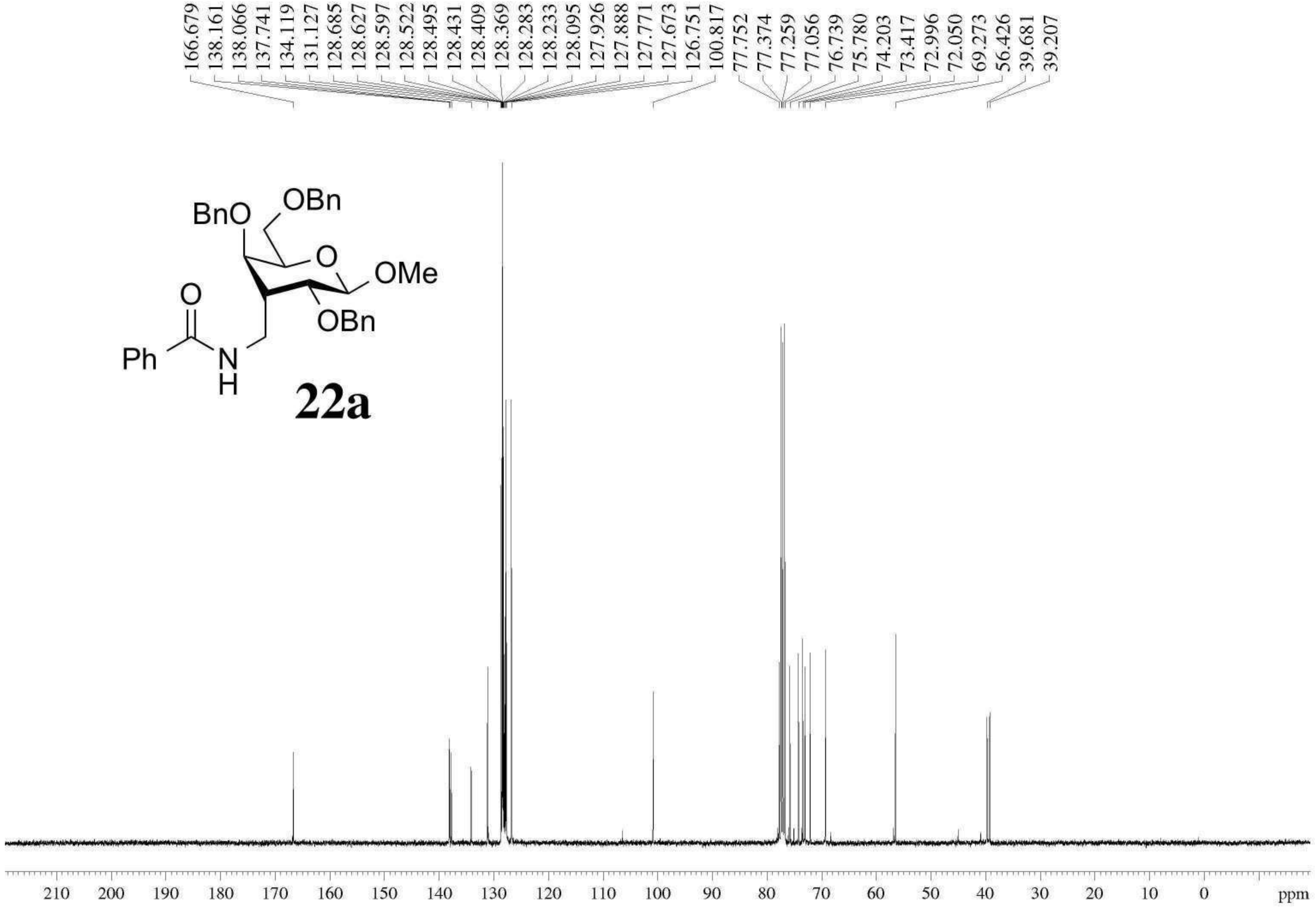
22a
166.679
138.161
138.066
137.741
134.119
131.127
128.685
128.627
128.597
128.522
128.495
128.431
128.409
128.369
128.283
128.233
128.095
127.926
127.888
127.771
127.673
126.751
100.817
77.752
77.374
77.259
77.056
76.739
75.780
74.203
73.417
72.996
72.050
69.273
56.426
39.681
39.207
210 200 190 180 170 160 150 140 130 120 110 100 90 80 70 60 50 40 30 20 10 0 ppm

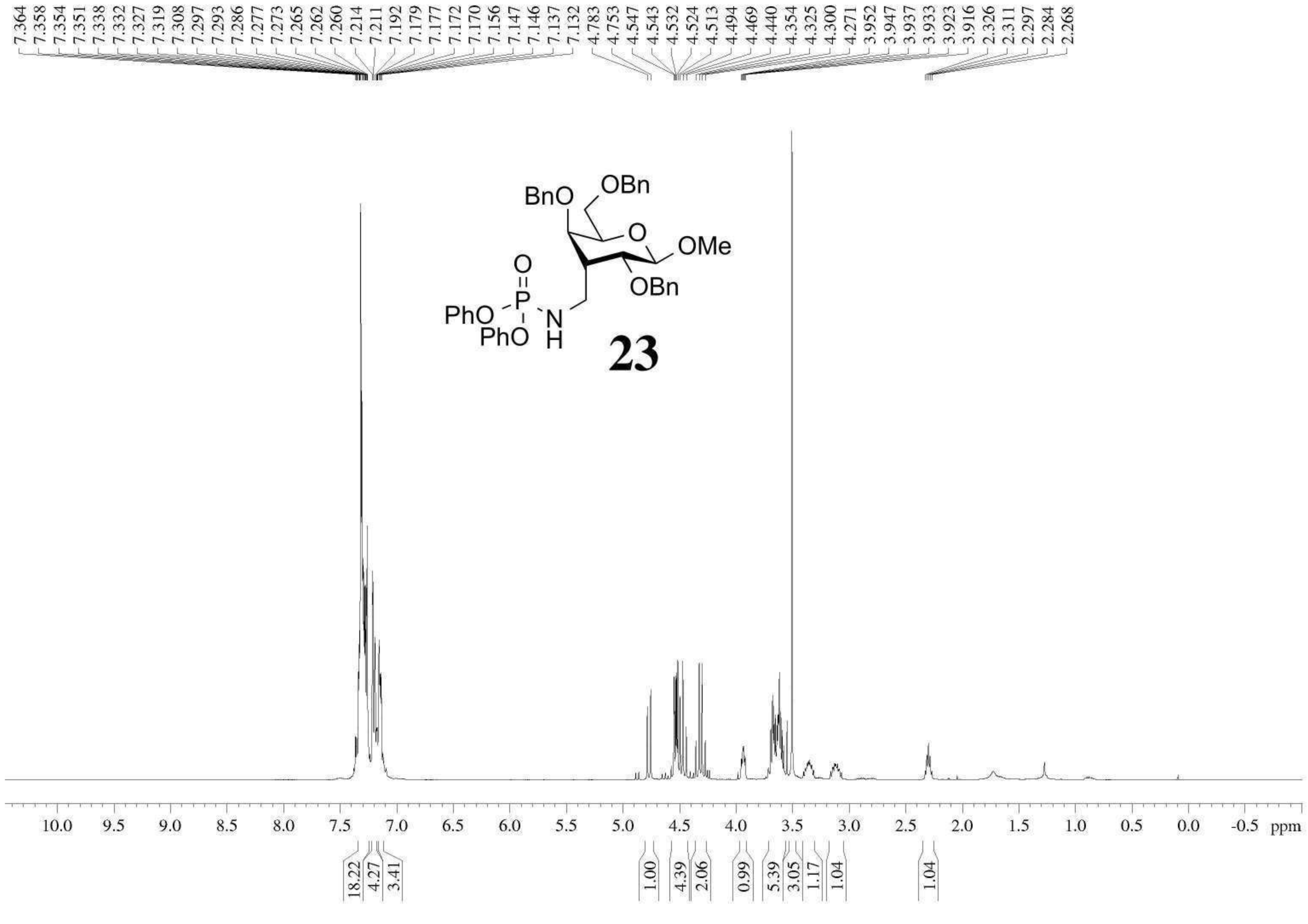

7.364
7.358
7.354
7.351
7.338
7.332
7.327
7.319
7.308
7.297
7.293
7.286
7.277
7.273
7.265
7.262
7.260
7.214
7.211
7.192
7.179
7.177
7.172
7.170
7.156
7.147
7.146
7.137
7.132
4.783
4.753
4.547
4.543
4.532
4.524
4.513
4.494
4.469
4.440
4.354
4.325
4.300
4.271
3.952
3.947
3.937
3.933
3.923
3.916
2.326
2.311
2.297
2.284
2.268
BnO
OBn
O
OMe
OBn
O
P
PhO
PhO
N
H
23
10.0
9.5
9.0
8.5
8.0
7.5
7.0
6.5
6.0
5.5
5.0
4.5
4.0
3.5
3.0
2.5
2.0
1.5
1.0
0.5
0.0
-0.5
ppm
18.22
4.27
3.41
1.00
4.39
2.06
0.99
5.39
3.05
1.17
1.04
1.04

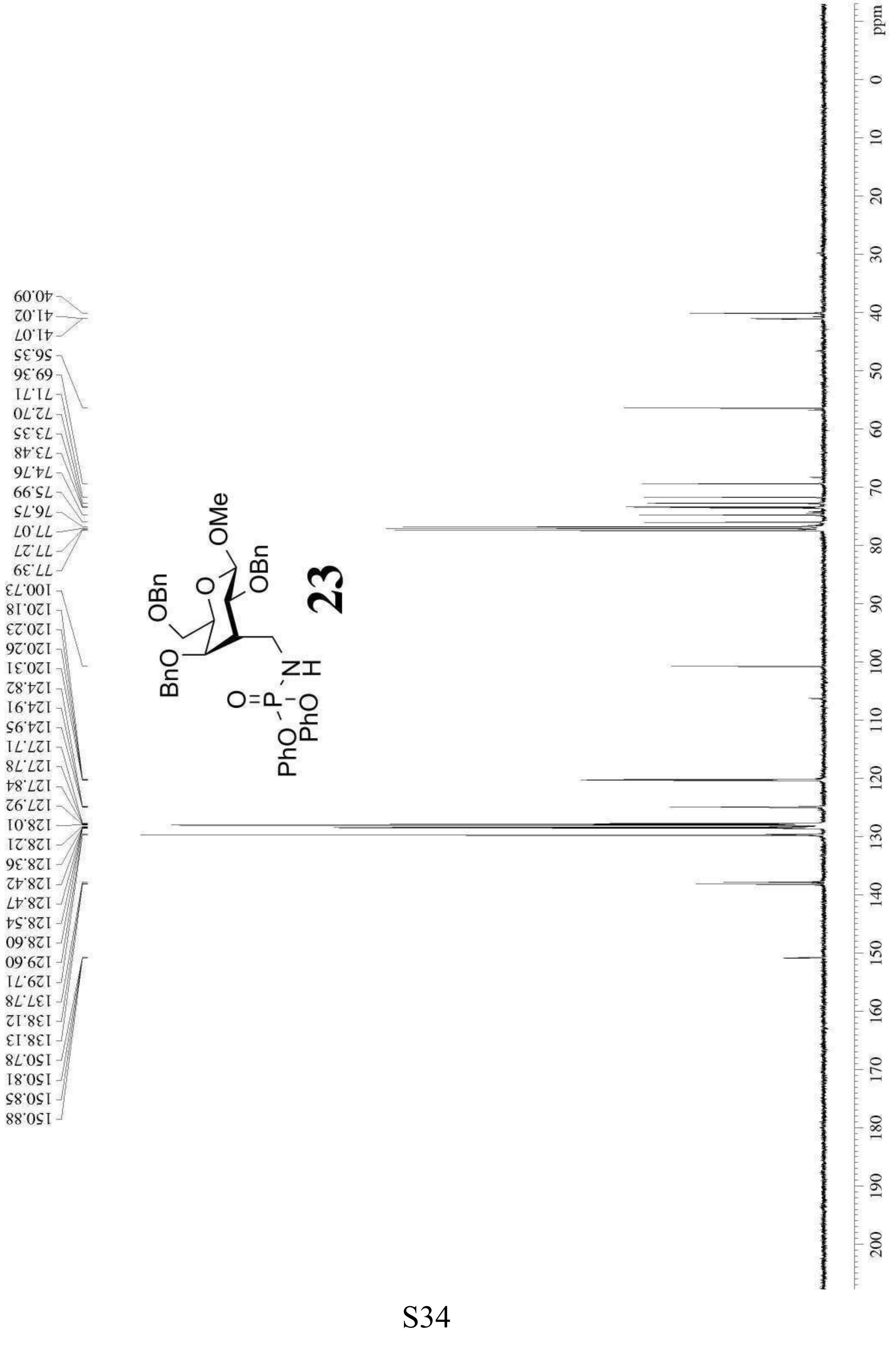
40.09
41.02
41.07
56.35
69.36
71.71
72.70
73.35
73.48
74.76
75.99
76.75
77.07
77.27
77.39
100.73
120.18
120.23
120.26
120.31
124.82
124.91
124.95
127.71
127.78
127.84
127.92
128.01
128.21
128.36
128.42
128.47
128.54
128.60
129.60
129.71
137.78
138.12
138.13
150.78
150.81
150.85
150.88
OBn
BnO
O
OMe
OBn
O
P
PhO
PhO
N
H
23
ppm
0
10
20
30
40
50
60
70
80
90
100
110
120
130
140
150
160
170
180
190
200

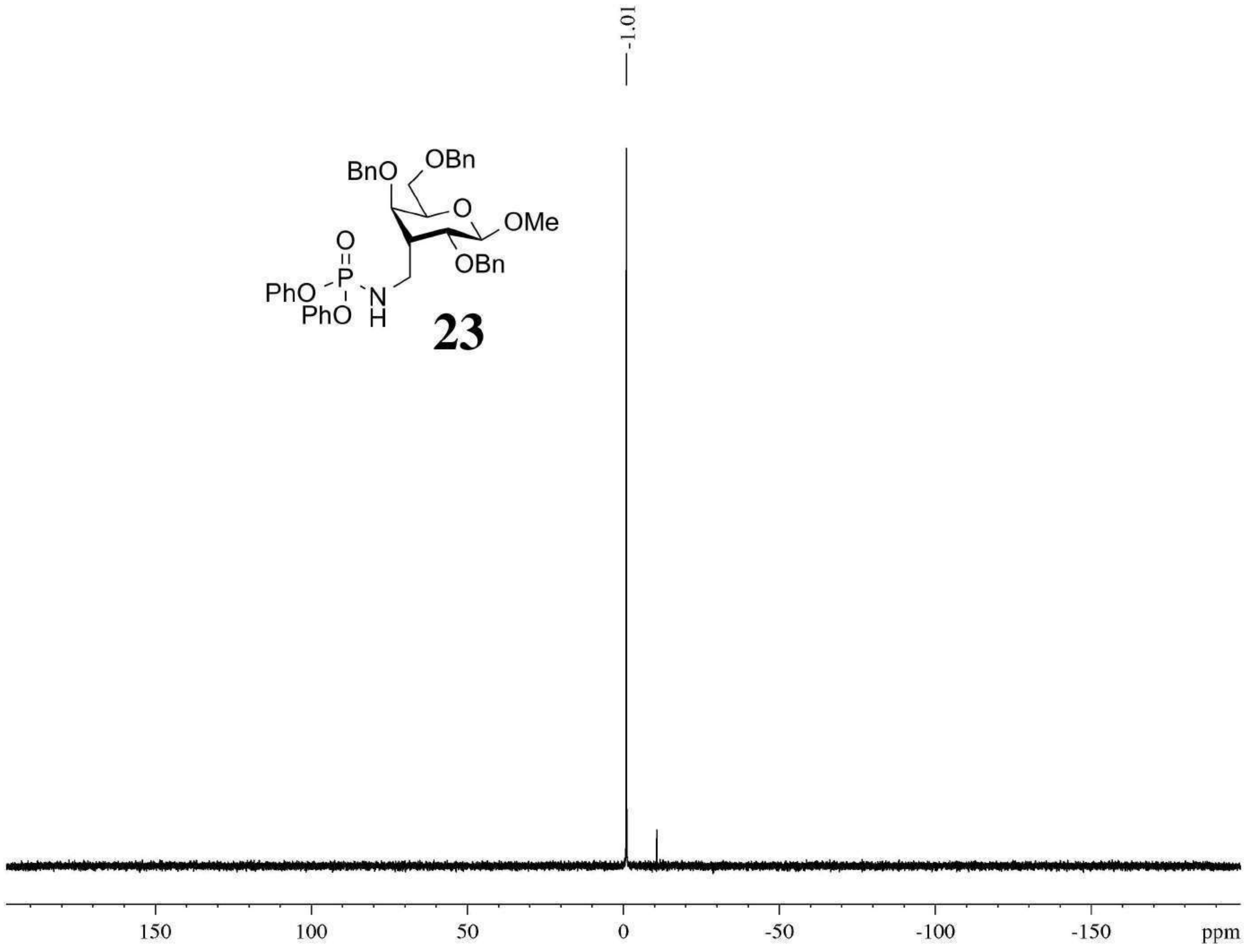
-1.01
BnO
OBn
O
OMe
OBn
PhO
PhO
P
O
N
H
23
150
100
50
0
-50
-100
-150
ppm

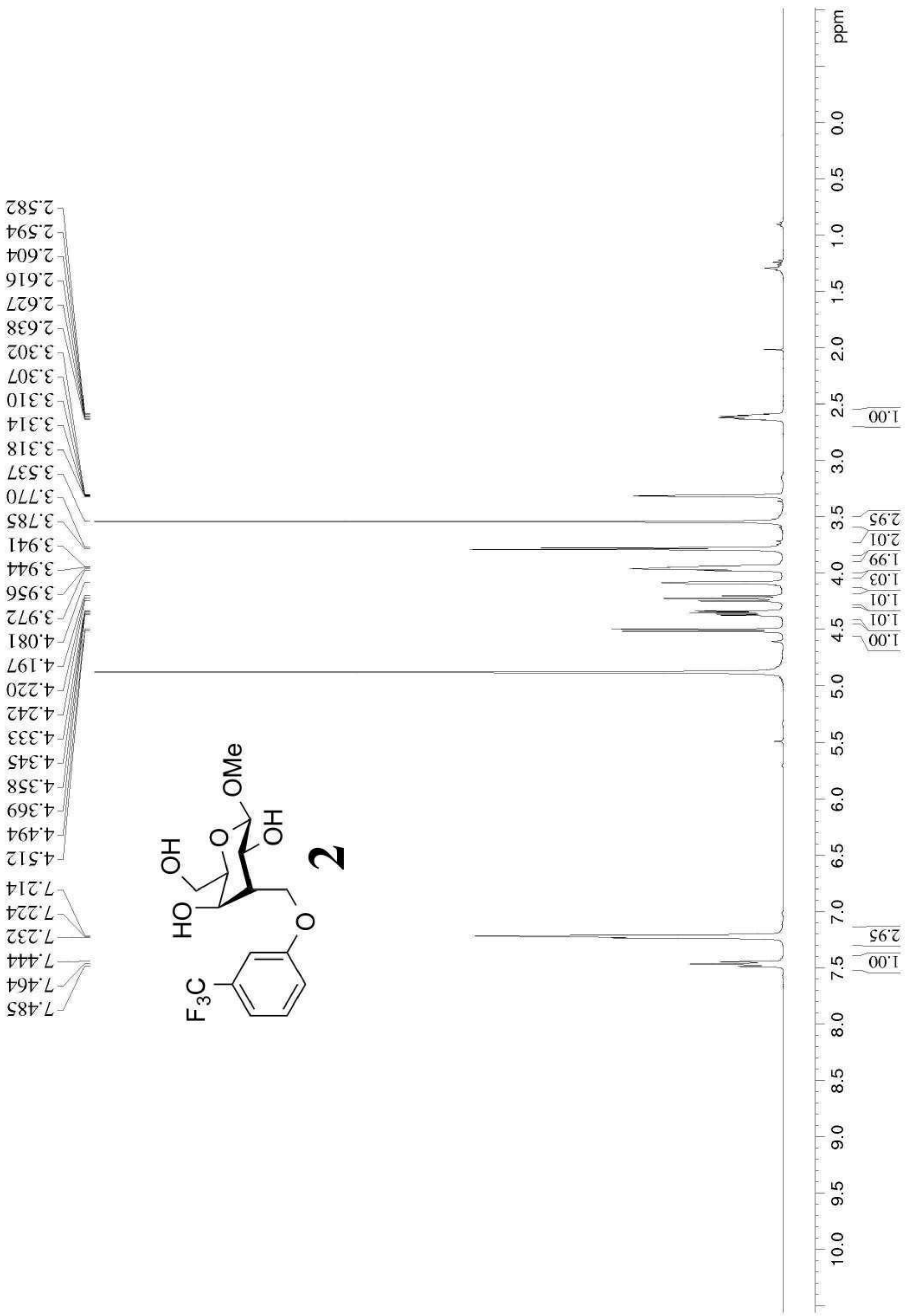
2.582
2.594
2.604
2.616
2.627
2.638
3.302
3.307
3.310
3.314
3.318
3.537
3.770
3.785
3.941
3.944
3.956
3.972
4.081
4.197
4.220
4.242
4.333
4.345
4.358
4.369
4.494
4.512
7.214
7.224
7.232
7.444
7.464
7.485
OMe
OH
HO
F3C
O
2
1.00
2.95
2.01
1.99
1.03
1.01
1.01
1.00
2.95
1.00
ppm

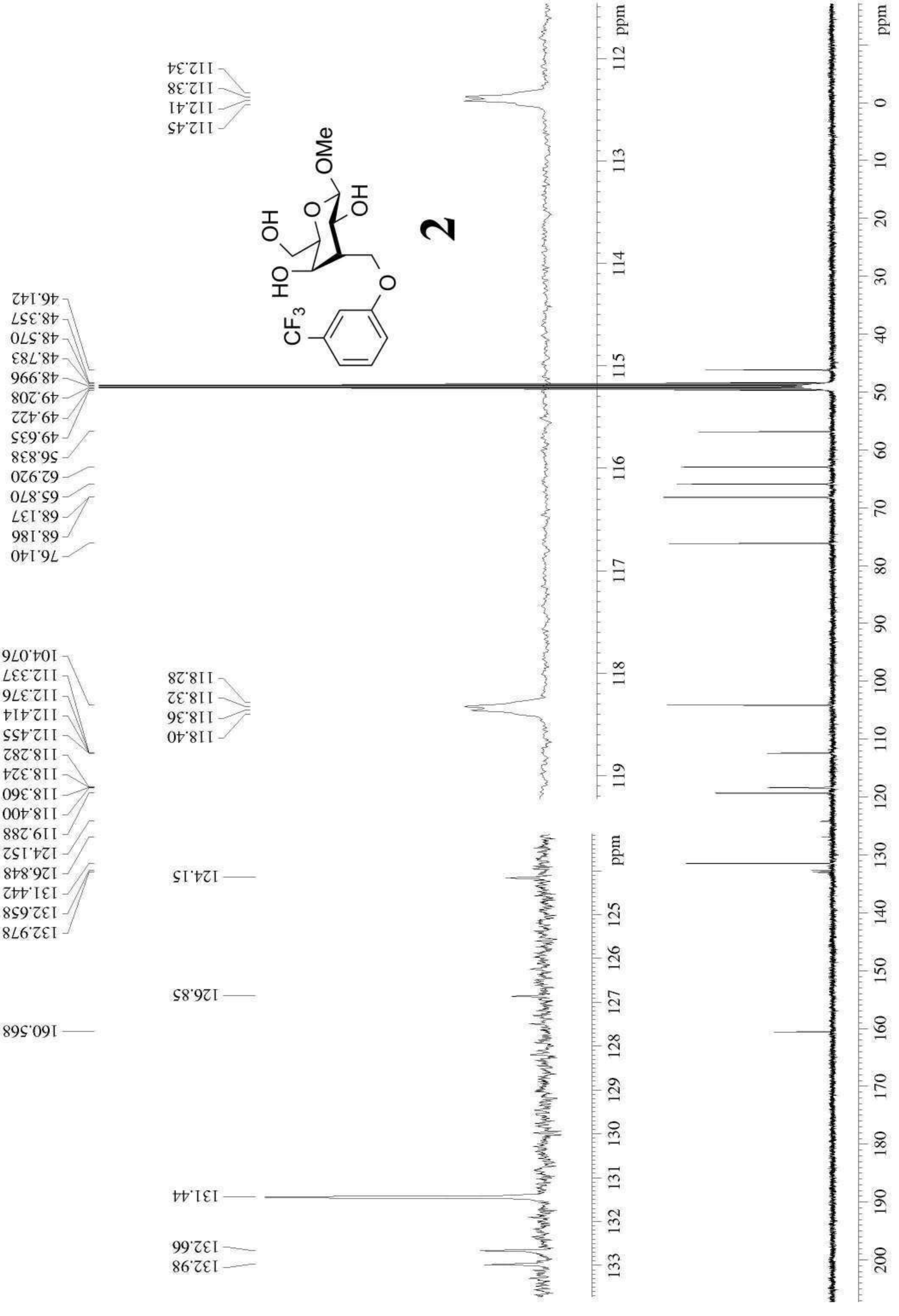
112.34
112.38
112.41
112.45
46.142
48.357
48.570
48.783
48.996
49.208
49.422
49.635
56.838
62.920
65.870
68.137
68.186
76.140
104.076
112.337
112.376
112.414
112.455
118.282
118.324
118.360
118.400
119.288
124.152
126.848
131.442
132.658
132.978
160.568
118.28
118.32
118.36
118.40
124.15
126.85
131.44
132.66
132.98
CF3
HO
OH
O
OMe
OH
O
2
ppm

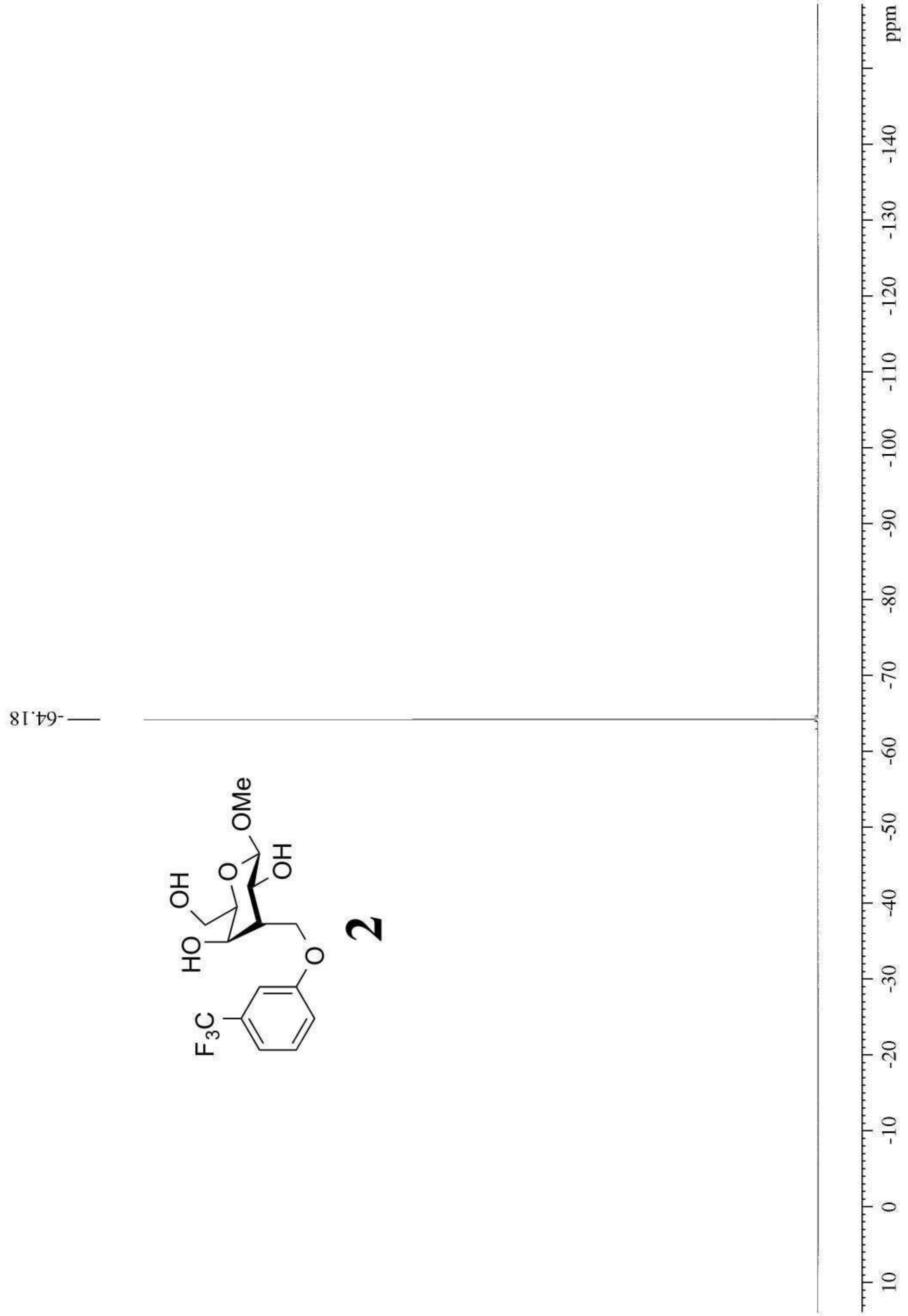
-64.18
F3C
HO
OH
O
OH
OMe
O
2
ppm
-140
-130
-120
-110
-100
-90
-80
-70
-60
-50
-40
-30
-20
-10
0
10

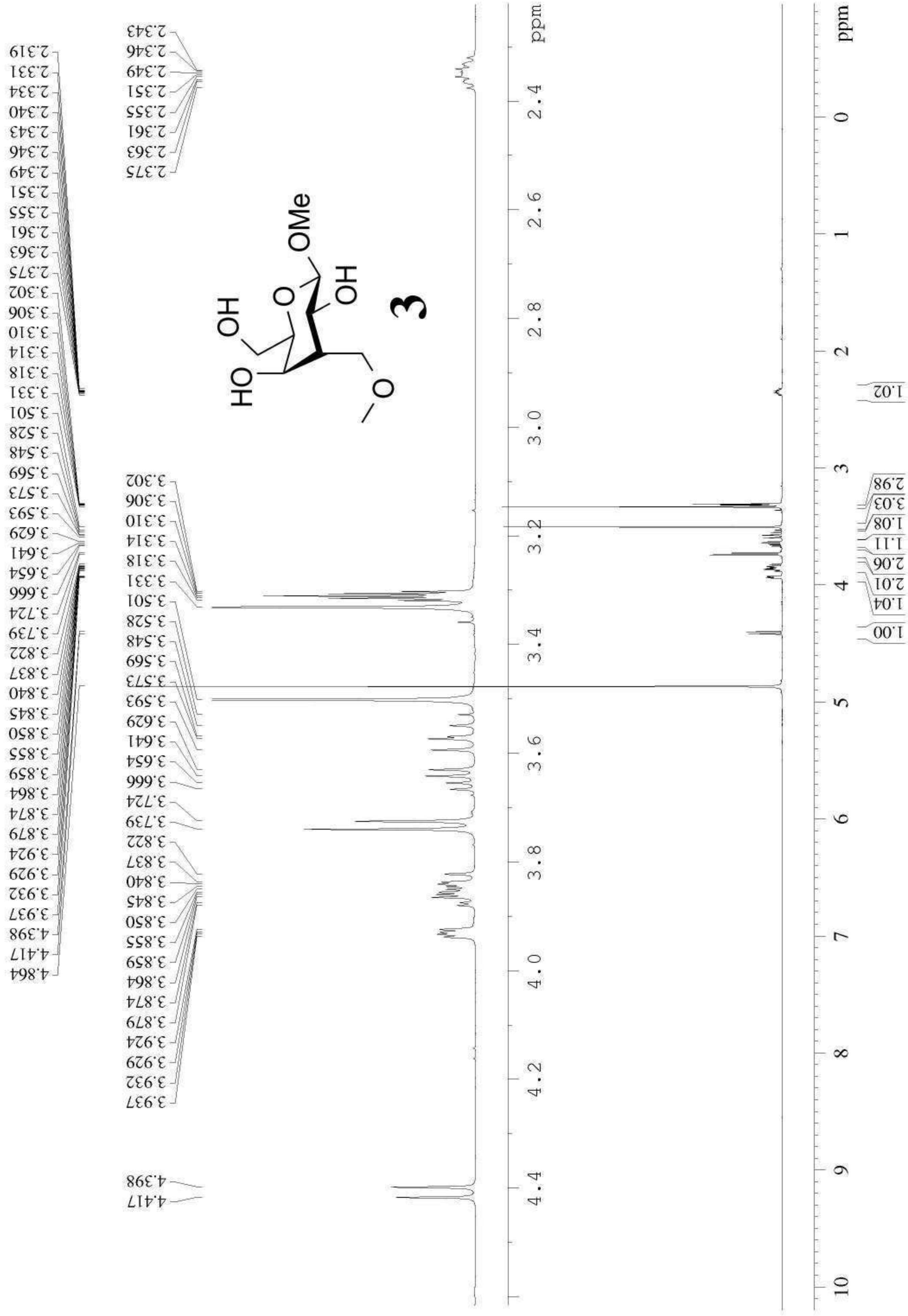
2.319
2.331
2.334
2.340
2.343
2.346
2.349
2.351
2.355
2.361
2.363
2.375
3.302
3.306
3.310
3.314
3.318
3.331
3.501
3.528
3.548
3.569
3.573
3.593
3.629
3.641
3.654
3.666
3.724
3.739
3.822
3.837
3.840
3.845
3.850
3.855
3.859
3.864
3.874
3.879
3.924
3.929
3.932
3.937
4.398
4.417
4.864
OMe
OH
HO
OH
O
3
ppm
1.02
2.98
3.03
1.08
1.11
2.06
2.01
1.04
1.00

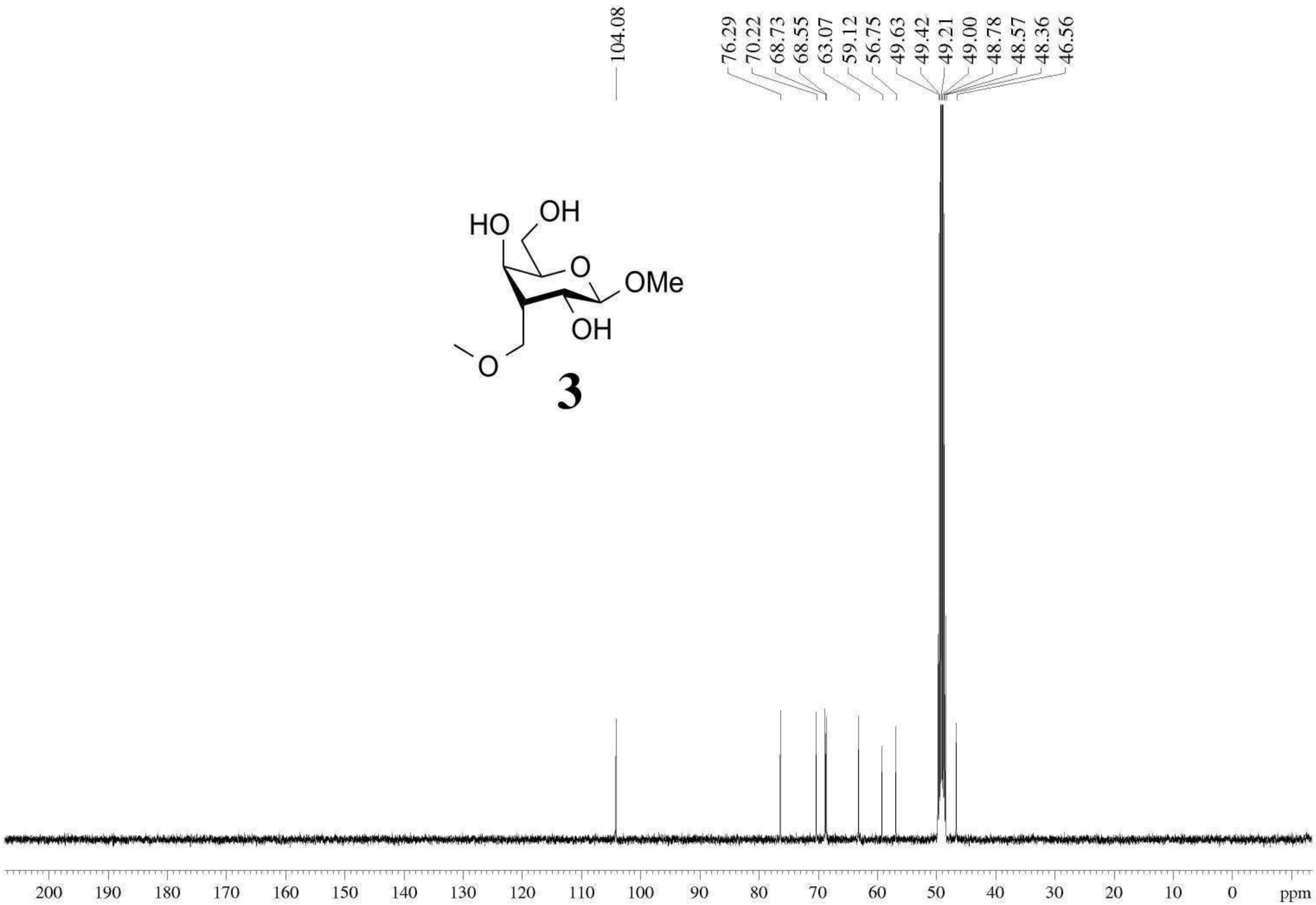
104.08
76.29
70.22
68.73
68.55
63.07
59.12
56.75
49.63
49.42
49.21
49.00
48.78
48.57
48.36
46.56
200 190 180 170 160 150 140 130 120 110 100 90 80 70 60 50 40 30 20 10 0 ppm
HO
OH
O
OMe
OH
O

3

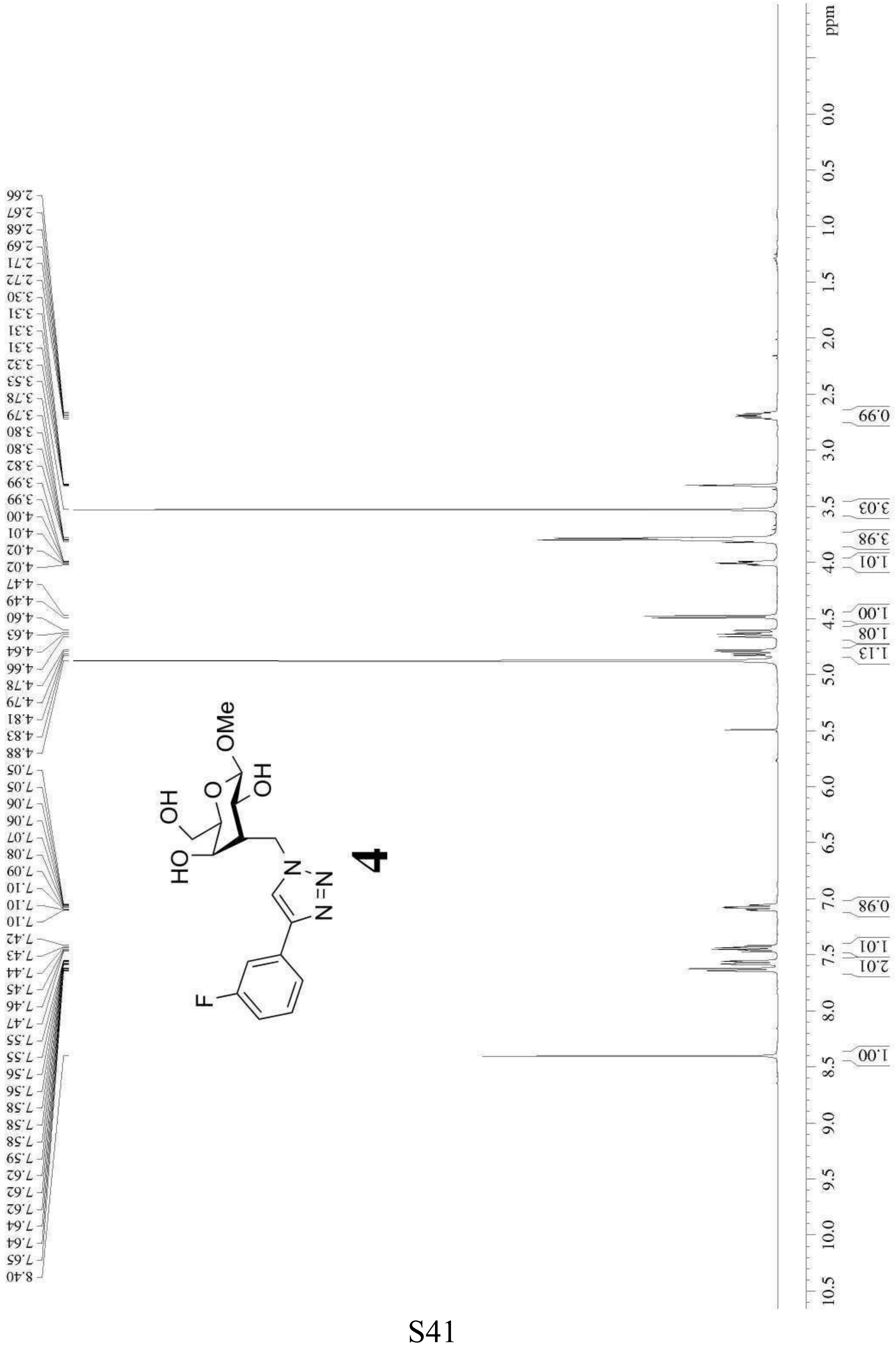
2.66
2.67
2.68
2.69
2.71
2.72
3.30
3.31
3.31
3.31
3.32
3.53
3.78
3.79
3.80
3.80
3.82
3.99
3.99
4.00
4.01
4.02
4.02
4.47
4.49
4.60
4.63
4.64
4.66
4.78
4.79
4.81
4.83
4.88
7.05
7.05
7.06
7.06
7.07
7.08
7.09
7.10
7.10
7.10
7.42
7.43
7.44
7.45
7.46
7.47
7.55
7.55
7.56
7.56
7.58
7.58
7.58
7.59
7.62
7.62
7.62
7.64
7.64
7.65
8.40
HO
OH
O
OMe
OH
N
N=N
F
4
0.99
3.03
3.98
1.01
1.00
1.08
1.13
0.98
1.01
2.01
1.00
ppm

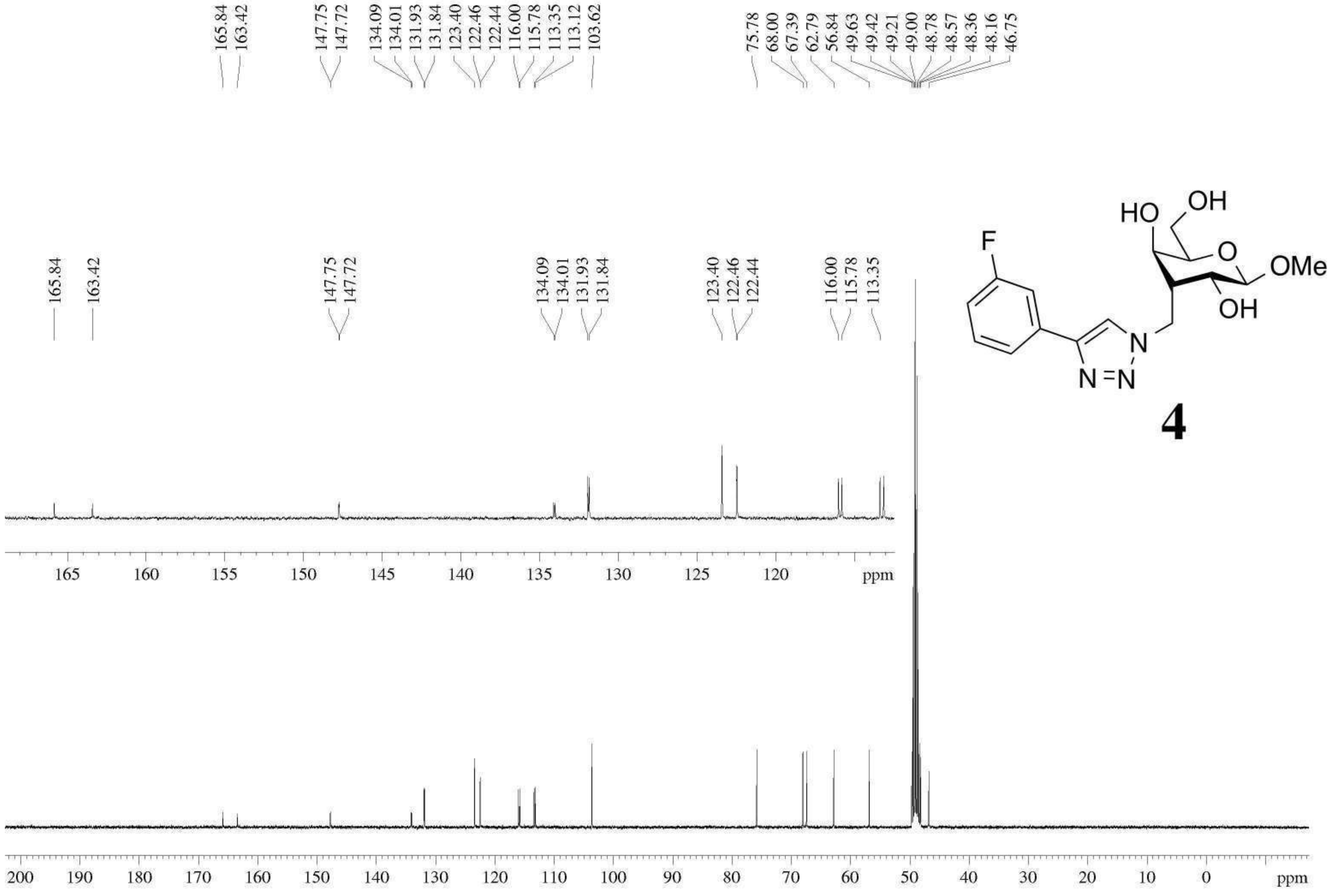

165.84
163.42
147.75
147.72
134.09
134.01
131.93
131.84
123.40
122.46
122.44
116.00
115.78
113.35
113.12
103.62
75.78
68.00
67.39
62.79
56.84
49.63
49.42
49.21
49.00
48.78
48.57
48.36
48.16
46.75
165.84
163.42
147.75
147.72
134.09
134.01
131.93
131.84
123.40
122.46
122.44
116.00
115.78
113.35
165 160 155 150 145 140 135 130 125 120 ppm
200 190 180 170 160 150 140 130 120 110 100 90 80 70 60 50 40 30 20 10 0 ppm
F
HO
OH
O
OMe
OH
N
N=N
4

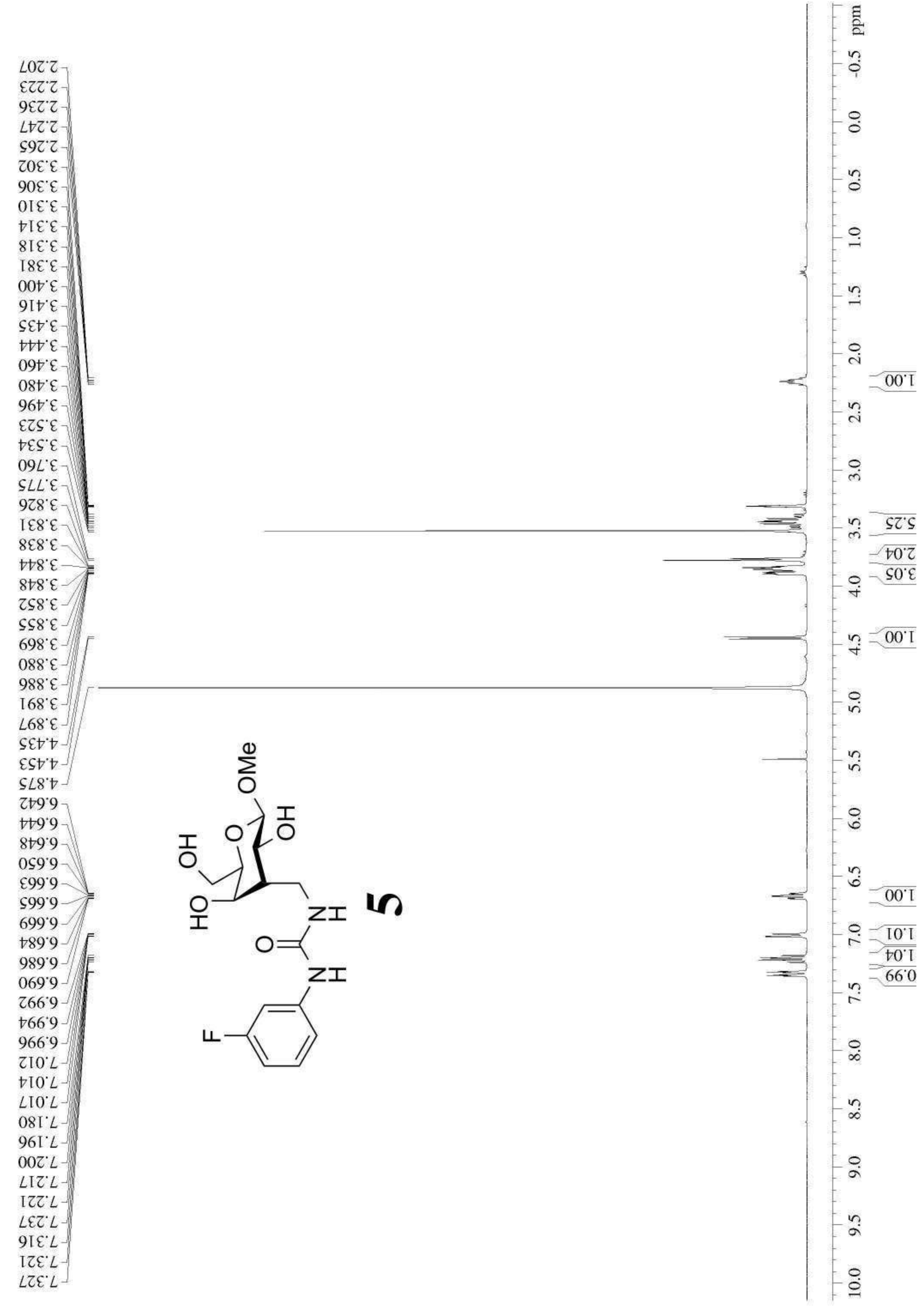

2.207
2.223
2.236
2.247
2.265
3.302
3.306
3.310
3.314
3.318
3.381
3.400
3.416
3.435
3.444
3.460
3.480
3.496
3.523
3.534
3.760
3.775
3.826
3.831
3.838
3.844
3.848
3.852
3.855
3.869
3.880
3.886
3.891
3.897
4.435
4.453
4.875
6.642
6.644
6.648
6.650
6.663
6.665
6.669
6.684
6.686
6.690
6.992
6.994
6.996
7.012
7.014
7.017
7.180
7.196
7.200
7.217
7.221
7.237
7.316
7.321
7.327
OH
HO
O
OMe
F
N
H
N
H
O
5
1.00
5.25
2.04
3.05
1.00
1.00
1.01
1.04
0.99
ppm
-0.5
0.0
0.5
1.0
1.5
2.0
2.5
3.0
3.5
4.0
4.5
5.0
5.5
6.0
6.5
7.0
7.5
8.0
8.5
9.0
9.5
10.0

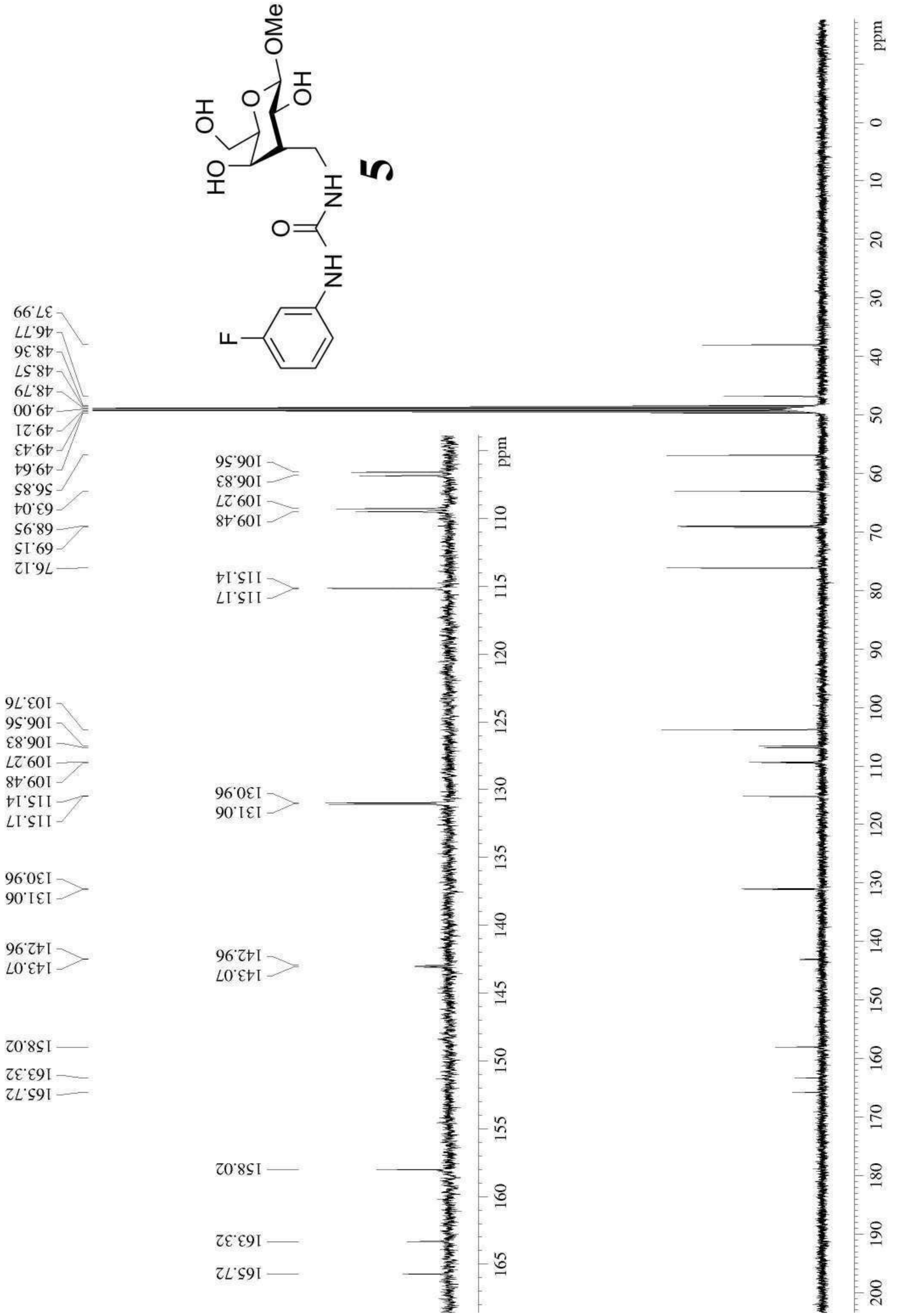

37.99
46.77
48.36
48.57
48.79
49.00
49.21
49.43
49.64
56.85
63.04
68.95
69.15
76.12
103.76
106.56
106.83
109.27
109.48
115.14
115.17
130.96
131.06
142.96
143.07
158.02
163.32
165.72
ppm
5

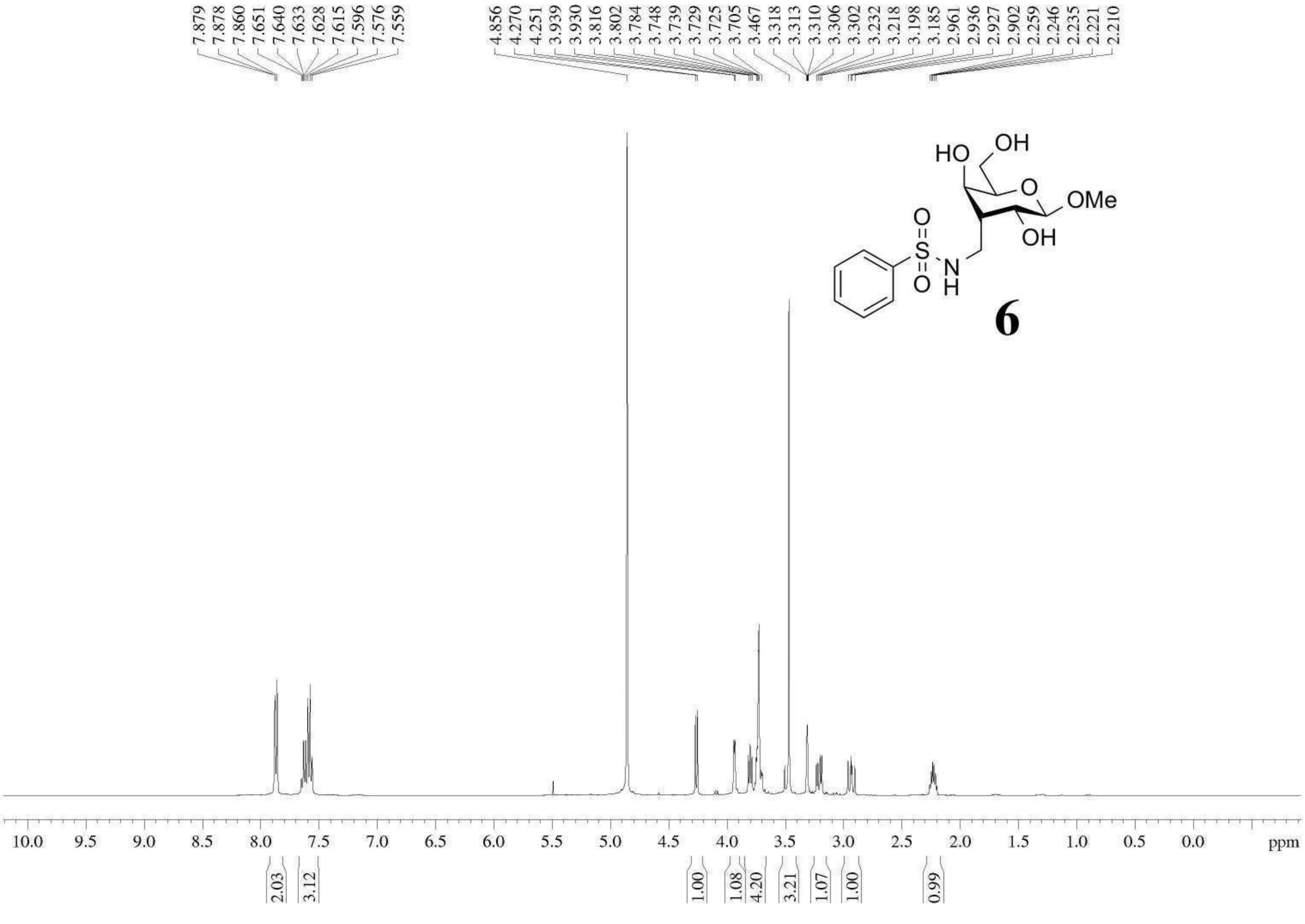
7.879
7.878
7.860
7.651
7.640
7.633
7.628
7.615
7.596
7.576
7.559
4.856
4.270
4.251
3.939
3.930
3.816
3.802
3.784
3.748
3.739
3.729
3.725
3.705
3.467
3.318
3.313
3.310
3.306
3.302
3.232
3.218
3.198
3.185
2.961
2.936
2.927
2.902
2.259
2.246
2.235
2.221
2.210
HO
OH
O
OMe
OH
O
S
O
N
H
6
10.0 9.5 9.0 8.5 8.0 7.5 7.0 6.5 6.0 5.5 5.0 4.5 4.0 3.5 3.0 2.5 2.0 1.5 1.0 0.5 0.0 ppm
2.03
3.12
1.00
1.08
4.20
3.21
1.07
1.00
0.99

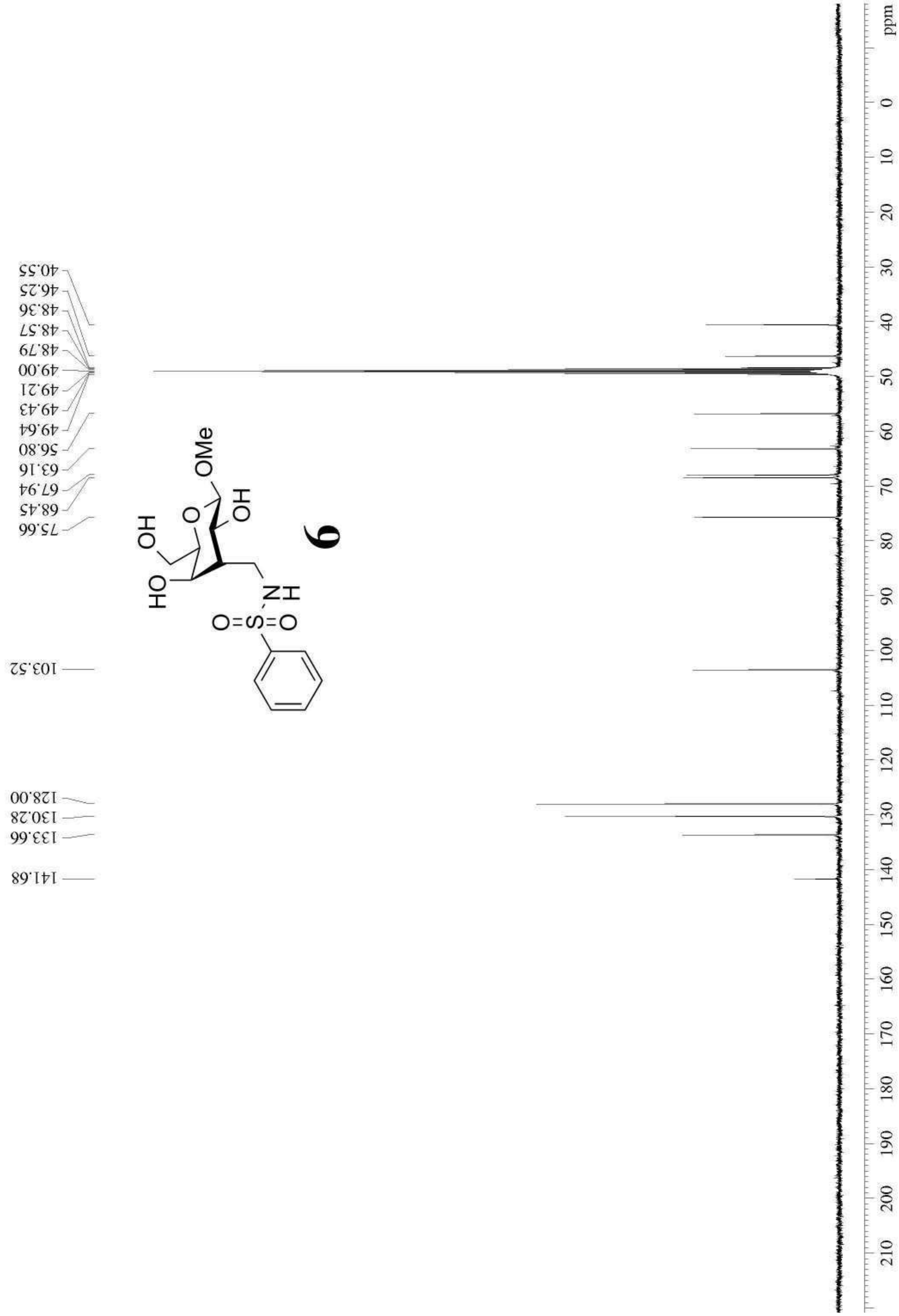
40.55
46.25
48.36
48.57
48.79
49.00
49.21
49.43
49.64
56.80
63.16
67.94
68.45
75.66
103.52
128.00
130.28
133.66
141.68
6
ppm
0
10
20
30
40
50
60
70
80
90
100
110
120
130
140
150
160
170
180
190
200
210

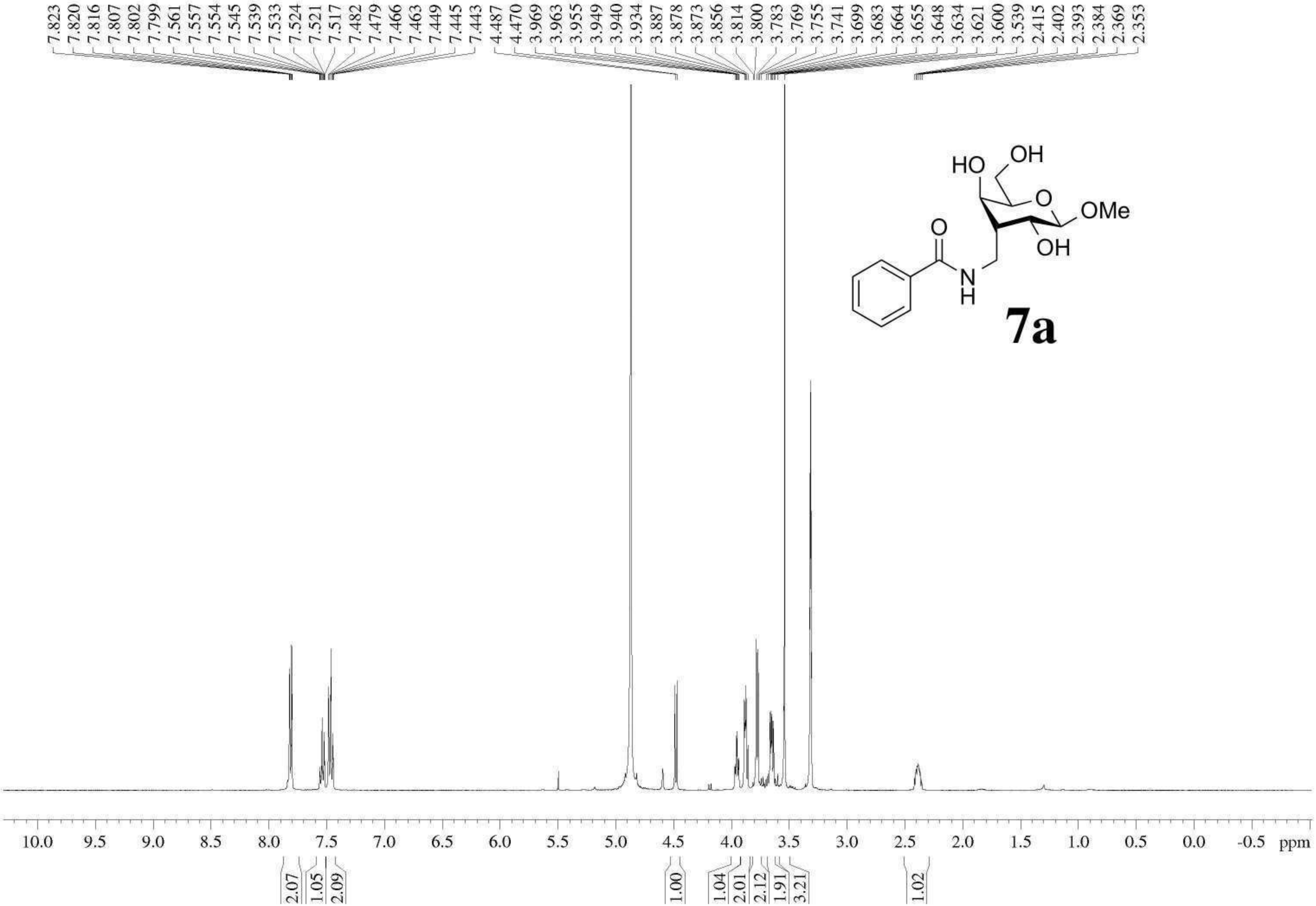
7.823
7.820
7.816
7.807
7.802
7.799
7.561
7.557
7.554
7.545
7.539
7.533
7.524
7.521
7.517
7.482
7.479
7.466
7.463
7.449
7.445
7.443
4.487
4.470
3.969
3.963
3.955
3.949
3.940
3.934
3.887
3.878
3.873
3.856
3.814
3.800
3.783
3.769
3.755
3.741
3.699
3.683
3.664
3.655
3.648
3.634
3.621
3.600
3.539
2.415
2.402
2.393
2.384
2.369
2.353
HO
OH
O
OMe
OH
O
N
H
7a
10.0 9.5 9.0 8.5 8.0 7.5 7.0 6.5 6.0 5.5 5.0 4.5 4.0 3.5 3.0 2.5 2.0 1.5 1.0 0.5 0.0 -0.5 ppm
2.07
1.05
2.09
1.00
1.04
2.01
2.12
1.91
3.21
1.02

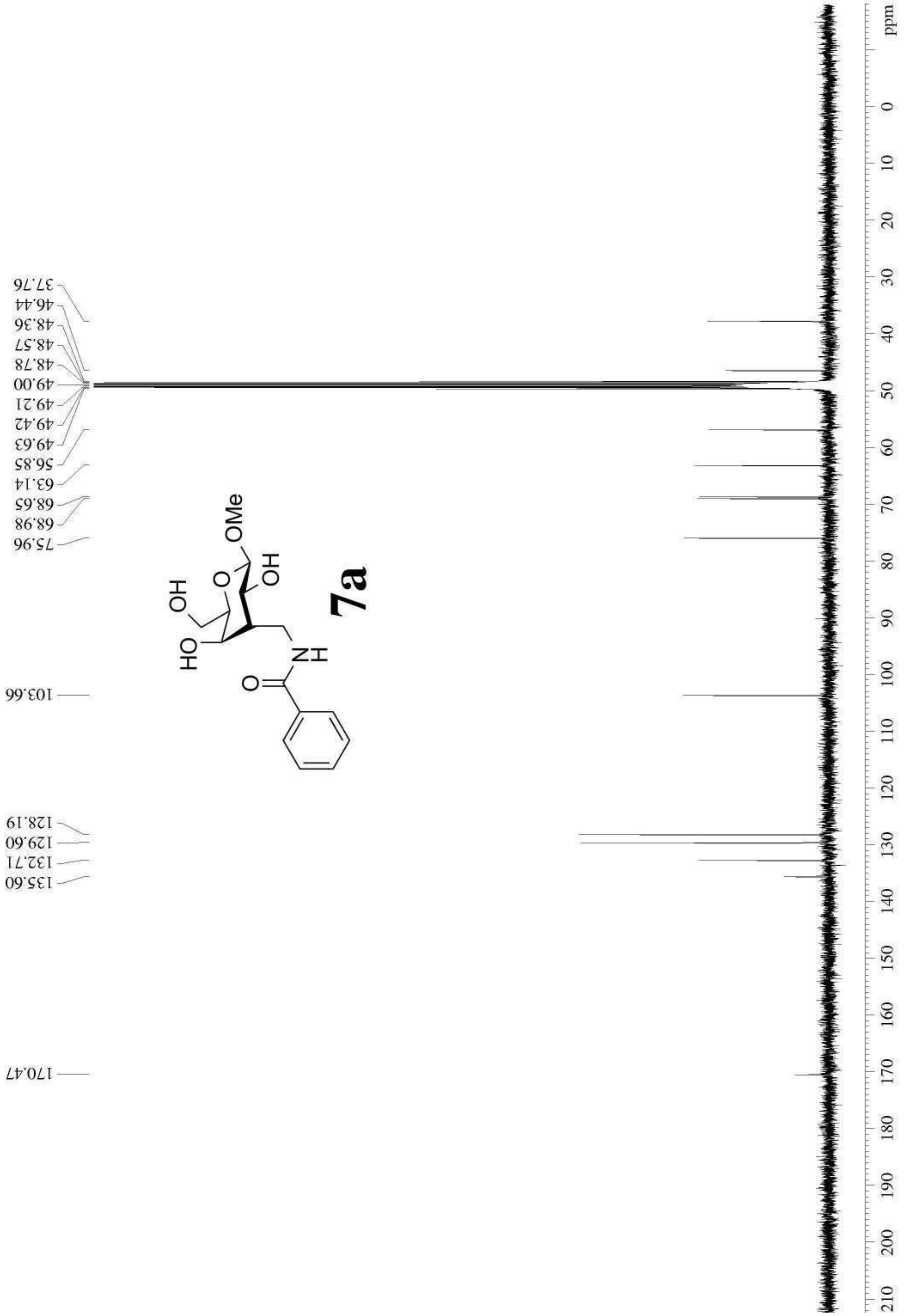

37.76
46.44
48.36
48.57
48.78
49.00
49.21
49.42
49.63
56.85
63.14
68.65
68.98
75.96
103.66
128.19
129.60
132.71
135.60
170.47
OMe
OH
HO
O
NH
7a
ppm

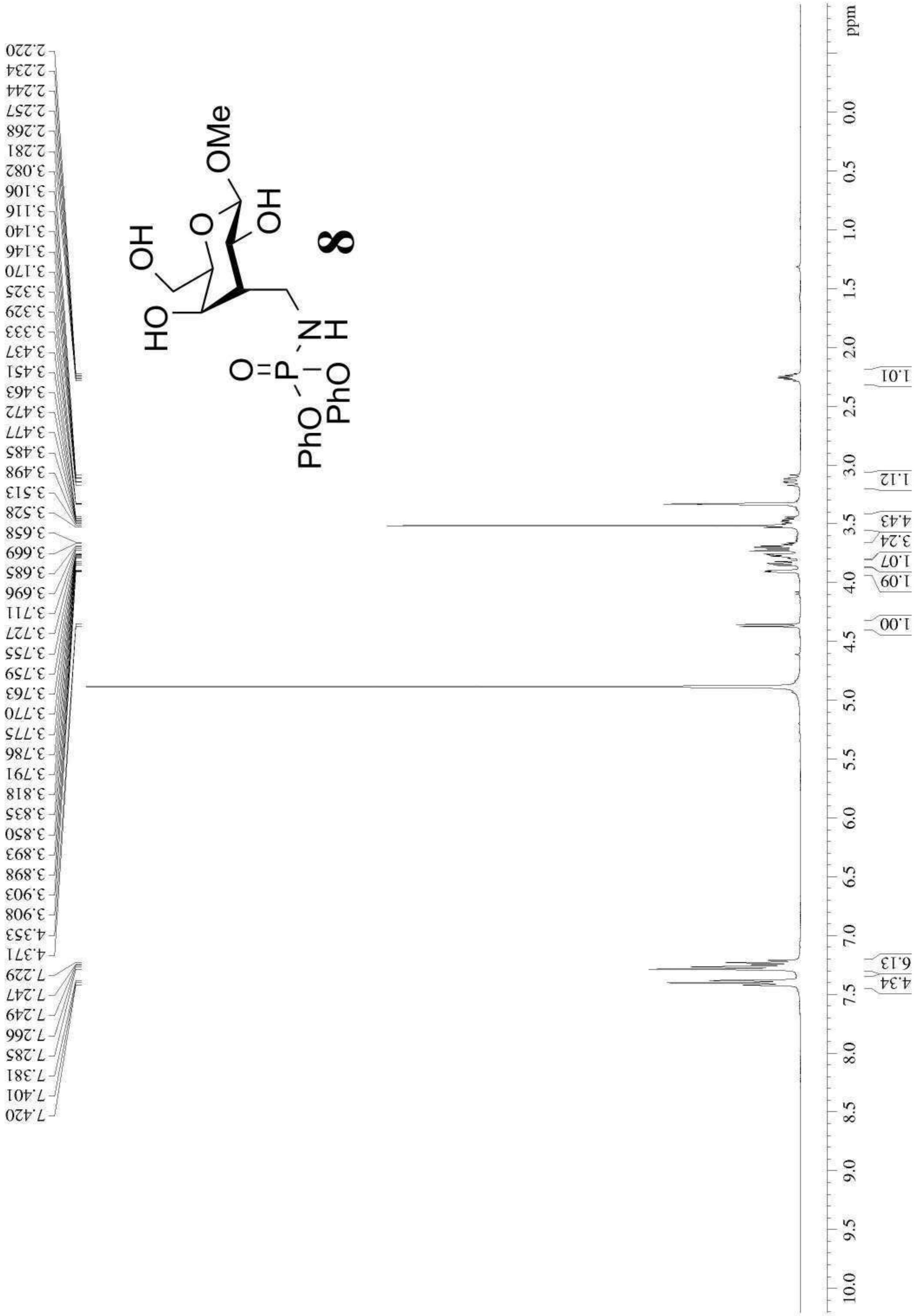
2.220
2.234
2.244
2.257
2.268
2.281
3.082
3.106
3.116
3.140
3.146
3.170
3.325
3.329
3.333
3.437
3.451
3.463
3.472
3.477
3.485
3.498
3.513
3.528
3.658
3.669
3.685
3.696
3.711
3.727
3.755
3.759
3.763
3.770
3.775
3.786
3.791
3.818
3.835
3.850
3.893
3.898
3.903
3.908
4.353
4.371
7.229
7.247
7.249
7.266
7.285
7.381
7.401
7.420
OMe
OH
HO
O
PhO
P
N
H
1.01
1.12
4.43
3.24
1.07
1.09
1.00
6.13
4.34
ppm

8

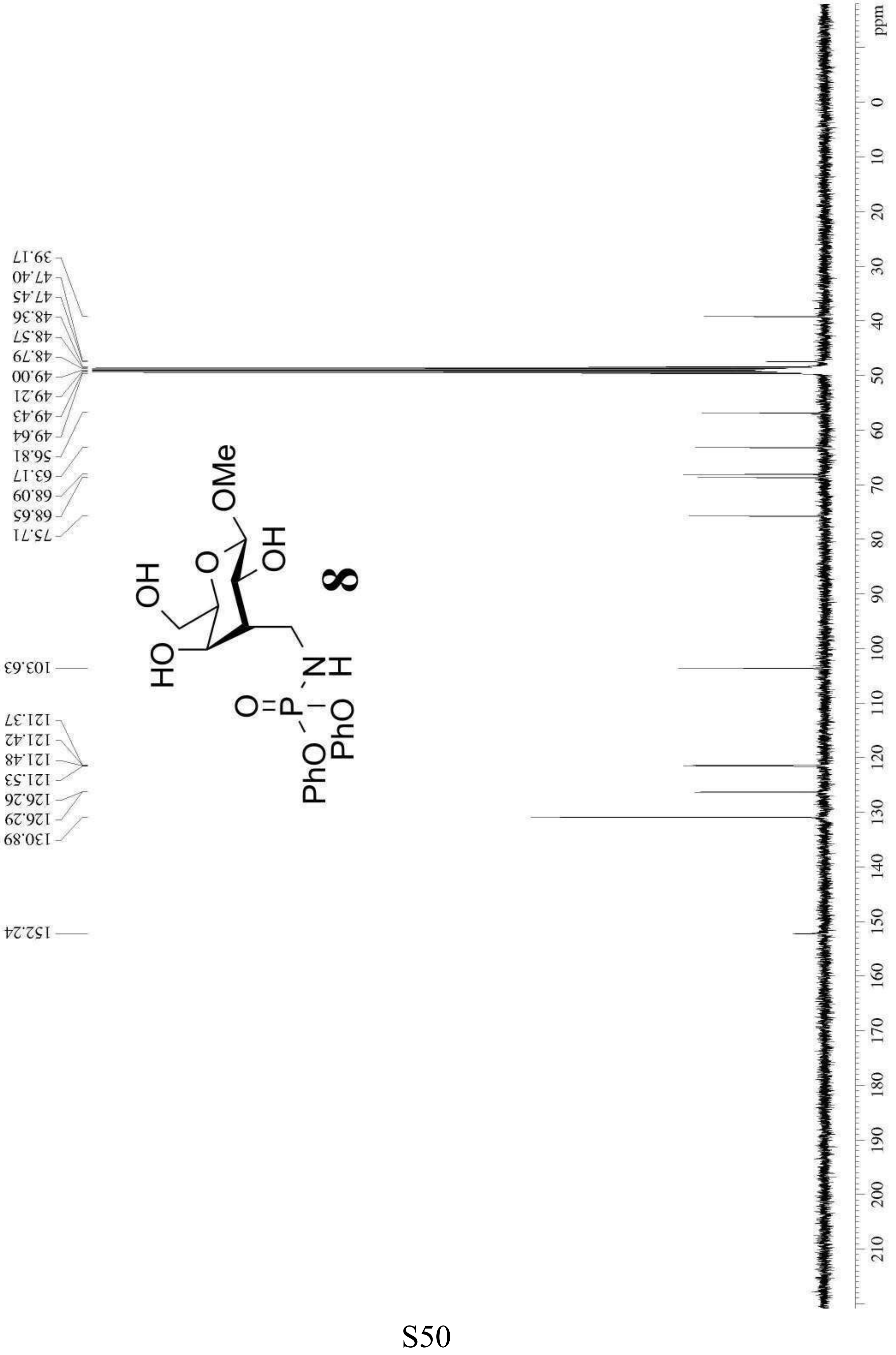
39.17
47.40
47.45
48.36
48.57
48.79
49.00
49.21
49.43
49.64
56.81
63.17
68.09
68.65
75.71
103.63
121.37
121.42
121.48
121.53
126.26
126.29
130.89
152.24
OMe
OH
HO
O
N
H
P
PhO
8
ppm
0
10
20
30
40
50
60
70
80
90
100
110
120
130
140
150
160
170
180
190
200
210

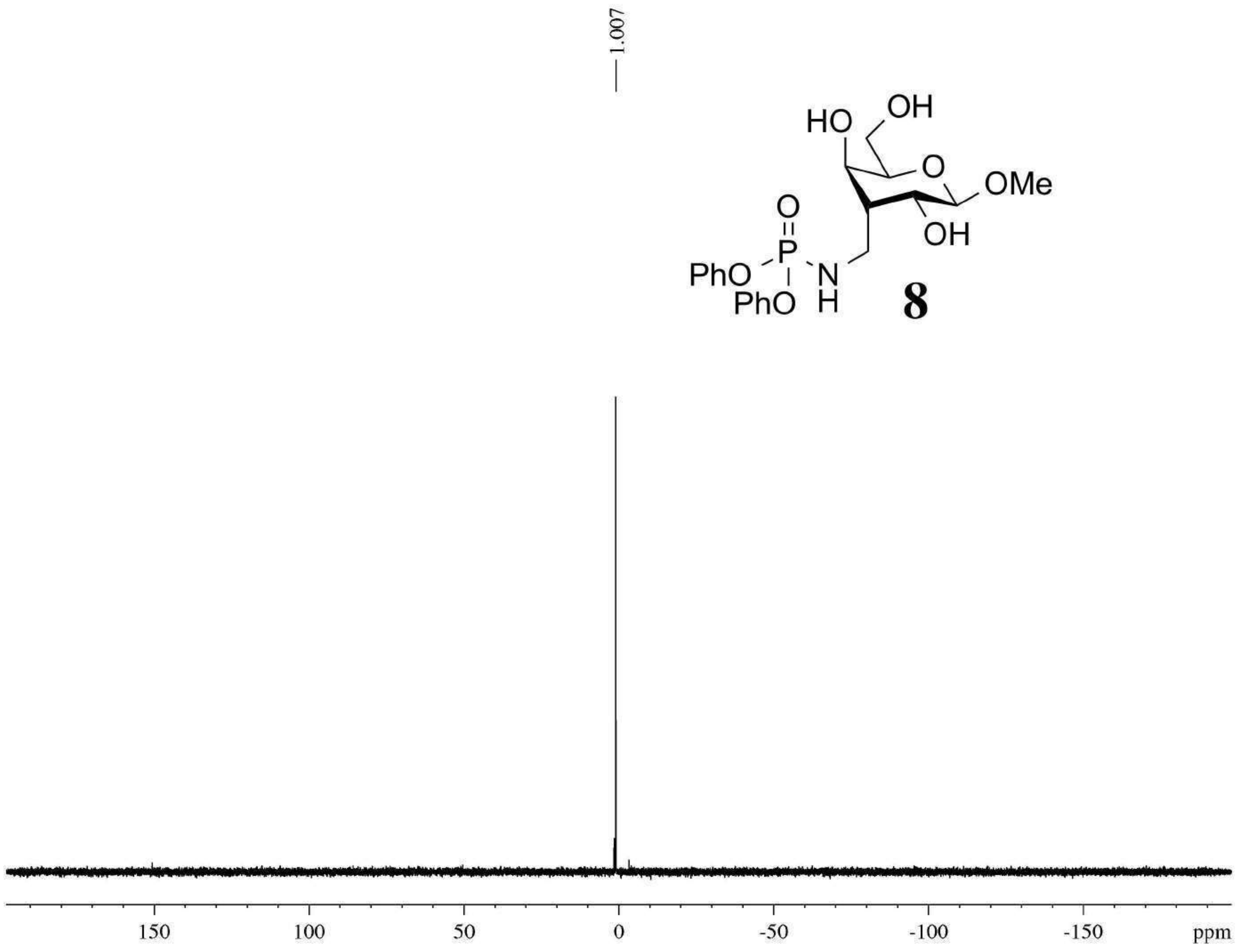

1.007
HO
OH
O
OMe
OH
O
P
PhO
PhO
N
H
8
150
100
50
0
-50
-100
-150
ppm

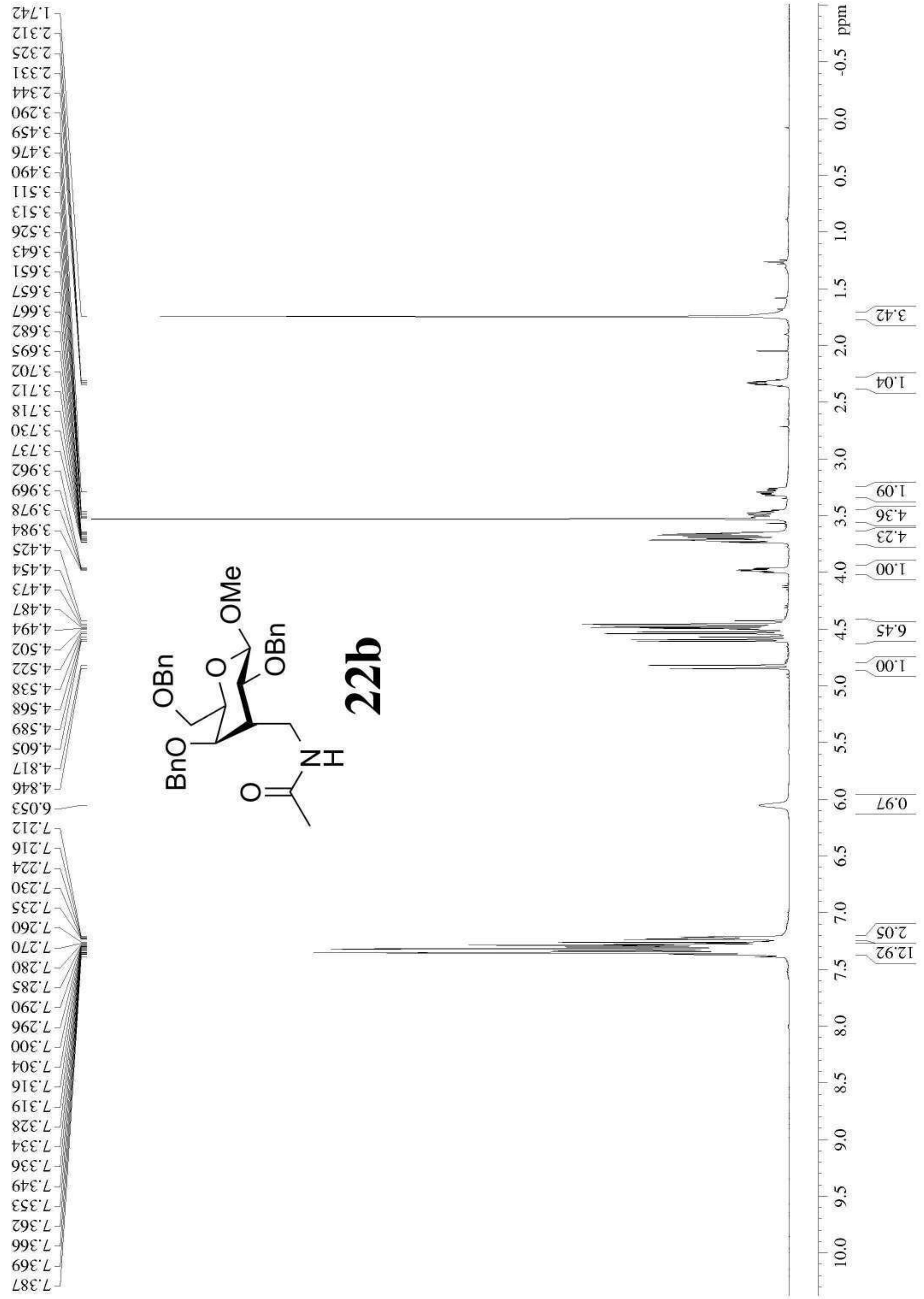
22b
ppm

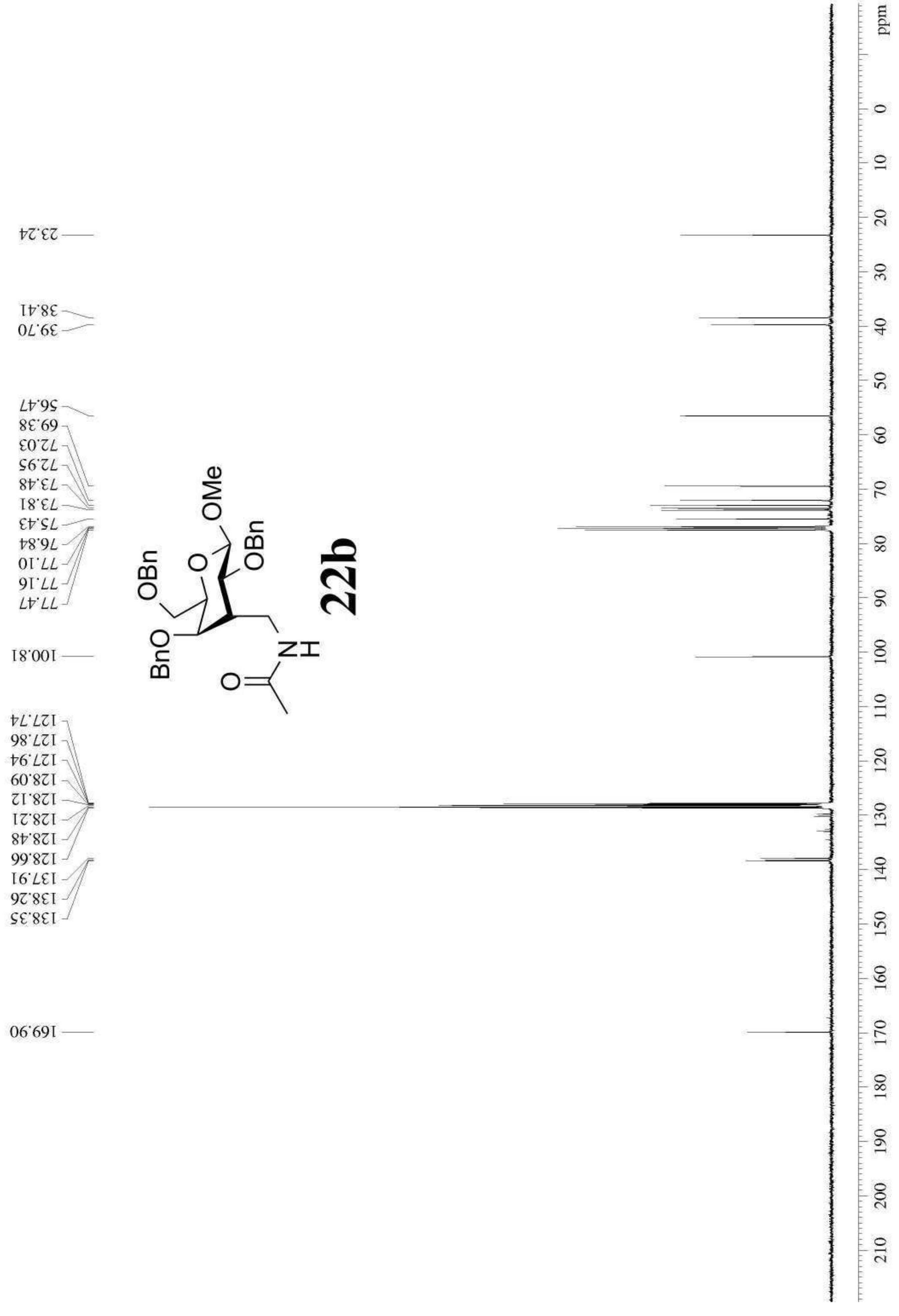

23.24
38.41
39.70
56.47
69.38
72.03
72.95
73.48
73.81
75.43
76.84
77.10
77.16
77.47
100.81
127.74
127.86
127.94
128.09
128.12
128.21
128.48
128.66
137.91
138.26
138.35
169.90
BnO
OBn
O
OMe
OBn
N
H
O
22b
ppm

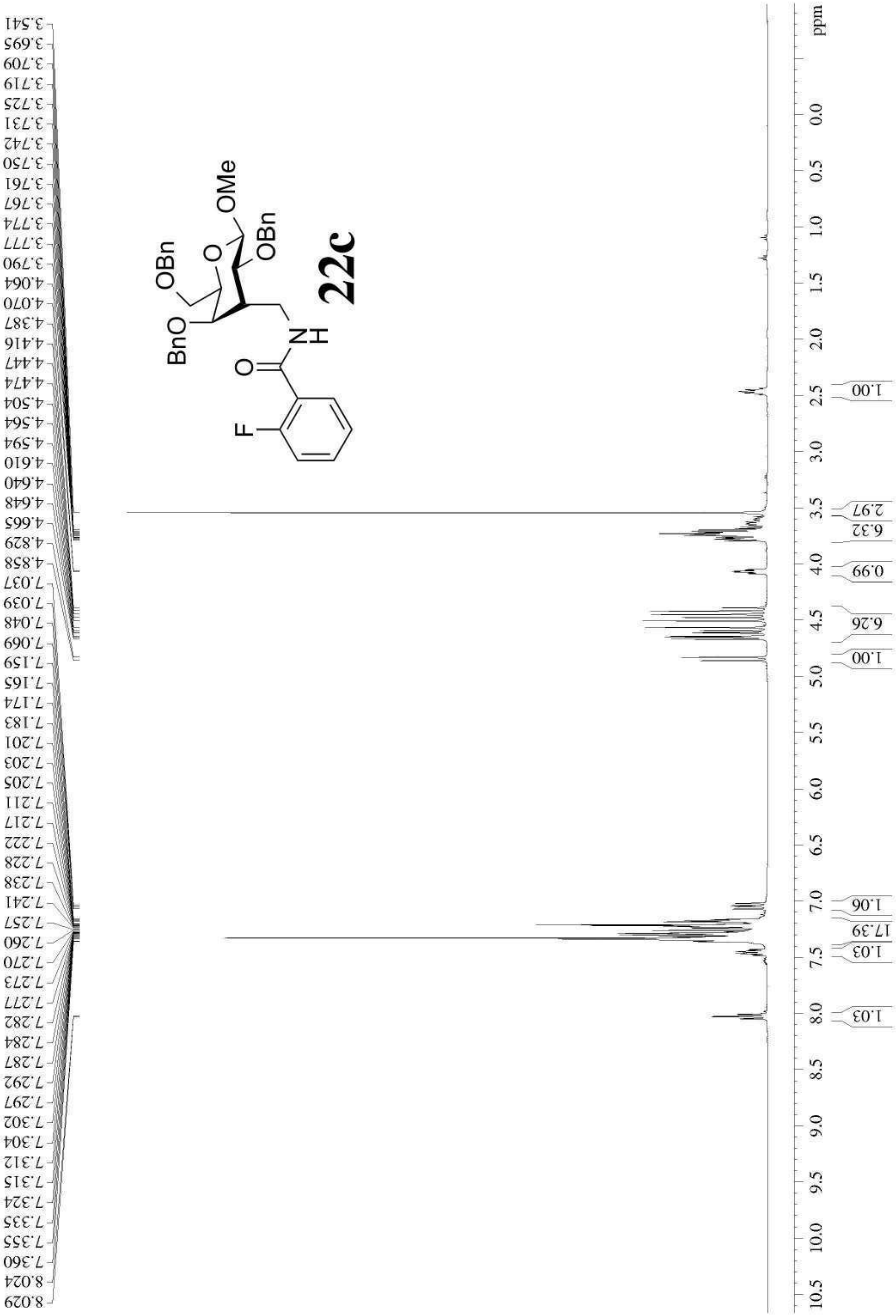
3.541
3.695
3.709
3.719
3.725
3.731
3.742
3.750
3.761
3.767
3.774
3.777
3.790
4.064
4.070
4.387
4.416
4.447
4.474
4.504
4.564
4.594
4.610
4.640
4.648
4.665
4.829
4.858
7.037
7.039
7.048
7.069
7.159
7.165
7.174
7.183
7.201
7.203
7.205
7.211
7.217
7.222
7.228
7.238
7.241
7.257
7.260
7.270
7.273
7.277
7.282
7.284
7.287
7.292
7.297
7.302
7.304
7.312
7.315
7.324
7.335
7.355
7.360
8.024
8.029
F
O
N
H
BnO
OBn
O
OMe
OBn
22c
1.00
2.97
6.32
0.99
6.26
1.00
1.06
17.39
1.03
1.03
ppm

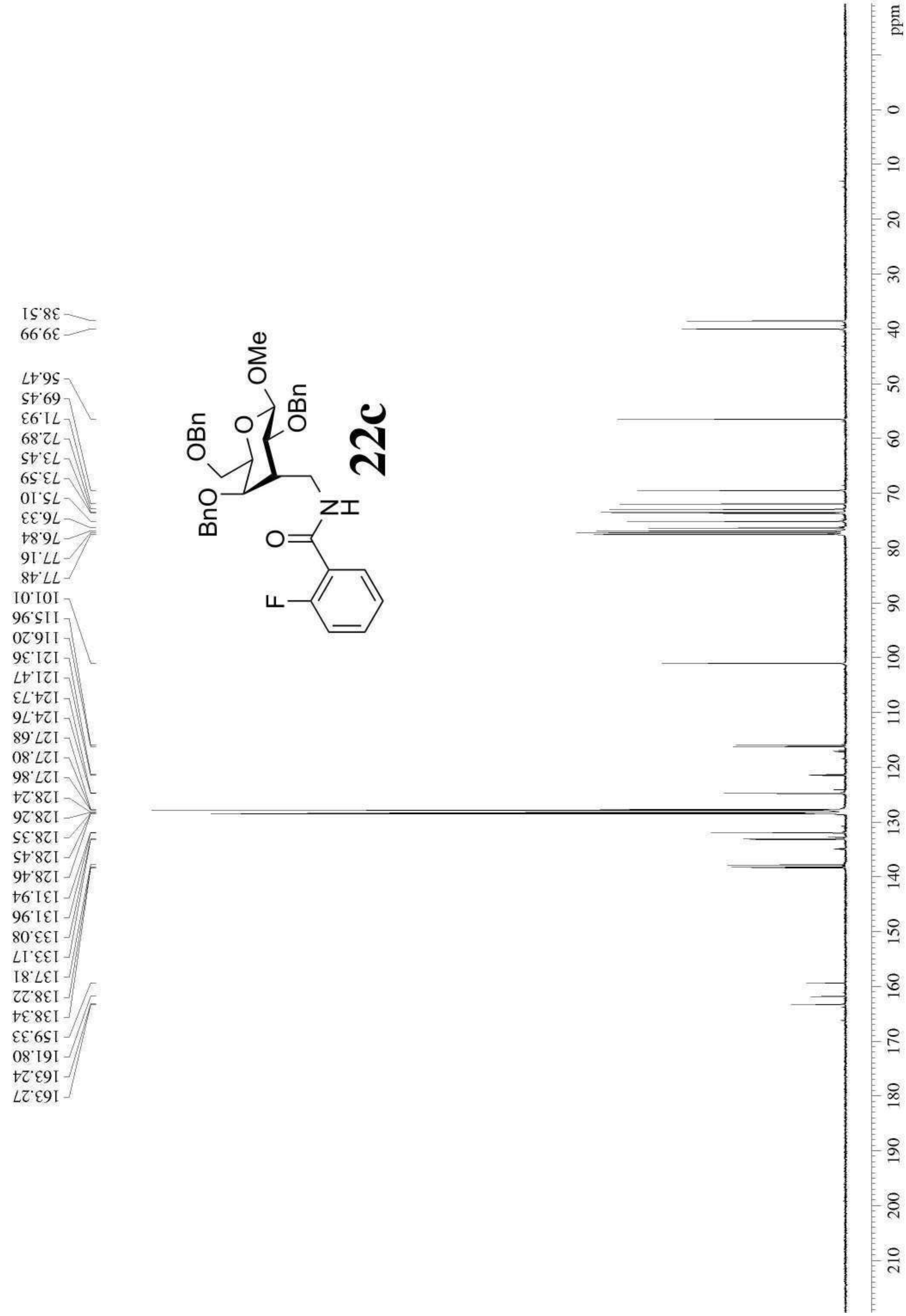
38.51
39.99
56.47
69.45
71.93
72.89
73.45
73.59
75.10
76.33
76.84
77.16
77.48
101.01
115.96
116.20
121.36
121.47
124.73
124.76
127.68
127.80
127.86
128.24
128.26
128.35
128.45
128.46
131.94
131.96
133.08
133.17
137.81
138.22
138.34
159.33
161.80
163.24
163.27
OBn
OMe
O
OBn
BnO
N
H
O
F
22c
ppm
0
10
20
30
40
50
60
70
80
90
100
110
120
130
140
150
160
170
180
190
200
210

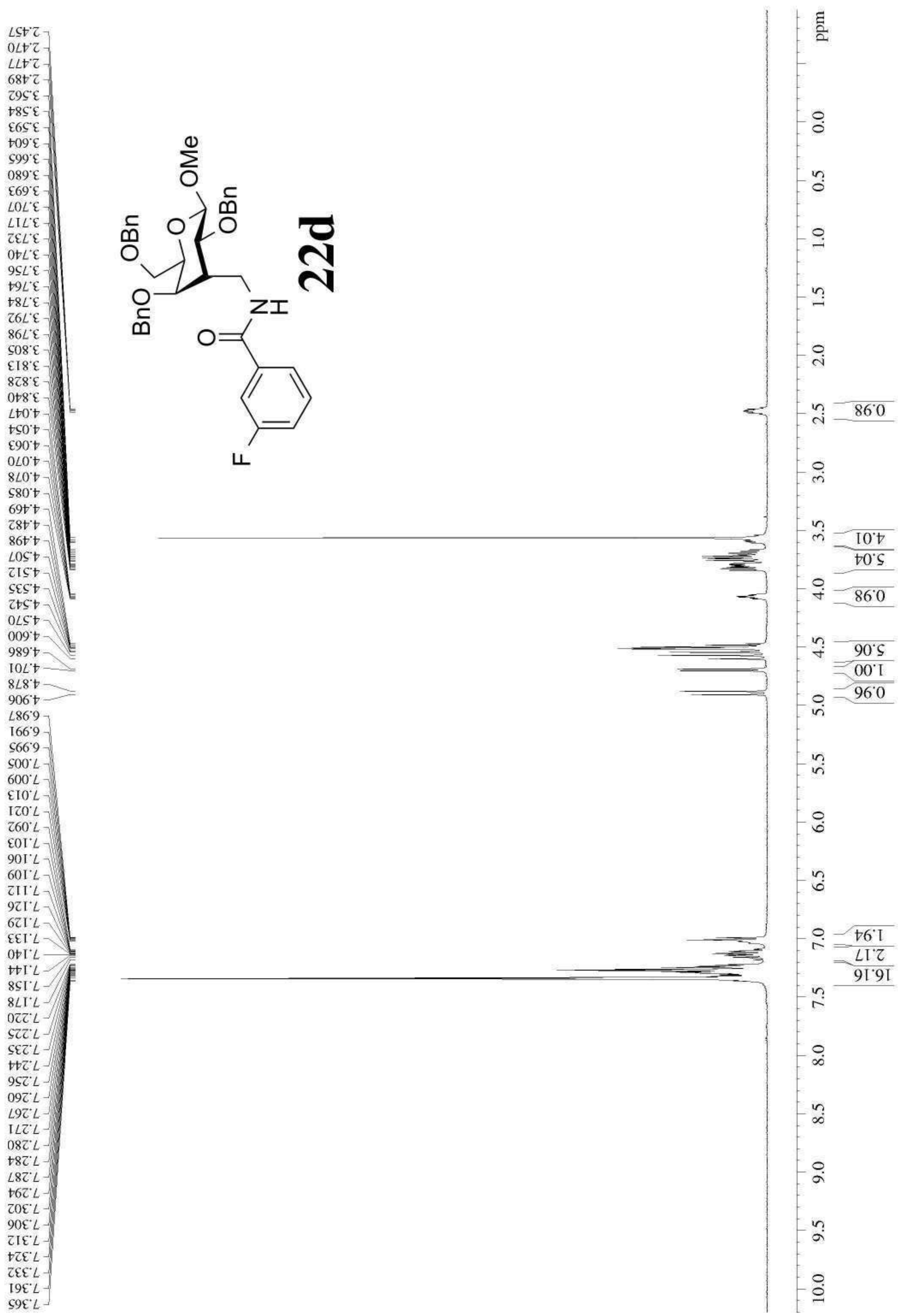

2.457
2.470
2.477
2.489
3.562
3.584
3.593
3.604
3.665
3.680
3.693
3.707
3.717
3.732
3.740
3.756
3.764
3.784
3.792
3.798
3.805
3.813
3.828
3.840
4.047
4.054
4.063
4.070
4.078
4.085
4.469
4.482
4.498
4.507
4.512
4.535
4.542
4.570
4.600
4.686
4.701
4.878
4.906
6.987
6.991
6.995
7.005
7.009
7.013
7.021
7.092
7.103
7.106
7.109
7.112
7.126
7.129
7.133
7.140
7.144
7.158
7.178
7.220
7.225
7.235
7.244
7.256
7.260
7.267
7.271
7.280
7.284
7.287
7.294
7.302
7.306
7.312
7.324
7.332
7.361
7.365
OBn
OMe
BnO
OBn
O
N
H
O
F
22d
0.98
4.01
5.04
0.98
5.06
1.00
0.96
1.94
2.17
16.16
ppm
0.0
0.5
1.0
1.5
2.0
2.5
3.0
3.5
4.0
4.5
5.0
5.5
6.0
6.5
7.0
7.5
8.0
8.5
9.0
9.5
10.0

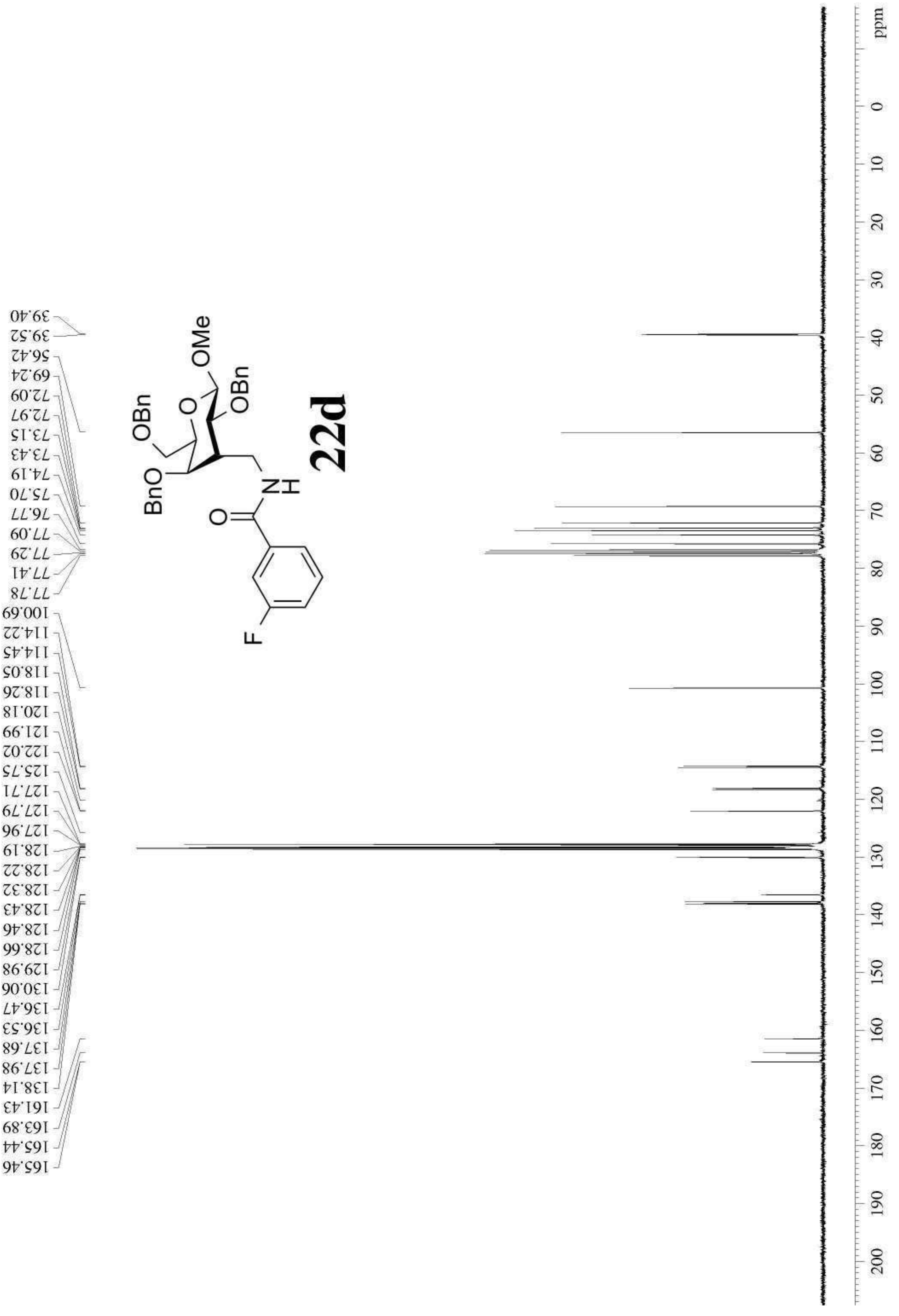
39.40
39.52
56.42
69.24
72.09
72.97
73.15
73.43
74.19
75.70
76.77
77.09
77.29
77.41
77.78
100.69
114.22
114.45
118.05
118.26
120.18
121.99
122.02
125.75
127.71
127.79
127.96
128.19
128.22
128.32
128.43
128.46
128.66
129.98
130.06
136.47
136.53
137.68
137.98
138.14
161.43
163.89
165.44
165.46
OBn
BnO
O
OMe
OBn
N
H
O
F
22d
ppm
0
10
20
30
40
50
60
70
80
90
100
110
120
130
140
150
160
170
180
190
200

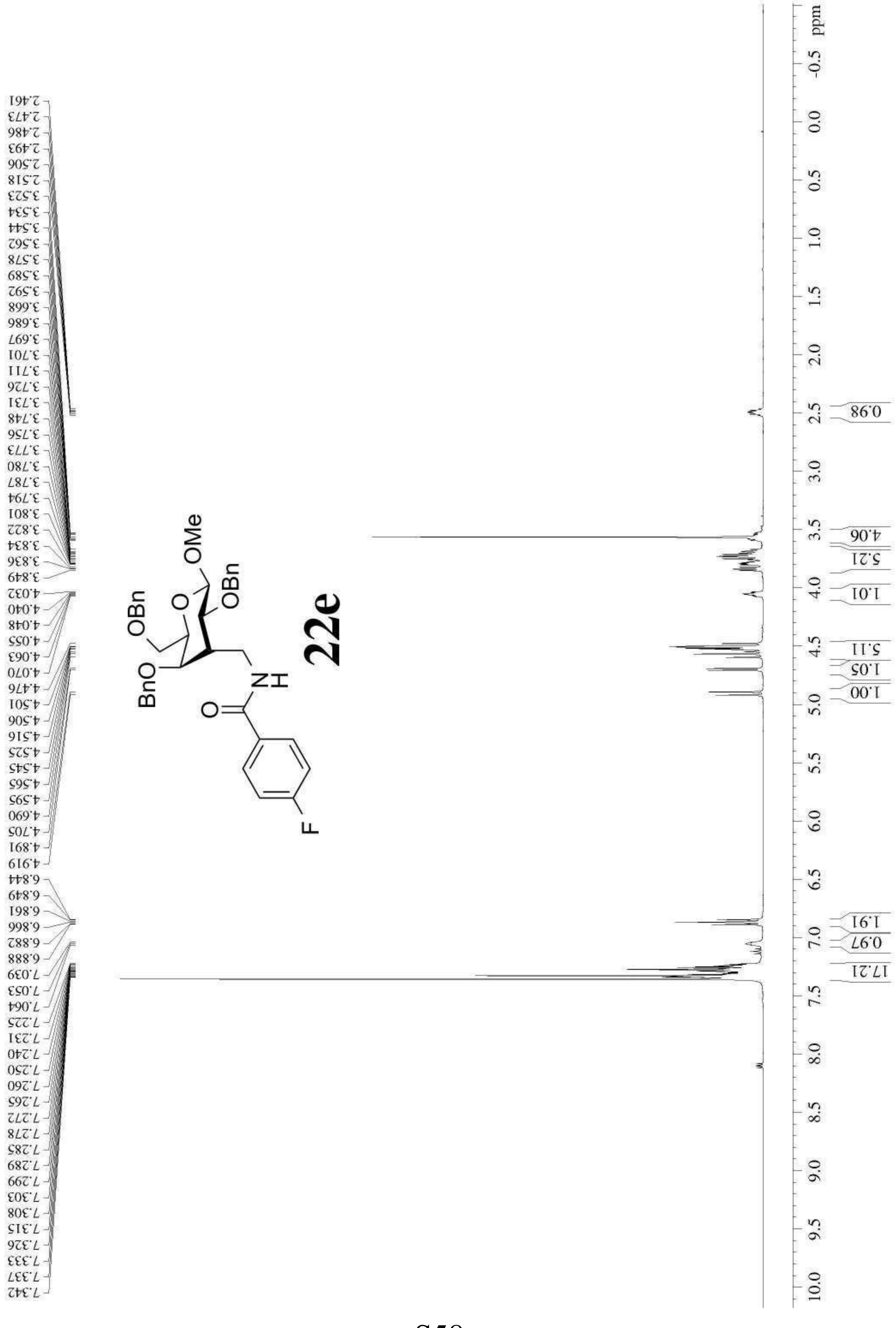
2.461
2.473
2.486
2.493
2.506
2.518
3.523
3.534
3.544
3.562
3.578
3.589
3.592
3.668
3.686
3.697
3.701
3.711
3.726
3.731
3.748
3.756
3.773
3.780
3.787
3.794
3.801
3.822
3.834
3.836
3.849
4.032
4.040
4.048
4.055
4.063
4.070
4.476
4.501
4.506
4.516
4.525
4.545
4.565
4.595
4.690
4.705
4.891
4.919
6.844
6.849
6.861
6.866
6.882
6.888
7.039
7.053
7.064
7.225
7.231
7.240
7.250
7.260
7.265
7.272
7.278
7.285
7.289
7.299
7.303
7.308
7.315
7.326
7.333
7.337
7.342
OBn
BnO
O
OMe
OBn
N
H
O
F
22e
0.98
4.06
5.21
1.01
5.11
1.05
1.00
1.91
0.97
17.21
ppm
-0.5
0.0
0.5
1.0
1.5
2.0
2.5
3.0
3.5
4.0
4.5
5.0
5.5
6.0
6.5
7.0
7.5
8.0
8.5
9.0
9.5
10.0

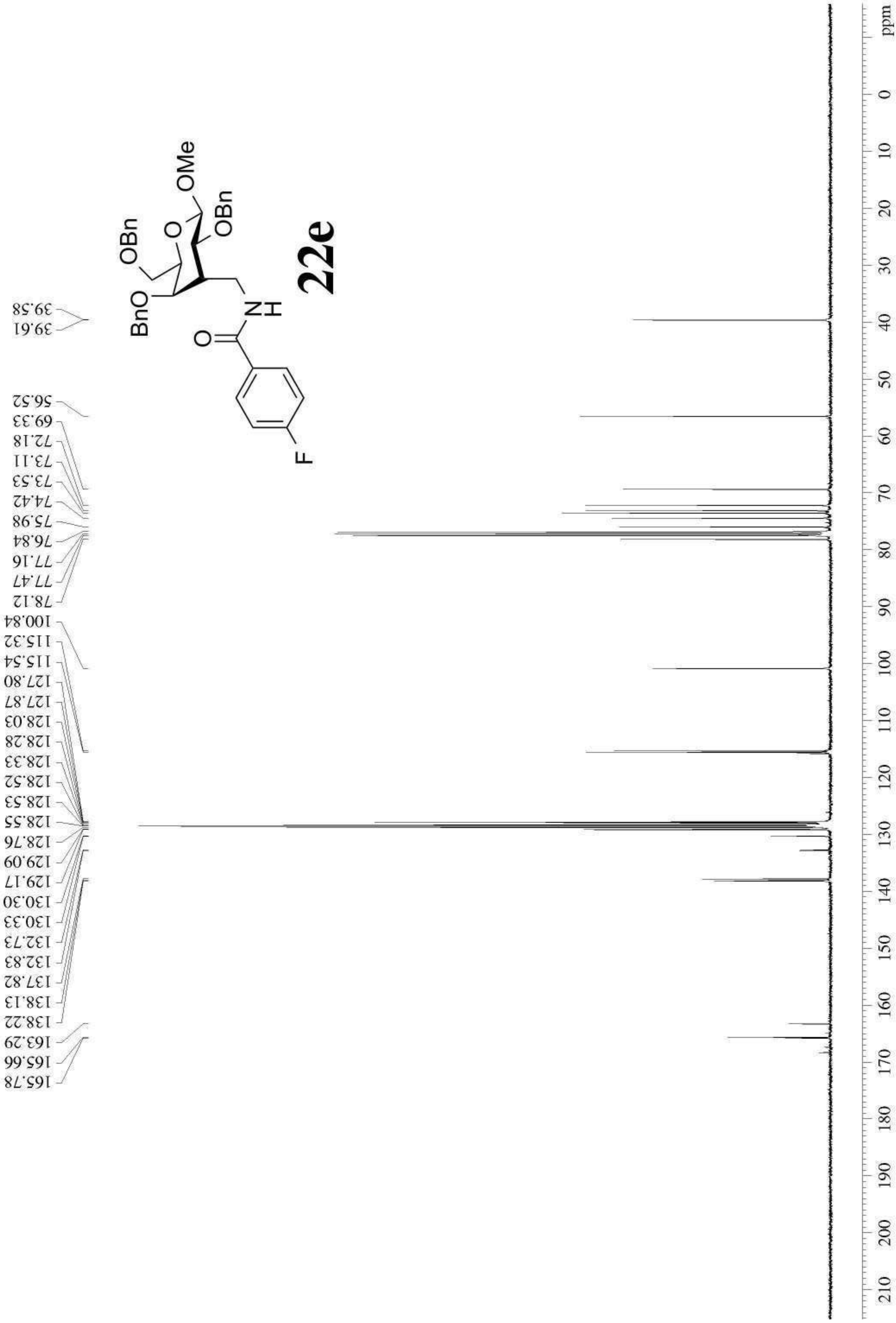
39.58
39.61
56.52
69.33
72.18
73.11
73.53
74.42
75.98
76.84
77.16
77.47
78.12
100.84
115.32
115.54
127.80
127.87
128.03
128.28
128.33
128.52
128.53
128.55
128.76
129.09
129.17
130.30
130.33
132.73
132.83
137.82
138.13
138.22
163.29
165.66
165.78
BnO
OBn
O
OMe
OBn
N
H
O
F
22e
ppm
0
10
20
30
40
50
60
70
80
90
100
110
120
130
140
150
160
170
180
190
200
210

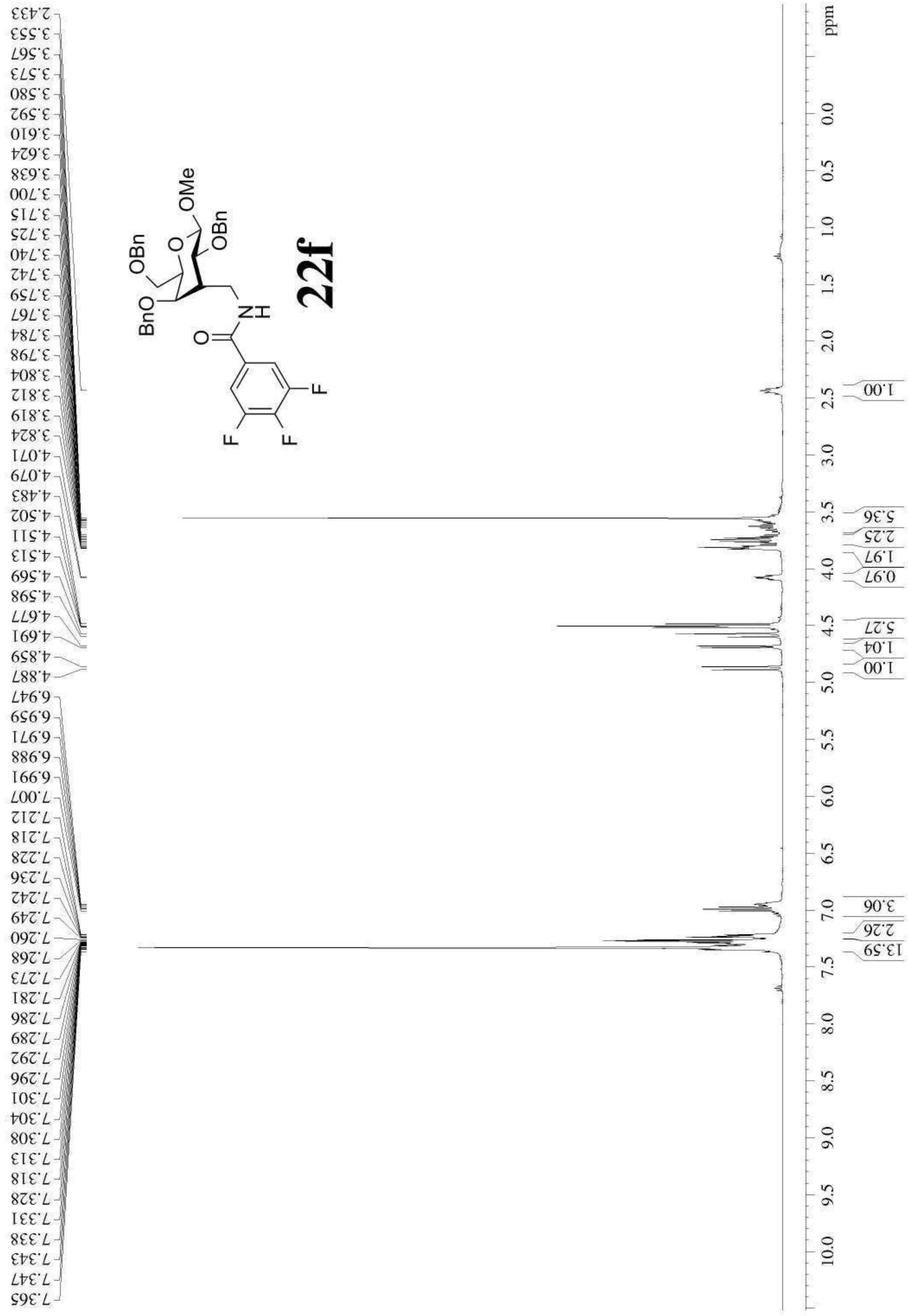
22f
ppm

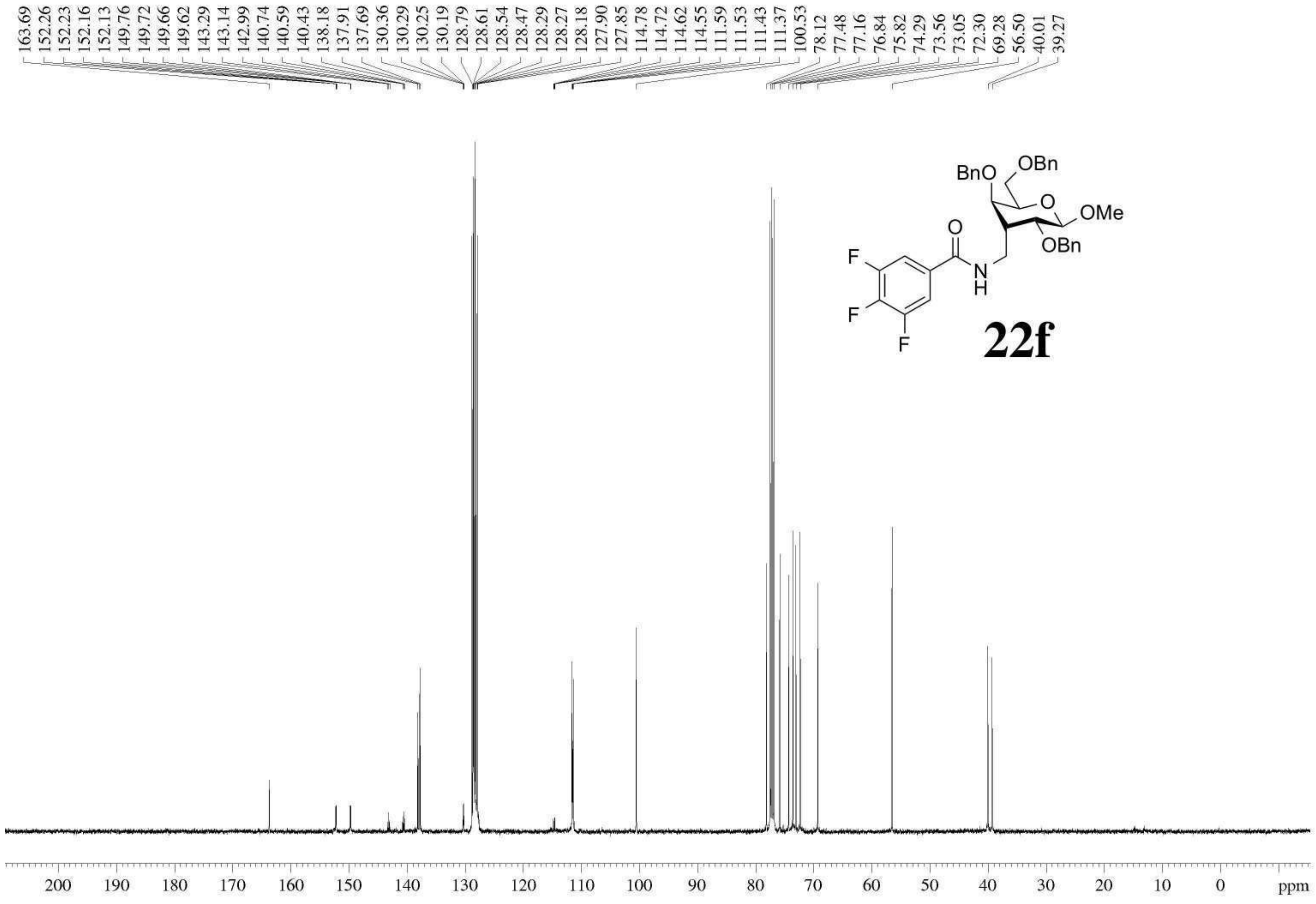
163.69
152.26
152.23
152.16
152.13
149.76
149.72
149.66
149.62
143.29
143.14
142.99
140.74
140.59
140.43
138.18
137.91
137.69
130.36
130.29
130.25
130.19
128.79
128.61
128.54
128.47
128.29
128.27
128.18
127.90
127.85
114.78
114.72
114.62
114.55
111.59
111.53
111.43
111.37
100.53
78.12
77.48
77.16
76.84
75.82
74.29
73.56
73.05
72.30
69.28
56.50
40.01
39.27
BnO
OBn
O
OMe
OBn
O
N
H
F
F
F
22f
200
190
180
170
160
150
140
130
120
110
100
90
80
70
60
50
40
30
20
10
0
ppm

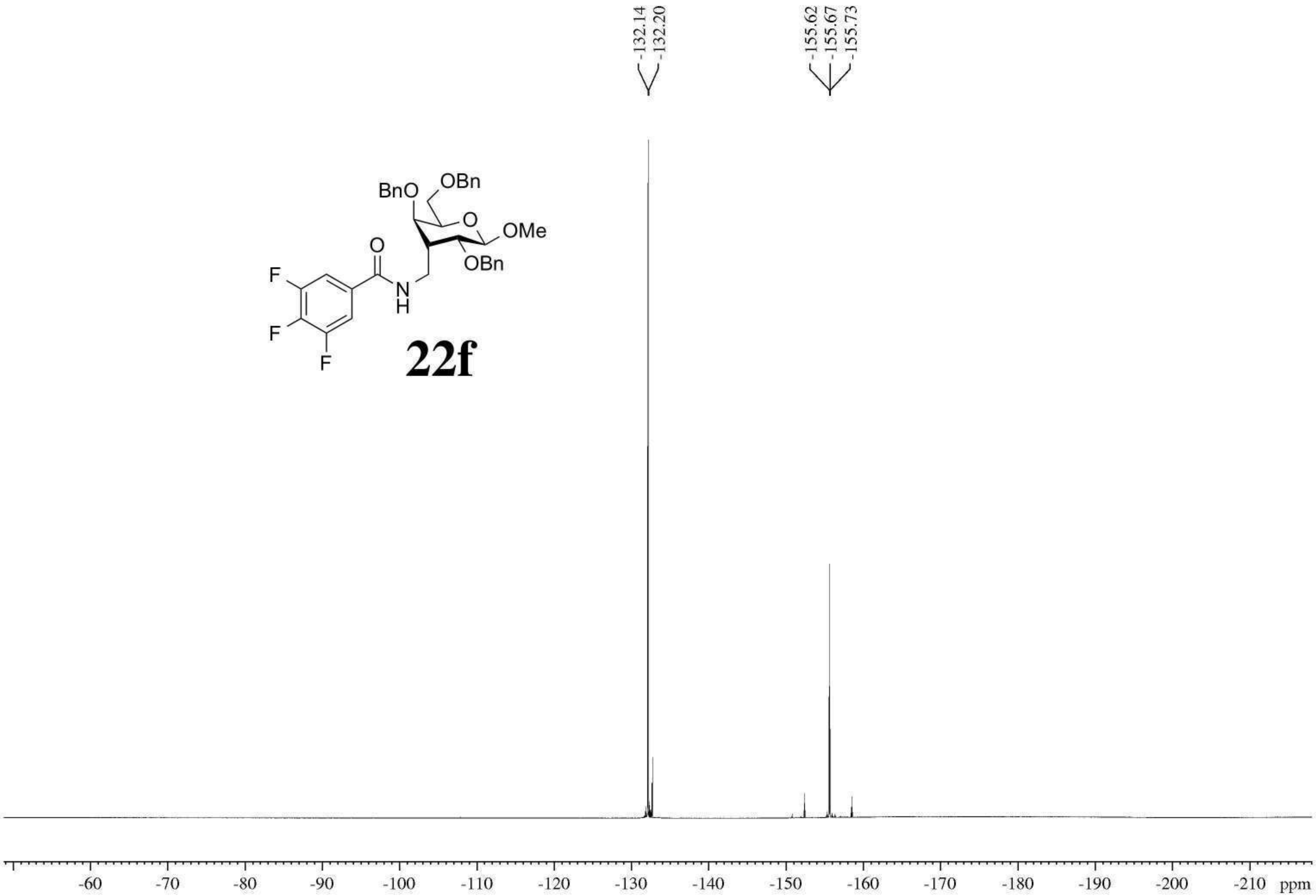
BnO
OBn
O
OMe
OBn
O
N
H
F
F
F
22f
-132.14
-132.20
-155.62
-155.67
-155.73
-60
-70
-80
-90
-100
-110
-120
-130
-140
-150
-160
-170
-180
-190
-200
-210
ppm

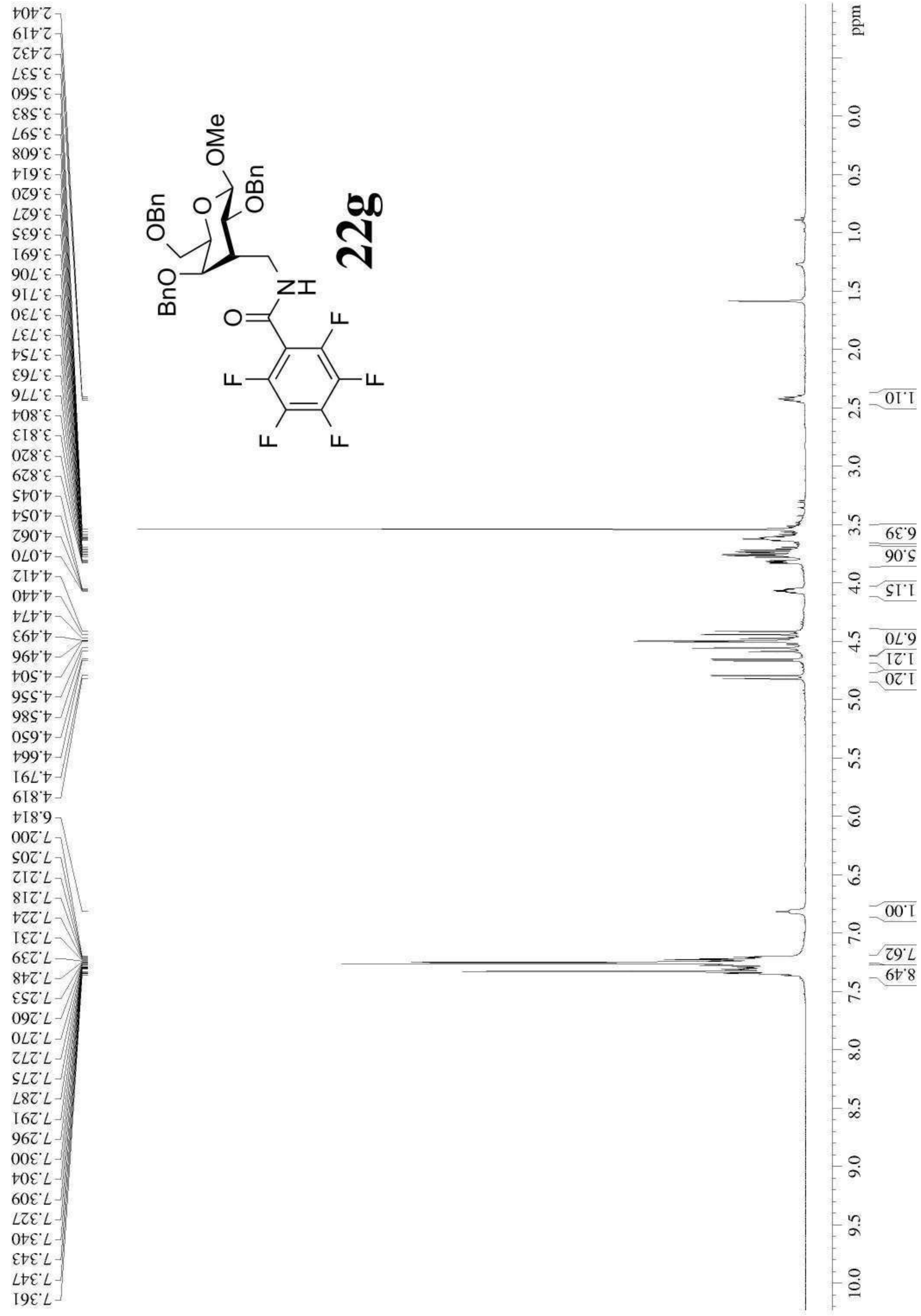
2.404
2.419
2.432
3.537
3.560
3.583
3.597
3.608
3.614
3.620
3.627
3.635
3.691
3.706
3.716
3.730
3.737
3.754
3.763
3.776
3.804
3.813
3.820
3.829
4.045
4.054
4.062
4.070
4.412
4.440
4.474
4.493
4.496
4.504
4.556
4.586
4.650
4.664
4.791
4.819
6.814
7.200
7.205
7.212
7.218
7.224
7.231
7.239
7.248
7.253
7.260
7.270
7.272
7.275
7.287
7.291
7.296
7.300
7.304
7.309
7.327
7.340
7.343
7.347
7.361
BnO
OBn
O
OMe
OBn
O
N
H
F
F
F
F
F
22g
1.10
6.39
5.06
1.15
6.70
1.21
1.20
1.00
7.62
8.49
ppm
0.0
0.5
1.0
1.5
2.0
2.5
3.0
3.5
4.0
4.5
5.0
5.5
6.0
6.5
7.0
7.5
8.0
8.5
9.0
9.5
10.0

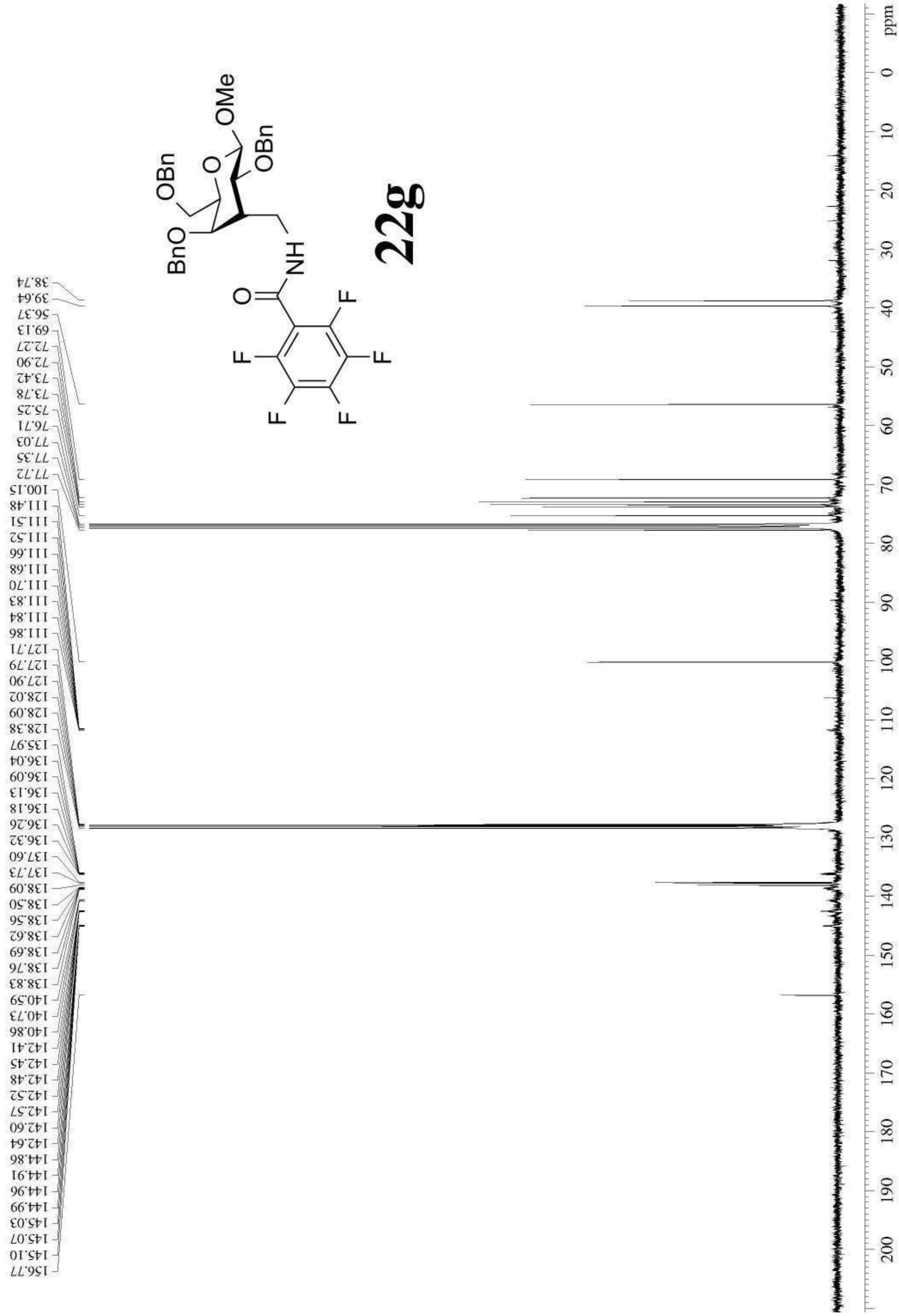
38.74
39.64
56.37
69.13
72.27
72.90
73.42
73.78
75.25
76.71
77.03
77.35
77.72
100.15
111.48
111.51
111.52
111.66
111.68
111.70
111.83
111.84
111.86
127.71
127.79
127.90
128.02
128.09
128.38
135.97
136.04
136.09
136.13
136.18
136.26
136.32
137.60
137.73
138.09
138.50
138.56
138.62
138.69
138.76
138.83
140.59
140.73
140.86
142.41
142.45
142.48
142.52
142.57
142.60
142.64
144.86
144.91
144.96
144.99
145.03
145.07
145.10
156.77
BnO
OBn
O
OMe
OBn
NH
O
F
F
F
F
F
ppm

22g

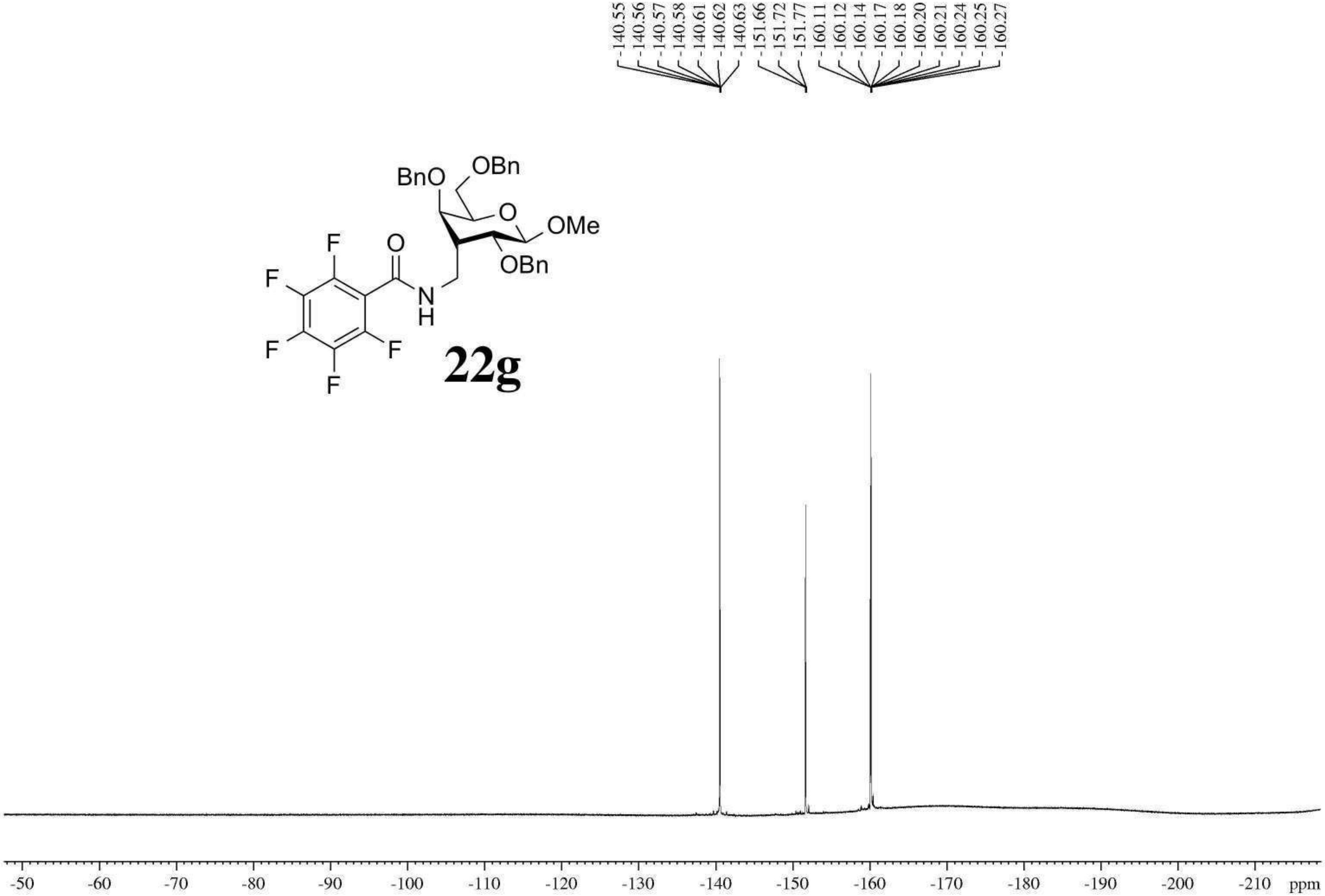
-140.55
-140.56
-140.57
-140.58
-140.61
-140.62
-140.63
-151.66
-151.72
-151.77
-160.11
-160.12
-160.14
-160.17
-160.18
-160.20
-160.21
-160.24
-160.25
-160.27
BnO
OBn
O
OMe
OBn
F
O
N
H
F
F
F
F
22g
-50
-60
-70
-80
-90
-100
-110
-120
-130
-140
-150
-160
-170
-180
-190
-200
-210
ppm

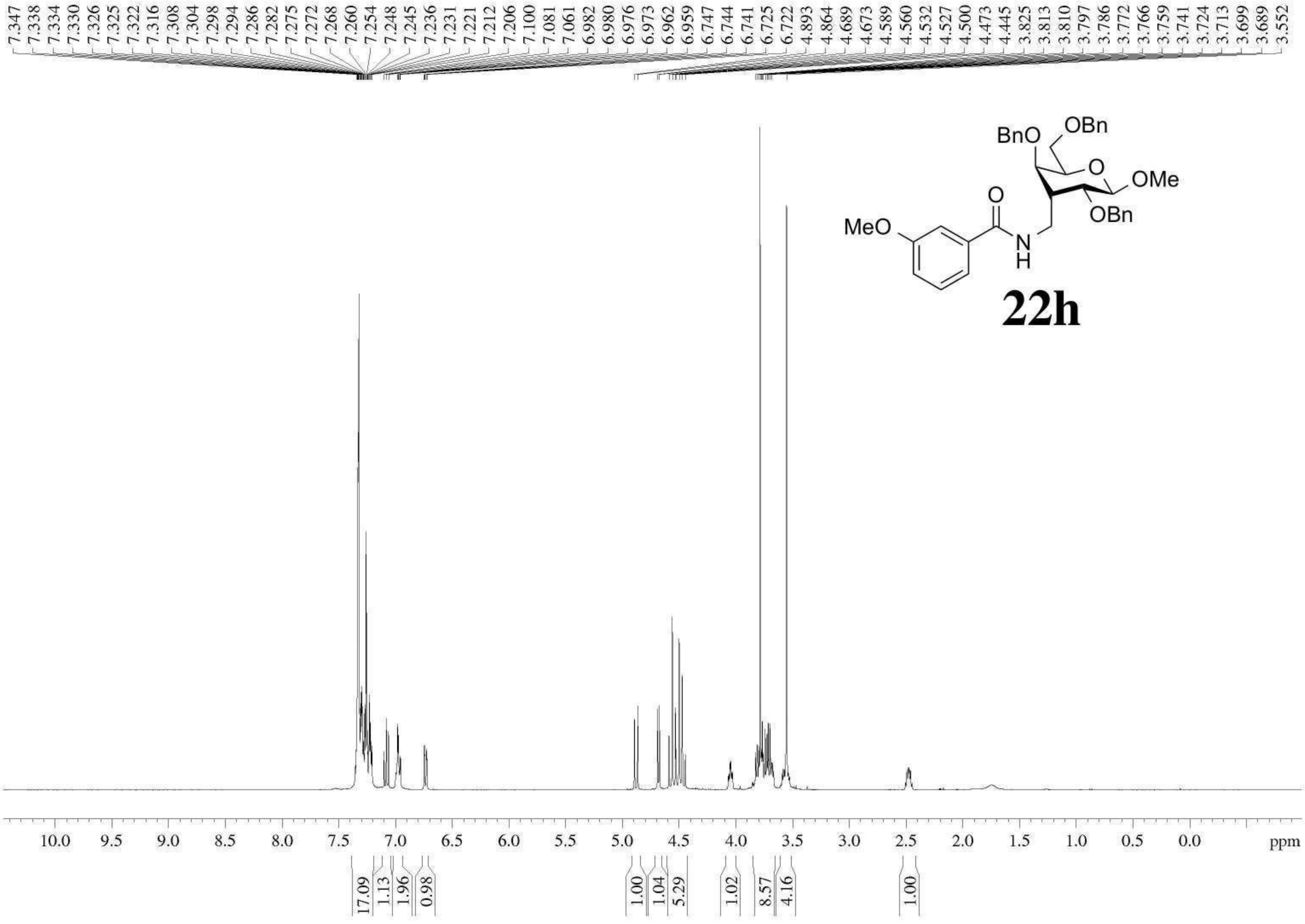
BnO
OBn
O
OMe
O
MeO
N
H
OBn
7.347
7.338
7.334
7.330
7.326
7.325
7.322
7.316
7.308
7.304
7.298
7.294
7.286
7.282
7.275
7.272
7.268
7.260
7.254
7.248
7.245
7.236
7.231
7.221
7.212
7.206
7.100
7.081
7.061
6.982
6.980
6.976
6.973
6.962
6.959
6.747
6.744
6.741
6.725
6.722
4.893
4.864
4.689
4.673
4.589
4.560
4.532
4.527
4.500
4.473
4.445
3.825
3.813
3.810
3.797
3.786
3.772
3.766
3.759
3.741
3.724
3.713
3.699
3.689
3.552
10.0
9.5
9.0
8.5
8.0
7.5
7.0
6.5
6.0
5.5
5.0
4.5
4.0
3.5
3.0
2.5
2.0
1.5
1.0
0.5
0.0
ppm
17.09
1.13
1.96
0.98
1.00
1.04
5.29
1.02
8.57
4.16
1.00

22h

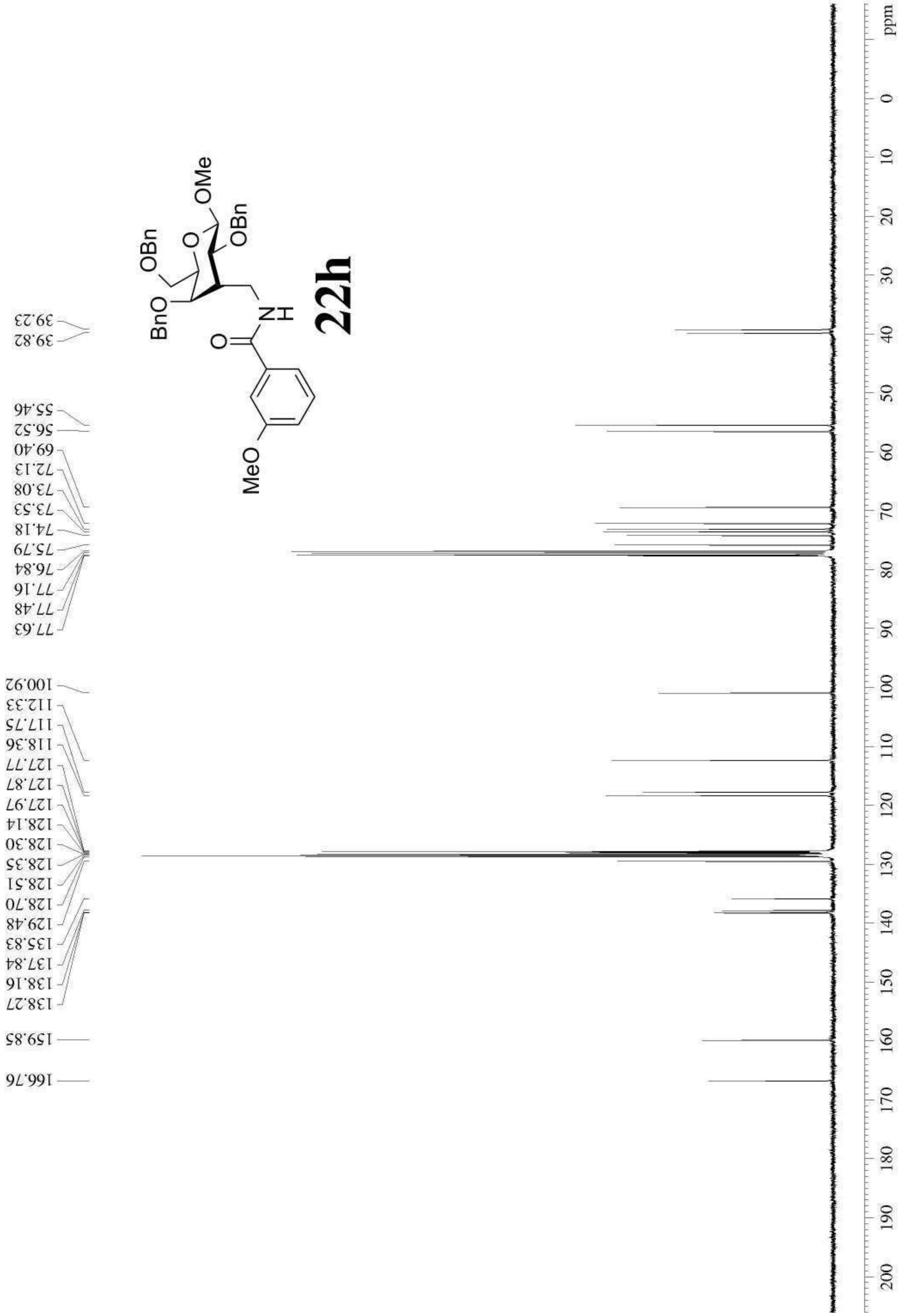

39.23
39.82
55.46
56.52
69.40
72.13
73.08
73.53
74.18
75.79
76.84
77.16
77.48
77.63
100.92
112.33
117.75
118.36
127.77
127.87
127.97
128.14
128.30
128.35
128.51
128.70
129.48
135.83
137.84
138.16
138.27
159.85
166.76
OBn
OMe
BnO
OBn
O
N
H
MeO
22h
ppm

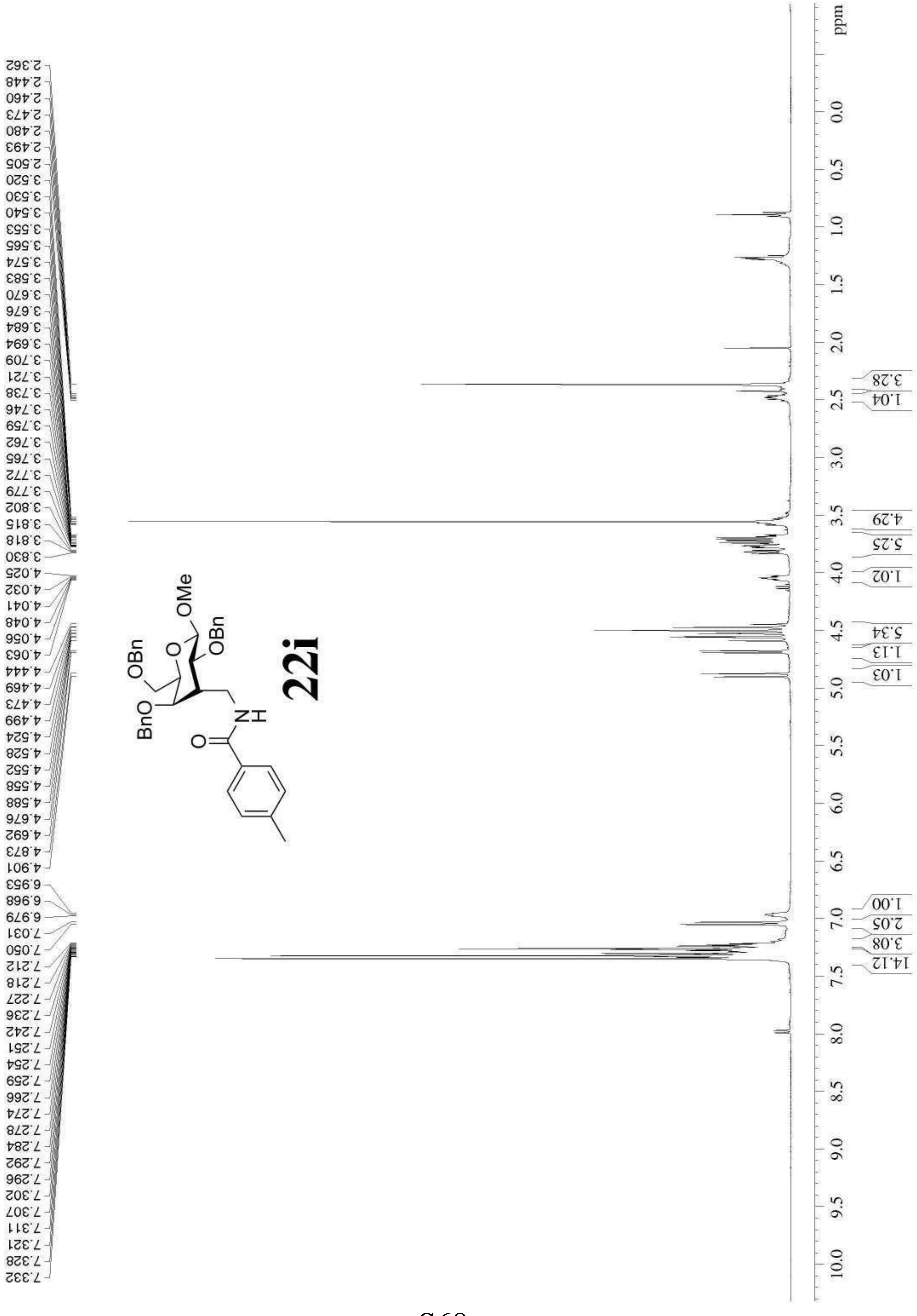
2.362
2.448
2.460
2.473
2.480
2.493
2.505
3.520
3.530
3.540
3.553
3.565
3.574
3.583
3.670
3.676
3.684
3.694
3.709
3.721
3.738
3.746
3.759
3.762
3.765
3.772
3.779
3.802
3.815
3.818
3.830
4.025
4.032
4.041
4.048
4.056
4.063
4.444
4.469
4.473
4.499
4.524
4.528
4.552
4.558
4.588
4.676
4.692
4.873
4.901
6.953
6.968
6.979
7.031
7.050
7.212
7.218
7.227
7.236
7.242
7.251
7.254
7.259
7.266
7.274
7.278
7.284
7.292
7.296
7.302
7.307
7.311
7.321
7.328
7.332
BnO
OBn
O
OMe
OBn
O
N
H
22i
3.28
1.04
4.29
5.25
1.02
5.34
1.13
1.03
1.00
2.05
3.08
14.12
ppm
0.0
0.5
1.0
1.5
2.0
2.5
3.0
3.5
4.0
4.5
5.0
5.5
6.0
6.5
7.0
7.5
8.0
8.5
9.0
9.5
10.0

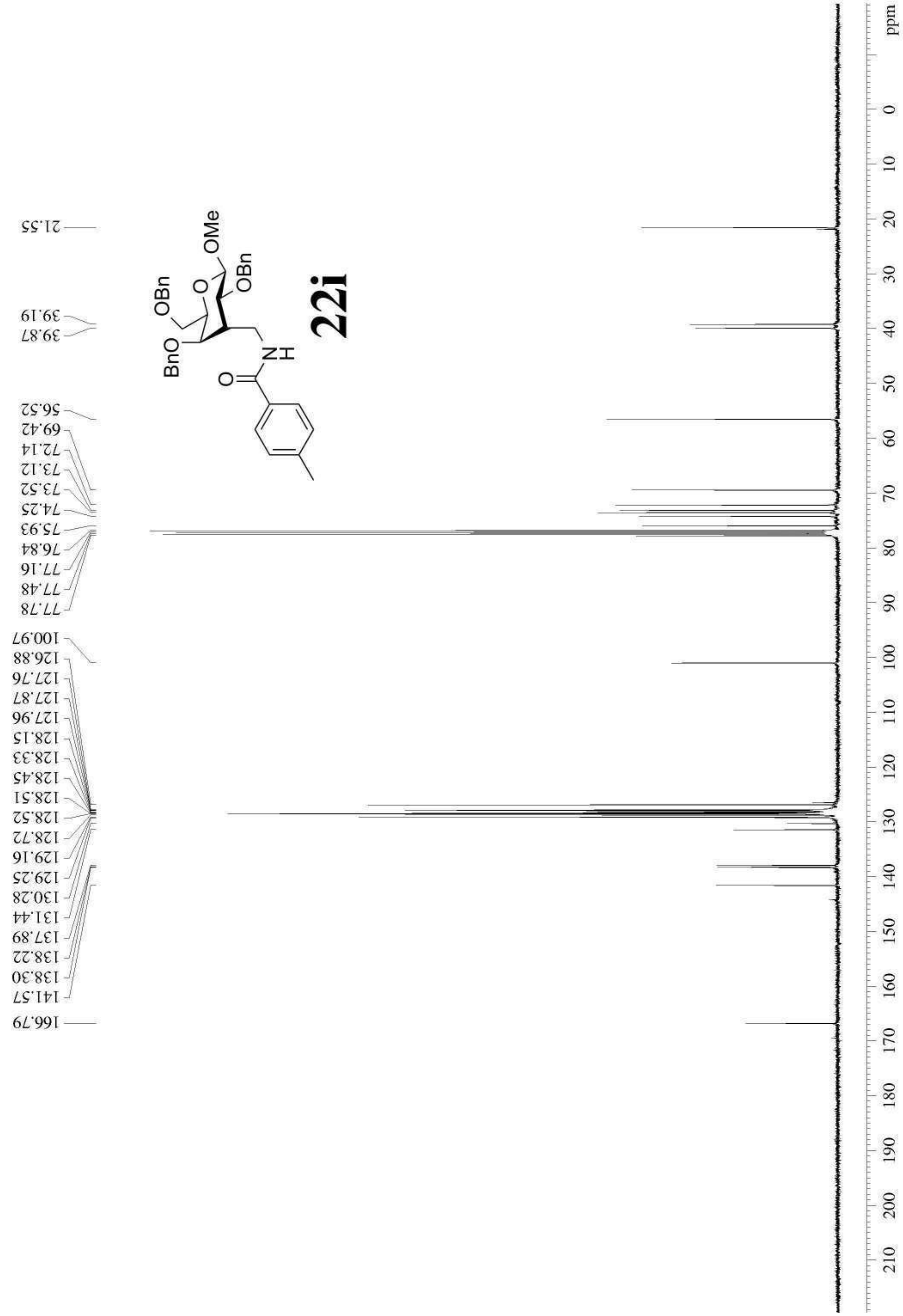
21.55
39.19
39.87
56.52
69.42
72.14
73.12
73.52
74.25
75.93
76.84
77.16
77.48
77.78
100.97
126.88
127.76
127.87
127.96
128.15
128.33
128.45
128.51
128.52
128.72
129.16
129.25
130.28
131.44
137.89
138.22
138.30
141.57
166.79
BnO
OBn
O
OMe
OBn
N
H
O
22i
ppm

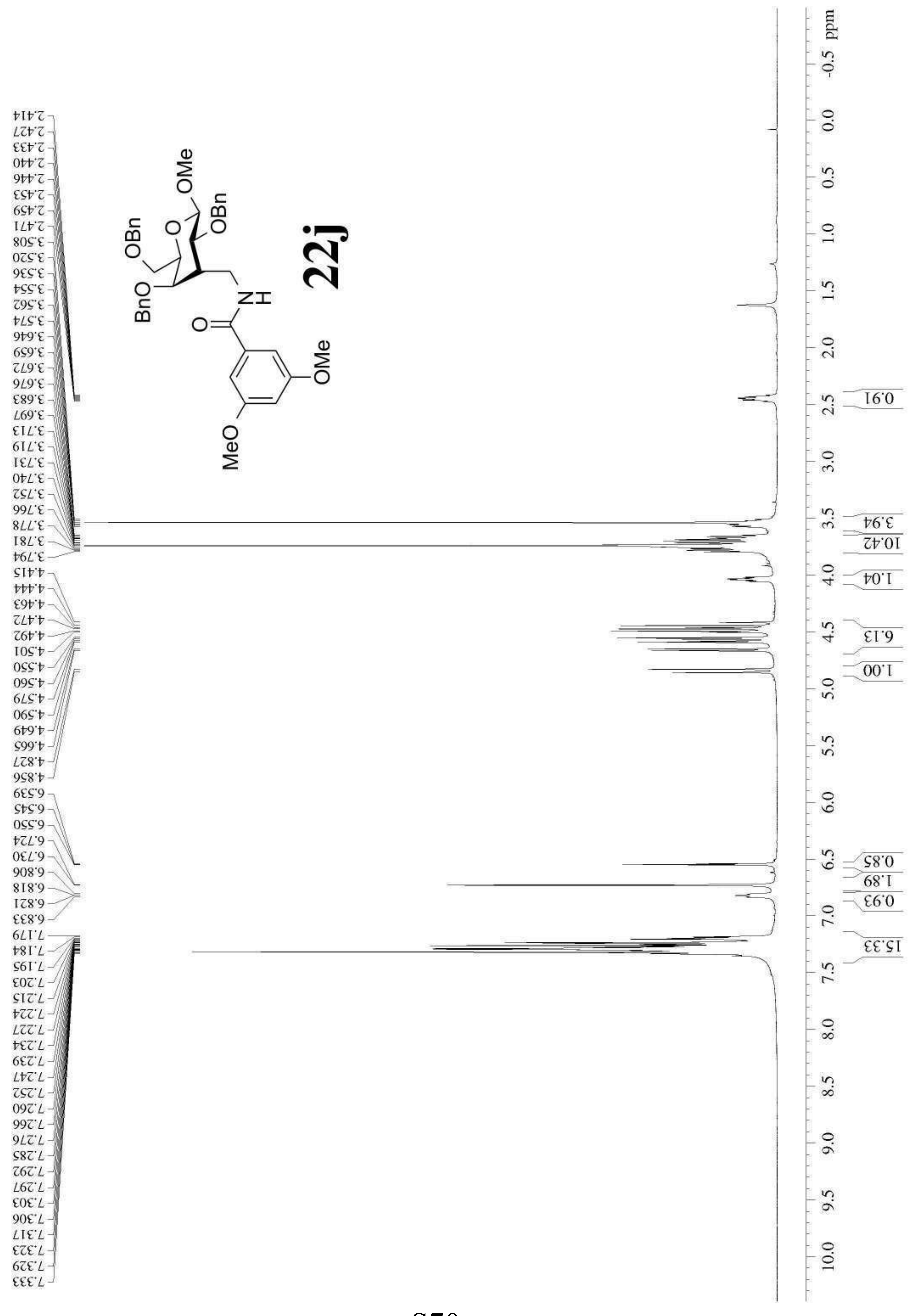
22j

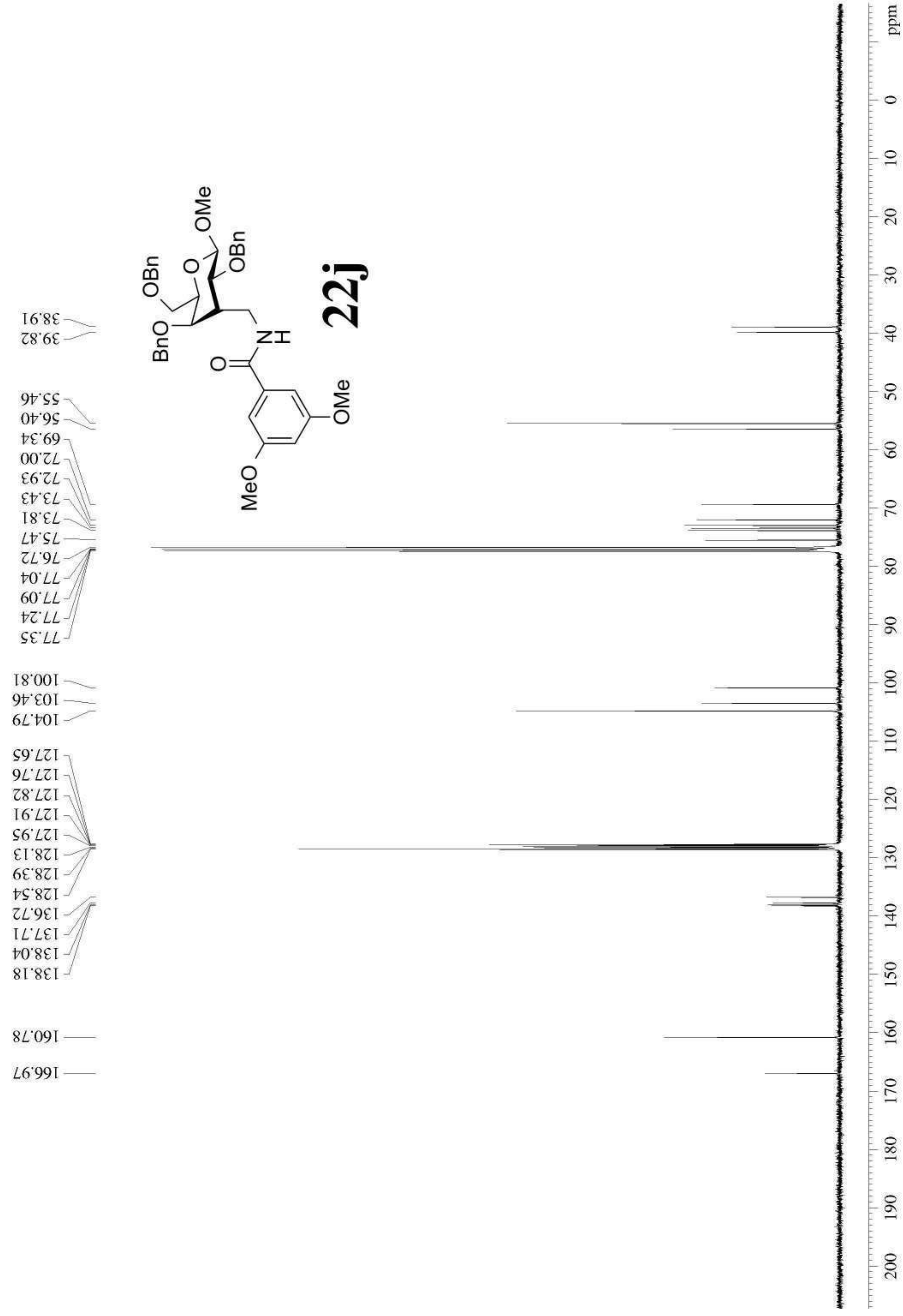
38.91
39.82
55.46
56.40
69.34
72.00
72.93
73.43
73.81
75.47
76.72
77.04
77.09
77.24
77.35
100.81
103.46
104.79
127.65
127.76
127.82
127.91
127.95
128.13
128.39
128.54
136.72
137.71
138.04
138.18
160.78
166.97
BnO
OBn
O
OMe
OBn
N
H
O
OMe
MeO
ppm
0
10
20
30
40
50
60
70
80
90
100
110
120
130
140
150
160
170
180
190
200

22j

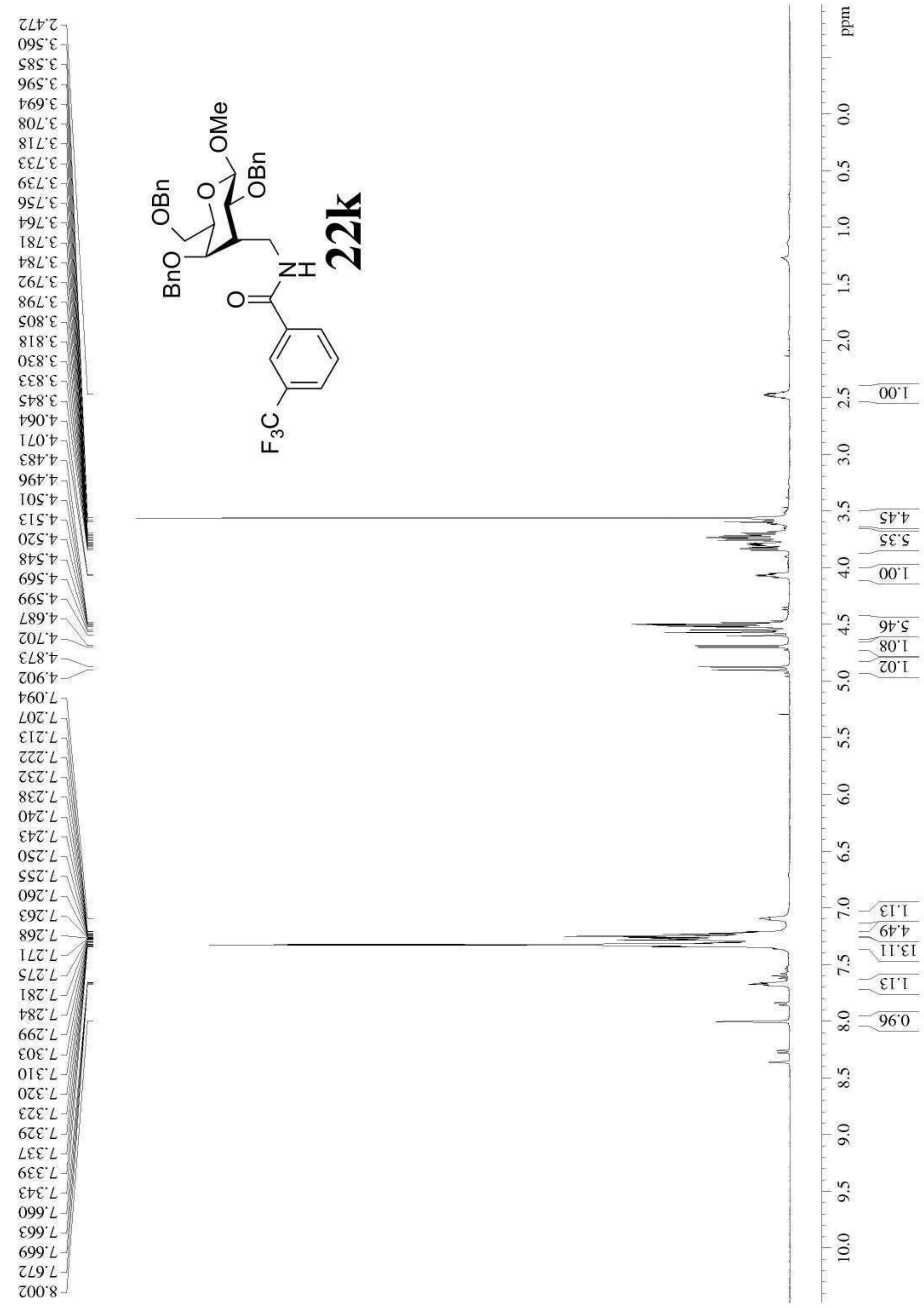
2.472
3.560
3.585
3.596
3.694
3.708
3.718
3.733
3.739
3.756
3.764
3.781
3.784
3.792
3.798
3.805
3.818
3.830
3.833
3.845
4.064
4.071
4.483
4.496
4.501
4.513
4.520
4.548
4.569
4.599
4.687
4.702
4.873
4.902
7.094
7.207
7.213
7.222
7.232
7.238
7.240
7.243
7.250
7.255
7.260
7.263
7.268
7.271
7.275
7.281
7.284
7.299
7.303
7.310
7.320
7.323
7.329
7.337
7.339
7.343
7.660
7.663
7.669
7.672
8.002
BnO
OBn
O
OMe
OBn
O
N
H
F3C
22k
1.00
4.45
5.35
1.00
5.46
1.08
1.02
1.13
4.49
13.11
1.13
0.96
ppm

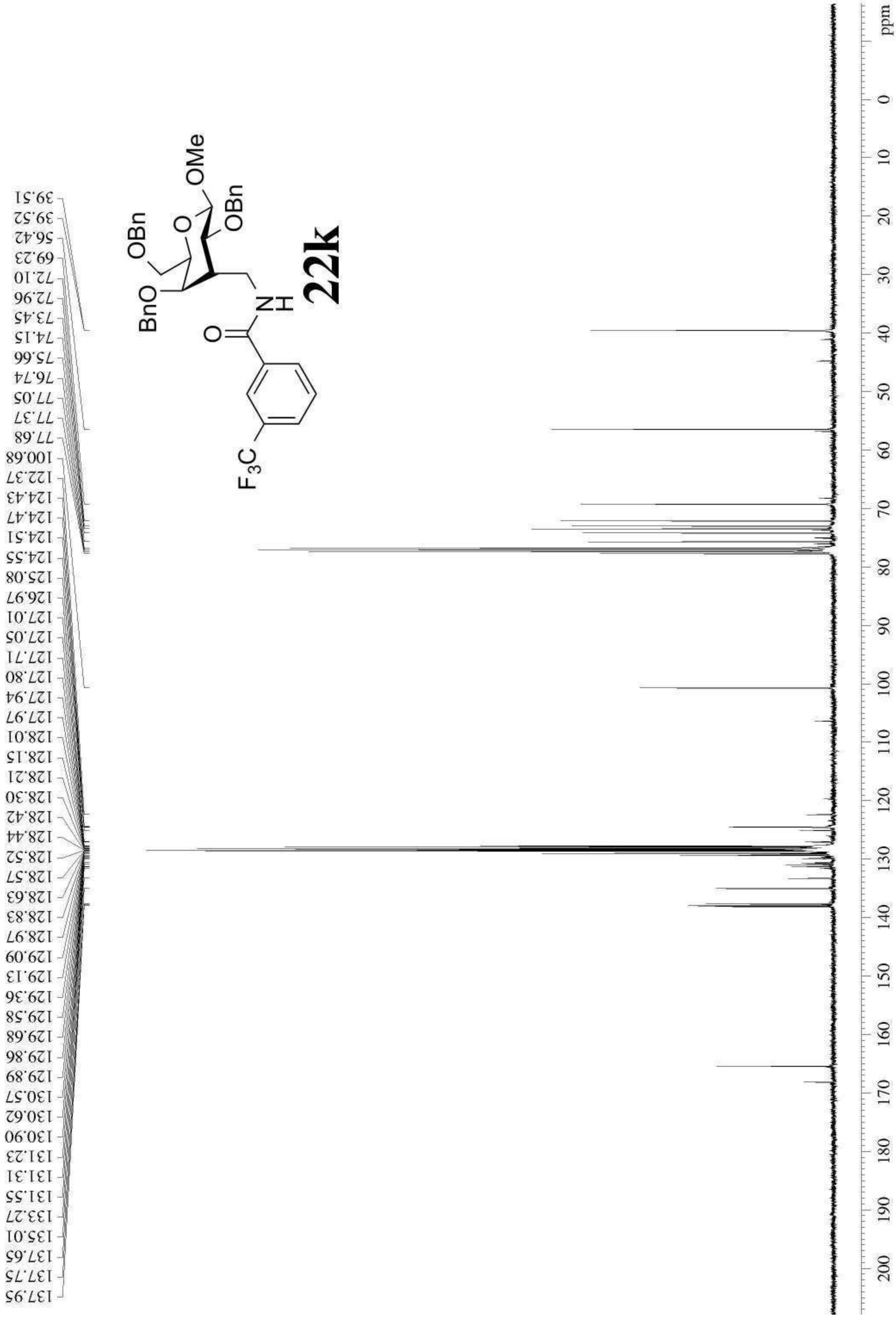
39.51
39.52
56.42
69.23
72.10
72.96
73.45
74.15
75.66
76.74
77.05
77.37
77.68
100.68
122.37
124.43
124.47
124.51
124.55
125.08
126.97
127.01
127.05
127.71
127.80
127.94
127.97
128.01
128.15
128.21
128.30
128.42
128.44
128.52
128.57
128.63
128.83
128.97
129.09
129.13
129.36
129.58
129.68
129.86
129.89
130.57
130.62
130.90
131.23
131.31
131.55
133.27
135.01
137.65
137.75
137.95
OMe
OBn
OBn
BnO
O
N
H
O
F_3C
22k
ppm

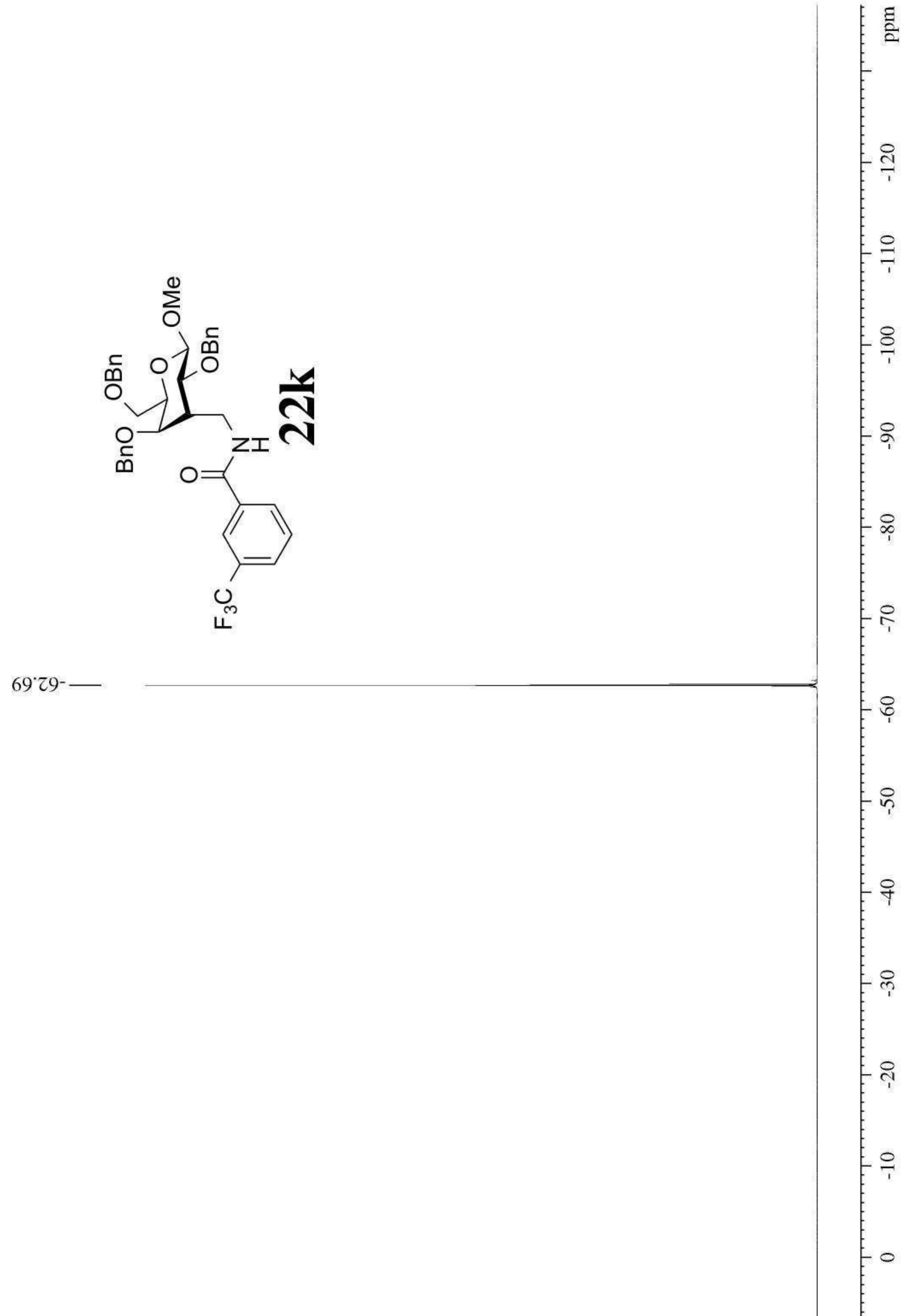

22k
-62.69
ppm
-120
-110
-100
-90
-80
-70
-60
-50
-40
-30
-20
-10
0

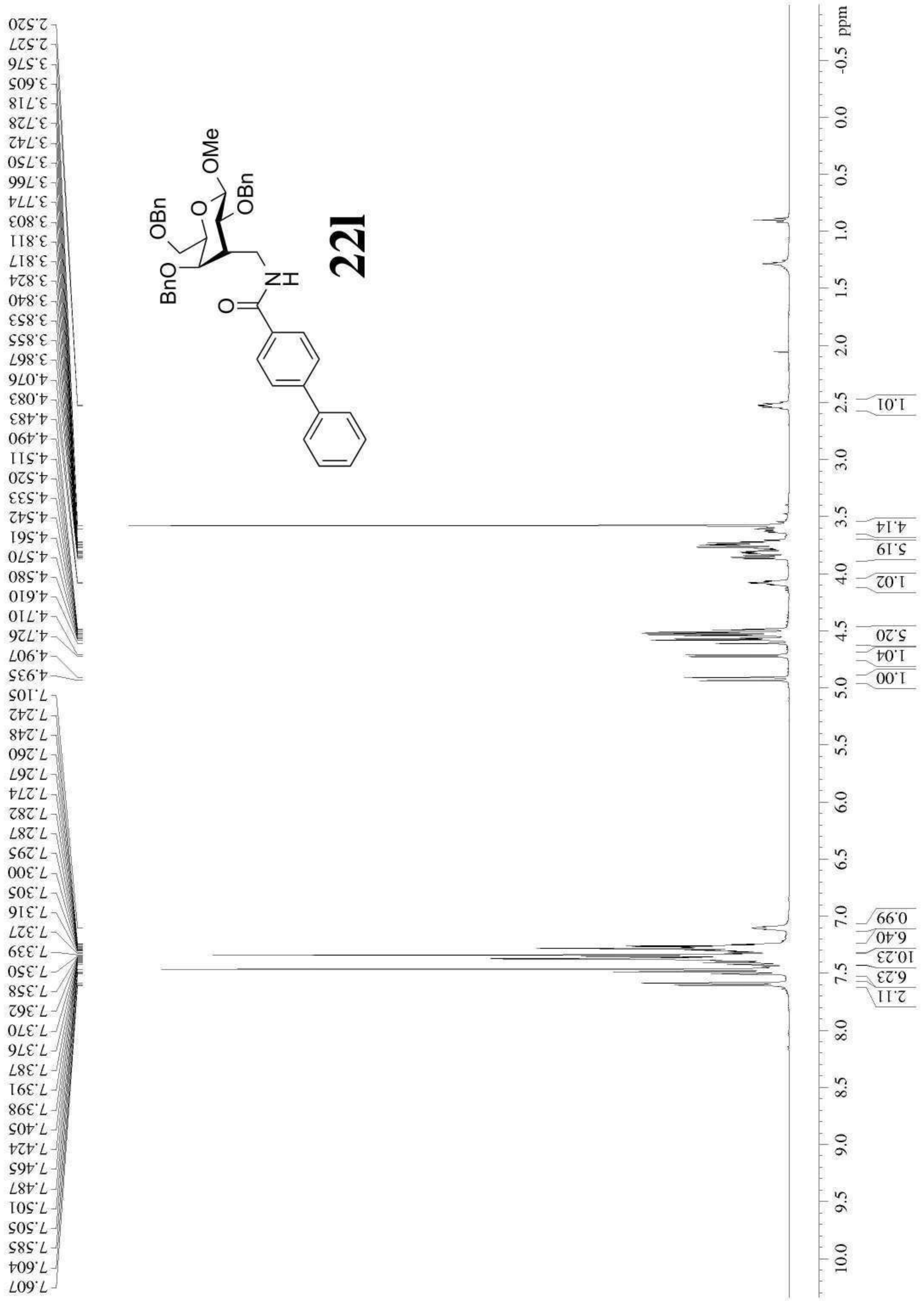
2.520
2.527
3.576
3.605
3.718
3.728
3.742
3.750
3.766
3.774
3.803
3.811
3.817
3.824
3.840
3.853
3.855
3.867
4.076
4.083
4.483
4.490
4.511
4.520
4.533
4.542
4.561
4.570
4.580
4.610
4.710
4.726
4.907
4.935
7.105
7.242
7.248
7.260
7.267
7.274
7.282
7.287
7.295
7.300
7.305
7.316
7.327
7.339
7.350
7.358
7.362
7.370
7.376
7.387
7.391
7.398
7.405
7.424
7.465
7.487
7.501
7.505
7.585
7.604
7.607
BnO
OBn
O
OMe
OBn
O
N
H
22l
1.01
4.14
5.19
1.02
5.20
1.04
1.00
0.99
6.40
10.23
6.23
2.11
ppm

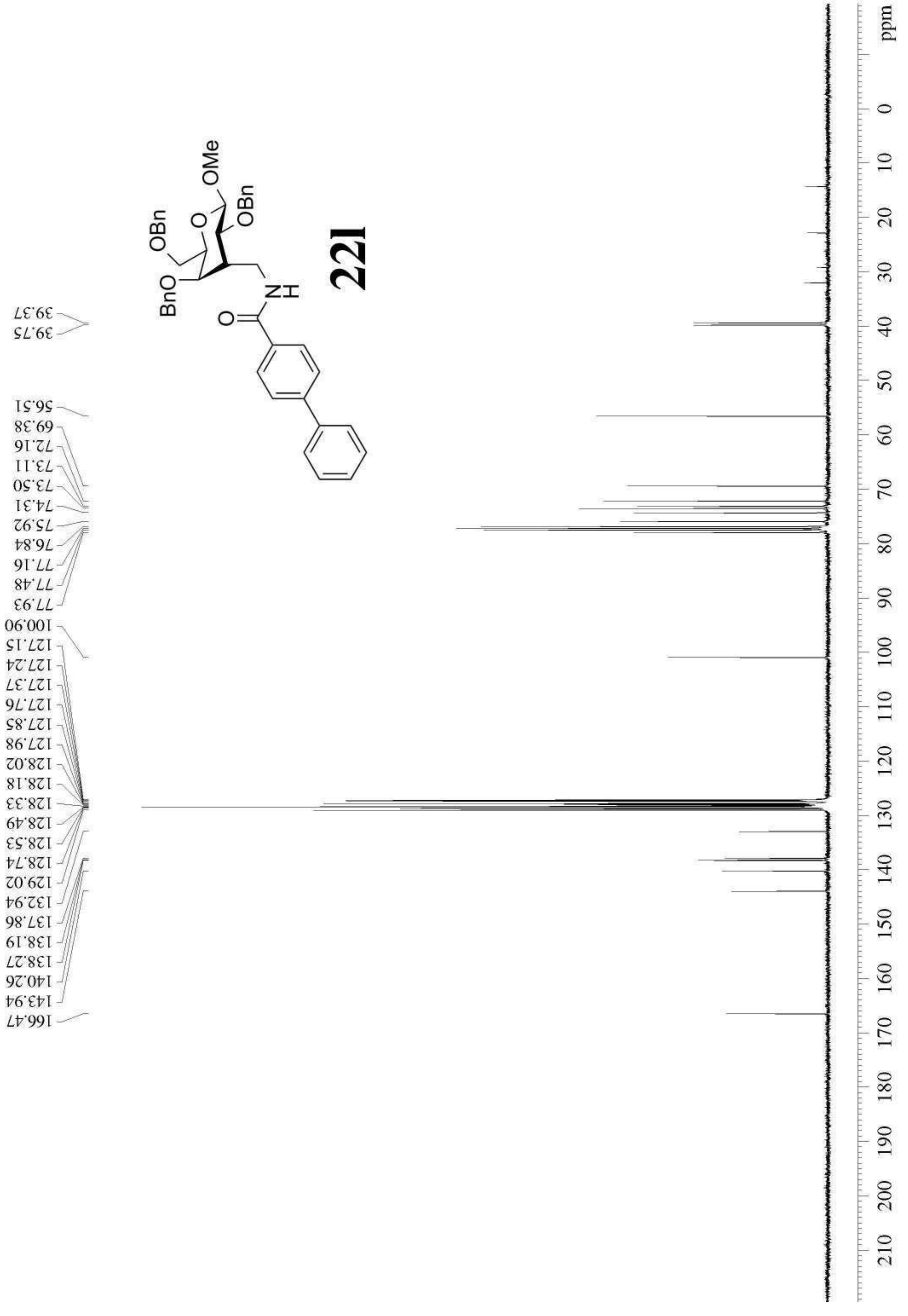
39.37
39.75
56.51
69.38
72.16
73.11
73.50
74.31
75.92
76.84
77.16
77.48
77.93
100.90
127.15
127.24
127.37
127.76
127.85
127.98
128.02
128.18
128.33
128.49
128.53
128.74
129.02
132.94
137.86
138.19
138.27
140.26
143.94
166.47
BnO
OBn
O
OMe
OBn
O
N
H
22l
ppm

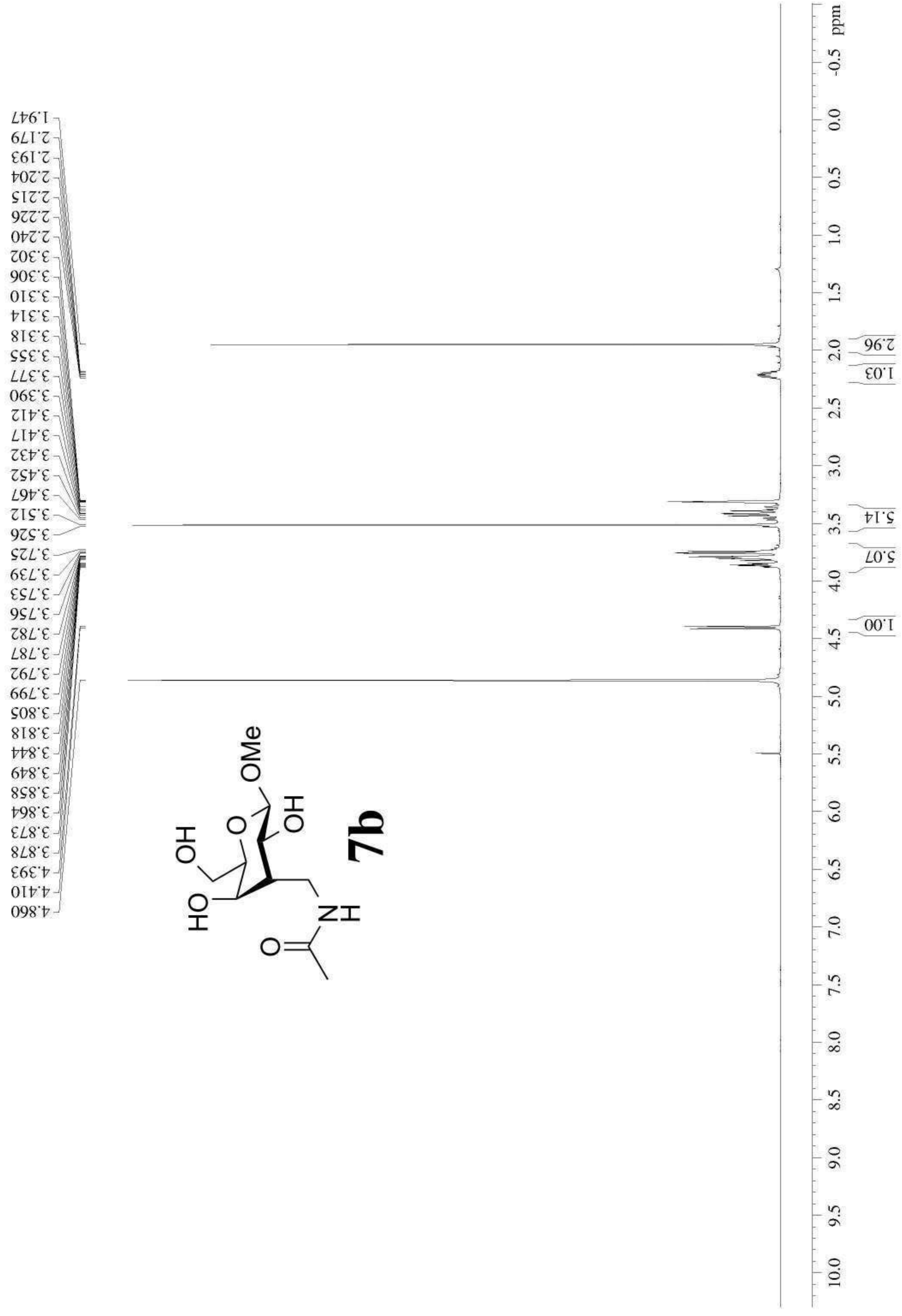
1.947
2.179
2.193
2.204
2.215
2.226
2.240
3.302
3.306
3.310
3.314
3.318
3.355
3.377
3.390
3.412
3.417
3.432
3.452
3.467
3.512
3.526
3.725
3.739
3.753
3.756
3.782
3.787
3.792
3.799
3.805
3.818
3.844
3.849
3.858
3.864
3.873
3.878
4.393
4.410
4.860
HO
OH
O
OMe
OH
N
H
O
7b
2.96
1.03
5.14
5.07
1.00
ppm

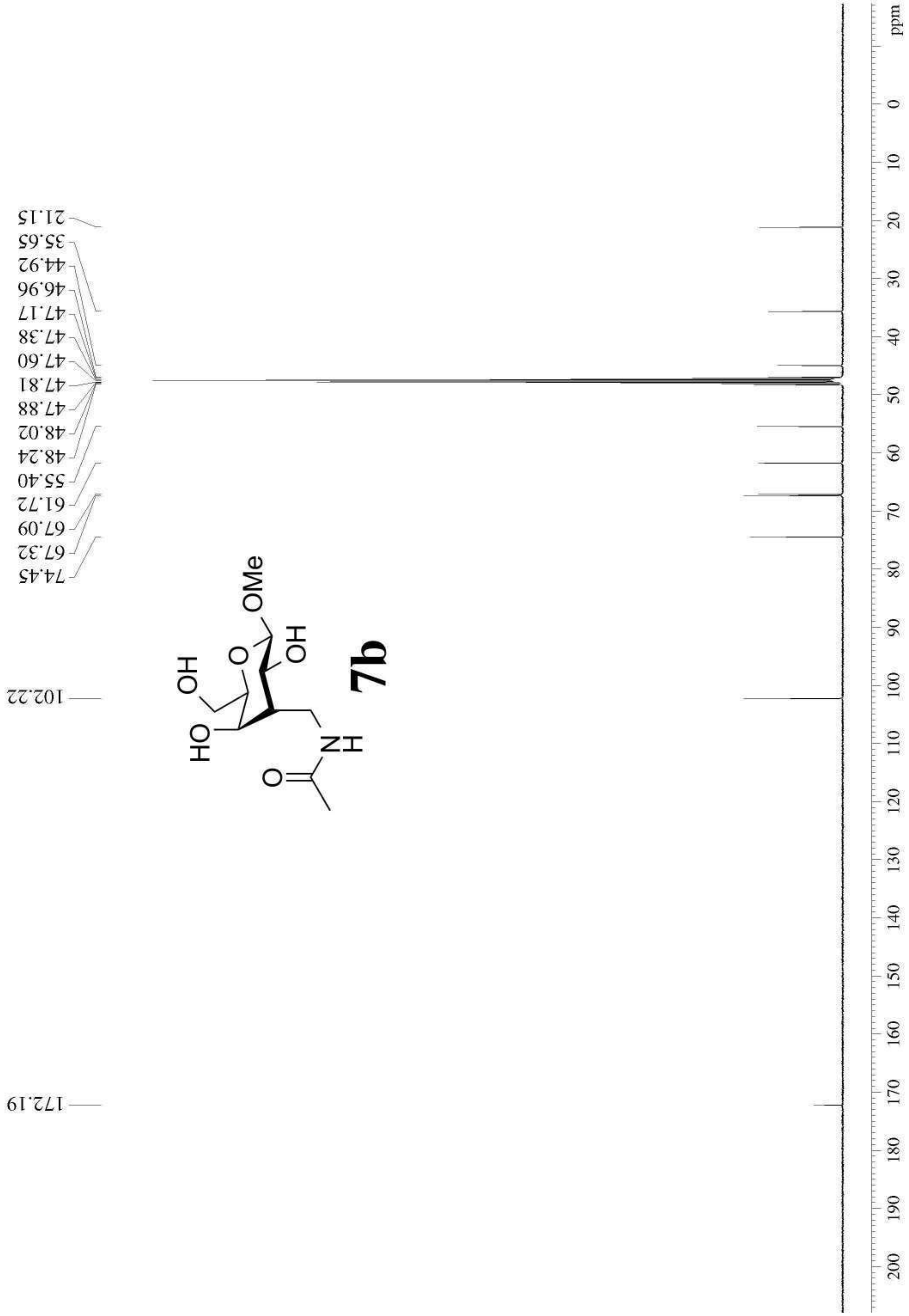
21.15
35.65
44.92
46.96
47.17
47.38
47.60
47.81
47.88
48.02
48.24
55.40
61.72
67.09
67.32
74.45
102.22
172.19
ppm
OMe
HO
OH
HO
O
N
H
O
7b

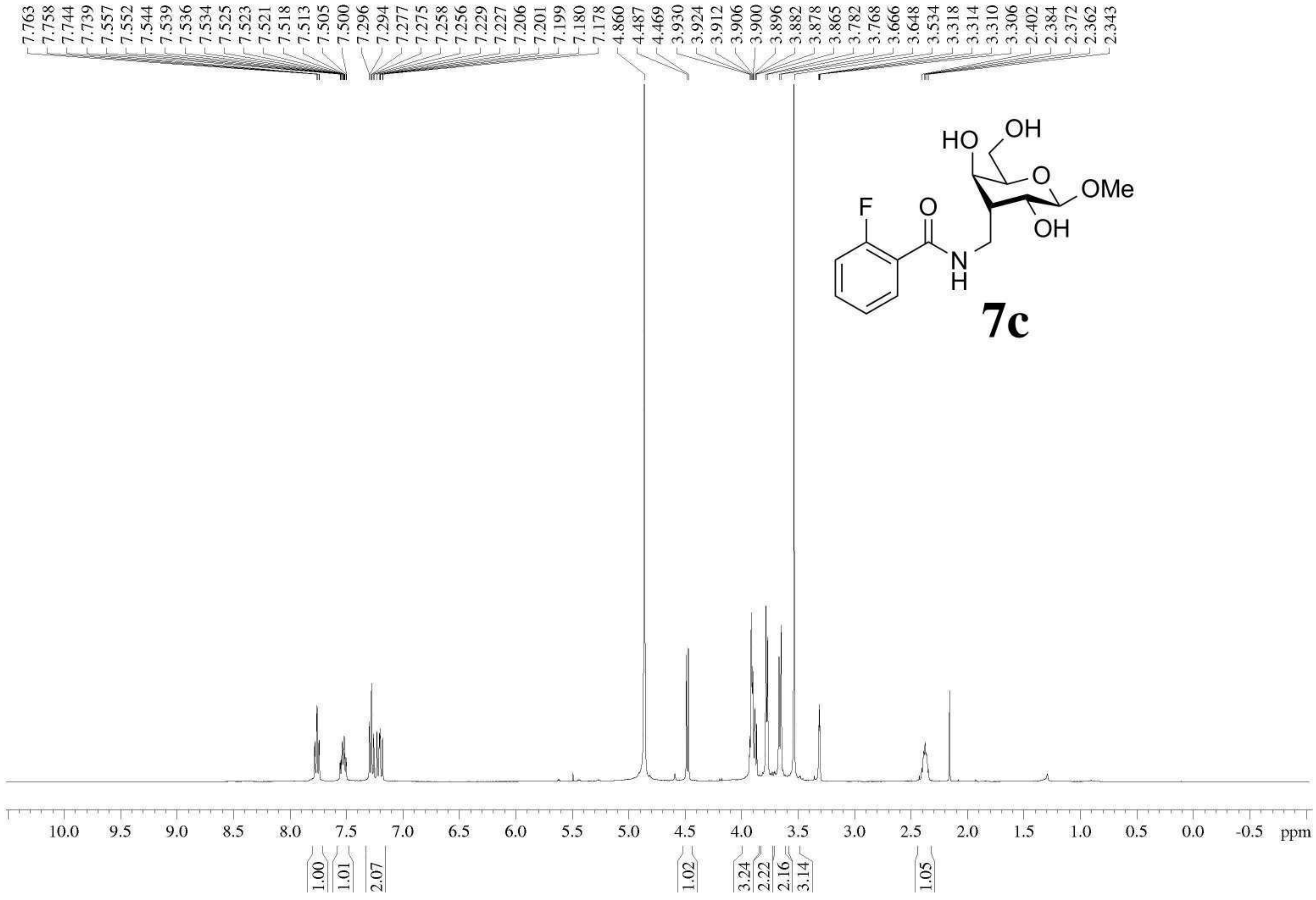
7.763
7.758
7.744
7.739
7.557
7.552
7.544
7.539
7.536
7.534
7.525
7.523
7.521
7.518
7.513
7.505
7.500
7.296
7.294
7.277
7.275
7.258
7.256
7.229
7.227
7.206
7.201
7.199
7.180
7.178
4.860
4.487
4.469
3.930
3.924
3.912
3.906
3.900
3.896
3.882
3.878
3.865
3.782
3.768
3.666
3.648
3.534
3.318
3.314
3.310
3.306
2.402
2.384
2.372
2.362
2.343
F
O
N
H
HO
OH
O
OMe
OH
7c
10.0
9.5
9.0
8.5
8.0
7.5
7.0
6.5
6.0
5.5
5.0
4.5
4.0
3.5
3.0
2.5
2.0
1.5
1.0
0.5
0.0
-0.5
ppm
1.00
1.01
2.07
1.02
3.24
2.22
2.16
3.14
1.05

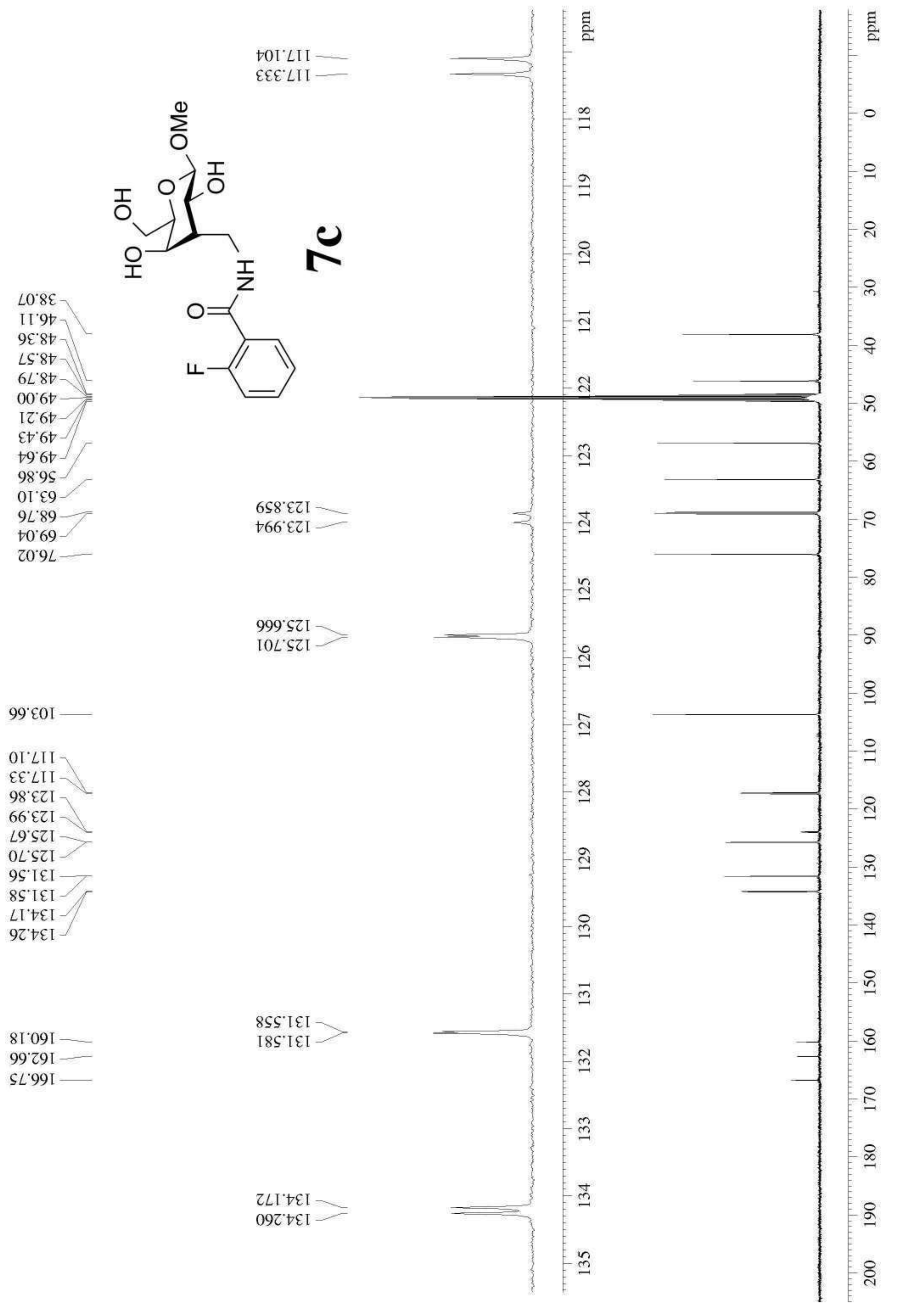
7c
F
O
NH
HO
OH
O
OMe
OH
38.07
46.11
48.36
48.57
48.79
49.00
49.21
49.43
49.64
56.86
63.10
68.76
69.04
76.02
103.66
117.10
117.33
123.86
123.99
125.67
125.70
131.56
131.58
134.17
134.26
160.18
162.66
166.75
117.104
117.333
123.859
123.994
125.666
125.701
131.558
131.581
134.172
134.260
ppm

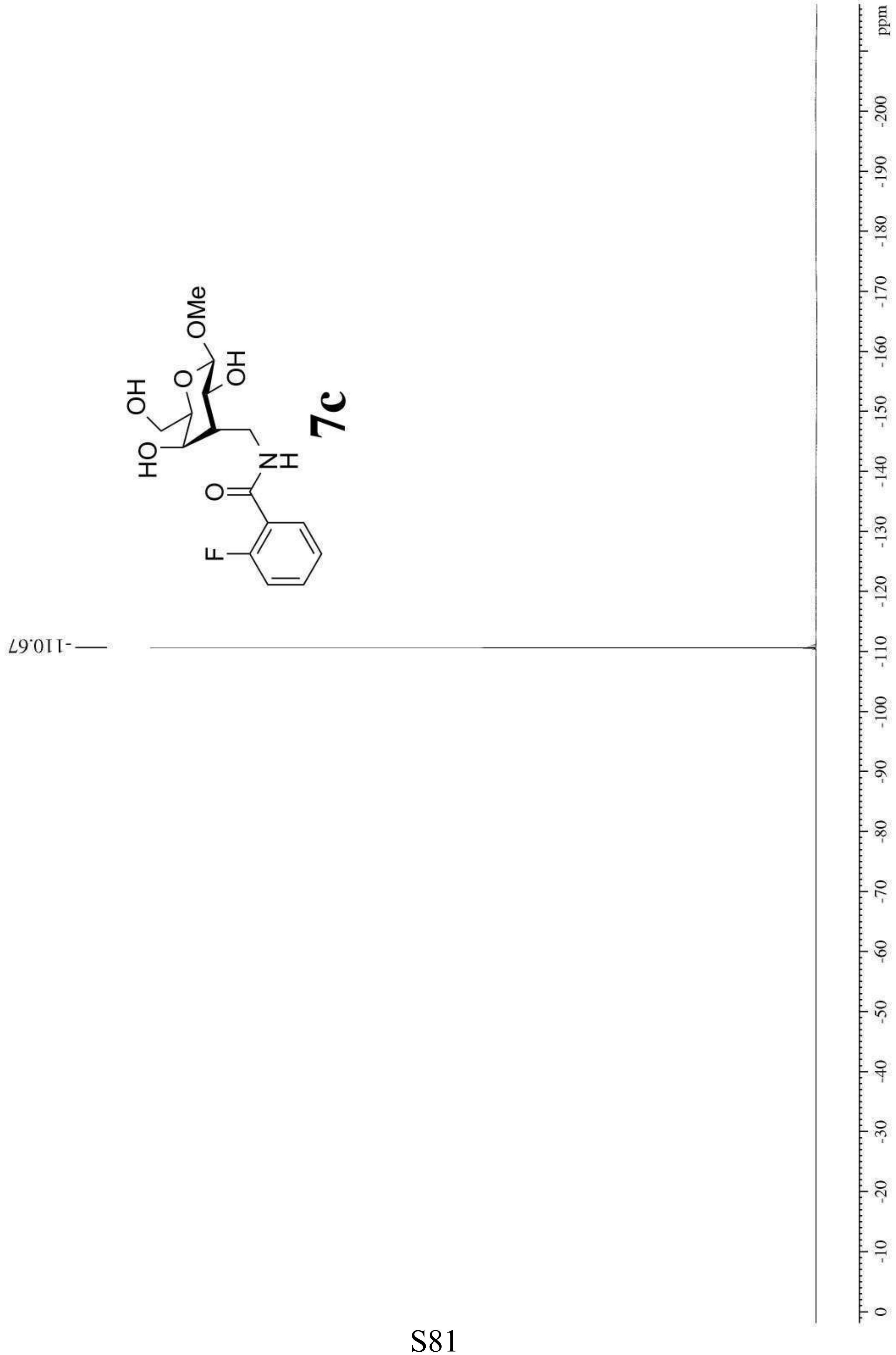
-110.67
7c
OMe
OH
HO
O
N
H
F
ppm
-200 -190 -180 -170 -160 -150 -140 -130 -120 -110 -100 -90 -80 -70 -60 -50 -40 -30 -20 -10 0

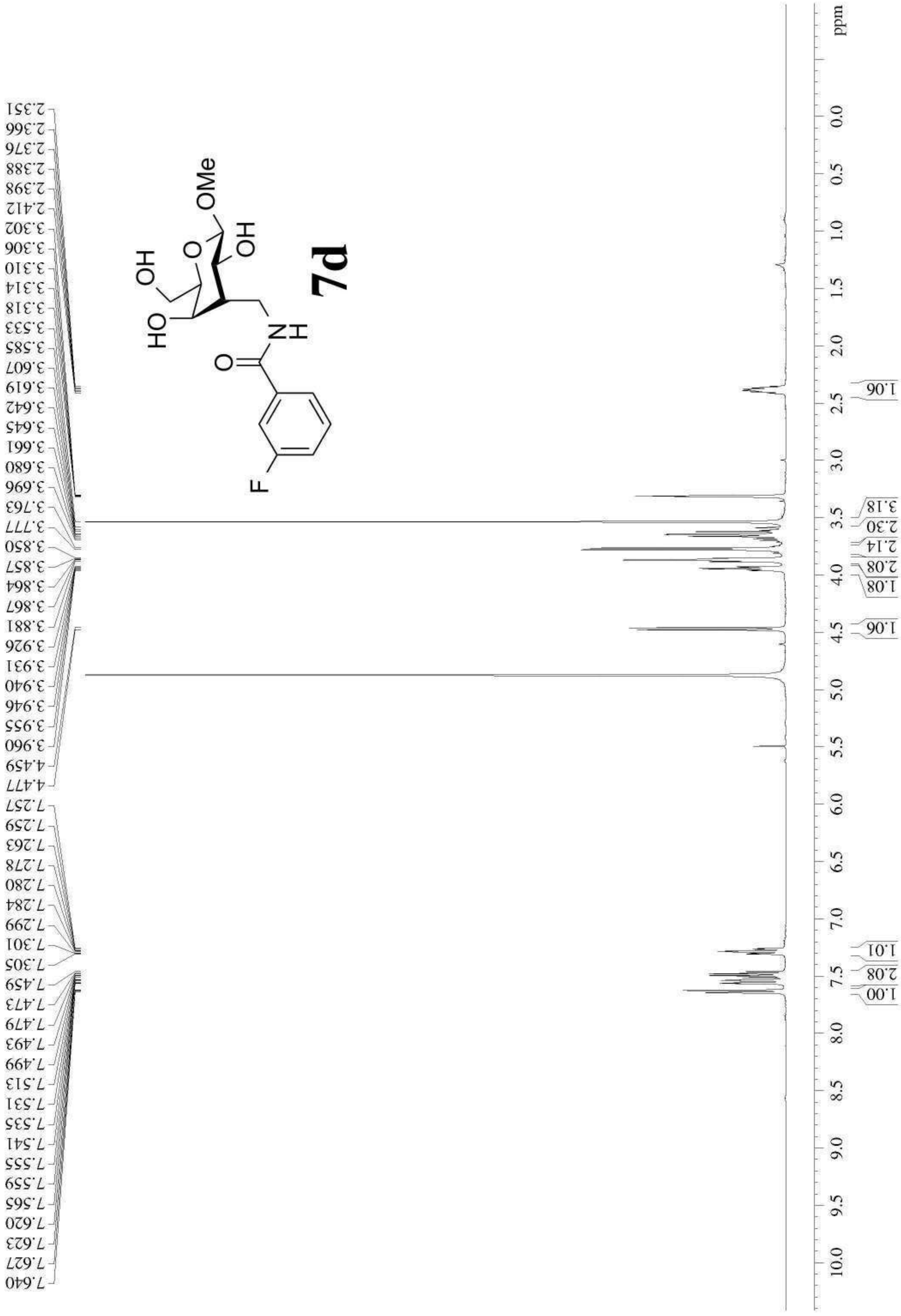
2.351
2.366
2.376
2.388
2.398
2.412
3.302
3.306
3.310
3.314
3.318
3.533
3.585
3.607
3.619
3.642
3.645
3.661
3.680
3.696
3.763
3.777
3.850
3.857
3.864
3.867
3.881
3.926
3.931
3.940
3.946
3.955
3.960
4.459
4.477
7.257
7.259
7.263
7.278
7.280
7.284
7.299
7.301
7.305
7.459
7.473
7.479
7.493
7.499
7.513
7.531
7.535
7.541
7.555
7.559
7.565
7.620
7.623
7.627
7.640
F
O
N
H
HO
OH
O
OH
OMe
7d
1.06
3.18
2.30
2.14
2.08
1.08
1.06
1.01
2.08
1.00
ppm
0.0
0.5
1.0
1.5
2.0
2.5
3.0
3.5
4.0
4.5
5.0
5.5
6.0
6.5
7.0
7.5
8.0
8.5
9.0
9.5
10.0

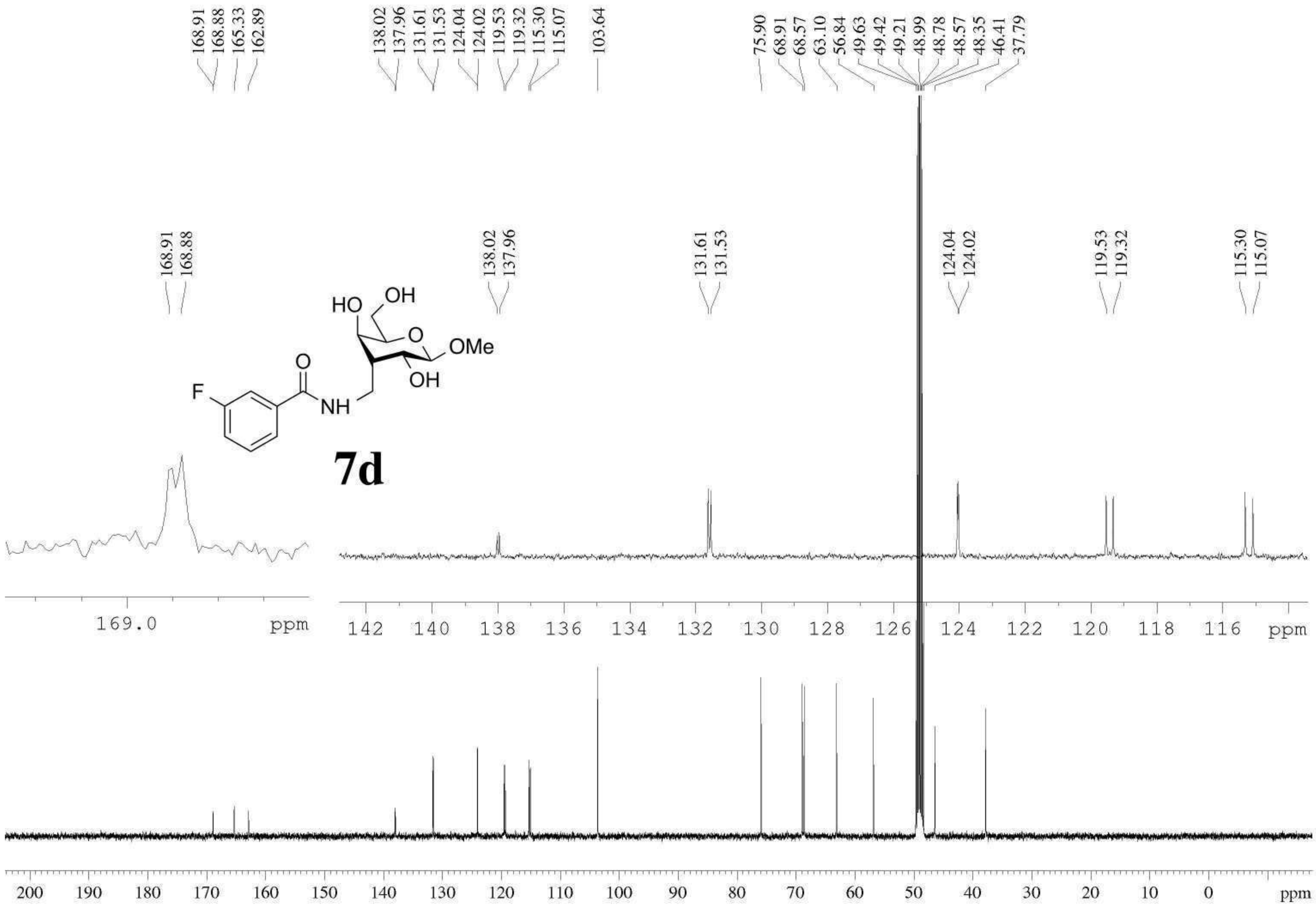
168.91
168.88
165.33
162.89
138.02
137.96
131.61
131.53
124.04
124.02
119.53
119.32
115.30
115.07
103.64
75.90
68.91
68.57
63.10
56.84
49.63
49.42
49.21
48.99
48.78
48.57
48.35
46.41
37.79
OMe
HO
OH
O
NH
F
7d
169.0
ppm
142 140 138 136 134 132 130 128 126 124 122 120 118 116 ppm
200 190 180 170 160 150 140 130 120 110 100 90 80 70 60 50 40 30 20 10 0 ppm

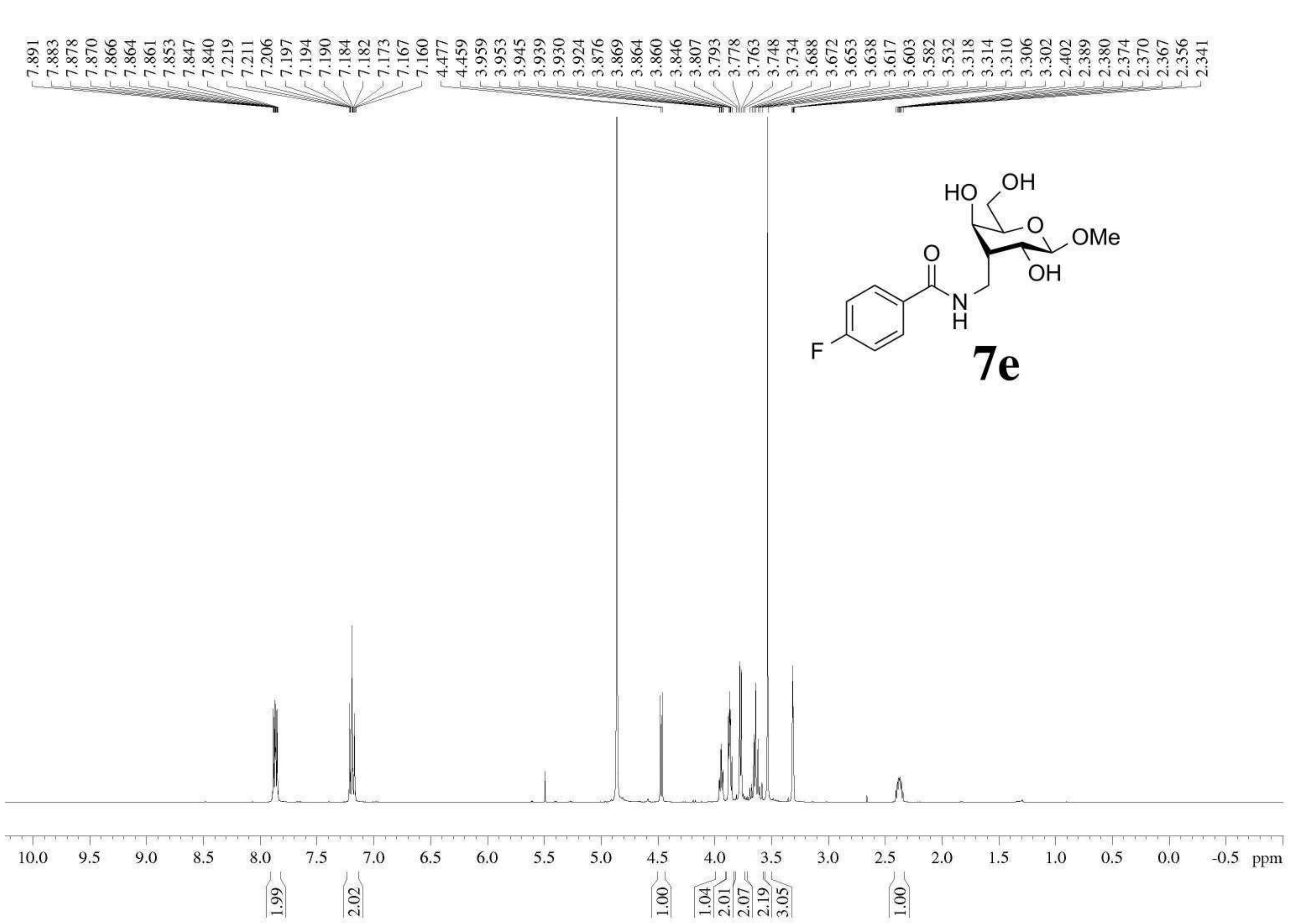
7.891
7.883
7.878
7.870
7.866
7.864
7.861
7.853
7.847
7.840
7.219
7.211
7.206
7.197
7.194
7.190
7.184
7.182
7.173
7.167
7.160
4.477
4.459
3.959
3.953
3.945
3.939
3.930
3.924
3.876
3.869
3.864
3.860
3.846
3.807
3.793
3.778
3.763
3.748
3.734
3.688
3.672
3.653
3.638
3.617
3.603
3.582
3.532
3.318
3.314
3.310
3.306
3.302
2.402
2.389
2.380
2.374
2.370
2.367
2.356
2.341
HO
OH
O
OMe
OH
N
H
F
7e
10.0
9.5
9.0
8.5
8.0
7.5
7.0
6.5
6.0
5.5
5.0
4.5
4.0
3.5
3.0
2.5
2.0
1.5
1.0
0.5
0.0
-0.5
ppm
1.99
2.02
1.00
1.04
2.01
2.07
2.19
3.05
1.00

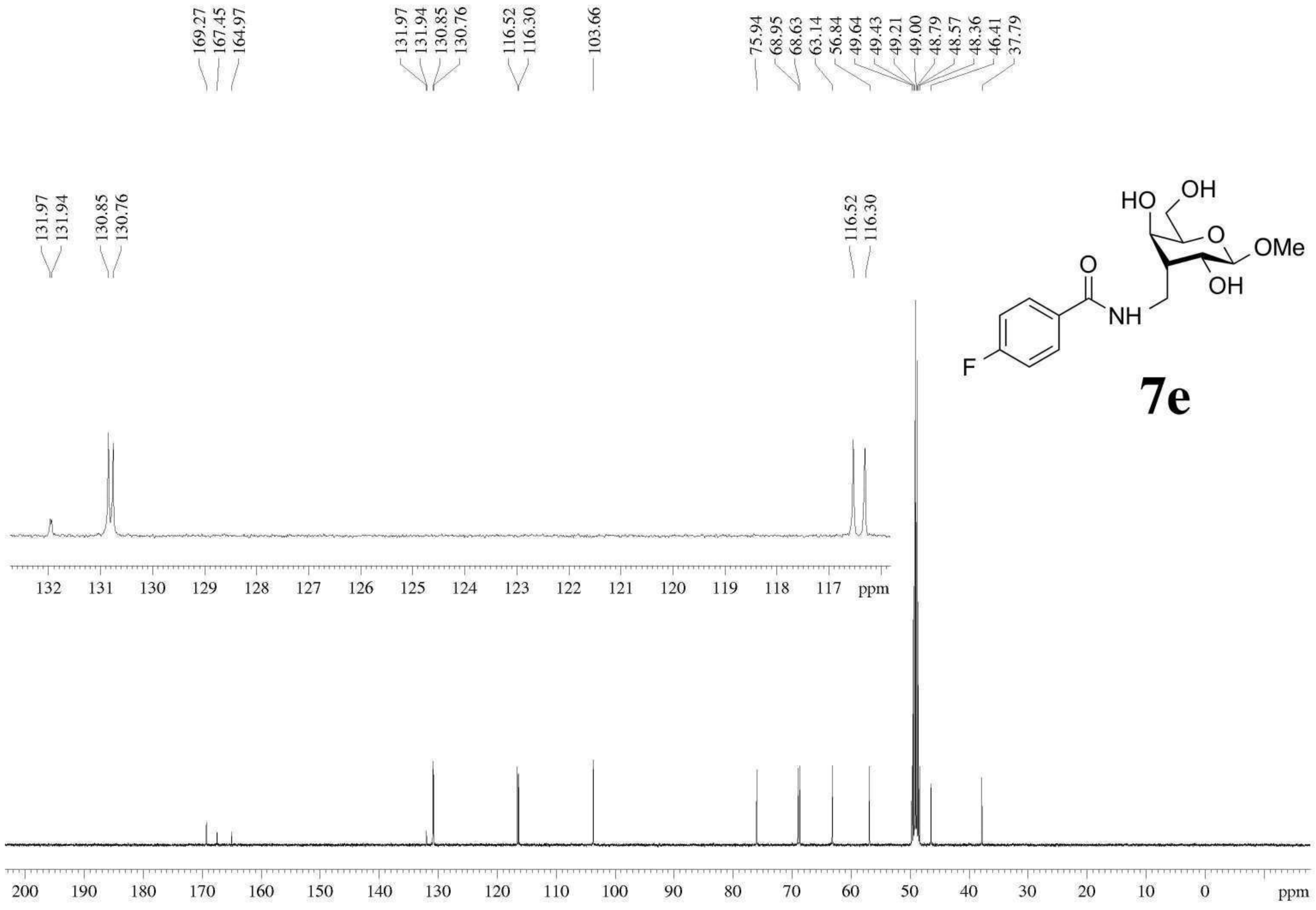
169.27
167.45
164.97
131.97
131.94
130.85
130.76
116.52
116.30
103.66
75.94
68.95
68.63
63.14
56.84
49.64
49.43
49.21
49.00
48.79
48.57
48.36
46.41
37.79
HO
OH
O
OMe
OH
NH
F
ppm

7e

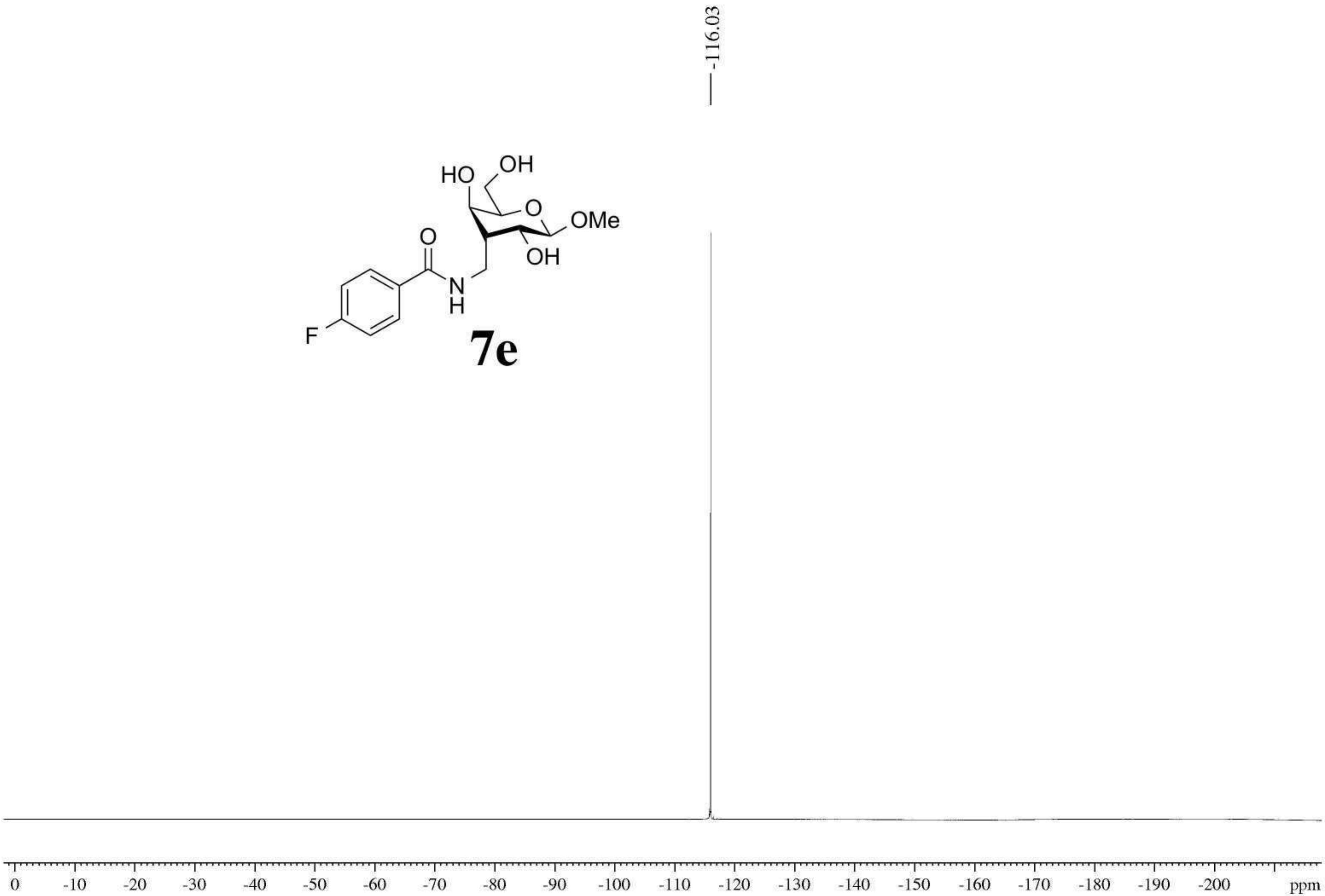

-116.03
HO
OH
O
OMe
OH
O
N
H
F
0 -10 -20 -30 -40 -50 -60 -70 -80 -90 -100 -110 -120 -130 -140 -150 -160 -170 -180 -190 -200 ppm

7e

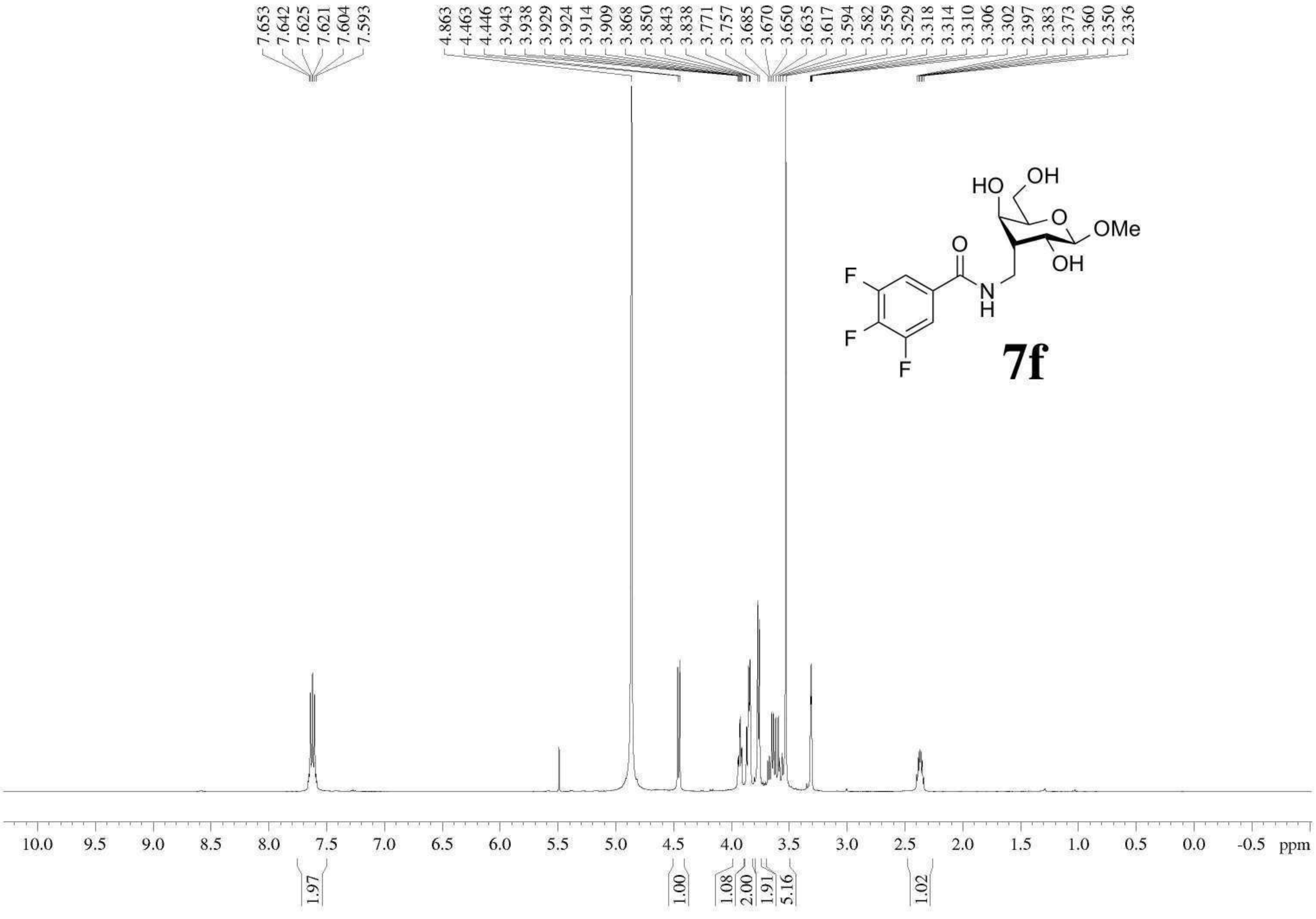
7.653
7.642
7.625
7.621
7.604
7.593
4.863
4.463
4.446
3.943
3.938
3.929
3.924
3.914
3.909
3.868
3.850
3.843
3.838
3.771
3.757
3.685
3.670
3.650
3.635
3.617
3.594
3.582
3.559
3.529
3.318
3.314
3.310
3.306
3.302
2.397
2.383
2.373
2.360
2.350
2.336
HO
OH
O
OMe
OH
O
F
N
H
F
F
10.0 9.5 9.0 8.5 8.0 7.5 7.0 6.5 6.0 5.5 5.0 4.5 4.0 3.5 3.0 2.5 2.0 1.5 1.0 0.5 0.0 -0.5 ppm
1.97
1.00
1.08
2.00
1.91
5.16
1.02

7f

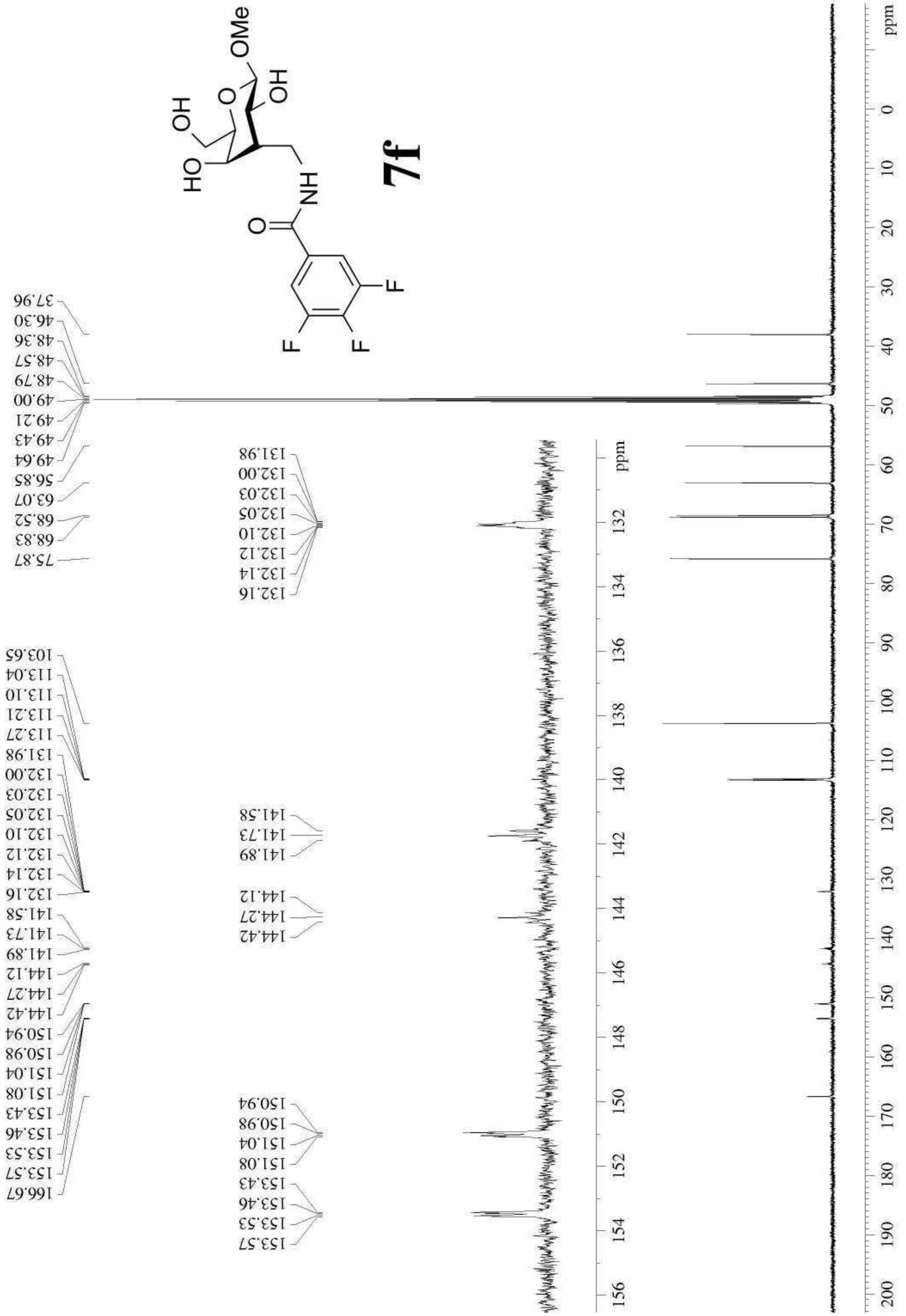
7f
37.96
46.30
48.36
48.57
48.79
49.00
49.21
49.43
49.64
56.85
63.07
68.52
68.83
75.87
103.65
113.04
113.10
113.21
113.27
131.98
132.00
132.03
132.05
132.10
132.12
132.14
132.16
141.58
141.73
141.89
144.12
144.27
144.42
150.94
150.98
151.04
151.08
153.43
153.46
153.53
153.57
166.67
ppm

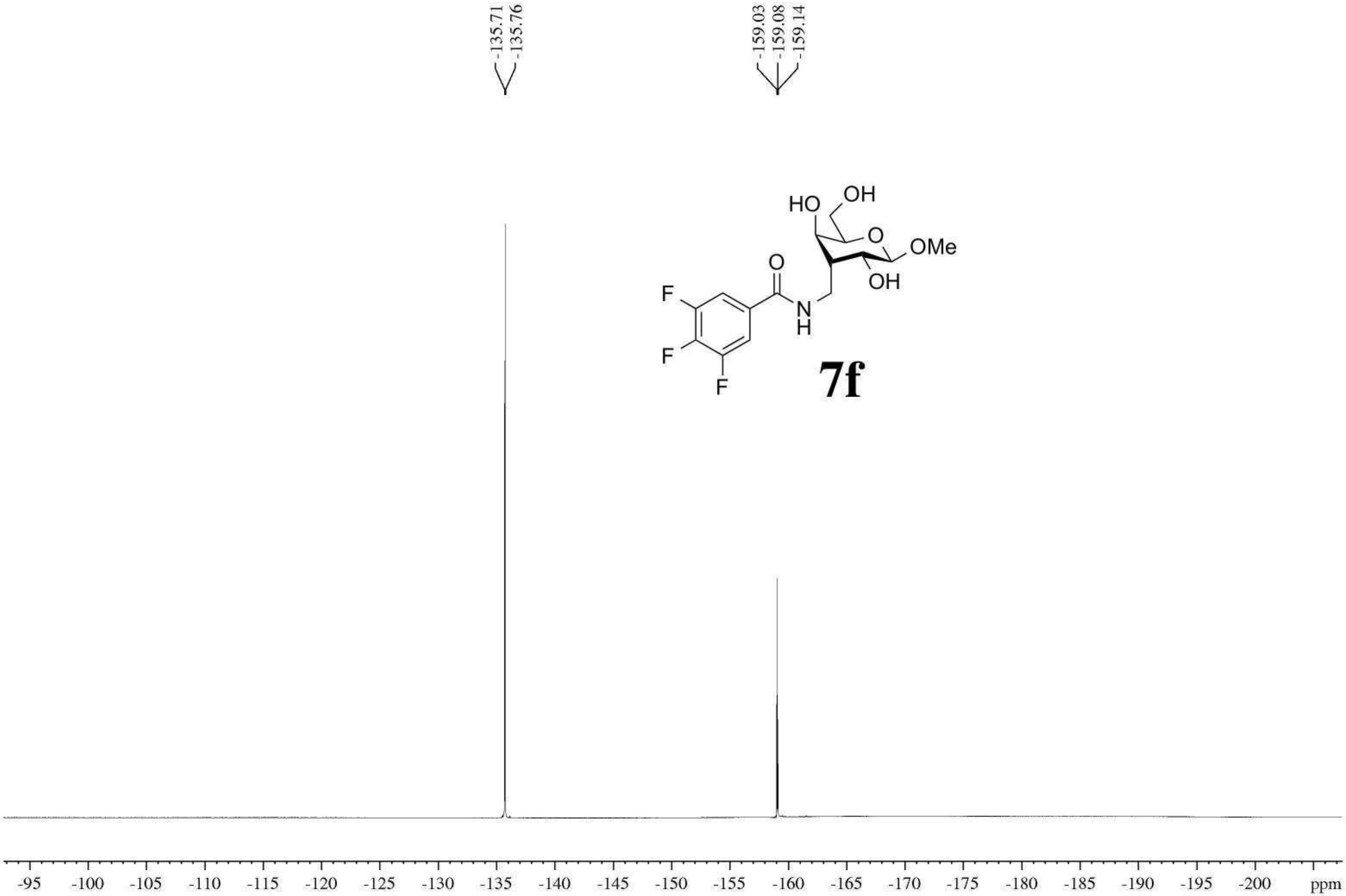
-135.71
-135.76
-159.03
-159.08
-159.14
HO
OH
O
OMe
OH
O
F
F
F
N
H
7f
-95 -100 -105 -110 -115 -120 -125 -130 -135 -140 -145 -150 -155 -160 -165 -170 -175 -180 -185 -190 -195 -200 ppm

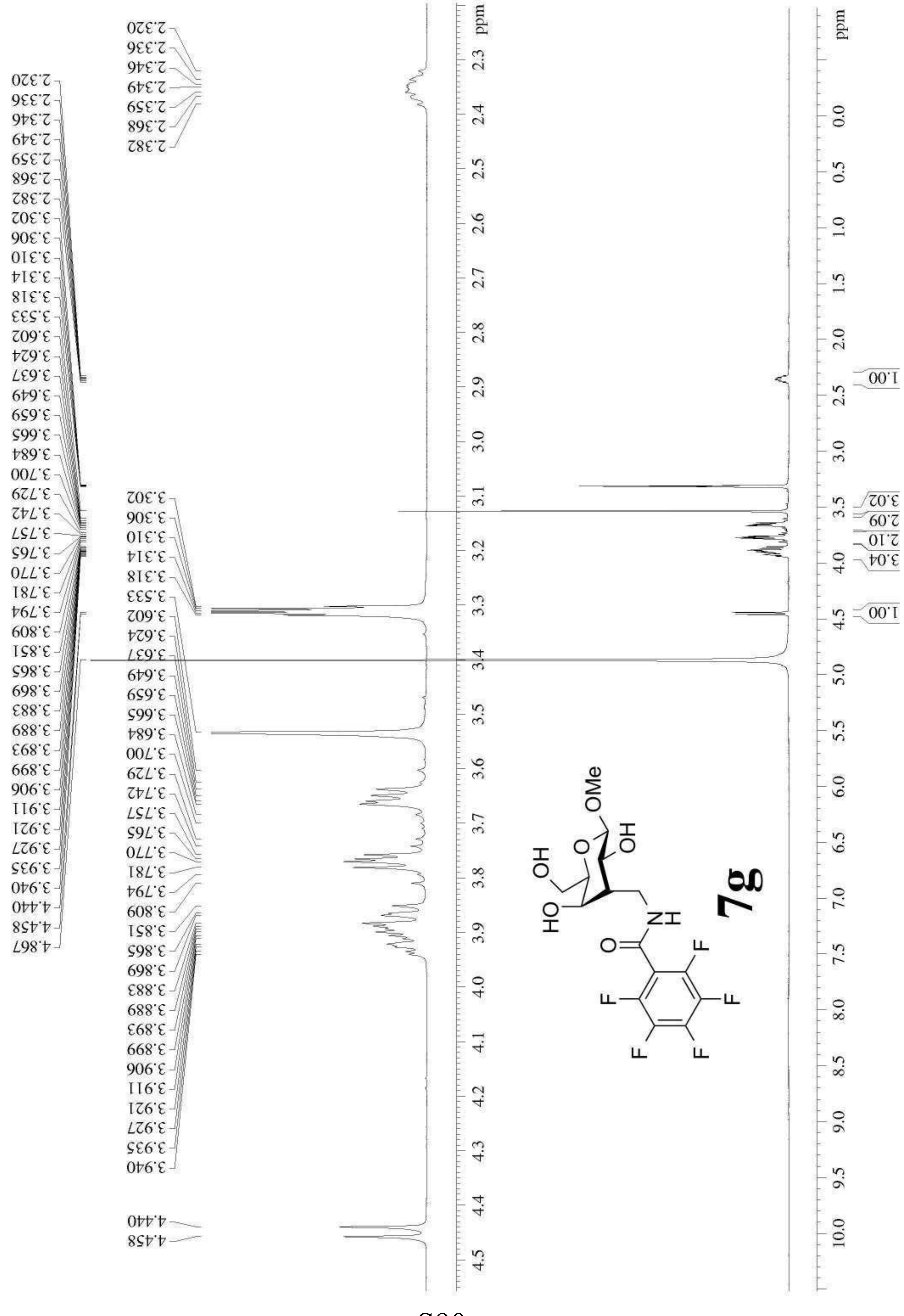

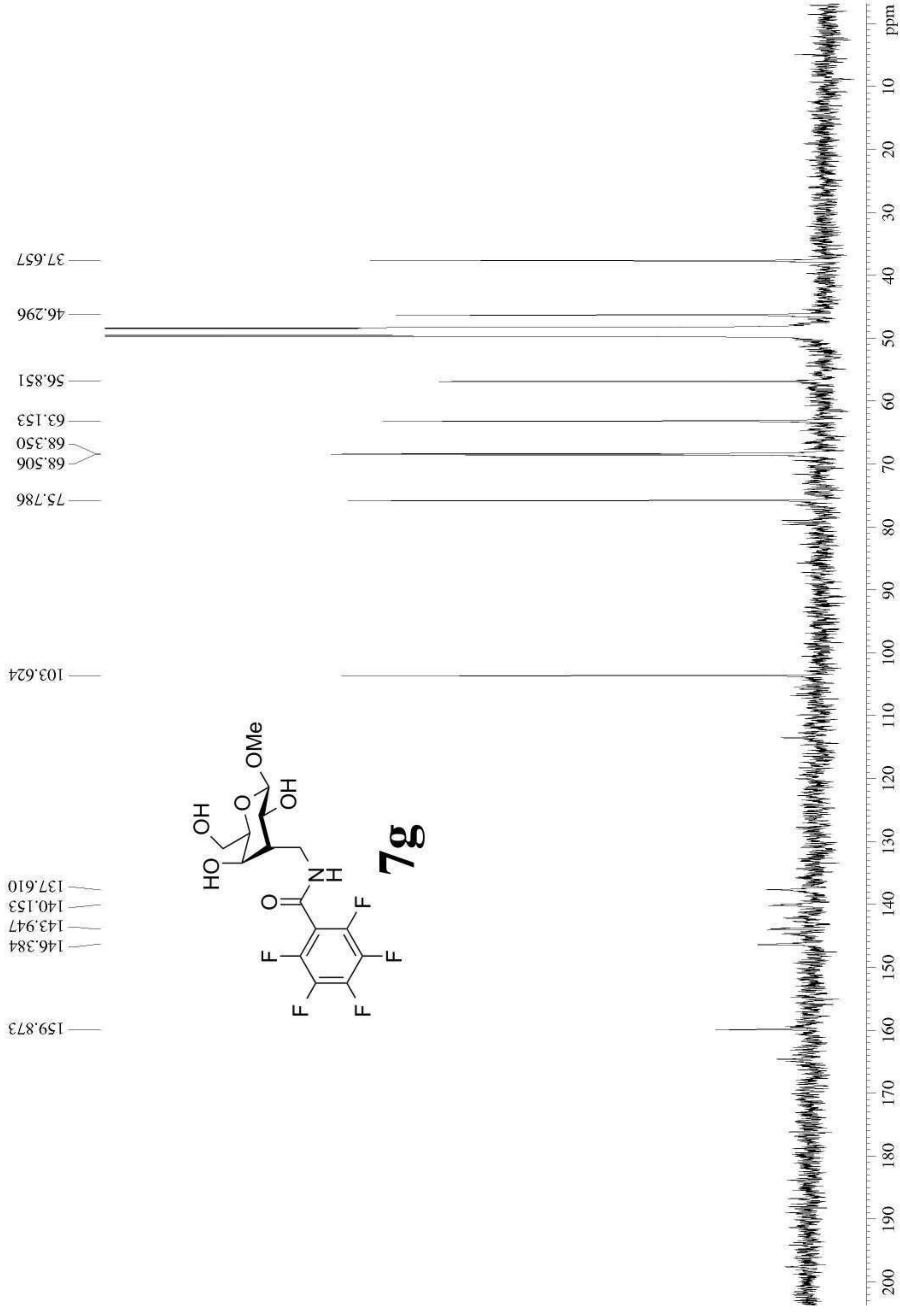
37.657
46.296
56.851
63.153
68.350
68.506
75.786
103.624
137.610
140.153
143.947
146.384
159.873
7g
ppm

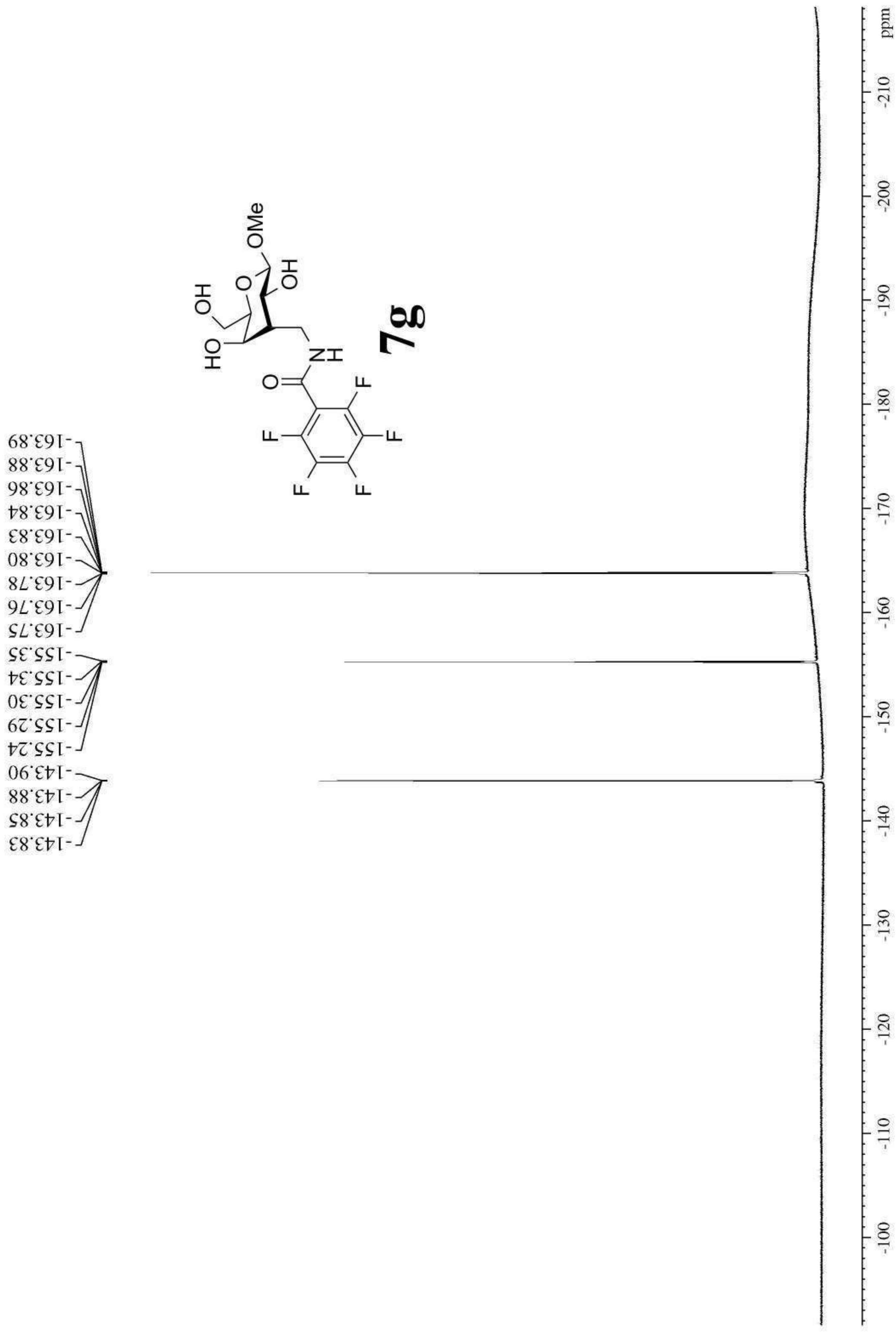
-163.89
-163.88
-163.86
-163.84
-163.83
-163.80
-163.78
-163.76
-163.75
-155.35
-155.34
-155.30
-155.29
-155.24
-143.90
-143.88
-143.85
-143.83
7g
ppm
-210
-200
-190
-180
-170
-160
-150
-140
-130
-120
-110
-100

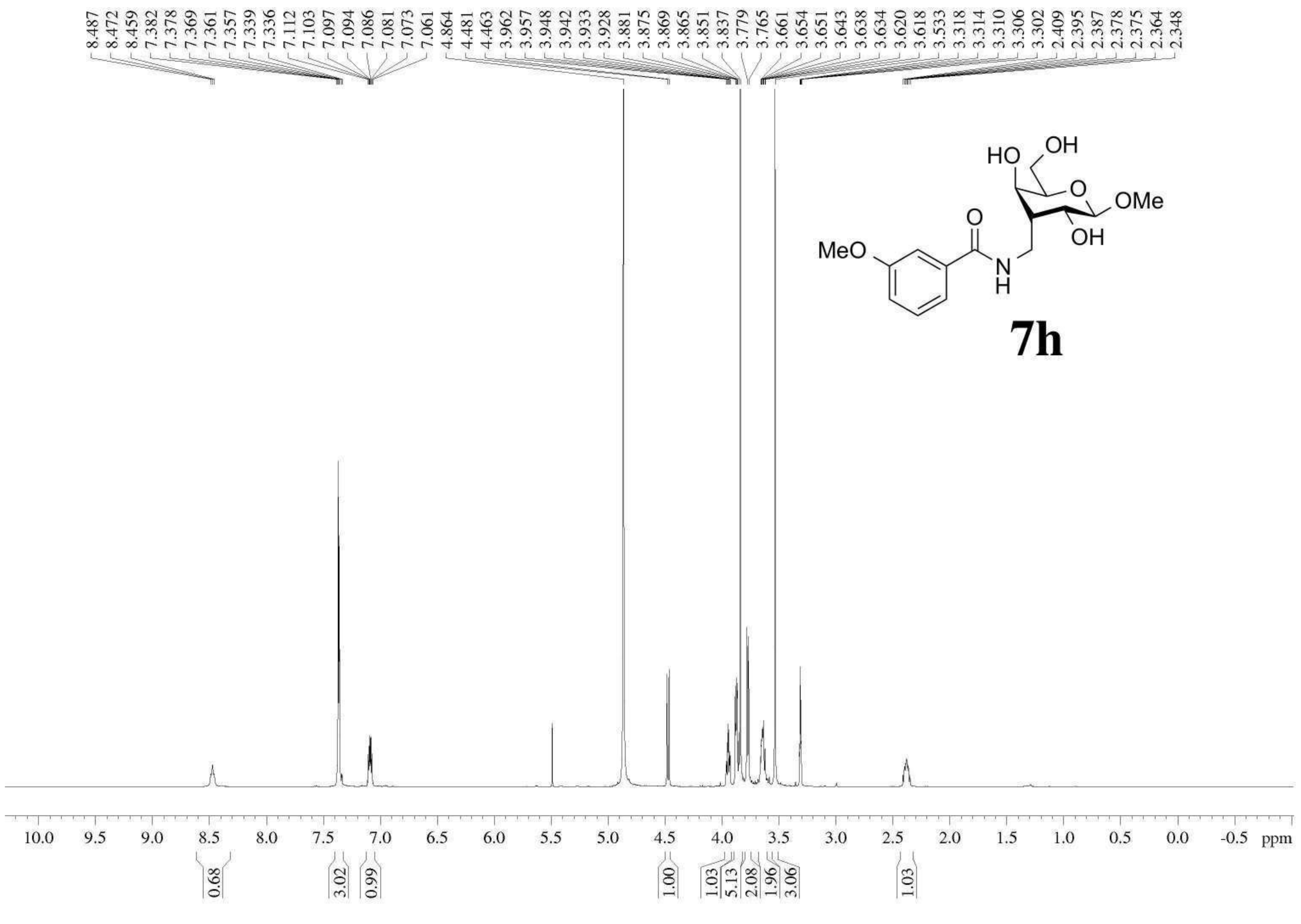
8.487
8.472
8.459
7.382
7.378
7.369
7.361
7.357
7.339
7.336
7.112
7.103
7.097
7.094
7.086
7.081
7.073
7.061
4.864
4.481
4.463
3.962
3.957
3.948
3.942
3.933
3.928
3.881
3.875
3.869
3.865
3.851
3.837
3.779
3.765
3.661
3.654
3.651
3.643
3.638
3.634
3.620
3.618
3.533
3.318
3.314
3.310
3.306
3.302
2.409
2.395
2.387
2.378
2.375
2.364
2.348
HO
OH
O
OMe
OH
MeO
N
H
7h
10.0 9.5 9.0 8.5 8.0 7.5 7.0 6.5 6.0 5.5 5.0 4.5 4.0 3.5 3.0 2.5 2.0 1.5 1.0 0.5 0.0 -0.5 ppm
0.68
3.02
0.99
1.00
1.03
5.13
2.08
1.96
3.06
1.03

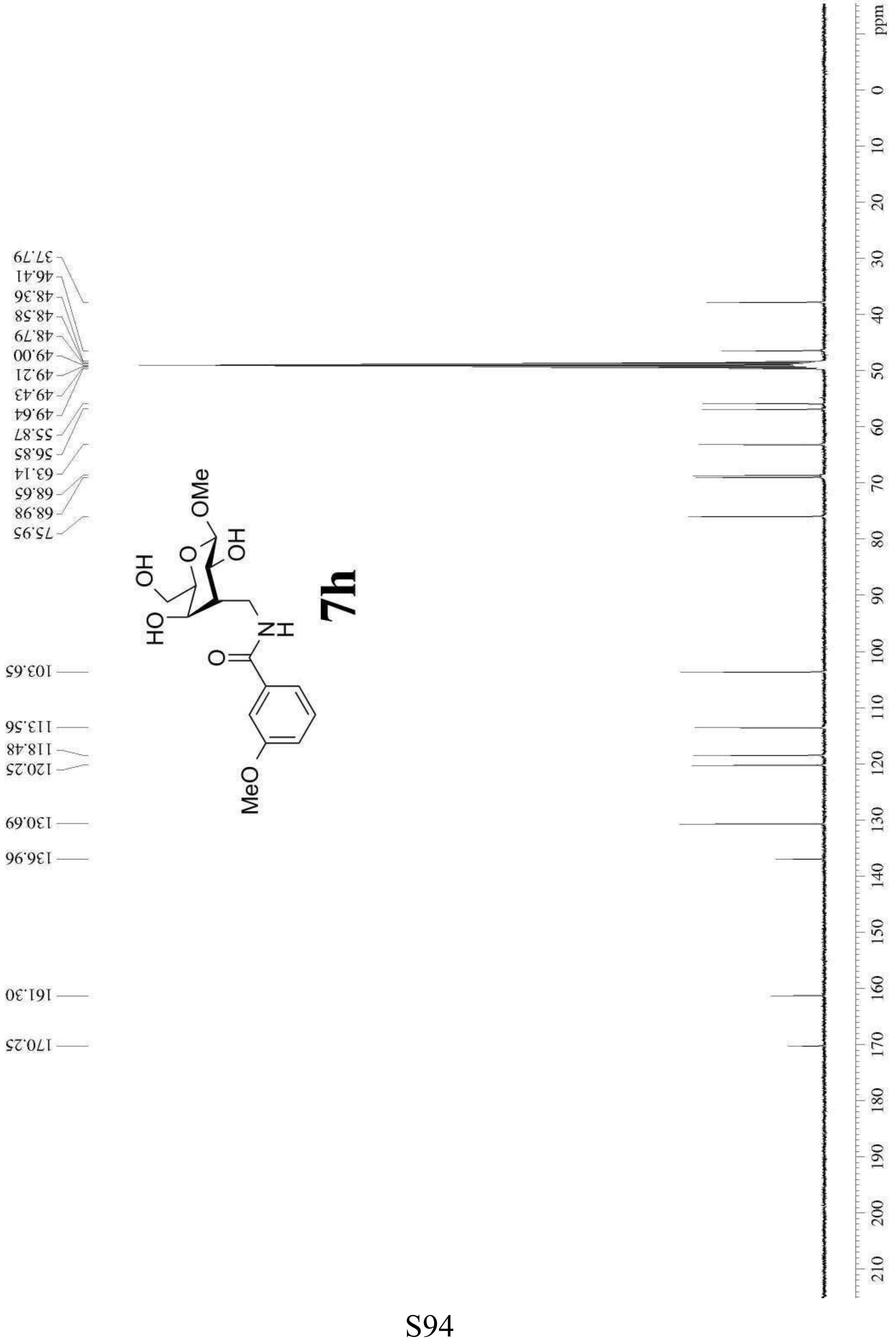

37.79
46.41
48.36
48.58
48.79
49.00
49.21
49.43
49.64
55.87
56.85
63.14
68.65
68.98
75.95
103.65
113.56
118.48
120.25
130.69
136.96
161.30
170.25
7h
ppm

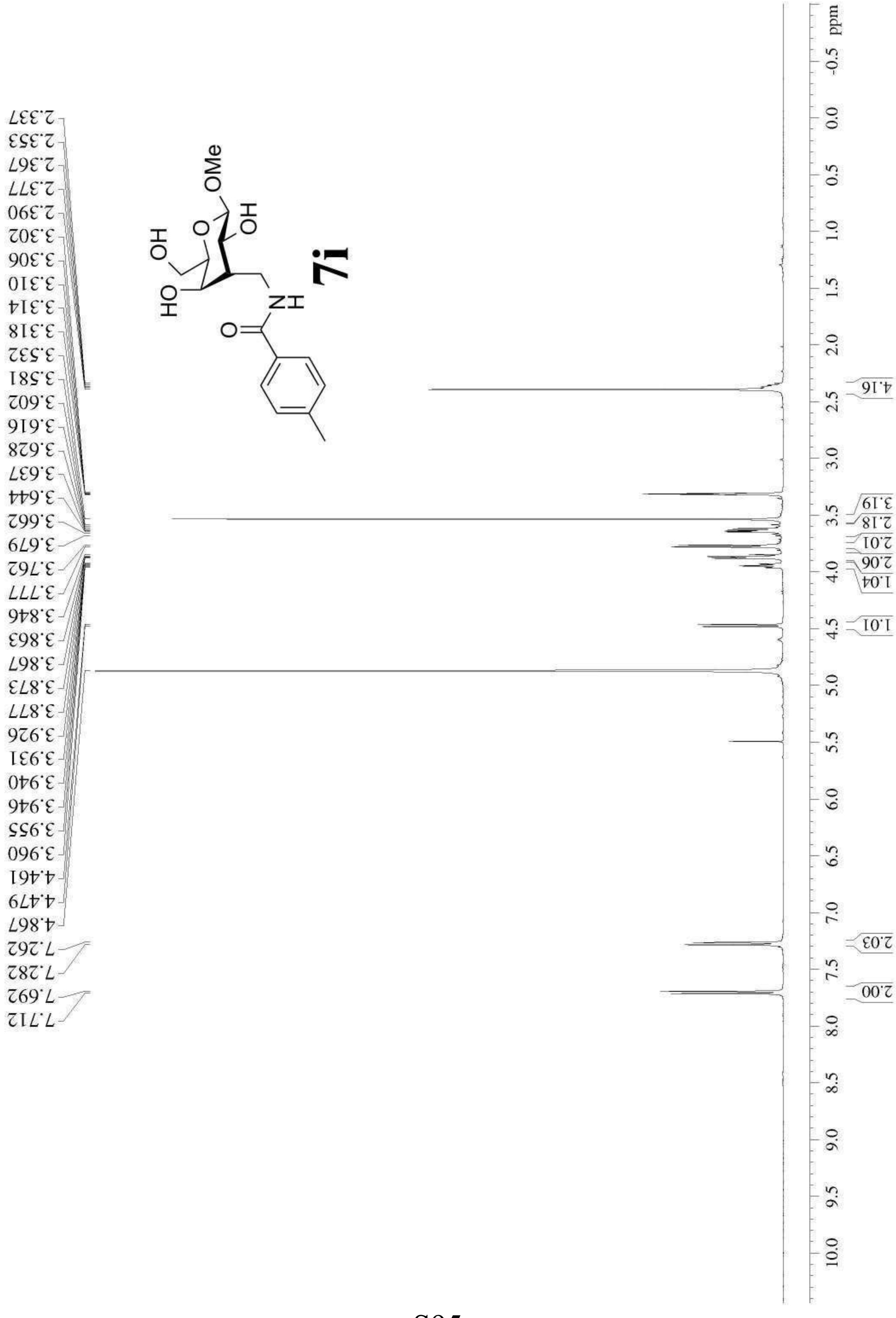
7i
OMe
OH
HO
HO
O
N
H
O
2.337
2.353
2.367
2.377
2.390
3.302
3.306
3.310
3.314
3.318
3.532
3.581
3.602
3.616
3.628
3.637
3.644
3.662
3.679
3.762
3.777
3.846
3.863
3.867
3.873
3.877
3.926
3.931
3.940
3.946
3.955
3.960
4.461
4.479
4.867
7.262
7.282
7.692
7.712
4.16
3.19
2.18
2.01
2.06
1.04
1.01
2.03
2.00
ppm

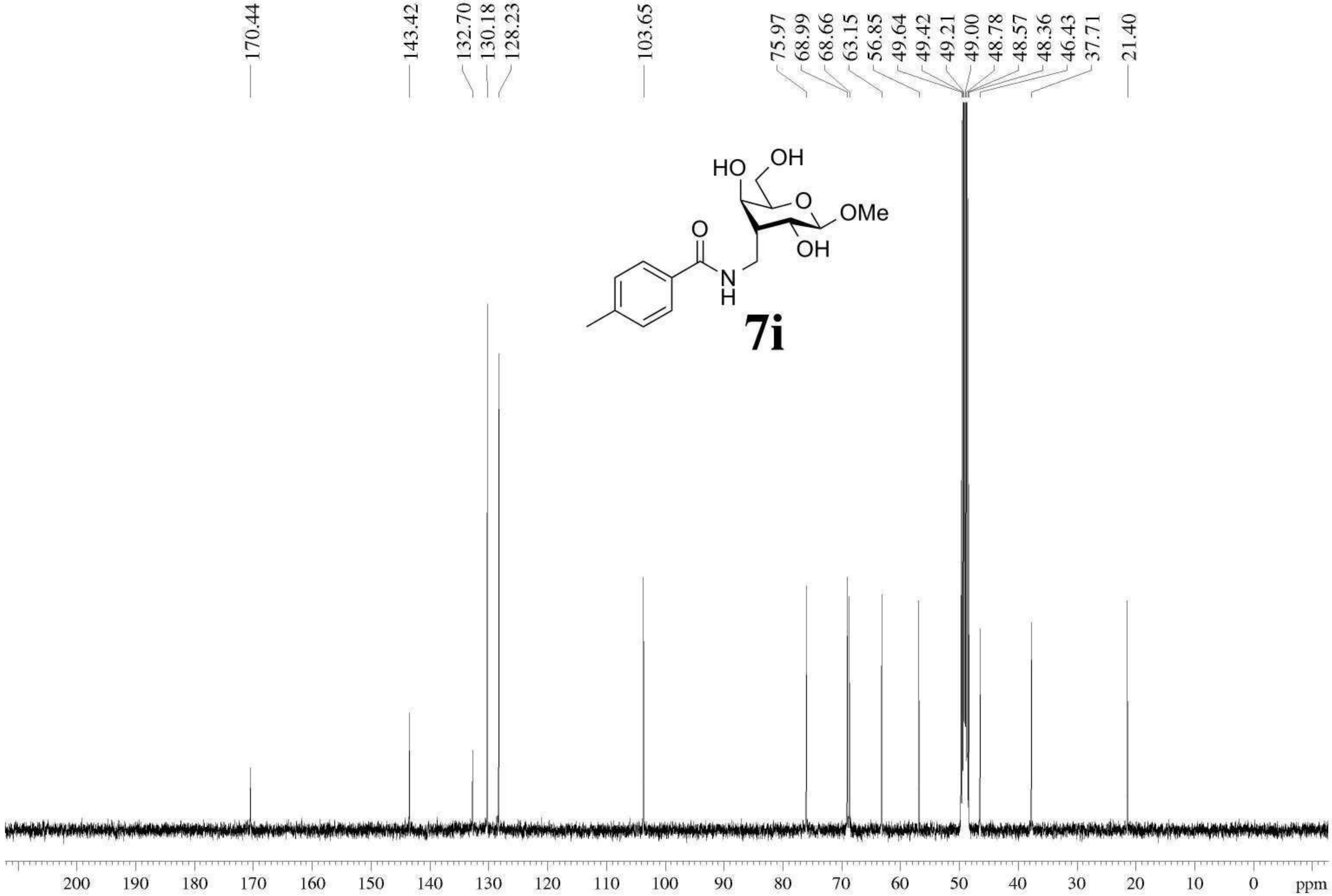
170.44
143.42
132.70
130.18
128.23
103.65
75.97
68.99
68.66
63.15
56.85
49.64
49.42
49.21
49.00
48.78
48.57
48.36
46.43
37.71
21.40
HO
OH
O
OMe
OH
O
N
H
7i
200
190
180
170
160
150
140
130
120
110
100
90
80
70
60
50
40
30
20
10
0
ppm

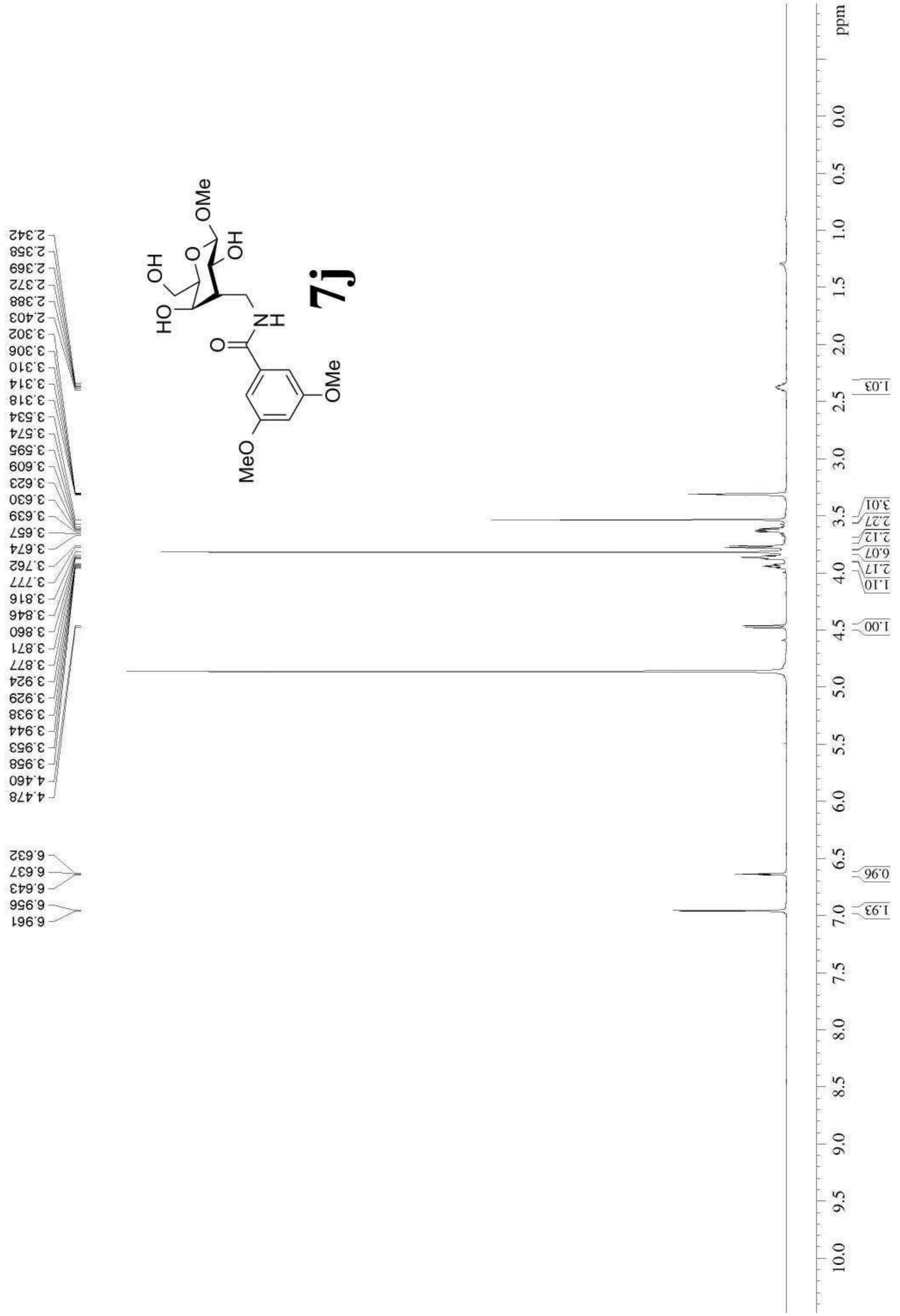
7j
ppm

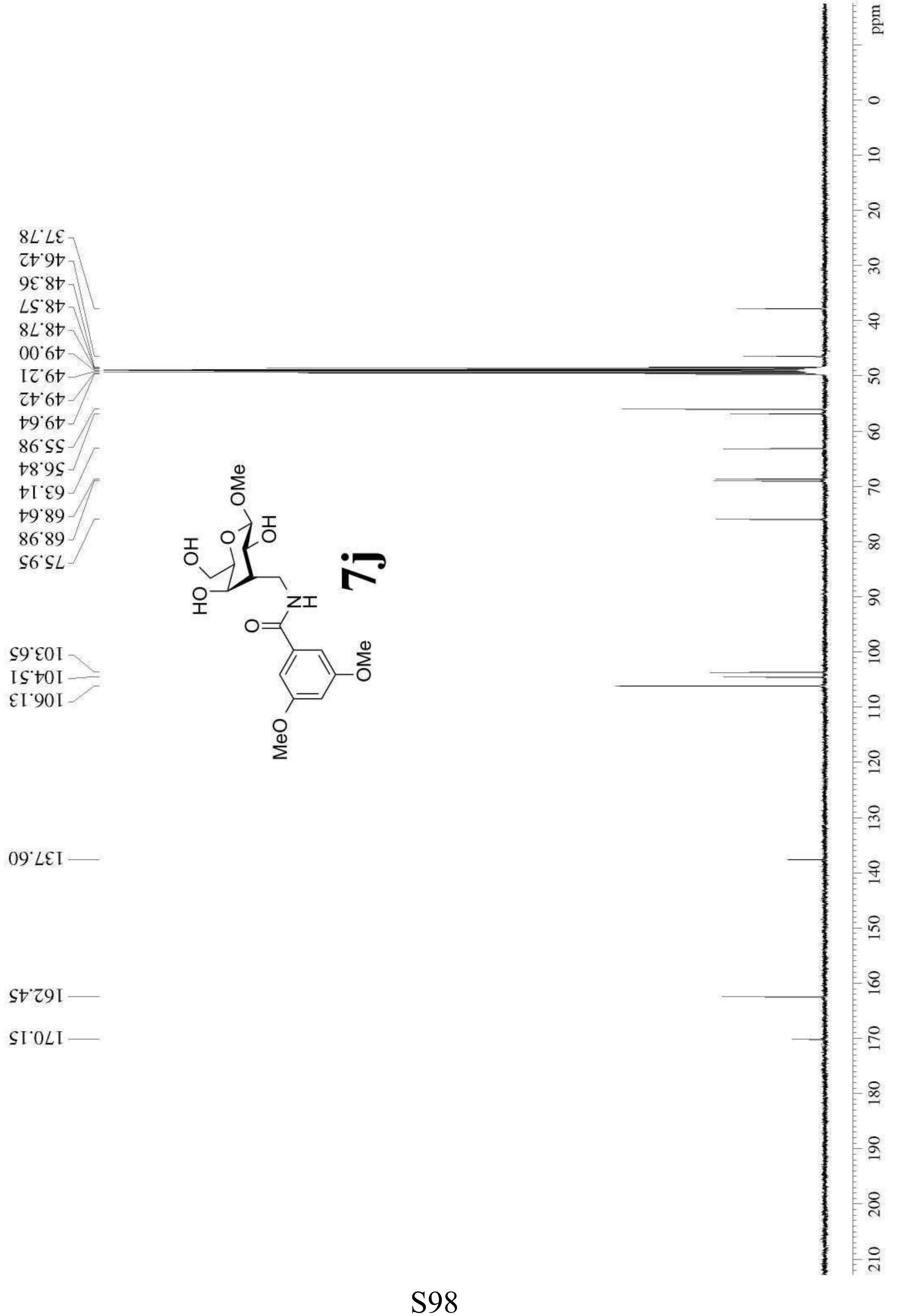
37.78
46.42
48.36
48.57
48.78
49.00
49.21
49.42
49.64
55.98
56.84
63.14
68.64
68.98
75.95
103.65
104.51
106.13
137.60
162.45
170.15
OMe
HO
OH
O
OH
HO
N
H
O
OMe
MeO
7j
ppm
0
10
20
30
40
50
60
70
80
90
100
110
120
130
140
150
160
170
180
190
200
210

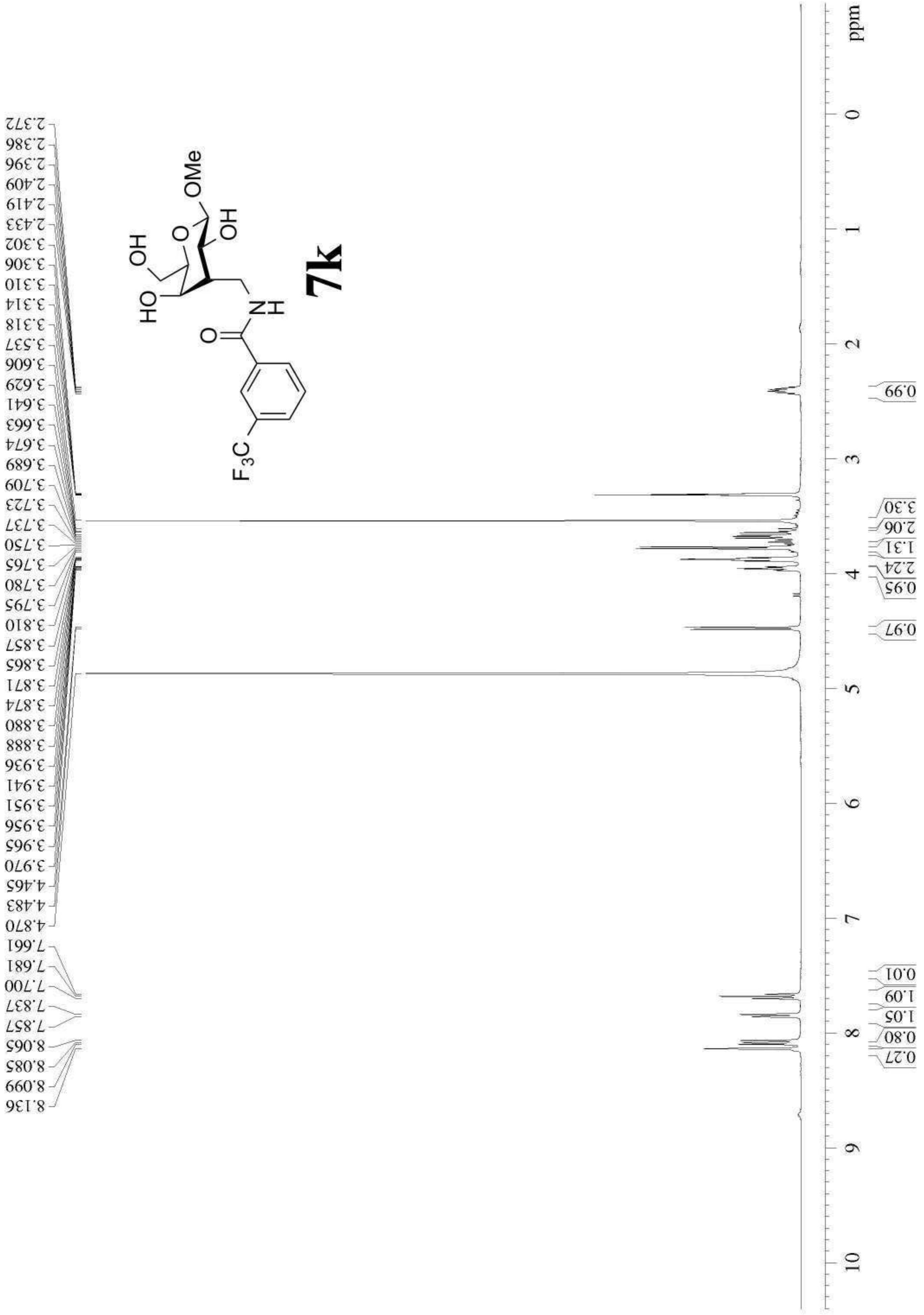
7k
OMe
OH
HO
O
N
H
F3C
2.372
2.386
2.396
2.409
2.419
2.433
3.302
3.306
3.310
3.314
3.318
3.537
3.606
3.629
3.641
3.663
3.674
3.689
3.709
3.723
3.737
3.750
3.765
3.780
3.795
3.810
3.857
3.865
3.871
3.874
3.880
3.888
3.936
3.941
3.951
3.956
3.965
3.970
4.465
4.483
4.870
7.661
7.681
7.700
7.837
7.857
8.065
8.085
8.099
8.136
0.99
3.30
2.06
1.31
2.24
0.95
0.97
0.01
1.09
1.05
0.80
0.27
ppm
0
1
2
3
4
5
6
7
8
9
10

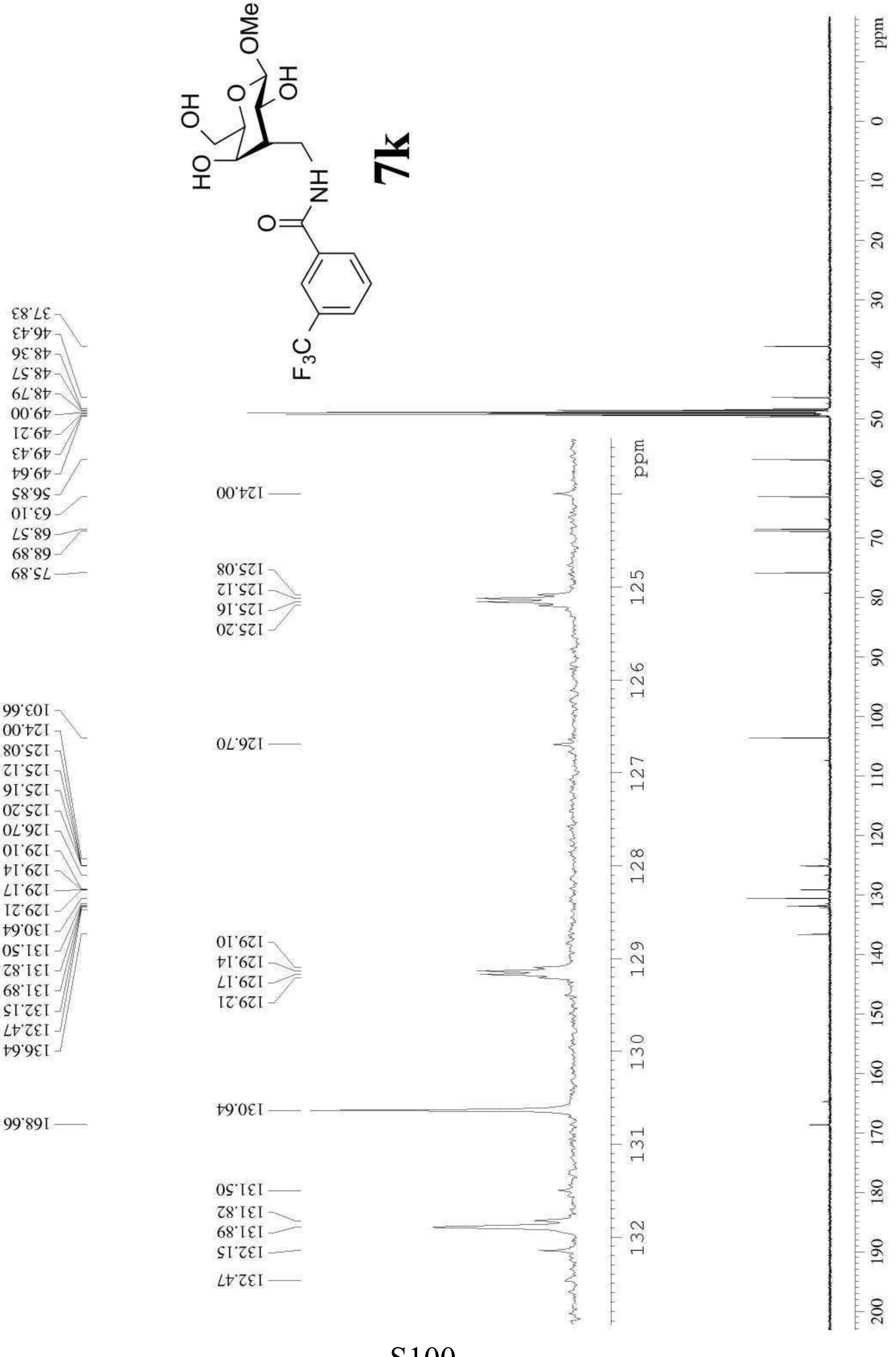
7k
37.83
46.43
48.36
48.57
48.79
49.00
49.21
49.43
49.64
56.85
63.10
68.57
68.89
75.89
103.66
124.00
125.08
125.12
125.16
125.20
126.70
129.10
129.14
129.17
129.21
130.64
131.50
131.82
131.89
132.15
132.47
136.64
168.66
ppm

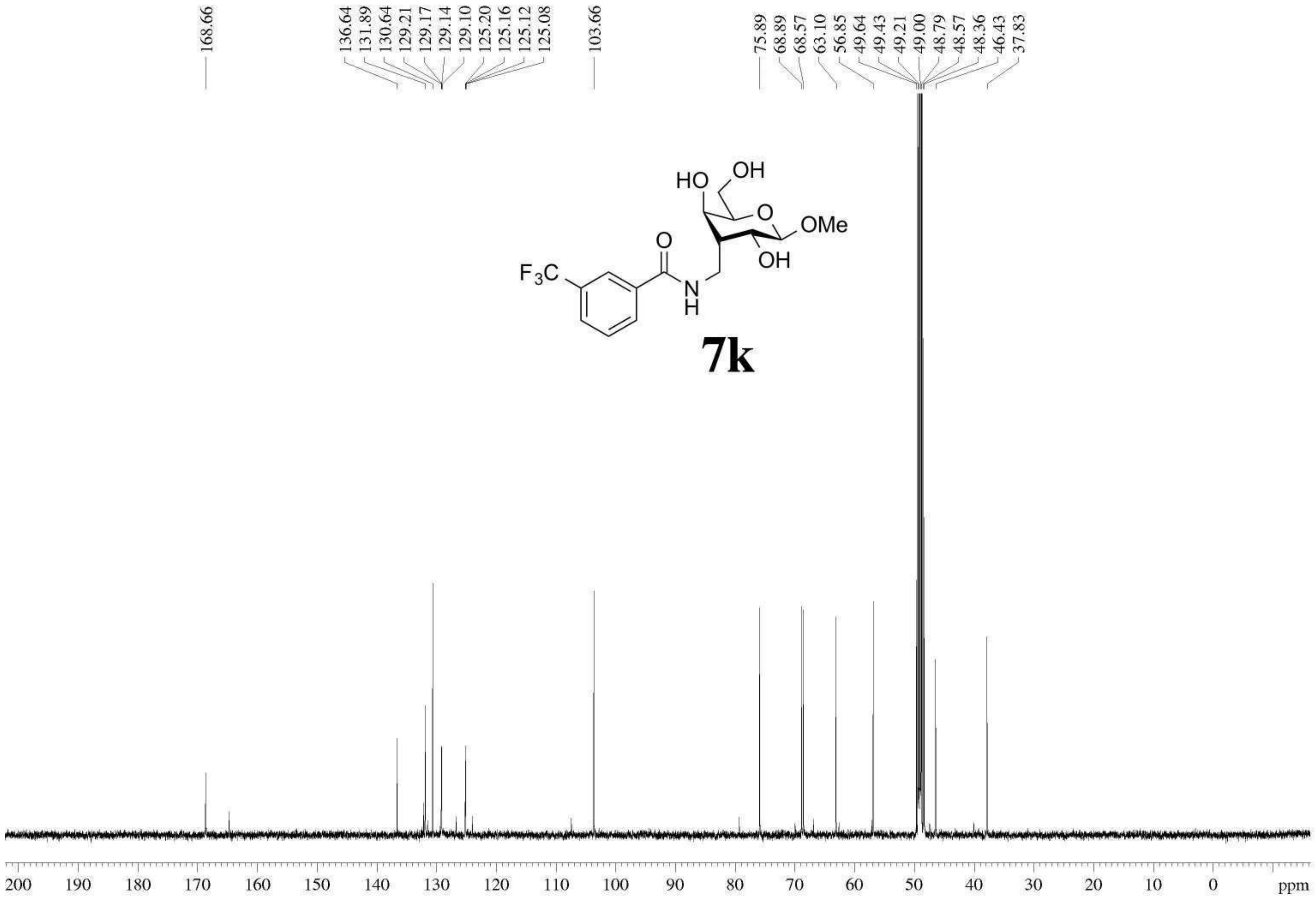
168.66
136.64
131.89
130.64
129.21
129.17
129.14
129.10
125.20
125.16
125.12
125.08
103.66
75.89
68.89
68.57
63.10
56.85
49.64
49.43
49.21
49.00
48.79
48.57
48.36
46.43
37.83
HO
OH
O
OMe
OH
F3C
O
N
H
7k
200 190 180 170 160 150 140 130 120 110 100 90 80 70 60 50 40 30 20 10 0 ppm

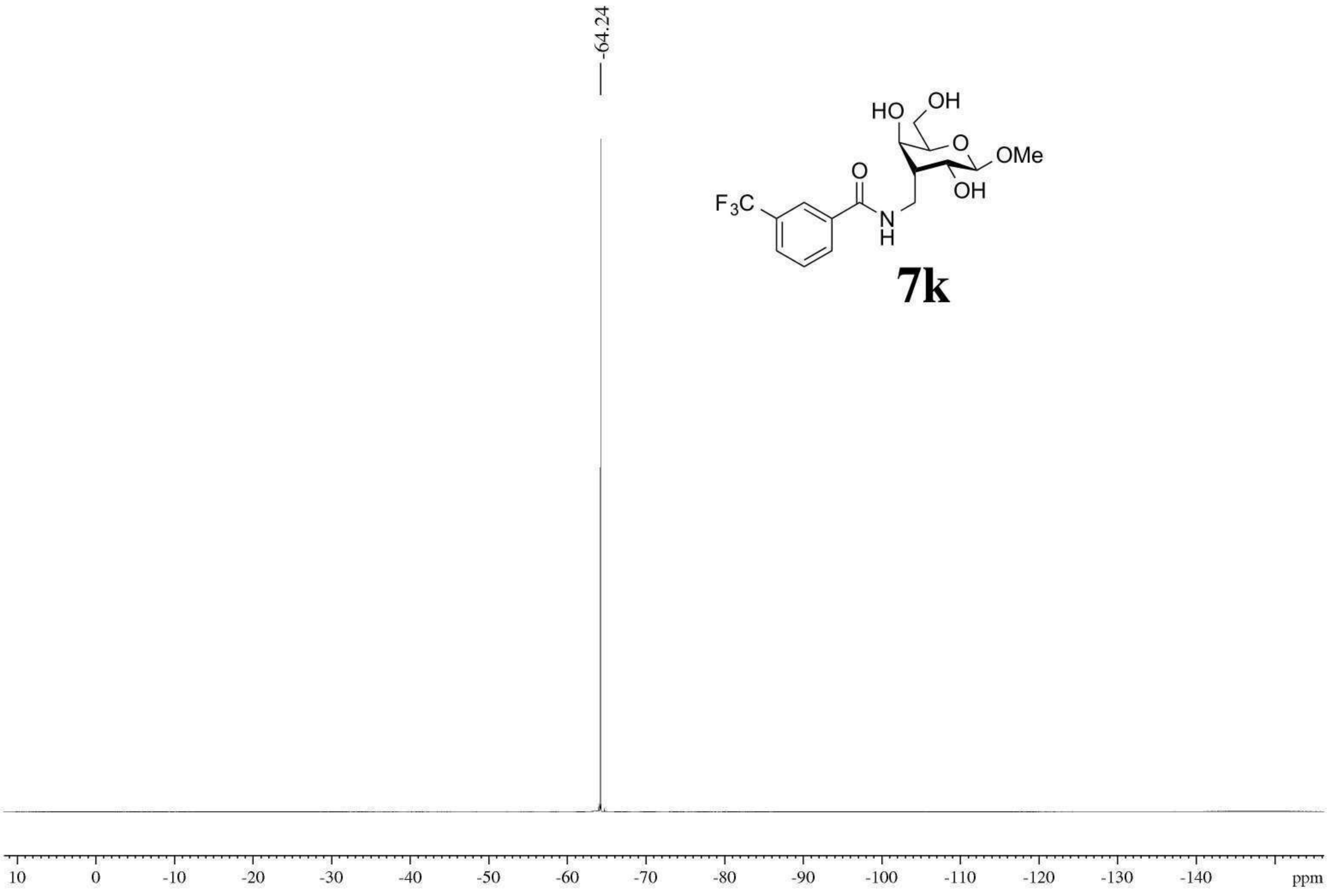
-64.24
HO
OH
O
OMe
OH
O
F3C
N
H
7k
10
0
-10
-20
-30
-40
-50
-60
-70
-80
-90
-100
-110
-120
-130
-140
ppm

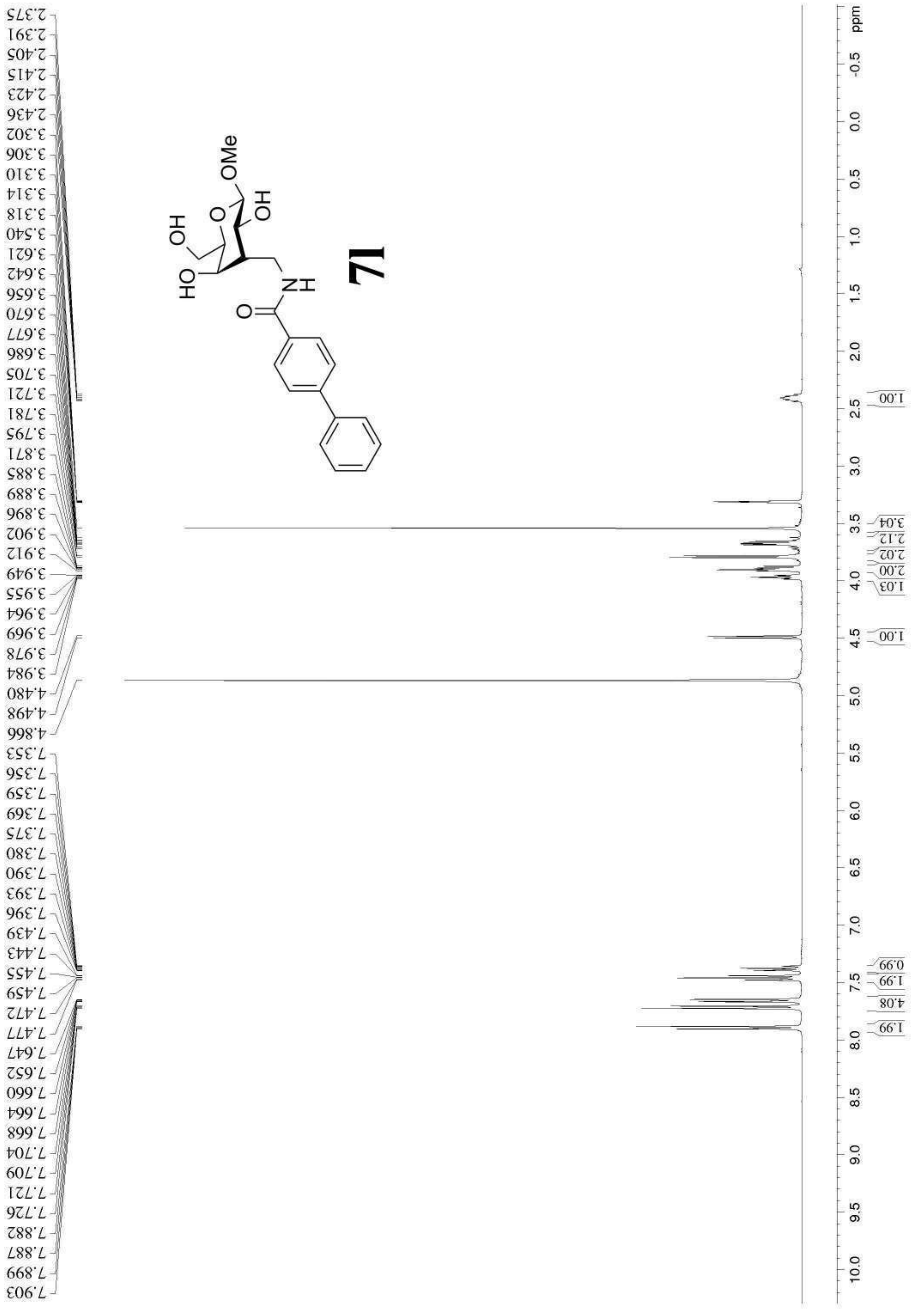
71
OMe
OH
HO
HO
O
N
H
O
2.375
2.391
2.405
2.415
2.423
2.436
3.302
3.306
3.310
3.314
3.318
3.540
3.621
3.642
3.656
3.670
3.677
3.686
3.705
3.721
3.781
3.795
3.871
3.885
3.889
3.896
3.902
3.912
3.949
3.955
3.964
3.969
3.978
3.984
4.480
4.498
4.866
7.353
7.356
7.359
7.369
7.375
7.380
7.390
7.393
7.396
7.439
7.443
7.455
7.459
7.472
7.477
7.647
7.652
7.660
7.664
7.668
7.704
7.709
7.721
7.726
7.882
7.887
7.899
7.903
1.00
3.04
2.12
2.02
2.00
1.03
1.00
0.99
1.99
4.08
1.99
ppm
-0.5
0.0
0.5
1.0
1.5
2.0
2.5
3.0
3.5
4.0
4.5
5.0
5.5
6.0
6.5
7.0
7.5
8.0
8.5
9.0
9.5
10.0

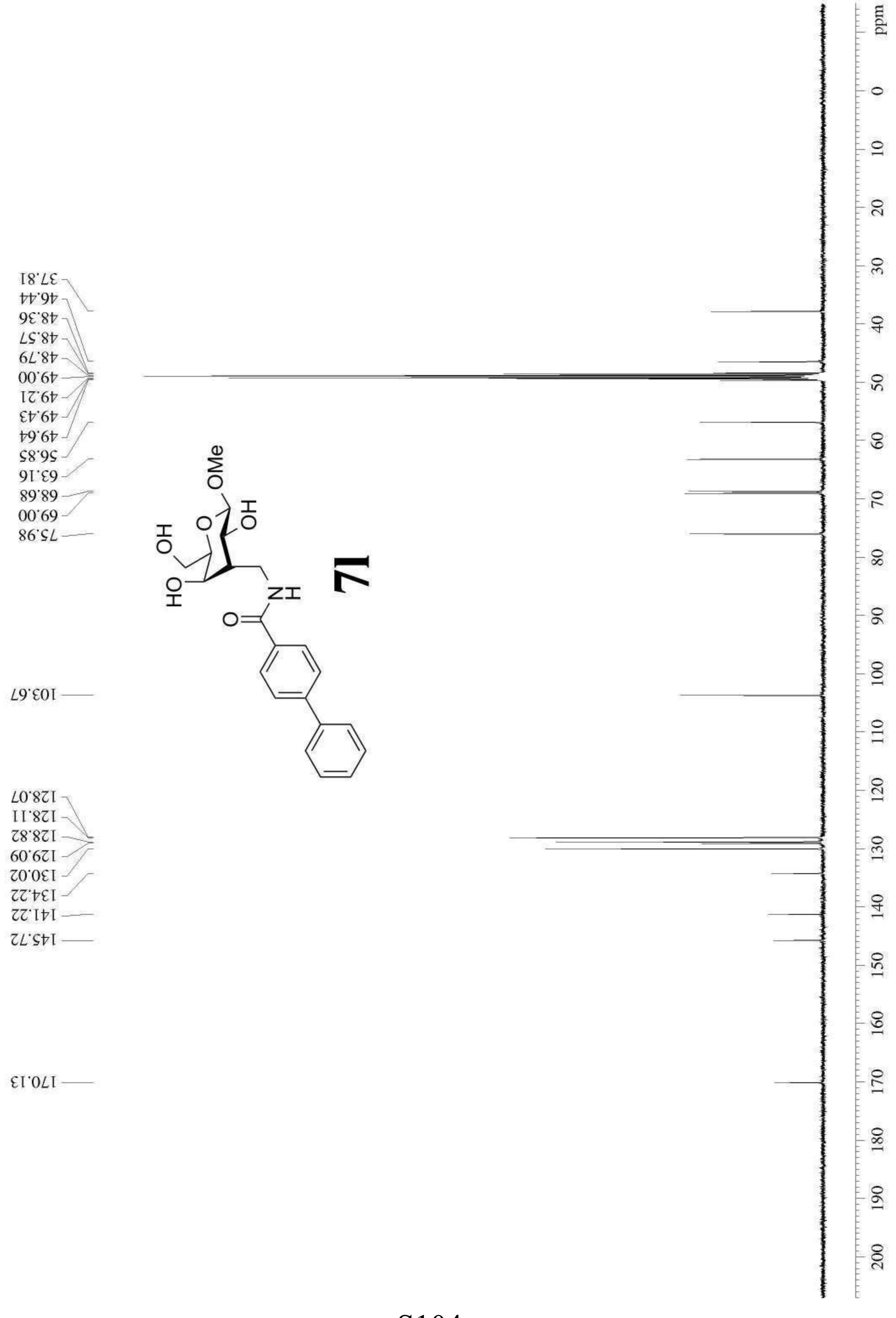
37.81
46.44
48.36
48.57
48.79
49.00
49.21
49.43
49.64
56.85
63.16
68.68
69.00
75.98
103.67
128.07
128.11
128.82
129.09
130.02
134.22
141.22
145.72
170.13
OMe
HO
O
OH
HO
N
H
O
71
ppm

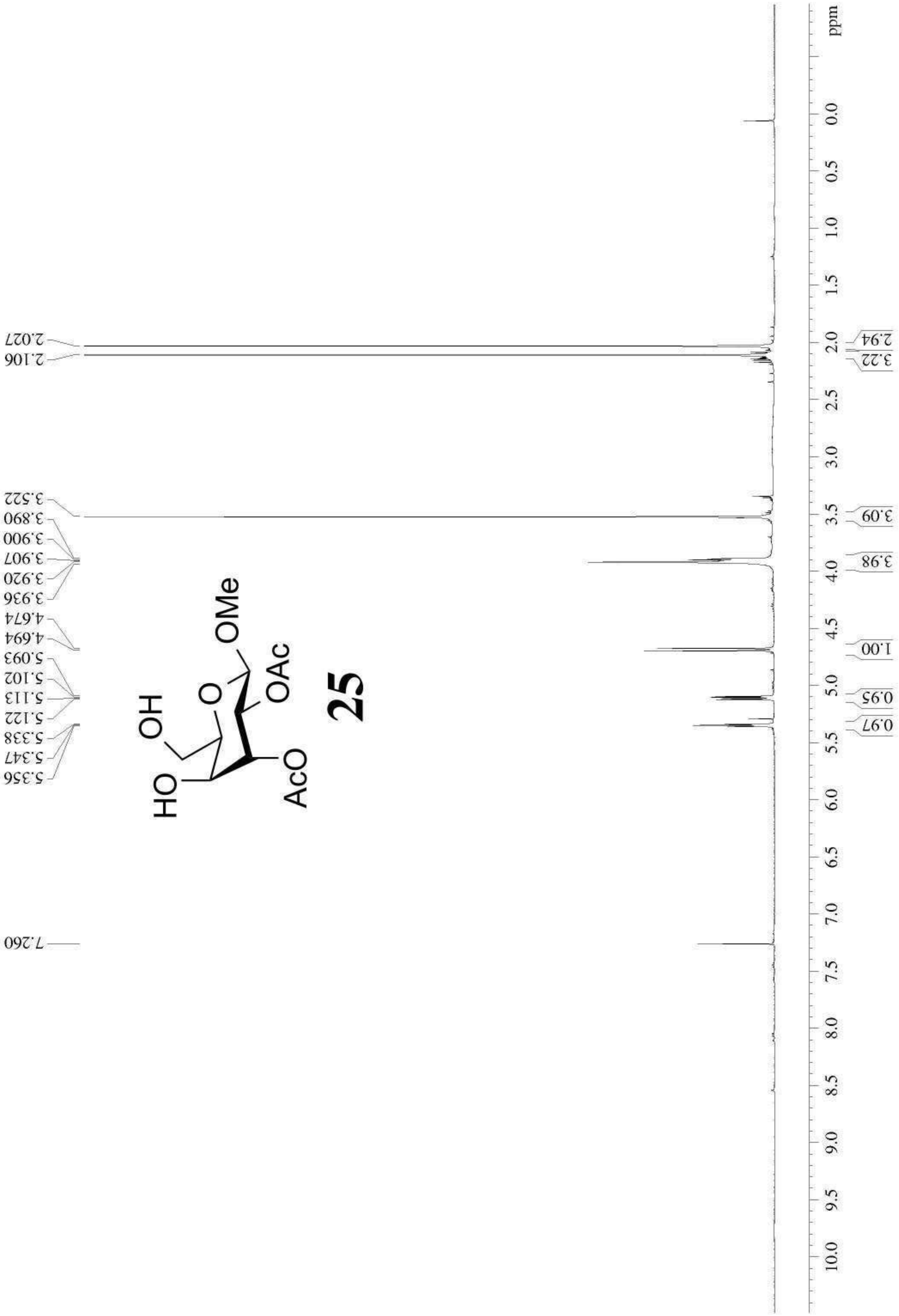
2.027
2.106
3.522
3.890
3.900
3.907
3.920
3.936
4.674
4.694
5.093
5.102
5.113
5.122
5.338
5.347
5.356
7.260
HO
OH
O
OMe
OAc
AcO
25
2.94
3.22
3.09
3.98
1.00
0.95
0.97
ppm
0.0
0.5
1.0
1.5
2.0
2.5
3.0
3.5
4.0
4.5
5.0
5.5
6.0
6.5
7.0
7.5
8.0
8.5
9.0
9.5
10.0

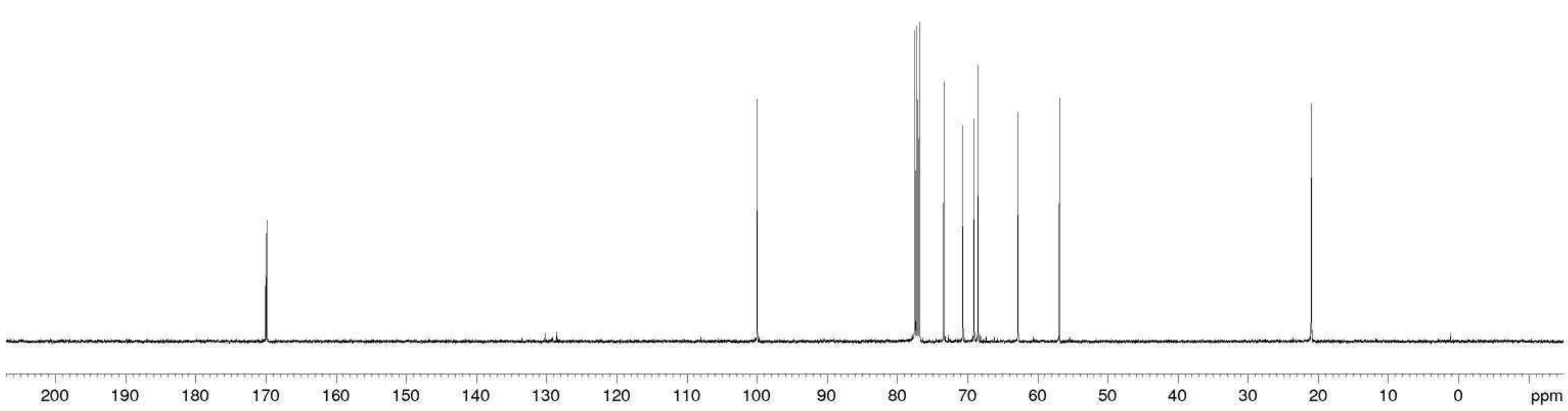
170.00
169.86
99.94
77.48
77.16
76.84
73.33
70.63
69.03
68.44
62.77
56.87
20.98
20.95
200 190 180 170 160 150 140 130 120 110 100 90 80 70 60 50 40 30 20 10 0 ppm

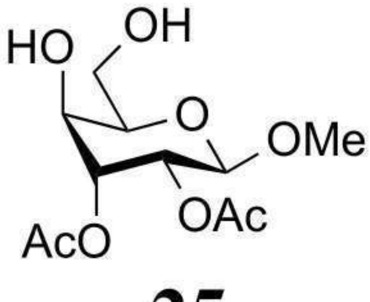
HO
OH
O
OMe
OAc
AcO

25

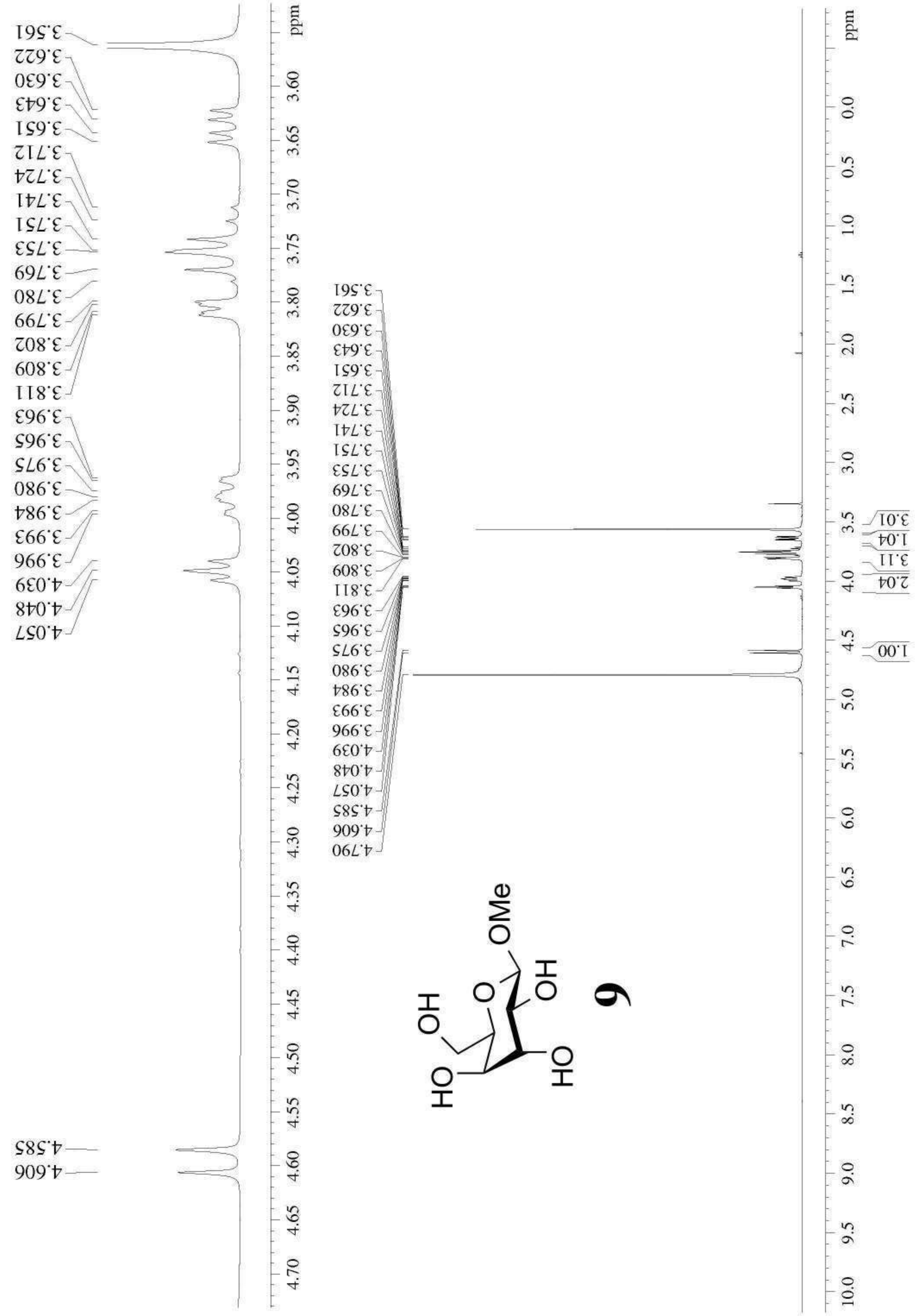
3.561
3.622
3.630
3.643
3.651
3.712
3.724
3.741
3.751
3.753
3.769
3.780
3.799
3.802
3.809
3.811
3.963
3.965
3.975
3.980
3.984
3.993
3.996
4.039
4.048
4.057
4.585
4.606
4.790
ppm
3.01
1.04
3.11
2.04
1.00
OMe
HO
OH
O
9

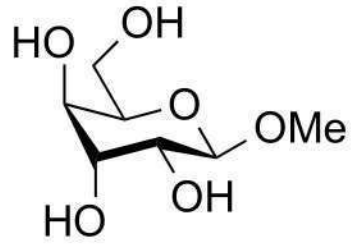

9

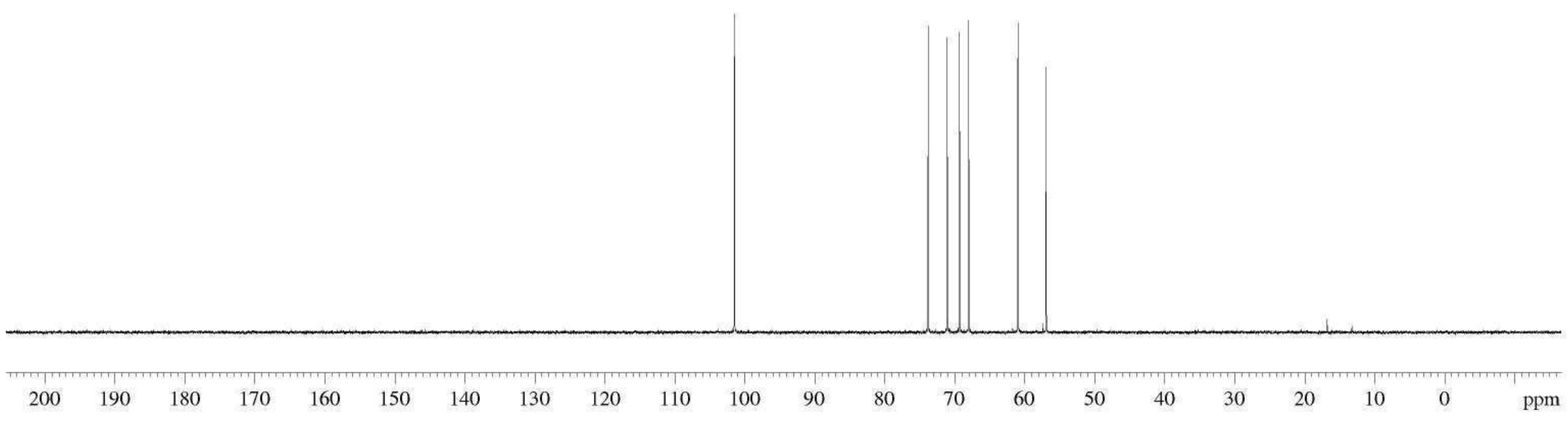
101.457
73.775
71.050
69.291
67.989
60.954
56.909
200 190 180 170 160 150 140 130 120 110 100 90 80 70 60 50 40 30 20 10 0 ppm

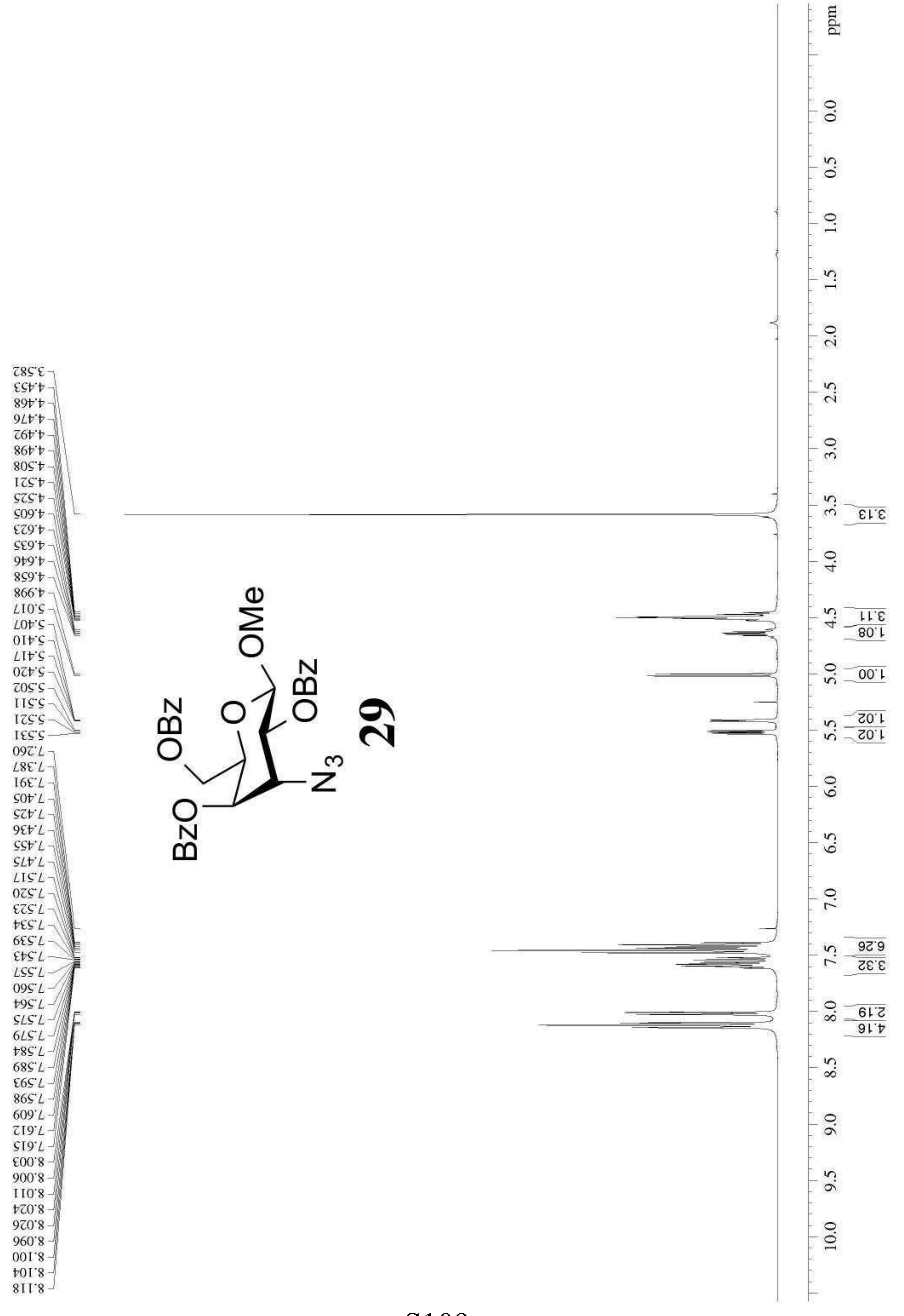
3.582
4.453
4.468
4.476
4.492
4.498
4.508
4.521
4.525
4.605
4.623
4.635
4.646
4.658
4.998
5.017
5.407
5.410
5.417
5.420
5.502
5.511
5.521
5.531
7.260
7.387
7.391
7.405
7.425
7.436
7.455
7.475
7.517
7.520
7.523
7.534
7.539
7.543
7.557
7.560
7.564
7.575
7.579
7.584
7.589
7.593
7.598
7.609
7.612
7.615
8.003
8.006
8.011
8.024
8.026
8.096
8.100
8.104
8.118
OBz
BzO
O
OMe
OBz
N3
29
3.13
3.11
1.08
1.00
1.02
1.02
6.26
3.32
2.19
4.16
ppm
0.0
0.5
1.0
1.5
2.0
2.5
3.0
3.5
4.0
4.5
5.0
5.5
6.0
6.5
7.0
7.5
8.0
8.5
9.0
9.5
10.0

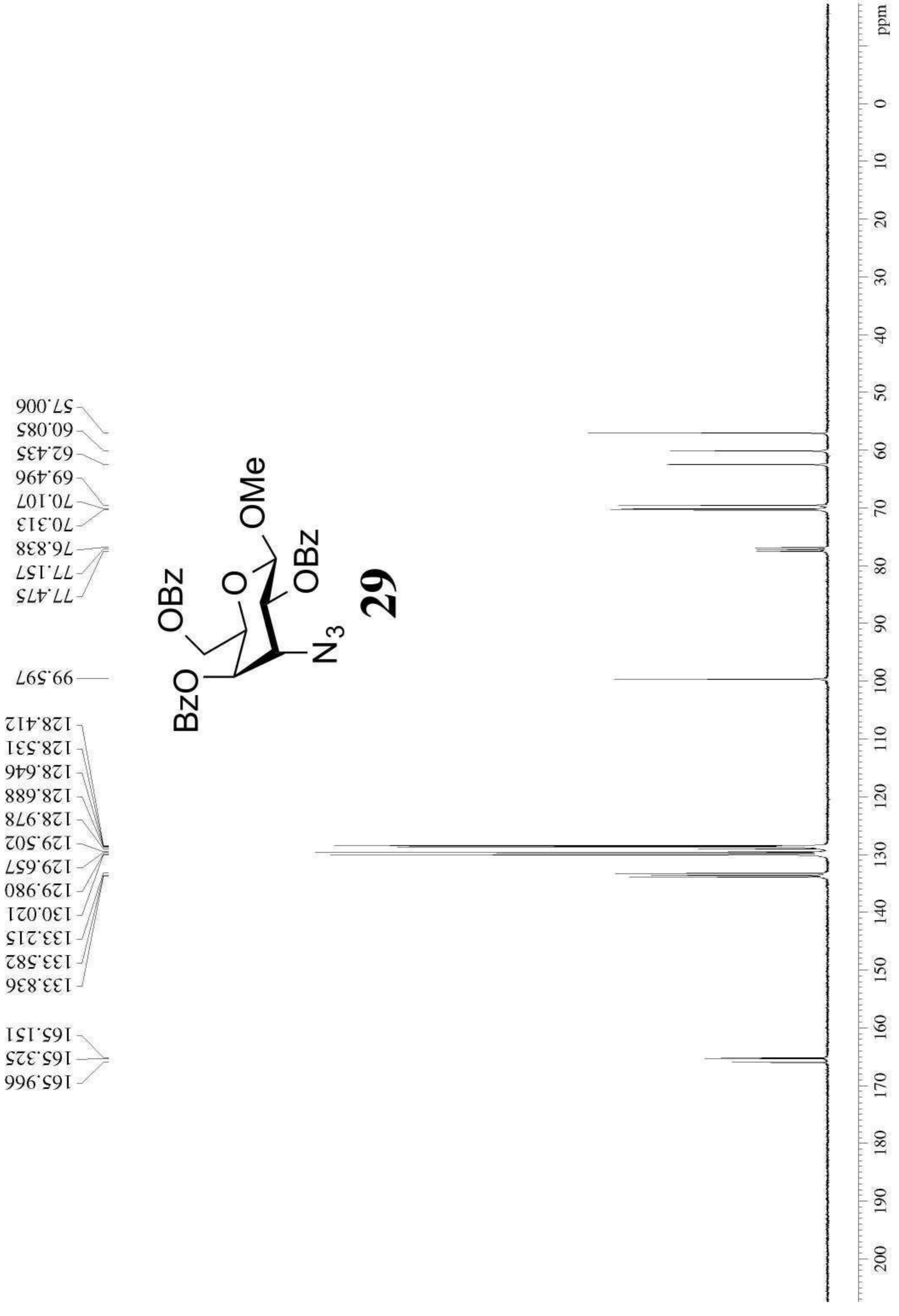
57.006
60.085
62.435
69.496
70.107
70.313
76.838
77.157
77.475
99.597
128.412
128.531
128.646
128.688
128.978
129.502
129.657
129.980
130.021
133.215
133.582
133.836
165.151
165.325
165.966
OBz
OMe
O
BzO
OBz
N_3
29
ppm

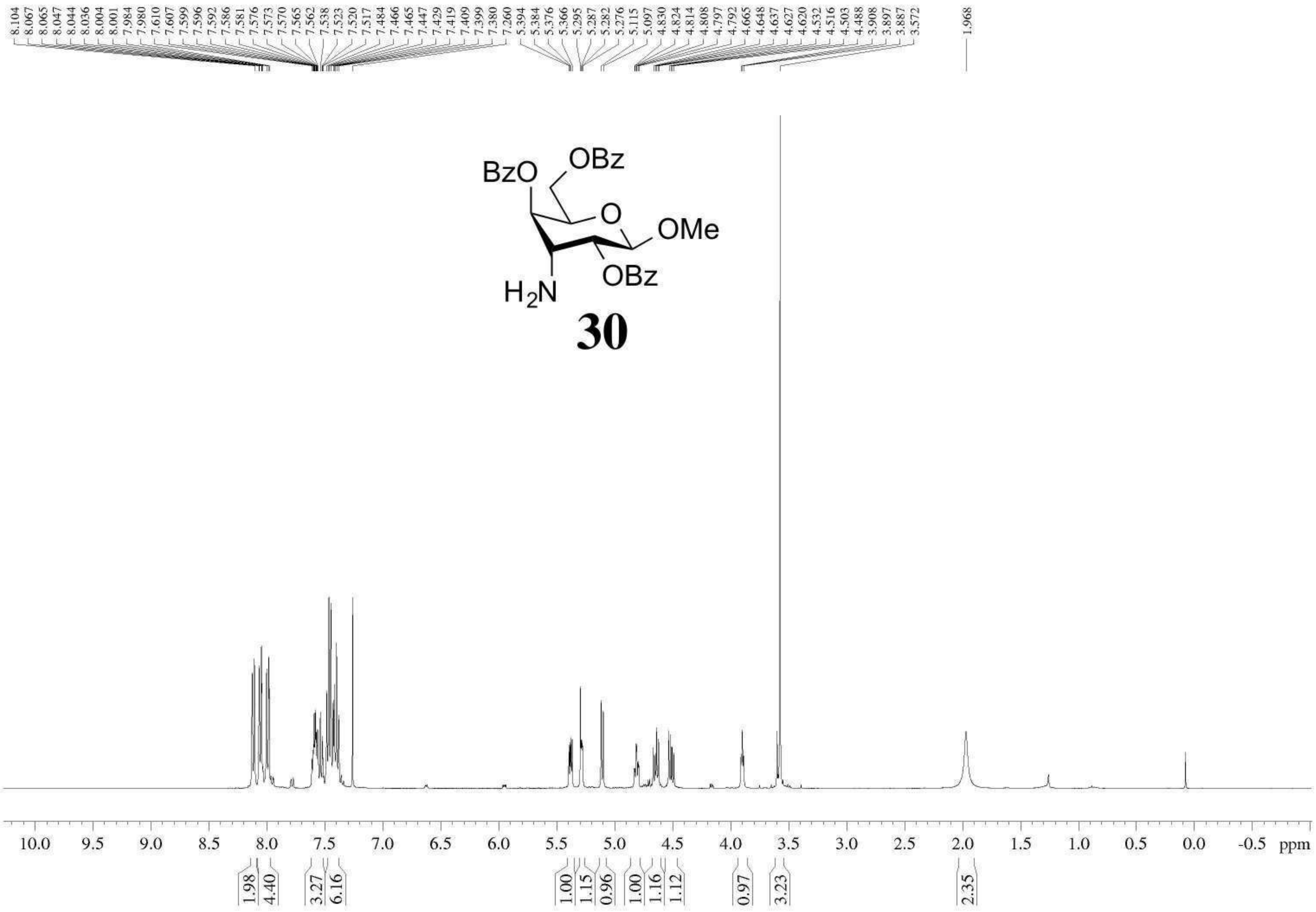
8.104 8.067 8.065 8.047 8.044 8.036 8.004 8.001 7.984 7.980 7.610 7.607 7.599 7.596 7.592 7.586 7.581 7.576 7.573 7.570 7.565 7.562 7.538 7.523 7.520 7.517 7.484 7.466 7.465 7.447 7.429 7.419 7.409 7.399 7.380 7.260 5.394 5.384 5.376 5.366 5.295 5.287 5.282 5.276 5.115 5.097 4.830 4.824 4.814 4.808 4.797 4.792 4.665 4.648 4.637 4.627 4.620 4.532 4.516 4.503 4.488 3.908 3.897 3.887 3.572
1.968
BzO
OBz
O
OMe
OBz
H_2N
10.0 9.5 9.0 8.5 8.0 7.5 7.0 6.5 6.0 5.5 5.0 4.5 4.0 3.5 3.0 2.5 2.0 1.5 1.0 0.5 0.0 -0.5 ppm
1.98 4.40 3.27 6.16 1.00 1.15 0.96 1.00 1.16 1.12 0.97 3.23 2.35

30

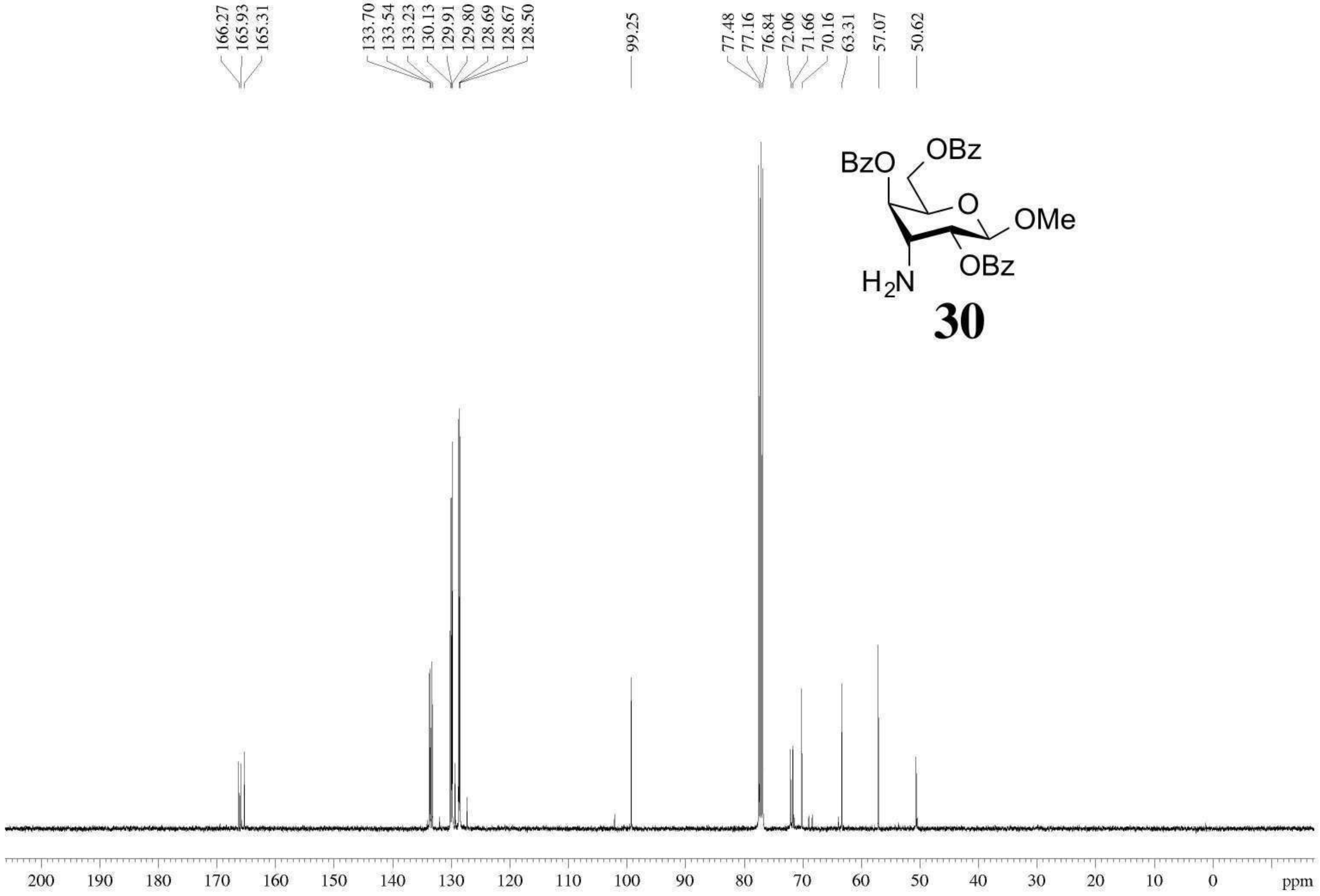
166.27
165.93
165.31
133.70
133.54
133.23
130.13
129.91
129.80
128.69
128.67
128.50
99.25
77.48
77.16
76.84
72.06
71.66
70.16
63.31
57.07
50.62
BzO
OBz
O
OMe
OBz
H_2N
30
200
190
180
170
160
150
140
130
120
110
100
90
80
70
60
50
40
30
20
10
0
ppm

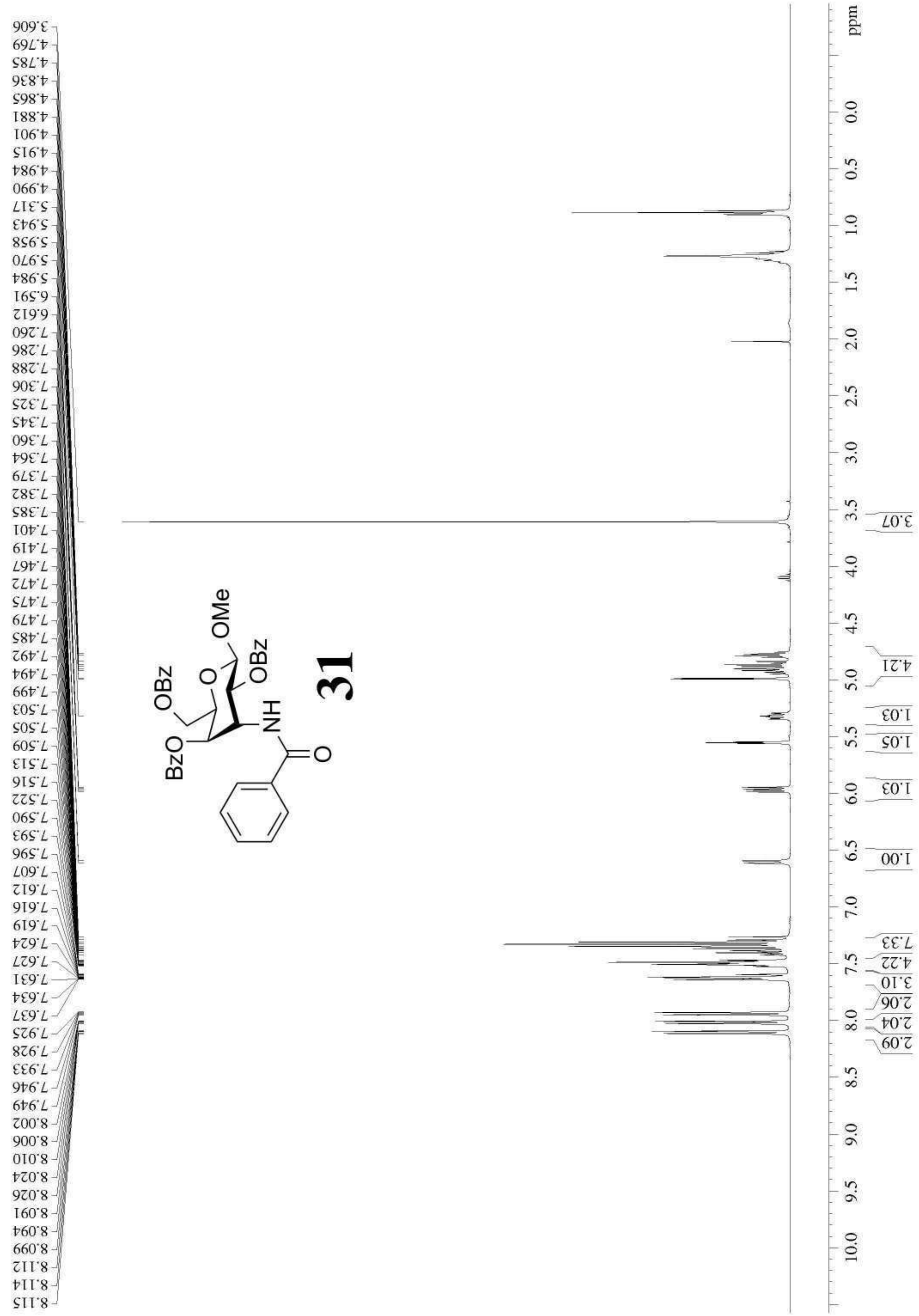
3.606
4.769
4.785
4.836
4.865
4.881
4.901
4.915
4.984
4.990
5.317
5.943
5.958
5.970
5.984
6.591
6.612
7.260
7.286
7.288
7.306
7.325
7.345
7.360
7.364
7.379
7.382
7.385
7.401
7.419
7.467
7.472
7.475
7.479
7.485
7.492
7.494
7.499
7.503
7.505
7.509
7.513
7.516
7.522
7.590
7.593
7.596
7.607
7.612
7.616
7.619
7.624
7.627
7.631
7.634
7.637
7.925
7.928
7.933
7.946
7.949
8.002
8.006
8.010
8.024
8.026
8.091
8.094
8.099
8.112
8.114
8.115
OBz
OMe
BzO
O
NH
OBz
O
31
3.07
4.21
1.03
1.05
1.03
1.00
7.33
4.22
3.10
2.06
2.04
2.09
ppm
0.0
0.5
1.0
1.5
2.0
2.5
3.0
3.5
4.0
4.5
5.0
5.5
6.0
6.5
7.0
7.5
8.0
8.5
9.0
9.5
10.0

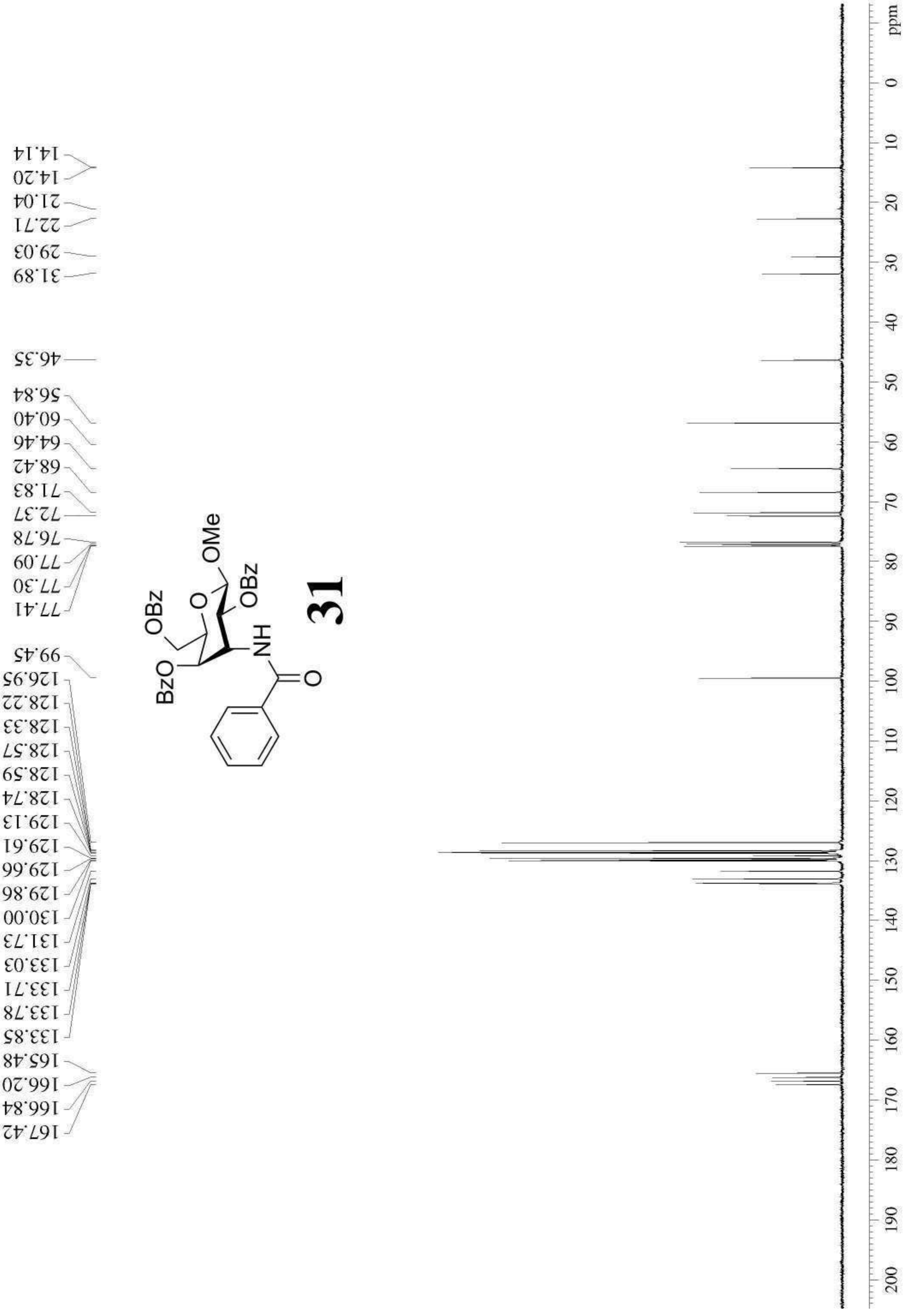
14.14
14.20
21.04
22.71
29.03
31.89
46.35
56.84
60.40
64.46
68.42
71.83
72.37
76.78
77.09
77.30
77.41
99.45
126.95
128.22
128.33
128.57
128.59
128.74
129.13
129.61
129.66
129.86
130.00
131.73
133.03
133.71
133.78
133.85
165.48
166.20
166.84
167.42
OBz
BzO
O
OMe
OBz
NH
O
31
ppm
0
10
20
30
40
50
60
70
80
90
100
110
120
130
140
150
160
170
180
190
200

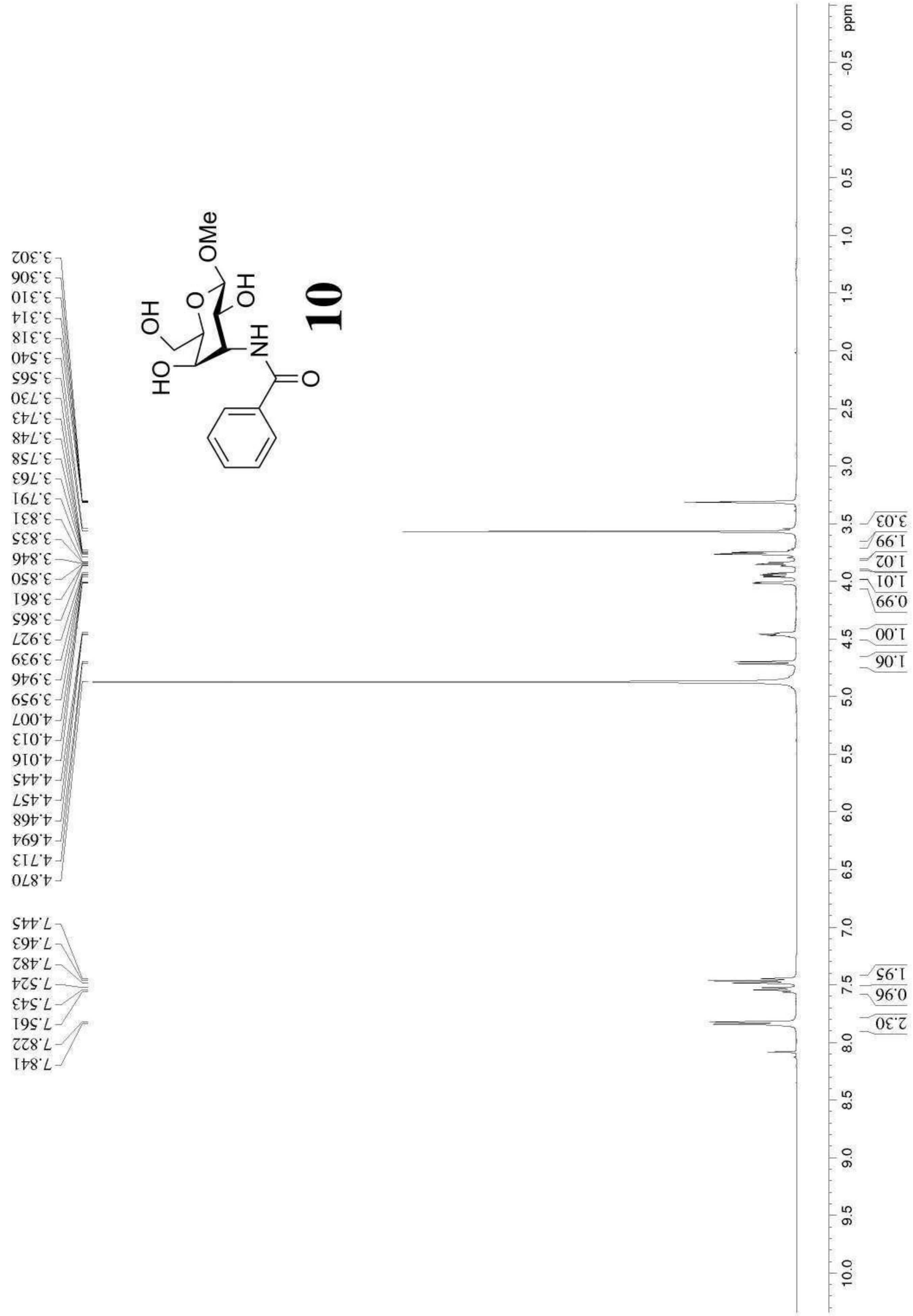
3.302
3.306
3.310
3.314
3.318
3.540
3.565
3.730
3.743
3.748
3.758
3.763
3.791
3.831
3.835
3.846
3.850
3.861
3.865
3.927
3.939
3.946
3.959
4.007
4.013
4.016
4.445
4.457
4.468
4.694
4.713
4.870
7.445
7.463
7.482
7.524
7.543
7.561
7.822
7.841
OMe
OH
HO
NH
O
10
3.03
1.99
1.02
1.01
0.99
1.00
1.06
1.95
0.96
2.30
ppm

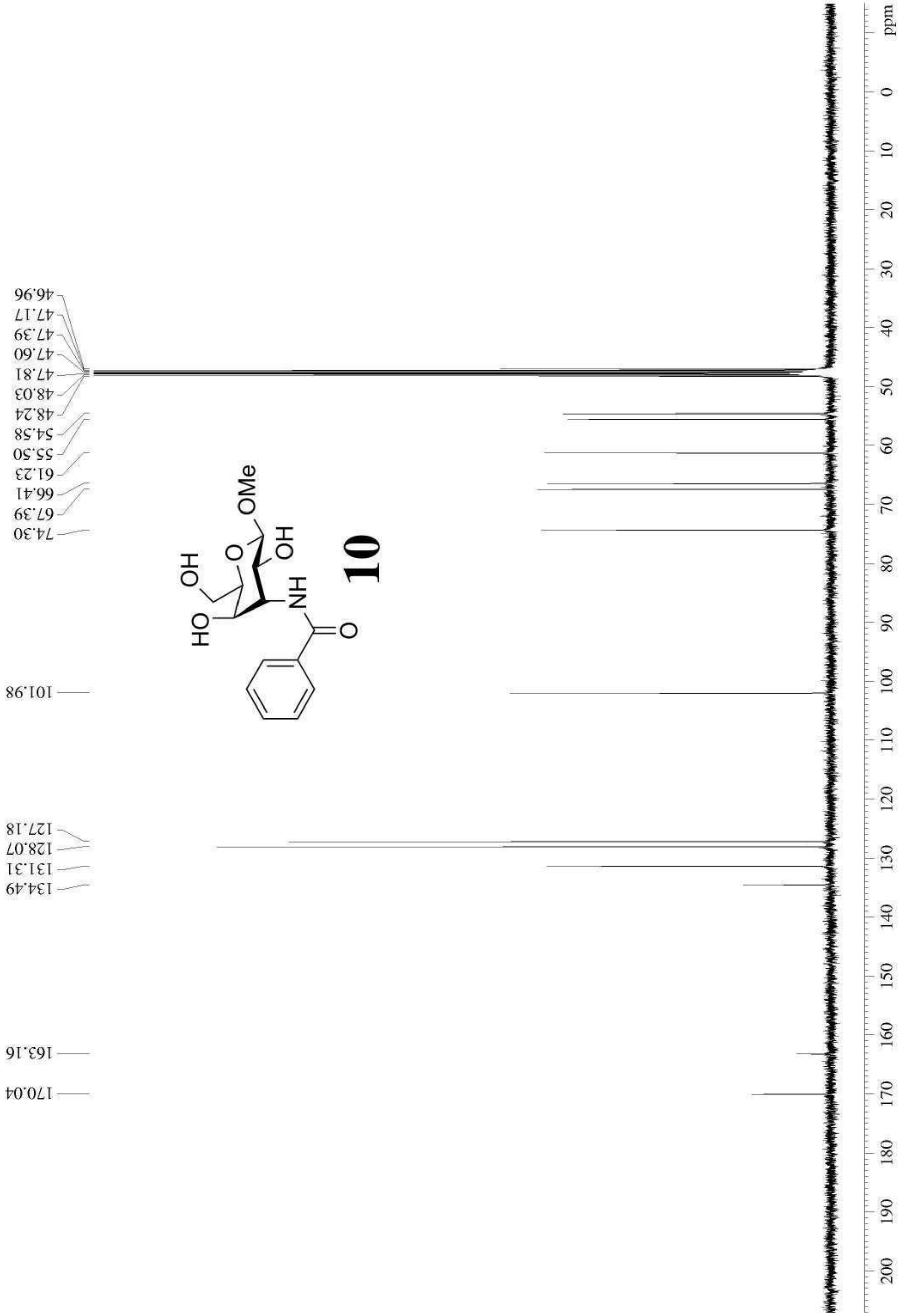
46.96
47.17
47.39
47.60
47.81
48.03
48.24
54.58
55.50
61.23
66.41
67.39
74.30
101.98
127.18
128.07
131.31
134.49
163.16
170.04
OMe
HO
OH
O
OH
NH
O
10
ppm
